# Transcriptome analysis of the pulp of citrus fruitlets suggests that domestication enhanced growth processes and reduced chemical defenses increasing palatability

**DOI:** 10.3389/fpls.2022.982683

**Published:** 2022-09-02

**Authors:** Estela Perez-Roman, Carles Borredá, Francisco R. Tadeo, Manuel Talon

**Affiliations:** Centro de Genómica, Instituto Valenciano de Investigaciones Agrarias, Moncada, Spain

**Keywords:** edible mandarins, fruit acidity, genetic admixture, secondary metabolism, wild citrus

## Abstract

To identify key traits brought about by citrus domestication, we have analyzed the transcriptomes of the pulp of developing fruitlets of inedible wild Ichang papeda (*Citrus ichangensis*), acidic Sun Chu Sha Kat mandarin (*C. reticulata*) and three palatable segregants of a cross between commercial Clementine (*C. x clementina*) and W. Murcott (*C. x reticulata*) mandarins, two pummelo/mandarin admixtures of worldwide distribution. RNA-seq comparison between the wild citrus and the ancestral sour mandarin identified 7267 differentially expressed genes, out of which 2342 were mapped to 117 KEGG pathways. From the remaining genes, a set of 2832 genes was functionally annotated and grouped into 45 user-defined categories. The data suggest that domestication promoted fundamental growth processes to the detriment of the production of chemical defenses, namely, alkaloids, terpenoids, phenylpropanoids, flavonoids, glucosinolates and cyanogenic glucosides. In the papeda, the generation of energy to support a more active secondary metabolism appears to be dependent upon upregulation of glycolysis, fatty acid degradation, Calvin cycle, oxidative phosphorylation, and ATP-citrate lyase and GABA pathways. In the acidic mandarin, downregulation of cytosolic citrate degradation was concomitant with vacuolar citrate accumulation. These changes affected nitrogen and carbon allocation in both species leading to major differences in organoleptic properties since the reduction of unpleasant secondary metabolites increases palatability while acidity reduces acceptability. The comparison between the segregants and the acidic mandarin identified 357 transcripts characterized by the occurrence in the three segregants of additional downregulation of secondary metabolites and basic structural cell wall components. The segregants also showed upregulation of genes involved in the synthesis of methyl anthranilate and furaneol, key substances of pleasant fruity aroma and flavor, and of sugar transporters relevant for sugar accumulation. Transcriptome and qPCR analysis in developing and ripe fruit of a set of genes previously associated with citric acid accumulation, demonstrated that lower acidity is linked to downregulation of these regulatory genes in the segregants. The results suggest that the transition of inedible papeda to sour mandarin implicated drastic gene expression reprograming of pivotal pathways of the primary and secondary metabolism, while palatable mandarins evolved through progressive refining of palatability properties, especially acidity.

## Introduction

Our current knowledge on citrus domestication is still very unprecise (Deng et al., 2020; Kalita et al., 2021; Rao et al., 2021). During the last years, most attention has been paid to the identification of genes or gene families impacting palatability traits, fruit characteristics and reproductive behavior. Among the palatability traits, wide evidence indicates that domestication has modulated pivotal genes regulating major components of citrus flavor, such as acidity (Butelli et al., 2019; Strazzer et al., 2019; Feng et al., 2021; Borredá et al., 2022), bitterness (Chen et al., 2021) or sweetness (Li et al., 2019; Feng et al., 2021). Domestication also appears to have reduced certain chemical defenses in citrus (Gonzalez-Ibeas et al., 2021a,b; Rao et al., 2021), as reported in many crops (Köllner et al., 2008; Yactayo-Chang et al., 2020). It has also been proposed that the increased fruit size of cultivated citrus was acquired during the citrus domestication stage (Wu et al., 2018; Gonzalez-Ibeas et al., 2021b). Several reproductive characteristics closer linked to yield, have also been suggested to be key domestication targets, such as flowering (FT, TFL1 LEAFY or AP1; Rao et al., 2021), self-incompatibility (S-locus; Liang et al., 2020), and apomixis (RWP, Nakano et al., 2012; Wang et al., 2017).

In previous work, we proposed that citrus domestication was led by apomixis and hybridization phenomena (Wu et al., 2018, 2021), a combination that drove reticulate evolution (Hamston et al., 2018) and the formation of a syngameon (Buck and Flores-Rentería, 2022), in the genus *Citrus*. Apomixis, that gives rise to nucellar embryony (polyembryony), allows asexual reproduction of the maternal phenotype. The rise of apomixis in the ancestral mandarin lineage, provided the framework to select through clonal propagation, plants with highly appreciated organoleptic and agronomical characteristics. On the other hand, all edible citrus bear unmistakable hybridization signatures of ancestral mandarins and pummelos (Wu et al., 2014, 2018), indicating that relevant introgressed traits were selected and fixed during domestication. During the last centuries, a myriad of crosses between ancestral hybrids and admixtures, gave rise to the current basic types of edible citrus that were progressively improved through somatic mutations and recurrent selection (Talon et al., 2020). We have also used genomic and transcriptomic analyses on wild and domesticated citrus to discriminate major determinants of evolution and domestication (Gonzalez-Ibeas et al., 2021a,b) and to identify genes involved in relevant physiological processes for domestication (Borredá et al., 2022).

In the current work, we follow this approach comparing gene expression in the fruitlet pulp of wild inedible Ichang papeda (ICH; *C. ichangensis* Swingle), acidic Sun Chu Sha Kat mandarin (SCM; *C. reticulata*, Blanco; *C. erythrosa* Tanaka) and three palatable genetic admixtures derived from a cross between two modern commercial mandarins, namely, Clementine (CLM; *C. x clementina* Hort. ex Tanaka) and W. Murcott (WMU, *C. x reticulata* Blanco). Recent developments suggest that ICH, that is considered one of the most primitive wild forms of citrus (Swingle and Reece, 1967; Yang et al., 2017), split from the main citrus clade around 7 million years (Wu et al., 2018). The ancestor of SCM probably appeared during the last 1.4 million years, after the divergence of the two main subspecies of mainland Asian mandarins (Wu et al., 2021). The main objective of this study was to provide insights on the genetic regulation of major biological processes affected by domestication in citrus.

## Materials and methods

### Plant material and sample processing

The plant materials used in this work were wild inedible Ichang papeda (ICH; *C. ichangensis* Swingle), acidic Sun Chu Sha Kat mandarin (SCM; *C. reticulata*, Blanco; *C. erythrosa* Tanaka) and three palatable genetic admixtures (S1, S2, and S3) derived from a cross between two modern commercial mandarins, namely, Clementine (CLM; *C. x clementina* Hort. ex Tanaka) and W. Murcott (WMU, *C. x reticulata* Blanco). Developing fruitlets were harvested from adult trees grown under normal culture practices at the IVIA germplasm bank and experimental fields, following the protocol described in Cercós et al. (2006). In essence, homogeneous fruits were selected by uniformity of size, appearance and absence of abiotic and biotic stress symptoms. For the transcriptomic analysis, the pulp of developing fruitlets of those two pure species, ICH and SCM, and the three pummelo/mandarin admixtures, S1, S2, and S3, was collected. Fruitlets were peeled, and flavedo (exocarp) and albedo (mesocarp) discarded. The remaining tissue, the fruit flesh consisting of juice vesicles (endocarp) including the segments with their membranes and vascular bundles, was frozen under liquid nitrogen and stored at –80°C until analyses. Three biological replicates of each sample were taken on July, 3rd, 2020 (62 days after anthesis), at the end the cell division stage (phase I) of the development of citrus fruits (Cercós et al., 2006), a critical period for the establishment of pivotal characteristics of the citrus ripe fruits (Terol et al., 2019). A second set of pulp samples to be used in qPCR analysis determinations, consisting exclusively of juice vesicles, was collected from ripening fruits on November 21st (190 days after anthesis).

### Phenotypical data

In order to study correlation between gene expression and acidity and because acid levels do not still show accumulation at early July, fruits were also collected once a month, during the ripening period (from October to February), when the maximum acid accumulation that generally occurs in September, has already taken place (Cercós et al., 2006). Biochemical parameters (acidity, °Brix, and maturity index) were registered in these samples. Citric acid equivalents (g/l) were determined by titration with 0.1 M sodium hydroxide and a phenolphthalein indicator. Soluble sugar content was measured with a refractometer ATAGO PR-1.

### RNA extraction, library preparation and sequencing

Total RNA from pulp samples was extracted with acid phenol and precipitated with lithium chloride. Three biological replicates were used for each sample. Library preparation and sequencing were carried out by a commercial service following standard protocols. Essentially, samples enriched in mRNA were randomly fragmented and cDNA synthesized. After adapter ligation, size selection and PCR enrichment, samples were sequenced in an Illumina NovaSeq 6000 platform yielding 150 bp pair-end reads. On average, each biological replicate produced 7.04 Gb of sequence data in 23497582 raw reads.

### RNA-seq read mapping and differential expressed genes analysis

The *C. clementina* reference genome (Wu et al., 2014) and its annotation data, as reported at the NCBI (National Center for Biotechnology Information [NCBI], 1988), were used for RNA analysis. First, raw reads were mapped using STARv2.7.6 (Dobin et al., 2013). Read counts were computed by featureCounts function in the Rsubread package (Liao et al., 2014). DESeq2 1.26 (Love et al., 2014) was used for expression analysis following author’s recommendation. Differential expressed genes (DEGs) were detected performing pair-wise comparisons of ICH and each one of segregants, against SCM. In these comparisons, the three biological replicates of each sample were treated as a group. Two combinations of Log_2_ Fold Change and alpha thresholds, either Log_2_FC = 0.58 and α = 0.05 or Log_2_FC = 1 and α = 0.01, were set for expression analysis.

### Pathway analysis

The set of DEGs between ICH-SCM resulting of the comparison using the softer thresholds, Log_2_FC = 0.58 and α = 0.05, was annotated using *C. clementina* KEGG data (Kanehisa et al., 2016). Enzymatic information and KEGG identifiers of those DEGs that mapped to the set of KEGG metabolic pathways were retrieve and Pathview 4.1 (Luo and Brouwer, 2013) was used to represent the differentially expressed. For easy interpretation, differential expression was represented by three uniform colors (up, red; blue, down; yellow, undetermined) without indication of the Log_2_FC. We reserved the term “undetermined” expression for genes that share the same functional annotation (i.e., same KEGG identifier), but showed opposite expression trends, as long as one of the biological replicates reached at least 100 reads. In a few cases where both the number of genes and reads with the same expression trend were unequivocally higher, the expression displayed by these genes was considered the dominant expression.

### Functional annotation and category assignment

In order to functionally characterize and categorize relevant DEGs found in the previous ICH-SCM comparison, a second analysis using more restrictive thresholds, Log_2_FC = 1 and α = 0.01, was performed. From the set of DEGs obtained in this second comparison, genes that were included in previous KEGG analyses and genes that did not reach 100 reads in at least one replicate, were removed. In a first step, the remaining DEGs were functionally annotated according to the Uniprot database (UniProt Consortium, 2021) and current literature. The DEGs were grouped into 45 user-defined categories, according to their involvement in major physiological, biochemical and genetic processes, and these categories were in turn split twice as much in subsequent subcategories. Genes that could not been assigned to a specific process were grouped in clusters defined by their molecular function. A group of uncharacterized DEGs, with general and ambiguous annotations or with undescribed assignments in plants was also created.

In another experiment, DEGs between the three palatable segregants and SCM were identified. Pairwise comparisons of each segregant, S1, S2, and S3, against SCM (Log_2_FC = 0.58 and α = 0.05) were parsed as above and genes that did not reach at least 100 reads in one of the biological replicates were similarly filtered out. DEGs were grouped into the aforementioned categories and common DEGs in the three segregants were included in a single list and manually characterized.

### RT-qPCR expression analysis

RT-qPCR was used to validate RNA-seq analysis in the pulp of developing fruitlets (Supplementary Figure 1) and to test expression of target genes in juice vesicles of ripening fruits (November). Two replicates of each sample/gene combination were performed in one-step reaction in a LightCycler Instrument. Each sample was incubated with the reverse transcriptase MultiScribe (Invitrogen) at 48°C during 30 min and with an RNAse Inhibitor (Applied Biosystems). Reaction mastermix also included LightCycler FastStart DNA Master Plus SYBR Green I kit for amplification step.

Relative gene expression was calculated using ΔΔCt method. CitUBC1 (Merelo et al., 2017) and CitACTIN11 (Strazzer et al., 2019) were used as housekeeping genes for data normalization. All primer sequences used are available in Supplementary Table 1.

### DNA extraction, sequencing and mapping

For each segregant, a sample of fresh leaves was collected and DNA purified using CTAB extraction method. Library preparation and whole genome sequencing (WGS) were carried out by a commercial service following a standard protocol. In short, genomic DNA was randomly sheared into short fragments that, subsequently, were end-repaired, A-tailed and further ligated with Illumina adapters. Fragments with adapters were PCR amplified, size selected, and purified. Sequencing was run in an Illumina NovaSeq 6000 platform yielding 150 bp pair-ended reads. On average, each sample produced 104484177 raw reads, generating 31.35 Gb of sequence data. The sequences were mapped to the *C. clementina* haploid reference (Wu et al., 2014) using BWA-MEM tool (Li, 2013). Map files were sorted and indexed using Samtools (Li et al., 2009). The mean read depth of each segregant was 68.8x, 75.9x, and 70.7x.

WGS data for *C. maxima* (CHP, pummelo) and *C. reticulata* (SCM, mandarin), the two pure species that make up the genome of the three segregants, were retrieved from the Sequence Read Archive database (National Center for Biotechnology Information [NCBI], 1988) and processed as above.

### Variant calling

Variant calling was performed using the GATK-4.0.0.0 software (Van der Auwera et al., 2013). We used the HaplotypeCaller tool to generate single-sample variant call format files that were combined using the CombineGVCF tool to get matrices including the samples of the study. Each site showing a quality value greater than 10 was genotyped by GenotypeGVCF and only calls tagged as SNP were filtered according to a set of standard filters specified in the variant caller practice guide. To include RNA-seq data, input files were reformatted to adapt the alignments that span introns using SplitNCigarReads tool. Raw matrices were filtered to get species informative markers (SIM). To this end, we retrieved sites holding fixed differences between *C. maxima* (CHP) and *C. reticulata* (SCM). The set of diagnostic markers only included sites with different alleles in homozygosis, supported by at least 20 reads.

### Admixture pattern

The above set of SNPs was used to define local ancestry segments along the genome of each segregant. The haplotype (pummelo or mandarin) of the admixture stretches was determined, as in Wu et al. (2018), using windows of 1000 markers. Essentially, for each SIM, the copy number of both ancestral alleles (i.e., 2, 1, or 0) was recorded and the ancestry of the window was inferred from the most frequent one. Stable segments were considered when a minimum of five windows in a row exhibited the same inheritance. Otherwise, the ancestry of the nearest stable block was applied. These data were used to compute the distribution of expressed genes in each segregant. Only genes that were covered with a minimum of 10 reads in any of the biological replicates were considered. Haplotype combinations were named, MA/MA (mandarin/mandarin), PU/MA (pummelo/mandarin), and PU/PU (pummelo/pummelo).

### Allele differential expression

Species informative markers were additionally used to study differentially expressed alleles in PU/MA regions. Only genes with heterozygous sites covered by a minimum average depth of 10x, showing identical genotype in the three biological replicates, were initially considered for this analysis. The final set of cDNAs used in the analysis of allele differential expression included genes, in which approximately the 80% of the SNPs spanning their sequences met these conditions. From this set of target genes, the occurrence of homozygous SIMs was assessed as exclusive expression of a certain allele.

### Admixture validation

We used PCR and Sanger sequencing to validate both a sequence change causing an admixture pattern shift such as crossover, and an event of allele differential expression (Supplementary Figures 2, 3).

## Results

To study differential gene expression as related to domestication in the pulp of developing fruitlets, two independent comparisons were performed. In one of them, the transcriptome of inedible Ichang papeda (ICH) and acidic Sun Chu Sha Kat mandarin (SCM) were contrasted. In a second evaluation, this mandarin was compared with a group formed by three palatable genetic pummelo/mandarin admixtures derived from a cross between the commercial mandarins Clementine and W. Murcott.

### Gene expression in inedible wild Ichang papeda versus acidic Sun Chu Sha Kat mandarin

**FIGURE 1:**
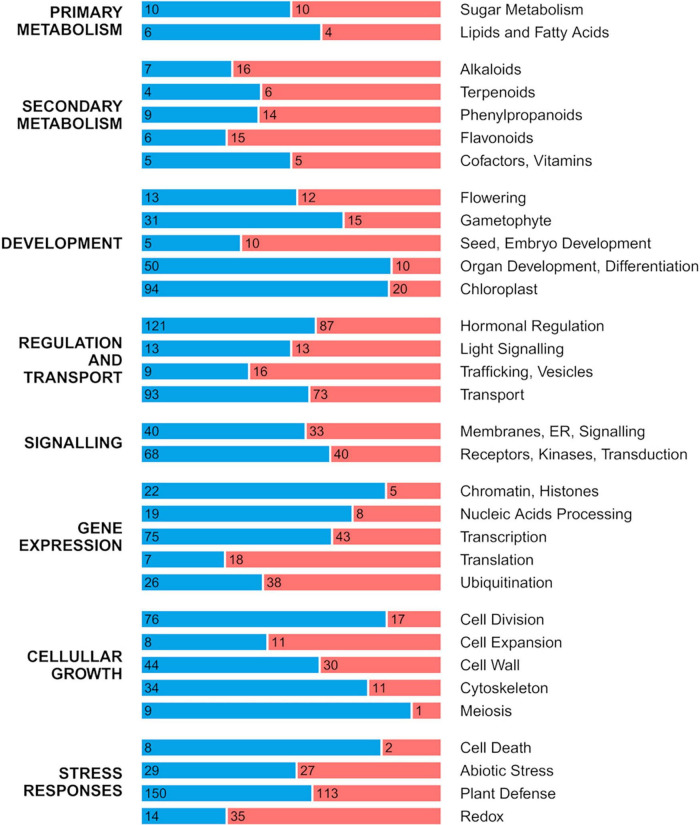
Distribution of up- and downregulated DEGs in the pulp of developing fruitlets of ICH as related to SCM. DEGs were manually classified into eight major groups that were subsequently split in 32 gene categories corresponding to the most populated categories presented in Supplementary Table 3, excluding the group of uncharacterized genes. Length of red and blue stretches represents the frequency of up- and downregulated genes, respectively, while numbers refer to the number of genes included in each category.

The transcriptome comparison between the developing fruitlet pulp of ICH and SCM (Log_2_ Fold Change threshold of 0.58 and alpha of 0.05) identified 7267 DEGs (Supplementary Table 2). Out of this set of genes, 2342 were mapped to 117 pathways, mostly related to metabolism, genetic information and cellular processes (Supplementary Figures 4.001–4.117), as defined in the KEGG database resource (Kanehisa et al., 2016). Genes that did not map to the KEGG collection, were compared using more stringent conditions (Log_2_ Fold Change threshold of 1 and alpha of 0.01). This analysis rendered 2832 DEGs (Supplementary Table 3) that were, first, functionally annotated according to the Uniprot database (UniProt Consortium, 2021), and then grouped into 45 user-defined categories. Figure 1 presents the 32 categories encompassing more than 10 members, excluding the group of uncharacterized processes. This classification overall indicates that only categories included in Secondary Metabolism and categories related to protein metabolism (Translation, Ubiquitination, Trafficking) were upregulated in the papeda while most of those grouped in Development, Regulation and Transport, Signaling, Gene Expression, Cellular Growth and Stress responses were downregulated. Thus, more than 60% of those 2832 DEGs were downregulated in the fruit flesh of ICH, a percentage rather identical to that observed in the group of uncharacterized genes. The results described below are restricted to categories potentially involved in domestication while the description and comments affecting to the rest of results are available in Supplementary Material.

#### Alkaloids

We grouped 23 DEGs into the Alkaloids category (Supplementary Table 3) while the KEGG pathway mapping added several additional genes to this cluster. According to the expression levels of transcripts associated with the synthesis of several principal alkaloids, the data suggest that these compounds are generally upregulated in ICH. In this species, for instance, caffeine synthase is upregulated (LOC112100740). The transcripts of codeine 3-*O*-demethylase, the last step in the biosynthesis of morphine (LOC18036840) in the Isoquinoline Alkaloid Pathway, also was upregulated. Similarly, synthesis of major indol alkaloids, i.e., ajmaline, vinblastine and vincristine and also that of iridoid compounds appears to be favored as suggested by the upregulation of relevant biosynthetic steps controlling the conversions of monoterpenoid precursors of indole alkaloid biosynthesis. These upregulated steps are geraniol 8-hydroxylase (5 genes out of 6), 8-hydroxygeraniol dehydrogenase (LOC18037214), the dehydrogenase involved in the biosynthesis of oxogeranial from hydroxygeraniol and 7-deoxyloganetin glucosyltransferase (LOC18042369), an iridoid glucosyltransferase involved in the synthesis of secologanin, one of the major intermediates in the indole alkaloid biosynthesis. It was also observed that a vinorine synthase (LOC18037906), a gene coding for the acetyltransferase catalyzing the formation of vinorine, a precursor of the monoterpenoid indole alkaloid ajmaline, was also upregulated. The synthesis of nicotine, a major alkaloid derived from nicotinic acid (Supplementary Figure 4.067), however, does not appear to be activated. In the acridone alkaloid biosynthesis, two methanol *O*-anthraniloyltransferases genes (LOC18050765 and LOC18050764), in principle implicated in the synthesis of methyl anthranilate, showed opposite expression tendencies. Likewise, evidence for the upregulation of the tropane biosynthesis, leading to alkaloids such as atropine, hyoscyamine and scopolamine was not obtained. The analysis of gene expression certainly showed upregulation of two tropinone reductase-like genes (LOC18039952 and LOC18039951) but the corresponding proteins do not appear to exhibit tropinone reductase activity (Jirschitzka et al., 2012). Interestingly, synthesis of both dopamine and serotonin (Supplementary Figures 4.033, 4.089), two amides that are considered bioactive alkaloid neurotransmitters, in principle, appear to be downregulated in ICH, since tyrosine/DOPA decarboxylase 5 (aromatic L-amino acid decarboxylase; LOC18043348, Supplementary Table 3, and LOC18046227, EC:4.1.1.28, Supplementary Figures 4.033, 4.087 and Supplementary Table 2) is repressed in this species. This enzyme (EC:4.1.1.28) may render tyramine, tryptamine, dopamine and serotonin. Likewise, a CYP71P1 gene (LOC18041681, EC:1.14.-.-, Supplementary Figure 4.033 and Supplementary Table 2) encoding tryptamine 5-hydroxylase that also catalyzes the conversion of tryptamine to serotonin, is repressed too.

#### Phenylpropanoids

The last steps in the formation of the derived aldehyde and alcohol of major phenylpropanoids (Supplementary Figure 4.081 and Supplementary Table 2), such as cinnamic, coumaric, caffeic, ferulic, hydroxyferulic and sinapic acids, are predominantly upregulated in ICH, as observed for instance, cinnamoyl-CoA reductase (LOC18035881, EC:1.2.1.44). However, trans-cinnamate 4-monooxygenase, CYP73A (LOC18055509, EC:1.14.14.91) and 4-coumarate–CoA ligase (LOC18034975, LOC18050151, and LOC18036308, EC:6.2.1.12) the main steps in the synthesis of *p*-Coumaroyl-Co A, the precursor of flavonoids and the non-flavonoid polyphenols, stilbenoid, diarylheptanoic and gingerol biosynthesis, are downregulated. In this pathway, *trans*-resveratrol di-*O*-methyltransferase (LOC18051743 and LOC18031586, Supplementary Tables 2, 3), the last step in the biosynthesis of the antifungal phytoalexin pterostilbene, is expressed a higher level in the papeda. In Supplementary Table 3, there are listed other DEGs that participate in the regulation of the phenylpropanoid biosynthesis such several members of the Cytochrome P450 71A1 family, that appear to act as trans-cinnamic acid 4-hydrolases, or the MYB family transcription factor PHL11. The enzymes listed in this table, caffeic acid 3-*O*-methyltransferases, caffeoylshikimate esterases-like, cinnamoyl-CoA reductases, and shikimate *O*-hydroxycinnamoyltransferases, that are not mapped at the KEGG pathways, appear in principle to be associated with the phenylpropanoid pathway and at least some of them, with lignin biosynthesis.

#### Flavonoids

In the flavonoid pathway, the synthesis of the pivotal intermediate flavanone naringenin in ICH appears to be downregulated (Supplementary Figure 4.082 and Supplementary Table 2), since chalcone isomerase (LOC18044429, EC:5.5.1.6), was repressed, although there were at least four different chalcone synthetases (LOC18042808, LOC18042812, LOC18033130, and LOC18051925, E.C:2.3.1.74), with opposite genetic expression levels. The synthesis of isoflavonoids (Supplementary Table 3) was mostly characterized by the upregulation of 2-hydroxyisoflavanone dehydratase-like (LOC112096719 and LOC112098436), that catalyze the final step in the formation of the isoflavonoid skeleton rendering daidzein, of isoflavone 4′-*O*-methyltransferase (LOC18053393) involved in the biosynthesis of formononetinin and of isoflavone 2′-hydroxylases (LOC18043085), that mediates the hydroxylation of daidzein and formononetin, to yield 2′-hydroxyisoflavones. In spite of downregulation of naringenin, the formation of the polyphenolic flavonols, kaempferol, quercetin, and myricetin appears to be upregulated (Supplementary Figure 5). Supplementary Table 3, for instance, reports several flavonoid 3′-monooxygenases (LOC18053376) and flavonoid 3′,5′-hydroxylases (LOC18048580) controlling the conversion of naringenin to eriodictyol, dihydrokaempferol, dihydroquercetin and dihydromyricetin, and Supplementary Figure 4.082 shows upregulation of naringenin 3-dioxygenase (LOC18036490, EC:1.14.11.9) and flavonol synthase (LOC18037475, EC:1.14.20.6, Supplementary Table 2), two regulating enzymes of the synthesis of kaempferol, quercetin and myricetin. The synthesis of the anthocyanidins, pelargonidin, cyanidin and delphinidin, and their corresponding anthocyanins (anthocyanidin glycosides; Khoo et al., 2017) was similarly upregulated in the papeda. Supplementary Table 2, for example, shows that two limiting steps in the synthesis of anthocyanidins and anthocyanins, anthocyanidin synthase (LOC18047155, EC:1.14.20.4, Supplementary Figure 4.082) and anthocyanidin 3-*O*-glucosyltransferase (LOC18047244, EC:2.4.1.115, Supplementary Figure 4.083), respectively, were clearly upregulated. In addition, Supplementary Table 3 enumerates a number of upregulated members of several gene families, coumaroyl-CoA:anthocyanidin 3-*O*-glucoside-6′′-*O*-coumaroyltransferase 1 (LOC18050842 and LOC18032737) malonyl-CoA:anthocyanidin 5-*O*-glucoside-6′′-*O*-malonyltransferase (LOC18055666, LOC18038126, LOC18044783, and LOC18046030) and putative anthocyanidin reductase (LOC18047966), suggesting that the metabolism of anthocyanins is very active in this species. It should be mentioned, that mandarins do not contain anthocyanins likely because the Ruby gene (synonymous AN2) is not functional in these varieties (Butelli et al., 2019; Wu et al., 2021). Supplementary Table 3 also lists 2 flavonol-specific transcription activators, MYB11 and MYB111 (LOC18049115 and LOC18031574) involved in the regulation of several genes of the flavonoid biosynthesis. The synthesis of the phytoalexin glyceollin, however, does not appear to be promoted since 3,9-dihydroxypterocarpan 6A-monooxygenase (LOC18031687), a previous biosynthetic step, was downregulated.

#### Terpenoids

In the terpenoid pathway, there were clear-cut differences between both species. The papeda showed upregulation of pivotal genes of the mevalonate pathway, in detriment of the MEP pathway, that takes place in plastids (Supplementary Figure 4.072). Thus, as related to the biosynthetic regulation of the isoprenoid precursors, isopentenyl pyrophosphate was promoted against dimethylallyl pyrophosphate. In addition, the pivotal gene, geranylgeranyl diphosphate synthase, (dimethylallyltranstransferase, LOC18039078, EC:2.5.1.1, and LOC18039079, EC:2.5.1.29, Supplementary Table 2), giving rise to main precursors of the different terpenoid types, were also expressed at higher levels. The synthesis of monoterpenoids was not basically modified except for the upregulation of 2 out of 3 (R)-limonene synthases 1 (LOC112098486 and LOC112098571, Supplementary Table 3), catalyzing the conversion of geranyl diphosphate to (+)-(4R)-limonene. In the diterpenoid biosynthesis, trimethyltridecatetraene/dimethylnonatriene synthase, CYP82G1 (LOC18049179 and LOC18049178, EC:1.14.14.58, Supplementary Figure 4.074 and Supplementary Table 2), catalyzing the production of the volatile homoterpenes DMNT and TMTT, was also upregulated. In the sesquiterpenoid pathway, the synthesis of farnesene appears to be downregulated since transcripts of α-farnesene synthase (LOC18053589, EC:4.2.3.46, Supplementary Figure 4.078 and Supplementary Table 2) are present at lower levels, while there were two (3S,6E)-nerolidol synthase 1 genes (LOC18033168 and LOC18051782, EC:4.2.3.48) expressed in opposite directions. Expression of premnaspirodiene oxygenase (LOC18052043), involved in the biosynthesis of the sesquiterpenoid, solavetivone, a potent antifungal phytoalexin, was upregulated, while that of α-copaene synthase-like (LOC112095677) that converts farnesyl diphosphate to the bicyclic olefins α-copaene and (*E*)-β-caryophyllene and participates in the synthesis of the macrocyclic sesquiterpene germacrene D, was downregulated (Supplementary Table 3). One important gene implicated in the synthesis of steroids and triterpenoids was squalene monooxygenase, SQLE (LOC18033947 and LOC18033838, EC:1.14.14.17, Supplementary Figures 4.012, 4.078 and Supplementary Table 2), that was downregulated in the papeda and therefore, probably limiting the flux toward these compounds. Consistently, the number of DEGs regulating triterpenoid metabolism were also scarce, since only the synthesis of β-amyrin appears to be upregulated (LOC18034001, EC:5.4.99.39, LOC18045727 and LOC18053646, Supplementary Tables 2, 3). Regarding carotenoids, only transcripts coding for enzymes mediating phytoene synthesis (LOC18051922, EC:2.5.1.32, Supplementary Figure 4.076 and Supplementary Table 2) were upregulated in the papeda, while the metabolism of α- and β-carotenes, including the synthesis of cryptoxanthin, lutein, astaxanthin and the xanthophyll cycle, was strongly repressed. Supplementary Table 3 also lists two upregulated β-D-glucosyl crocetin β-1,6-glucosyltransferases (LOC18045744 and LOC18044391), catalyzing the β 1-6 glucosylation of crocetin, a natural apocarotenoid. In addition, this table reports on other two glycosyltransferases (LOC18054015 and LOC18033356) conjugating diterpenes that are downregulated, and an upregulated gene of the diterpenoid metabolism, cytochrome P450 76M5 (LOC18031421), involved in the biosynthesis of oryzalexin, a class of phytoalexins. The terpenoid pathways implicating plant hormones such as GAs, ABA, cytokinins and brassinosteroids are described in the Hormonal category. Upregulation of the committed steps of the synthesis of tocopherol and tocotrienol (vitamin E), homogentisate phytyltransferase/geranylgeranyltransferase (LOC18055996, EC:2.5.1.115 and EC:2.5.1.116, Supplementary Figure 4.013 and Supplementary Table 2) was also found in the papeda.

#### Hormonal regulation

The comparison between the transcriptome of ICH and SCM also rendered a relatively high number of DEGs involved in hormone biosynthesis and action. According to the KEGG mapping of biosynthetic DEGs (Supplementary Table 2), the synthesis of active cytokinins, brassinosteroids and ethylene was downregulated in the papeda. Regarding cytokinins biosynthesis, transcripts for cytokinin dehydrogenase, CKX (LOC18042746 and LOC18033392, EC:1.5.99.12), an enzyme that inactivates isopentenyl adenine, were upregulated, in contrast to those of two glucosyltransferases conjugating zeatin, (LOC18031300, EC:2.4.1.215, and LOC18038024 and LOC18037288, EC:2.4.1.-), that were repressed (Supplementary Figure 4.077). The synthesis of brassinosteroids depending upon steroid precursors, also appears to be strongly downregulated (Supplementary Figure 6), since most steps, including last steps in the synthesis of brassinolide, such as brassinosteroid-6-oxidase 1, CYP85A1 (LOC18038016 and LOC18044268, EC:1.14.-.-) and PHYB activation tagged suppressor 1, CYP734A1 (LOC18054829, EC:1.14.-.-), were downregulated (Supplementary Figure 4.075). The generation of ethylene does not appear to be promoted either in the papeda, because the last step in its synthesis, 1-aminocyclopropane-1-carboxylate oxidase (LOC18050524, EC:1.14.17.4) was repressed, although the previous conversion catalyzed by 1-aminocyclopropane-1-carboxylate synthase (LOC18048242 and LOC18046436, EC:4.4.1.14), was upregulated (Supplementary Figure 4.024). Although there was upregulation of early steps in the gibberellin biosynthesis, including the conversions between ent-kaurene to inactive GA_12_ (LOC18046916, EC:1.14.14.107, Supplementary Figure 4.074) no differences in the expression of biosynthetic genes controlling the formation of active GAs between both species were found. A similar situation was observed in the synthesis of jasmonate and methyl-jasmonate, characterized by the upregulation of no less than 6 early biosynthetic steps (MFP2, LOC18032845, EC:4.2.1.17 and ACX, LOC18047109, EC:1.3.3.6, Supplementary Figure 4.053). The synthesis of xanthoxin, a precursor of ABA, and ABA degradation, was upregulated in the papeda, since genes coding for 9-*cis*-epoxycarotenoid dioxygenase (LOC18043465 and LOC18050641, EC:1.13.11.51) and abscisic acid 8′-hydroxylase (LOC18039758, EC:1.14.14.137, Supplementary Figure 4.076), the proteins controlling these conversions were expressed at higher levels. The data also suggest that auxin synthesis was also promoted because two amidases (LOC18033993 and LOC18034584, EC:3.5.1.4), an aldehyde dehydrogenase (LOC18036436, EC:1.2.1.3) and at least an indole-3-pyruvate monooxygenase (LOC18032700, EC:1.14.13.168) participating in the auxin synthesis, were expressed at higher levels (Supplementary Figure 4.033). In contrast, the amino acid derived polyamines, spermidine (LOC18039571, EC:1.5.3.17), spermine (LOC18043803, LOC18051913 and LOC18054425, EC:1.5.3.16) and putrescine (LOC18049691, EC:4.1.1.17) were apparently downregulated (Supplementary Figure 4.029 and Supplementary Table 4). Moreover, the expression levels of pivotal receptors, transporters and regulators implicated in the hormone signal transduction, indicate that the receptors of cytokinins, CRE1 (LOC18052080, EC:2.7.13.3), brassinosteroids, BRI1 (LOC18035850, EC:2.7.10.1, and EC:2.7.11.1) and ethylene, ETR (LOC18031847, EC:2.7.13.-) are repressed in the papeda, like most components of the auxin transduction pathway, including the auxin flux carrier AUX1 (LOC18034947, LOC18038157, and LOC18045480) and the regulators TR1 (LOC18052162) or GH3 (LOC18035901, LOC18053209, LOC18054772, LOC18033692, and LOC18041056). In contrast, JAR1 (LOC18049830, EC:6.3.2.52, ST 7267) and PP2C (LOC18043434, EC:3.1.3.16), major regulator of jasmonate and ABA responses, respectively and GID1 (LOC18049839 and LOC18043172), the receptor of GAs, were both upregulated (Supplementary Figure 4.109). Regarding MAPK Signaling Pathway, upregulation of MKK3 (LOC18051058, EC:2.7.12.2) and MPK6 (LOC18047683, EC:2.7.11.24, Supplementary Figure 4.107 and Supplementary Table 2), was the most significant observation as related to hormonal regulation.

In addition, Hormonal Regulation category included 208 DEGs, out of which more than 58% were downregulated in ICH (Supplementary Table 3). Although all phytohormones were represented in this set of genes, not all of them exhibited the same down/up regulation ratio. In particular, transcripts related to auxins (49/19, transport, homeostasis, ARFs, AUX/IAA, SAURs, response, biosynthesis, signaling), cytokinins (6/2, transport, receptors, transcription factors), gibberellins (13/4, biosynthesis, response, signaling, transcription factors) and jasmonic acid (13/8, transcripts related to biosynthesis, transport, response, receptor, induced response, signaling, transcription factors) had higher number of downregulated genes. Downregulation frequency of these categories ranked from 0.62 to 0.76. Genes linked to ethylene (6/18, transcription factors, induced responses, ERFs) displayed the opposite tendency, while transcripts associated with ABA (21/20, biosynthesis, response, receptor, induced response, signaling, transcription factors), polyamines (1/1, transporters), salicylic acid (8/10, transcription factors, induced responses, signaling, biosynthesis) and brassinosteroids (4/4, transcription factors, response, signaling, homeostasis) exhibited a down/up ratio that hardly departs from 0.5 (Supplementary Table 3).

Other categories related to growth, such as Gametophyte, Organ Development, Differentiation, Chloroplast, Cell division, Meiosis, Cytoskeleton, Cell Wall, Receptors and Protein Kinases, Chromatin, Histones, Transcription, and Nucleic Acids Processing were also downregulated in the papeda (Supplementary Material).

#### Primary metabolism

Regarding carbon metabolism, striking differences were found between both species, since practically all genes coding enzymes of central regulatory pathways such as glycolysis (Supplementary Figure 4.001), including the generation of pyruvate, acetyl CoA and acetaldehyde (Supplementary Figure 4.057), were clearly up-regulated in ICH. In the pentose phosphate pathway (Supplementary Figure 4.003), the synthesis of the pivotal intermediate, glyceraldehyde 3P, was similarly upregulated. Expression of genes involved in sucrose synthesis and degradation do not appear to be clearly modified, while the formation of ADP-glucose and amylose (Supplementary Figure 4.042), but not that of starch, also were upregulated in the papeda, as that of α amylase (LOC18043125 and LOC18045113, EC:3.2.1.1, Supplementary Figure 4.042 and Supplementary Table 2). In this species, it was also repressed a regulatory subunit of the probable trimeric SNF1-related protein kinase, (SnRK; LOC18052199, Supplementary Table 3) complex, that appears to play a role in the transduction cascade regulating gene expression and carbohydrate metabolism. In the papeda, other important differences were found in the metabolism of organic acids, especially the tricarboxylic cycle (TCA), that showed up-regulation of most genes coding for their regulatory enzymes (Supplementary Figure 4.002 and Supplementary Table 2), including those of the pyruvate dehydrogenase complex (LOC18045003 and LOC18037469, EC:2.3.1.12). A noticeable exception to this observation, however, was the conversion of oxoglutarate to succinyl-CoA that was down-regulated (LOC18044474, EC:1.2.4.2). Succinyl-CoA synthetase alpha subunit (LOC18045656, EC:6.2.1.4 and EC:6.2.1.5), on the other hand, was upregulated. Expression of genes regulating cytoplasmatic organic acid metabolism was similarly altered, since several subunits of ATP-citrate lyase, ACLY (LOC18032750 and LOC18043354, Supplementary Table 2 and LOC18039980, Supplementary Table 3), that converts citrate into oxaloacetate and cytosolic acetyl-CoA, were upregulated. Likewise, genes coding for a series of enzymes acting sequentially, such as aconitate hydratase, ACO3 (LOC18055416, EC:4.2.1.3, Supplementary Figure 4.058), one isocitrate dehydrogenase [NADP], IDH1, (LOC18031748, EC:1.1.4.2) and aspartate transaminase, GOT1 (LOC18054901, EC:2.6.1.1, Supplementary Figure 4.017), rendering the amino acid glutamate from 2-oxogluarate, were also upregulated. In addition, three additional enzymes, glutamate decarboxylase, GAD5 (LOC18046053 and LOC18052541, EC:4.1.1.15), butyrate pyruvate transaminase, POP2 (LOC18039191, EC:2.6.1.96) and succinate-semialdehyde dehydrogenase, SSADH (LOC18031917, EC:1.2.1.24), that together make up the GABA shunt, showed the same tendency (Supplementary Figure 4.021). The conversion of glutamate to glutamine (LOC18044424, EC:6.3.1.2) and pyrroline-carboxylate (LOC18045924, EC:1.2.1.88) also was favored in ICH (Supplementary Figure 4.021). The oxidative phosphorylation likewise seems to be more active in ICH, since all DEGs implicated in this process were upregulated (Supplementary Figure 4.014 and Supplementary Table 2). These genes code for several components of complex I, NADH hydrogenase, including NADH-quinone oxidoreductase subunit A (LOC18034396, EC:7.1.1.2); complex III, cytochrome c reductase, including ubiquinol-cytochrome c reductase cytochrome b/c1 subunit (LOC18051936, EC:7.1.1.8), complex IV, cytochrome oxidase, including cytochrome c oxidase cbb3-type subunit I, COX6A and COX6B (LOC18037730 and LOC18055702, EC:7.1.1.9) and complex V, ATP synthase, including H + -transporting ATPase (LOC18055993, LOC18039766, LOC18053876, and LOC18035736, EC:7.1.2.1). Major changes in the lipids and fatty acids pathways are discussed in Supplementary Material.

#### Amino acid metabolism

Amino acid metabolism and the synthesis of several derived compounds differ in both species. Supplementary Table 4 speculates on the regulation of the synthesis of these compounds in each species, based on gene expression levels of the last regulatory steps (Supplementary Figures 4.017, 4.021–4.036, 4.039, 4.042). According to this information, the synthesis of amino acids tends to be upregulated in ICH, except for the production of valine, leucine, and isoleucine that was clearly repressed, and their degradation upregulated. Data related to amino acid-derived hormones and alkaloids are specified elsewhere in this section. In the cyanoamino acid metabolism, it is worth to mention that in ICH, linamarin synthase (LOC18036876, Supplementary Table 3), an UDP glycosyltransferase producing cyanogenic glucosides was upregulated. Consistently, β-glucosidase 13 (orthologous of β-glucosidase 12 of cassava, linamerase, LOC18044658, EC:3.2.1.21, Supplementary Table 2), that converts cyanogenic glucosides into acetone cyanohydrins such as mandelonitrile, and mandelonitrile lyase (LOC18037363, EC:4.1.2.10), that releases HCN, hydrogen cyanide, from the acetone were both downregulated (Supplementary Figure 4.039). The synthesis of glucosinolates may be promoted in the papeda, since a flavin-containing monooxygenase FMO GS-OX-like 4 (LOC18049889, Supplementary Table 3) and two mRNAs coding for cytochrome P450 83B1 (LOC18041363 and LOC18046366, ST 2832) that catalyze the oxime metabolizing step in indole glucosinolate biosynthesis were upregulated.

#### Transport

In the papeda, the Transport category was also enriched in downregulated genes (Figure 1), although specific differences were found among the wide range of transport systems included in this group (Supplementary Table 3). For instance, ABC transporters for glutathione S-conjugates, vacuolar ATPases, and copper, magnesium, sulfate, and zinc transporters were mostly upregulated, while transporters of amino acids, ascorbate, cation channels, proton antiporters, components of the mitochondrial electron transport chain, mechanosensitive channels, metal-nicotianamine transporters, nitrate, sodium, cadmium, nuclear import, oligopeptides and xenobiotics were mostly downregulated. Other transporters, such as aquaporins, purines, and transporters of boron, calcium, and potassium showed similar number of up- and downregulated genes. The number of sugar transporters was relatively high (19) and some genes were highly expressed in SCM mandarin in contrast to ICH, such as the vacuolar hexose transporter SWEET17 (LOC18032835) and other hexoses carriers (LOC18031330 and LOC18048094) or monosaccharide transporters (LOC18031593). ALTM4 (LOC18043583), an aluminum-activated malate transporter was also downregulated in papeda.

#### Abiotic stress

As related to stress responses, upregulation enrichment was observed in the papeda fruitlet flesh predominantly in three cases (Supplementary Table 3): in heat shock proteins (4/11), in cold responses (3/5), and in oxidative stress (6/35). Out of the five upregulated genes involved in cold responses, CORA, a cold and drought-regulated protein, showed the highest expression observed in this analysis (LOC18054952). Among the three downregulated transcripts, there were two transcription factors, the activator ICE1 (LOC18051975) and the repressor MYBS3 (LOC18055469), that regulate the cold-induced transcription of DREB1/CBF genes. Additional genes involved in cold responses mediated by ABA were also differentially expressed among both species. For instance, negative regulators of ABA such as MSI4 (LOC18036552) and ERD15 (LOC18045483) were expressed at lower levels. JUB1 (LOC18044819), another gene participating in the response to freezing was upregulated. As mentioned before, a further category enriched in the papeda with upregulated DEGs was that of Redox (Figure 1 and Supplementary Table 3). It includes mostly genes coding for oxidoreductase enzymes, that play major roles in the antioxidant defense system, such as oxidases, reductases, peroxidases, cytochromes P450, mono- and dioxygenases and glutathione S-transferases. In the glutathione-ascorbate cycle, an efficient metabolic pathway to detoxify H_2_O_2_, monodehydroascorbate reductase (LOC18039033, E.C:1.6.5.4) was upregulated, while ascorbate peroxidase (LOC18039197, LOC18040244, LOC18037637, LOC18042802, and LOC18035392, E.C. 1.11.1.11) appears to be predominantly repressed (Supplementary Figures 4.007, 4.041 and Supplementary Table 2). Two glutathione peroxidases (LOC18047364, EC:1.11.1.9, Supplementary Figure 4.041, Supplementary Table 2 and LOC18047405, Supplementary Table 3), that were also upregulated, might further contribute to H_2_O_2_ removal. In addition, the data indicate that several sequential enzymes, such as nicotinamidase (LOC18034819, Supplementary Table 3), nicotinate phosphoribosyltransferase 1 (LOC18036859, EC:6.3.4.21), pyrimidine and pyridine-specific 5′-nucleotidase (LOC18050280, EC:3.1.3.-), nicotinamide/nicotinic acid mononucleotide adenylyltransferase (LOC18055145, EC:2.7.7.1), and NAD + kinase (LOC18042070, EC:2.7.1.23), implicated through the salvage pathway in the synthesis of the central coenzyme NAD + /NADH, were upregulated in the papeda (Supplementary Figure 4.067 and Supplementary Table 2).

### Gene expression in palatable pummelo/mandarin genetic admixtures versus acidic Sun Chu Sha Kat mandarin

To select mRNAs that were differentially expressed in the pulp of developing fruitlets of acidic and palatable mandarins, the transcriptomes of SCM and three palatable pummelo/mandarin genetic admixtures, coded S1, S2, and S3, were compared through RNA-seq analysis. Pairwise comparisons of each segregant against SCM (Log_2_FC = 0.58 and α = 0.05) were generated and the 357 DEGs showing similar expression patterns in the three palatable mandarins and opposite in SCM were selected following the criteria utilized in previous comparisons (Supplementary Table 5).

#### Alkaloids

Out of the eight DEGs clustered in the Alkaloids category, seven of them belonged to three groups that were previously identified in the comparison between ICH and SCM. Five of these genes (3/2) belonged to the group of tropinone reductase homologs, that as commented above do not appear to possess tropinone reductase activity. LOC18039754 and LOC112096160, the two genes with highest expression in this group showed opposite tendencies. The other two genes, geraniol 8-hydroxylase (LOC18040911), involved in the biosynthesis of terpenoid indole alkaloids and in the biosynthesis of flavonoids, and methanol *O*-anthraniloyltransferase (LOC18050771), that generates methyl anthranilate, were both upregulated in the segregants. The remaining gene was downregulated and is annotated as hyoscyamine 6-dioxygenase-like (LOC112100197), the limiting step in the synthesis of scopolamine in the tropane alkaloid biosynthesis.

#### Terpenoids

The Terpenoids category (4/2) was characterized by the upregulation of zeta-carotene desaturase (LOC112098137), chloroplastic/chromoplastic-like, that plays a crucial role catalyzing the conversion of zeta-carotene to lycopene in the biosynthesis of carotenoids. The three palatable mandarins also showed upregulation of (–)-germacrene D synthase-like (LOC112100727), suggesting an activation of the synthesis of the germacrin-type sesquiterpenoid, germacrene *D*, a class of volatile organic hydrocarbon with antimicrobial and insecticidal properties. The conversion of these sesquiterpenes to the derived lactones was probably downregulated, since α-copaene synthase-like (LOC112095677), that catalyzes several of these conversions, was repressed. In the acyclic sesquiterpenoid pathway, two additional downregulated genes were (*E*)-β-farnesene synthase-like (LOC112101583), a cyclase catalyzing the production of β-farnesene, and dimethylnonatriene synthase (LOC18049179), a cytochrome P450 82G1 involved in the biosynthesis of homoterpenes such as TMTT. (R)-limonene synthase 1 (LOC112098571), chloroplastic-like, that synthetizes the monoterpene limonene also was downregulated.

#### Sugar metabolism

In the three palatable mandarins, sugar metabolism (6/1) was characterized by the repression of sucrose synthase 2 (LOC18032959), probable galactinol-sucrose galactosyltransferase 2 (LOC18031328) and stachyose synthase (LOC18050124). The first gene is implicated in sucrose cleavage and the other two in the synthesis of raffinose, stachyose, and verbascose. Other repressed genes were probable trehalose-phosphate phosphatase F (LOC18049897), that produces free trehalose and phosphoenolpyruvate carboxylase kinase 1, that through decarboxylation renders oxalacetate to fuel the citric acid cycle. The only gene upregulated in this category was enolase (LOC18031514), that is responsible of the conversion of 2-phosphoglycerate (2-PG) to phosphoenolpyruvate (PEP).

#### Transport

This category grouped a set of genes (10/9) showing different patterns of expression. Relevant upregulated genes, for instance, were an aquaporin (LOC18045515), a calcium-transporting ATPase (LOC18042874), a potassium transporter (LOC18043735), and two different sugar transporters ERD6-like (LOC18040371 and LOC18046355), which are vacuolar H + /glucose symporters involved in the export of glucose to cytosol (Klemens et al., 2014). Downregulated genes were a boron transporter (LOC18036378), a nitrate reductase (LOC18041814), a zinc transporter (LOC18050857), and overall, an ATPase 10, plasma membrane-type (LOC18035736), that has been previously associated with citric acid accumulation in lemon juice (Aprile et al., 2011). There were also two members of the sodium/hydrogen exchanger gene family (LOC18040436 and LOC18039966), involved in acidity regulation in oranges (Wang et al., 2021), expressed in opposite directions.

Other categories are discussed in Supplementary Material, although two transcripts should be mentioned for their potential relevance. One of these is UDP-glycosyltransferase 74B1 (LOC18044914), that is involved in the biosynthesis of benzyl-glucosinolate and appears to be downregulated in the three segregants. The other gene is 2-methylene-furan-3-one reductase (LOC18050984), that codes for the enone oxidoreductase rendering furaneol, the key flavor compound in strawberries (Raab et al., 2006), and is expressed at higher levels in the palatable mandarins.

### Gene expression as related to acidity in palatable pummelo/mandarin genetic admixtures versus Sun Chu Sha Kat mandarin

**FIGURE 2:**
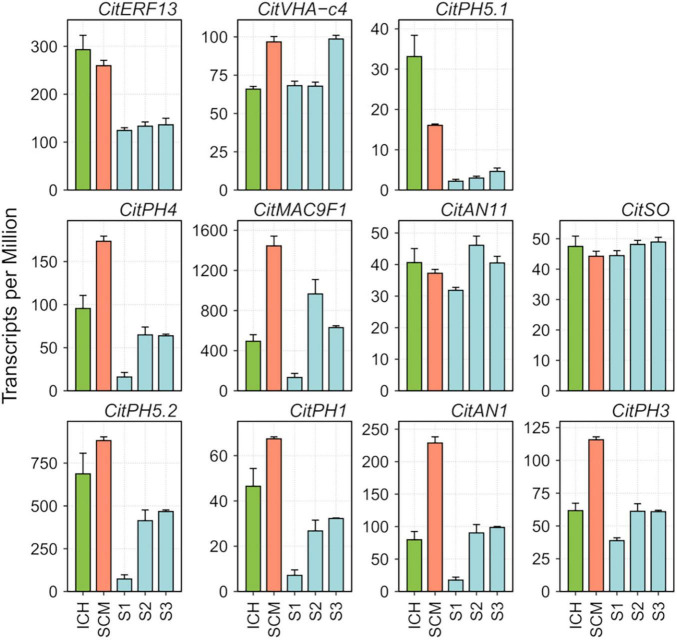
RNA abundance of relevant genes reported to be involved in acid regulation of citrus fruits (Aprile et al., 2011; Butelli et al., 2019; Strazzer et al., 2019; Huang et al., 2021), obtained in RNA-seq analyses of the pulp of developing fruitlets of ICH, SCM, and S1, S2, and S3 segregants. Transcripts per Million were computed using DESeq2 read count normalization. Vertical bars represent standard error from three biological replicates.

The finding that ATPase 10, plasma membrane-type (Aprile et al., 2011), a pivotal component of the vacuolar proton-pumping P-ATPase complex that regulates acidity in citrus (Strazzer et al., 2019), was downregulated in the three segregants, prompted us to focus our attention on the expression of the rest of components of this complex and of other related genes previously associated with this process (Huang et al., 2021). This set of genes, listed in Supplementary Table 6, included CitPH1 (LOC18037376, magnesium-transporting ATPase, P-type 1), two existing versions of CitPH5; namely, CitPH5.2 (LOC18035739, ATPase 10, plasma membrane-type) and CitPH5.1 (LOC18035736, ATPase 10, plasma membrane-type), CitAN1 (LOC18047507, basic helix-loop-helix protein A, synonymous Noemi), CitPH3 (LOC18038669, WRKY transcription factor 44), CitERF13 (LOC18047942, ethylene-responsive transcription factor 13), CitAN11 (LOC18032473, protein TRANSPARENT TESTA GLABRA 1), CitSO (LOC18039929, protein PIN-LIKES 6), CitVHA-c4 (LOC18041768, V-type proton ATPase 16 kDa proteolipid subunit), CitMAC9F1 (LOC18037289, uncharacterized LOC180372899), and CitPH4 (LOC18053295, transcription factor MYB34) (Shi et al., 2015; Li et al., 2016; Butelli et al., 2019; Shi et al., 2019; Strazzer et al., 2019; Huang et al., 2021; Wang et al., 2021). Although most of these genes showed relatively low TPM (Transcripts per Million) values (Figure 2), generally combined with high variability between replicates, raw data show that 8 out of these 11 transcripts, namely, CitPH1, CitPH5.2, CitMAC9F1, CitAN1, CitPH3, CitPH4, CitPH5.1, and CitERF13, were expressed at lower levels in the palatable mandarins. Further qPCR analyses confirmed these tendencies in the samples tested (Supplementary Table 7).

**FIGURE 3:**
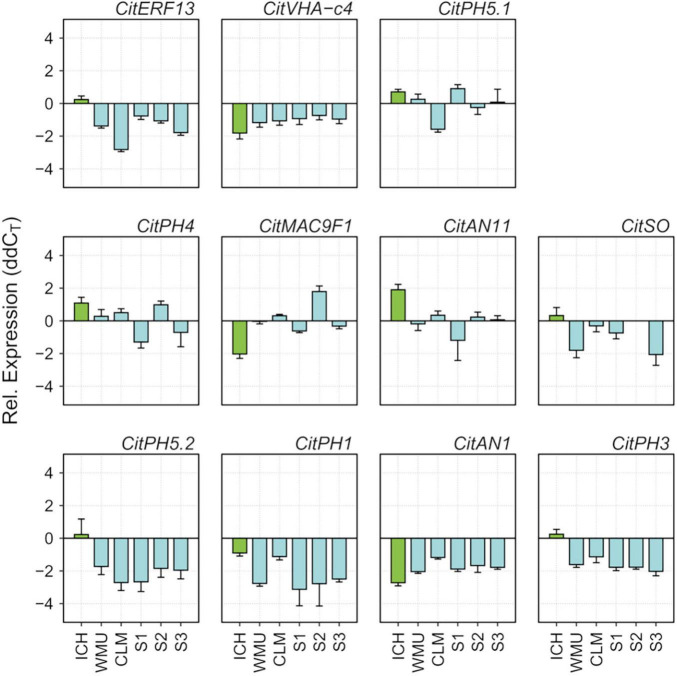
Relative expression of genes reported to be involved in acid regulation of citrus fruits (Aprile et al., 2011; Butelli et al., 2019; Strazzer et al., 2019; Huang et al., 2021), determined by RT-qPCR in juice vesicles of ripening fruits (November) of ICH, SCM, WMU, CLM and S1, S2, and S3 segregants. Vertical bars represent standard error from two technical replicates.

Correlation between expression of these genes and acidity, was studied in mature fruit, since the period of acid accumulation in mandarins usually starts about early July and reaches maximum acid levels around the end of September (Cercós et al., 2006). During ripening, fruits of SCM and the three palatable mandarins contained approximately the same sugar quantities (°Br), although SCM fruits were much more acidic, which resulted in lower, unacceptable maturity indices (Supplementary Figure 7). Total acidity in the segregants reached palatability levels similar to those of commercial Clementine and W. Murcott, while SCM still contained higher and unpleasant amounts of acids at the end of the ripening period. qPCR data from juice vesicles of samples collected in November showed that transcript levels of CitVHA-c4, CitPH1, CitPH5.2, CitAN1, CitPH3, CitSO, and CitERF13 were downregulated in the three segregants in comparison with SCM, as observed in Clementine and W. Murcott fruits (Figure 3). Taken together, these observations suggest that CitPH5.1, CitPH4, CitMAC9F1, CitAN11 appear to play a minor role controlling acidity during ripening.

### Differential allele expression in pummelo/mandarin genetic admixtures

**FIGURE 4:**
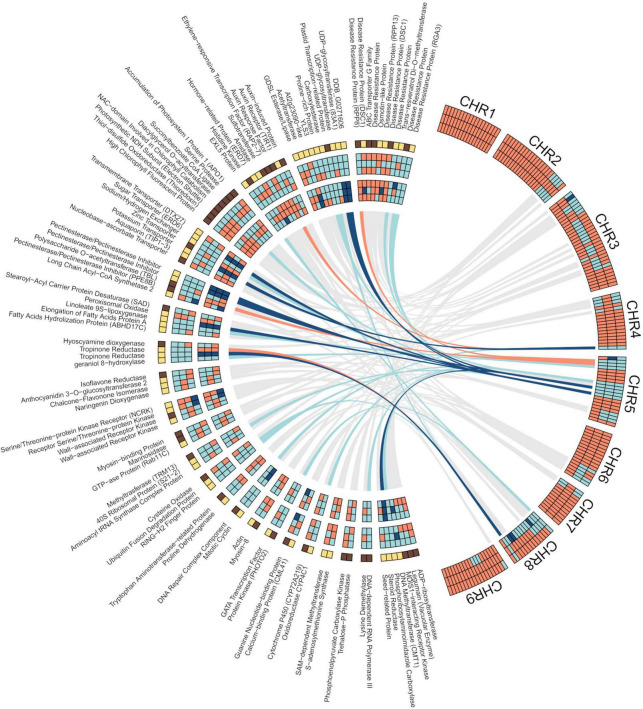
Shared DEGs in the pulp of developing fruitlets of segregants, S1, S2, and S3, as related to SCM. Right Circos. Chromosome admixture structure of the three segregants. From top to bottom, S3 (first row), S2 (second row), and S1 (third row); orange: mandarin/mandarin; light blue: pummelo/mandarin; dark blue: pummelo/pummelo. Left Circos. First row (from top to bottom): gene expression; yellow: up; dark brown: down. Second, third, and fourth rows: gene genotype; orange: mandarin/mandarin; light blue: pummelo/mandarin; dark blue: pummelo/pummelo. Fifth, six, and seventh rows: allele expression; orange: mandarin/mandarin; light blue: pummelo/mandarin; dark blue: pummelo/pummelo. Ribbons connect each gene which its physical position in the reference genome. DEGs with the same genotype in the three segregants are connected by colored ribbons; orange: mandarin/mandarin; light blue: pummelo/mandarin; dark blue: pummelo/pummelo. All other DEGs are connected to their respective positions by gray ribbons. DEGs at the outside of the Circos are clustered by categories (Supplementary Table 5) and ordered by the number of members of the category, from top to bottom: Plant defense (11); Uncharacterized process (10); Hormonal regulation (9); Chloroplast (8); Transport (7); Cell wall (5); Lipid and fatty acids (5); Alkaloids (4); Flavonoids (4); Receptors, kinases, transduction (4); Trafficking, vesicles (3); Translation (3); Ubiquitination (3); Aminoacid metabolism (2); Cell division (2); Cytoeskeleton (2); Light signaling (2); Membranes, RE, signaling (2); Redox (2); *S*-adenosylmethionine metabolism (2); Sugar metabolism (2); and Transcription (2). A final cluster included those categories with a single member: Seed, embryo development (1); Organ development, differentiation (1); Nucleobases (1); Nucleic acids processing (1); Gametophyte and fertilization (1); Cell death (1); and Abiotic stress (1). Results were plotted in R language and environment (R Core Team, 2021), using packages included in Tidyverse collection (Wickham et al., 2019) and circlize (Gu et al., 2014).

We additionally studied the influence of pummelo alleles on the differential gene expression of the three segregants as related to SCM. The data show that the percentage of genes expressed in each haplotype sequence, i.e., MA/MA, PU/MA, and PU/PU, in the three segregants was, on average, 73, 25, and 1%, respectively. However, the frequency of DEGs calculated for each haplotype was higher in PU/PU (0.33), intermediate in PU/MA (0.16) and lower in MA/MA (0.09) sequences (Supplementary Figure 8). As related to the differential expression of the pummelo and the mandarin alleles, in non-DEGs, the percentage of expressed alleles was slightly higher for MA (52–58%) than for PU (42–48%), while in the set of DEGs the PU allele was predominantly expressed (68-75%), in detriment of the MA allele (25–32%). There also was a clear-cut difference between both alleles when the expression trend is considered since most MA (8 out of 12) alleles were downregulated, whereas virtually all PU alleles (27 out of 28) were upregulated. In the three segregants, 4 genes located in pummelo introgressed areas (LOC18035377, tropinone reductase homolog At2g29170; LOC18042174, pectinesterase/pectinesterase inhibitor, PPE8B; LOC18045400, auxin response factor 4; LOC112098377, disease resistance RPP8-like protein 3), only exhibited expression (down or up) of the MA alleles (Figure 4). In contrast, the MA alleles of other 9 genes (LOC18040371, sugar transporter ERD6-like 18; LOC18042131, UDP-glycosyltransferase 83A1; LOC112096160, tropinone reductase homolog At2g29170-like; LOC18043735, potassium transporter 5; LOC112095422, probable pectinesterase/pectinesterase inhibitor 21; LOC18041614, protein DDB_G0271606; LOC18041176, 3-oxo-Delta(4,5)-steroid 5-beta-reductase; LOC1804023, probable linoleate 9S-lipoxygenase 5; LOC18040524, probable pectinesterase/pectinesterase inhibitor 25) were not expressed at all. These set of genes expressing only PU alleles were all upregulated.

## Discussion

**FIGURE 5:**
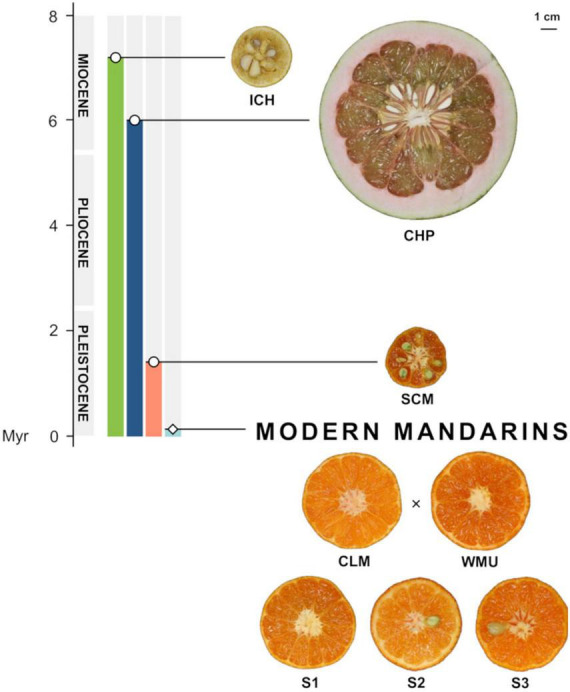
Timeline showing the emergence of the citrus mentioned in this study, according to Wu et al. (2018, 2021). ICH, Ichang papeda (*C. ichangensis*); CHP, Chandler pummelo (*C. maxima*); SCM, Sun Chu Sha Kat mandarin (*C. reticulata*); CLM, Clementine mandarin (*C. clementina*); WMU, W. Murcott mandarin (*C. reticulata*), and segregants S1, S2, and S3. The hybrid origin of these three segregants is marked with a cross.

The main goal of this work was to identify major domestication traits in citrus, based on the comparisons of gene expression patterns in the pulp of developing fruitlets of inedible and edible citrus types. The citrus examined were wild inedible Ichang papeda (ICH; *C. ichangensis*Swingle), acidic Sun Chu Sha Kat mandarin (SCM; *C. reticulata*, Blanco; *C. erythrose* Tanaka) and three selected palatable genetic admixtures, S1, S2, and S3, derived from a cross between Clementine (CLM; *C. x clementina* Hort. ex Tanaka) and W. Murcott (WMU, *C. x reticulata* Blanco). According to Wang et al. (2017), there were two main mandarin domestication events that generated two mandarin subpopulations differentiated by the degree of acidy. The parentals CLM and WMU were selected for this study because they are representative commercial mandarins of the two adjacent clades of the low acidity subpopulation of mandarins. We carried out the comparison with the three segregants rather than with the parental varieties, to reduce the number of false positives that could be generated comparing two genetically related varieties. The three segregants were selected for the study because their fruits exhibited morphological parameters (Figure 5) and organoleptic traits (Supplementary Figure 7) in the range shown by the parent varieties. ICH, that grows in a truly wild state, is an endemic citrus thought to be originated in glacial refugia in Wuling Mountains and Ta-pa Mountains in southwestern and middle-west China (Yang et al., 2017). It is currently found in natural populations in these areas, is the most cold-resistant citrus and is also tolerant to both damp and drought conditions (Swingle and Reece, 1967; Yang et al., 2017). ICH is considered one of the most primitive wild forms of citrus, produces inedible fruits with very little flesh and juice, if any, and contains acrid and sour oils that release aroma reminiscent of lemons. Recent developments suggest that ICH split from the main citrus clade around 7 million years ago (Wu et al., 2018). According to Tanaka (1954), SCM is an antique mandarin that was very common in temperate China, occurred in Assam and was also cultivated in Japan. The small SCM fruits, as those of several other traditional mandarins (Wu et al., 2018), are acidic or acidic-sweet, moderately sharp to the taste and very spicy. The ancestor of SCM probably appeared during the last 1.4 million years, after the divergence of the two main subspecies of mainland Asian mandarins (Wu et al., 2021). Under a genomic point of view, ICH and SCM contain pure genomes, i.e., do not show foreign genome introgressions (Wu et al., 2018), while S1, S2, and S3, are genetic admixtures carrying pummelo introgressions, in a mandarin genome background (Wu et al., 2014).

Samples used for RNA-seq analyses were collected during the transition between the phases of cell division and cell elongation of citrus fruits (Cercós et al., 2006; Tadeo et al., 2020), a period that appears to be critical for the establishment of major ripening characteristics (Terol et al., 2019). A first comparison between ICH and SCM rendered 7267 DEGs (Supplementary Table 2), that were mapped (Supplementary Figures 4.001–4.117) against the pathway collection of the KEGG database (Kanehisa et al., 2016). The remaining genes were filtered using more stringent criteria and a final set of 2832 genes were ranked according to the Uniprot database annotation (UniProt Consortium, 2021) in 45 categories (Supplementary Table 3). In a second analysis, the expression of the three palatable segregants, S1, S2, and S3, was studied as related to SCM. This analysis identified 357 DEGs that showed similar expression in the three palatable mandarins but opposite expression in SCM (Supplementary Table 5). The discussion that follows below highlights the most relevant results derived from those transcriptomic comparisons, while the rest of results are discussed in Supplementary Material.

### Differential gene expression in Ichang papeda versus Sun Chu Sha Kat mandarin: Upregulation of secondary metabolism

In ICH, expression of pivotal genes controlling secondary metabolites, as illustrated in the categories Alkaloids, Terpenoids, Phenylpropanoids, Flavonoids, Glucosinolates and Cyanogenic glucosides (Figure 1), was typically upregulated. Regarding Alkaloids, the data indicate that there are several pathways that were promoted in the papeda. Thus, the synthesis of caffeine, a methylxanthin which acts as a natural defense compound (Nathanson, 1984), appears to be promoted, as is that of morphine (Supplementary Table 3) in the isoquinoline alkaloid pathway. In this route, however, the data also suggest that the syntheses of both dopamine and tyramine and also of serotonin, an indoleamine, are downregulated. Interestingly, it has been recently shown that suppression of serotonin biosynthesis increases resistance to insect pests (Lu et al., 2018). Several biosynthetic genes controlling the conversions that render the iridoid glycoside secologanin, the building unit in the biosynthesis of indole and isoquinoline alkaloids, were upregulated in the monoterpenoid pathway (Supplementary Table 3). Iridoid glycosides show a broad defensive spectrum due to their deterrent character on herbivorous and the post-ingestive toxic effects on fungal pathogens (Biere et al., 2004). The upregulation of the synthesis of secologanin and that of other precursors of the indol alkaloids, such as ajmaline, vinblastine and vincristine, may also indicate that the indole alkaloid pathway, was similarly upregulated in the papeda.

This species also showed upregulation of practically all biosynthetic genes of the mevalonate pathway, leading to the isoprenoid intermediates, isopentenyl-PP and dimethylallyl pyrophosphate-PP. The data suggest that two genes encoding geranylgeranyl-PP, the enzyme responsible for the synthesis of the precursors of the main terpenoid groups, were also upregulated (Supplementary Figure 4.072). The synthesis of mono-, sesqui-, and homoterpenoids, the crucial groups of terpenoid volatiles operating attracting parasitoids or repelling herbivores, were characterized in the papeda by a relative high activity. Thus, most of the genes involved in the biosynthesis of the monoterpene limonene, a natural insecticide, antifeedant, antifungal and attractant for pollinators (Erasto and Viljoen, 2008), were upregulated, as it was the synthesis of the phytoalexin, solavetivone, a potent antifungal sesquiterpenoid. In this group, transcripts of α-farnesene synthase (Supplementary Figure 4.078), generating farnesene, that acts as pheromone, a natural insect repellent, and α-copaene synthase-like, that renders several bicyclic olefins and sesquiterpene hydrocarbons, were in contrast expressed at lower levels. There were also two (3S,6E)-nerolidol synthase 1 genes expressed in opposite directions (Supplementary Figure 4.078 and Supplementary Table 2). This enzyme participates in the synthesis of the homoterpene 4,8-dimethyl-1,3,7-nonatriene, DMNT (Degenhardt and Gershenzon, 2000), an observation linked to the upregulation of trimethyltridecatetraene/dimethylnonatriene synthase, encoding the enzyme (EC: 1.14.14.58), generating 4,8,12-trimethyl-1,3(E),7(E),11-tridecatetraene, TMTT, in the Diterpenoid Pathway (Supplementary Figure 4.074). DMNT and TMTT are two irregular acyclic monoterpenes exhibiting pivotal roles attracting parasitoids and predators of herbivores (Tholl et al., 2011). The synthesis of oryzalexin, a diterpenoid phytoalexin, also appears to be upregulated in the papeda (Supplementary Table 3, Schmelz et al., 2014). In the triterpenoid group, the synthesis of β-amyrin (Supplementary Figure 4.078 and Supplementary Table 3), a common plant saponin with important antimicrobial, antifungal, and anti-feedant properties (Faizal and Geelen, 2013), was similarly upregulated. Regarding carotenoids, only transcripts coding for enzymes mediating phytoene synthesis were upregulated in the papeda. Mandarins exhibit a wide range of carotenoids (Figure 5, Gross, 1987), and accordingly showed upregulation of this pathway (Supplementary Figure 4.076). In the phenylpropanoid pathway, main steps in the synthesis of the flavonoid precursors are downregulated, although the synthesis of flavonols, anthocyanidins and anthocyanins were clearly upregulated in the papeda (Supplementary Figures 4, 4.082, 4.083). Citrus fruits contain a wide range of flavonoids (Nogata et al., 2006), although mandarins and most cultivated citrus species do not, because carry defectives alleles of Ruby gene encoding a MYB transcription factor controlling anthocyanin biosynthesis (Butelli et al., 2017; Wu et al., 2021). From data in Supplementary Figure 4.082, it is suggested that Ruby (synonymous AN2) may participate in the regulation of the expression regulation of naringenin 3-dioxygenase, flavonol synthase and anthocyanidin synthase, key players in the synthesis of anthocyanidins. Flavonoids show antipathogenic activity and participate in the defense against biotic stresses caused by herbivory and pathogenicity. For instance, many flavonoids including the flavonols, kaempferol, quercetin and myricetin may act as deterrents against insects. Flavonoids also reduce the effects of abiotic stresses, such as UV radiation and heat, and show relevant antioxidant properties (Mierziak et al., 2014). In the pathway of the non-flavonoid polyphenol, trans-resveratrol di-*O*-methyltransferase, the last step in the biosynthesis of the antifungal phytoalexin pterostilbene, is expressed at higher levels in the papeda (Supplementary Table 3). The data also suggest that the synthesis of glucosinolates and cyanogenic glucosides, two kinds of phytoanticipins, may be promoted in the papeda. These are constitutive chemicals, whose non-toxic forms and the catalyzing enzymes that release the toxic compounds are stored in different cells (glucosinolates) or subcellular compartments (cyanogenic glucosides) (Yactayo-Chang et al., 2020).

The depletion of defensive chemicals is a process generally linked to domestication (Moreira et al., 2018), and in SCM appears to have played a critical role in the production of tastier and more flavorful citrus, since these compounds are essentially of bitter taste and toxic to arthropods and vertebrates (Matsuura and Fett-Neto, 2015). It is interesting also to mention that while chemical defenses, secondary metabolites that represent a major barrier to herbivorous insects, are restricted in SCM, in the Plant defense category (Supplementary Material), populated by all kind of protein-based defenses against microbial pathogens, there are more genes up than downregulated.

### Differential gene expression in Ichang papeda versus Sun Chu Sha Kat mandarin: Downregulation of growth

Another important difference in gene expression between both species is the prevalence in the papeda of downregulation of many genes (>60%), involved in practically all processes of growth and development (Supplementary Table 3). Categories enriched with downregulated genes included genes with roles in Development, Chloroplast, Hormonal Regulation, Signaling, Gene Expression, and Cellular Growth (Figure 1). While it may be reasonable to find expression of genes involved in chloroplast metabolism or even photosynthesis in the developing fruit pulp, where the transition chloroplast/chromoplast maybe still ongoing, the finding that a high number of genes unquestionably involved in processes such as flowering, fertilization, or organ development (leaves, gametophytes, roots, etc.) were also expressed in this fruit tissue, might be unexpected. There are no convincing explanations for this observation, except perhaps that certain transcripts have not been yet degraded, or that in addition to the reported functions, some genes could also be implicated in fruit growth in hitherto unknown roles. Repressed genes controlling papeda development were also associated with the biosynthesis of cytokinins, brassionosteroids, ethylene (Supplementary Figures 4.077, 4.075, 4.024), or polyamines (Supplementary Table 4 and Supplementary Figure 4.029) and with the transduction of plant hormones in general (Supplementary Figure 4.109). In addition, Supplementary Table 3 also reports that a majority of DEGs included in the Hormonal Regulation category, were downregulated in ICH, particularly those linked to auxins and gibberellins. The repression is also evident in groups of genes related to cellular growth (Figure 1), including the categories of Cell Division (Supplementary Figures 4.099, 4.102–4.105), Meiosis, Cytoskeleton and Cell Wall (Supplementary Table 3). Consistently, the data also revealed lower levels of gene expression in basic genetic processes regulating growth, such as nucleocytoplasmic transport (Supplementary Figure 4.094), mRNA surveillance pathway (Supplementary Figure 4.095), the processing of the nucleic acids or the chromatin condensation and transcription (Supplementary Table 3). Similarly, low expression was associated with signaling transduction pathways involving genes in Receptors, Kinases, Transduction category or GTPases and second messengers as reported in Supplementary Table 3. Biosynthesis of steroids, one of the most important components of the cellular membranes was also strongly repressed in the papeda (Supplementary Figure 4.012). Other categories enriched with downregulated genes were Transport and Plant Defense (Supplementary Table 3).

### Differential gene expression in Ichang papeda versus Sun Chu Sha Kat mandarin: Activation of secondary metabolism versus growth stimulation

**FIGURE 6:**
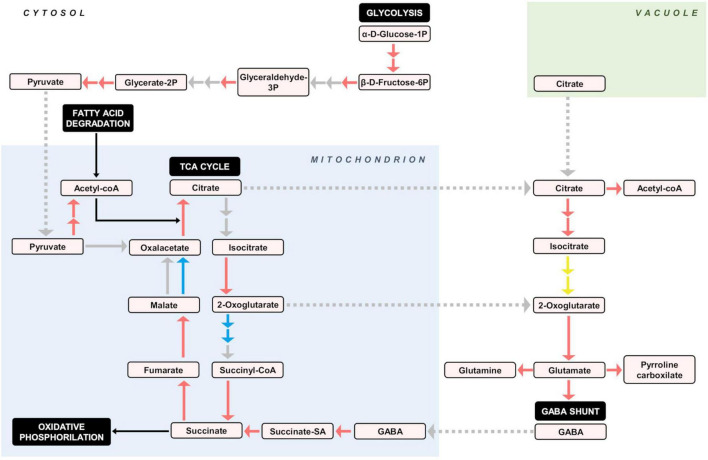
Proposed activity of central carbon metabolic pathways, deduced from DEGs in the pulp of developing fruitlets of ICH as related to SCM. Bigger solid arrows indicate gene regulation: red = upregulation, blue = downregulation, yellow = undetermined, and gray = no differential expression. Small solid black arrows represent substrate inputs, while gray dotted arrows indicate directional transport. Reaction products and pivotal pathways are embedded in colored and black boxes, respectively.

The RNA-seq analysis, overall, reveals that upregulation in the papeda was mostly associated with the increase of chemical defenses (Table 1), a situation that may imply a penalty in terms of energy and development, as suggested by the downregulation of relevant DEGs involved in a wide variety of growth processes. These conspicuous differences appear to be related to the control of the carbon flux through central pathways of the primary metabolism. Thus, the KEGG data show that gene expression of practically all genes encoding enzymatic activities involved in glycolysis (Supplementary Figure 4.001), cytoplasmatic citric acid degradation (Supplementary Figures 4.017, 4.021, 4.058), GABA shunt (Supplementary Table 2), fatty acid degradation (Supplementary Figure 4.010), TCA cycle (Supplementary Figure 4.002), and several subunits of the major regulatory complex of the oxidative phosphorylation process (Supplementary Figure 4.014) were upregulated in the papeda. As in SCM, in the highly acidic species lemon and citron, several genes involved in the TCA cycle and GABA shunt also displayed reduced expression during ripening (Borredá et al., 2022). Based on these observations, we propose that in the papeda, the generation of energy in the ATP form, is stimulated through the increase of the carbon flux *via* both glycolysis and fatty acid degradation, generating pyruvate and acetyl CoA, respectively, to fuel the Krebs cycle (Figure 6). The activation of the TCA cycle increases the production of both succinate, a substrate of complex II of the mitochondrial electron transport chain, and citric acid, that after transport to the cytosol may increase cytoplasmatic acidity to a detrimental level for normal cellular functions. Citric acid may be, then, stored in the vacuole, catalyzed to Acetyl CoA or further metabolized into glutamate entering the GABA shunt, that finally restores the carbon pool that the TAC cycle requires. We proposed in a previous work (Cercós et al., 2006), that in cultivated Clementine the GABA shunt, a powerful proton consuming reaction, is a very efficiently way to reduce both citric acid and cytoplasmatic acidity in ripe fruit flesh, while this current work suggests that this mechanism is active in developing fruitlets of wild citrus. Current consensus agrees that the regulation of acid metabolism in citrus, is basically focused on the generation of citric acid in the TCA, and its storage in the vacuole and later reduction in the cytosol through the GABA and ATP citrate lyase pathways (Cercós et al., 2006; Li et al., 2019; Sadka et al., 2019; Feng et al., 2021). However, our suggestion expands this concept identifying the regulatory TCA as a hub linking catabolism of fatty acids, production of organic acids and activation of oxidative phosphorylation, for the generation of energy as requested by growth and/or environmental demands. In this view, citric acid appears to be the major player in a system, that is balanced modulating its concentration and compartmentalization through processes that ultimately determine fruit acidity, a pivotal citrus organoleptic trait.

**TABLE 1:** Expression of genes involved in the biosynthesis of chemical defenses and associated compounds, in the pulp of developing fruitlets of ICH as related to SCM.

Chemical defense	Gene Id	Gene name	Compound	Expression
Alkaloids	LOC18037906	Vinorine synthase	Ajmaline	Up
Alkaloids	LOC112100740	Probable caffeine synthase 4	Caffeine	Up
Alkaloids	LOC18036840	Codeine *O*-demethylase	Morphine	Up
Alkaloids	LOC18037214	8-Hydroxygeraniol dehydrogenase	Vinblastine	Up
Alkaloids	LOC18042369	7-Deoxyloganetin glucosyltransferase	Secologanin	Up
Alkaloids	LOC18043348; LOC18046227	tyrosine/DOPA decarboxylase 5	Serotonin	Down
Alkaloids	LOC18041681	Tryptamine 5-hydroxylase (CYP71P1)	Serotonin	Down
Alkaloids	LOC18043348; LOC18046227	Tyrosine/DOPA decarboxylase 5	Dopamine	Down
Terpenoids	LOC112098571; LOC112098486	(R)-limonene synthase 1, chloroplastic-like	Limonene	Up
Terpenoids	LOC18045727	Beta-amyrin synthase	ß-amyrin	Up
Terpenoids	LOC18049179; LOC18049178	cytochrome P450 82G1	TMTT	Up
Terpenoids	LOC18051782	(3S,6E)-nerolidol synthase 1	DMNT	Up
Terpenoids	LOC18033168	(3S,6E)-nerolidol synthase 1	DMNT	Down
Terpenoids	LOC112095677	α-copaene synthase-like	α-copaene, (*E*)-β-caryophyllene and germacrene D.	Down
Terpenoids	LOC18053589	α-farnesene synthase	Farnesene	Down
Phytoalexins	LOC18031421	Cytochrome P450 76M5	Oryzalexin	Up
Phytoalexins	LOC18052043	Premnaspirodiene oxygenase	Solavetivone	Up
Phytoalexins	LOC18031586; LOC18051743	*Trans*-resveratrol di-*O*-methyltransferase	Pterostilbene	Up
Flavonoids	LOC18037475	Flavonol synthase/flavanone 3-hydroxylase	Kaempferol	Up
Flavonoids	LOC18037475	Flavonol synthase/flavanone 3-hydroxylase	Quercetin	Up
Flavonoids	LOC18037476	Flavonol synthase/flavanone 3-hydroxylase	Myricetin	Up
Cyanogenic glucosides	LOC18036876	Linamarin synthase 1	Linamarin	Up
Glucosinolate metabolism	LOC18049889	Flavin-containing monooxygenase FMO GS-OX-like 4	Methylsulfinylalkyl glucosinolates	Up

These changes differentially affected nitrogen and carbon allocation in both species. The most important difference in amino acid production was probably related to the degradation of leucine, isolecucine and valine, that was promoted in the papeda (Supplementary Table 4). These amino acids are degraded to Acetyl CoA and succinyl CoA that may thus fuel the TCA (Supplementary Figures 4.002, 4.025). In addition, upregulation in the papeda, of practically all genes controlling fatty acid degradation (Supplementary Figure 4.010), also appears to contribute to provide higher amounts of Acetyl-CoA to fuel the citrate cycle. In addition, the degradation of leucine produces hydroxymethylglutaryl, an intermediate of the mevalonate pathway in the terpenoid pathway, that may then be reinforced (Supplementary Figure 4.072). This pathway also provides precursors for monoterpenoid and isoquinoline alkaloid (Supplementary Figure 4.087), whose synthesis appears to be upregulated in the papeda as suggested above. Synthesis of major intermediates participating in carbon primary metabolism, for instance, was apparently more active in ICH (Supplementary Figure 4.045). In contrast, the number of upregulated DEGs involved in transport of hexoses and monosaccharides, including the vacuolar hexose transporter SWEET17 and NDR1/HIN1-like protein 26, required for correct sugar partitioning between source leaves and sink organs, was higher in SCM mandarin than in ICH (Supplementary Table 3).

The above results, overall, indicate that growth and development is rather restricted in inedible wild ICH, while secondary metabolism and the production of chemical defenses in particular (Table 1), are clearly upregulated. The dichotomy between growth stimulation versus activation of secondary metabolism is a common situation in the plant kingdom, which poses to plants, as sessile organisms, a dilemma that is resolved balancing the cost of investment in chemical defense and the availability of resources for its development. The payment of these costs, which takes place in the form of energy and carbon and nitrogen supplies, implies a proportional reduction in the growth and development of the plant (Mithöfer and Boland, 2012). It has been indicated, for instance, that pathogen and insect tolerance and resistance of domesticated citrus has generally declined compared with wild relatives (Bernet et al., 2005). We have previously shown through genomic analysis that citrus domestication tended to reduce chemical defenses involving cyanogenesis and alkaloids (Gonzalez-Ibeas et al., 2021b), while in the current work we expand this concept and show evidence that practically all major groups of chemical defenses, including alkaloids, terpenoids, glucosinolates, and cyanogenic glycosides are repressed in SCM. Therefore, the results support the suggestion that in our system, the papeda restricts its growth to allocate resources and energy to the production of defensive chemicals to escape herbivory.

### Differential gene expression in Ichang papeda versus Sun Chu Sha Kat mandarin: Upregulation of cold tolerance

The RNA-seq analysis, on the other hand, did not provide strong indications or evidence that both species behave differently facing abiotic stresses (Figure 1), except for several genes involved in cold and oxidative stresses (Supplementary Table 3). The set of DEGs related to cold was mostly characterized by the upregulation of genes implicated in cellular responses, including CORA, a cold and drought-regulated protein that showed the highest expression observed in this analysis (Jha et al., 2021). Key transcription factors governing cold response genes were also differentially expressed between both species. The most striking difference was related to the absence in the papeda of MYBS3 mRNA, a central transcription repressor that suppresses the DREB1/CBF-dependent signaling pathway regulating cold stress responses (Su et al., 2010). Also noticeable was the downregulation of negative regulators of abscisic acid such as MSI4 (Banerjee et al., 2017) and ERD15 (Kariola et al., 2006), components of stress responses, including freezing resistance. Furthermore, JUB1, a gene that modulates cellular H_2_O_2_ levels (Wu et al., 2012), enhancing tolerance to various abiotic stresses including cold (Fang et al., 2021), was upregulated (Supplementary Table 3). It is also worth to highlight that DEGs related to sphingolipid metabolism were repressed, except the conversion of ceramide to phytoceramide-1-phosphate (Supplementary Figure 4.054), that appears to be important for the resistance to cold (Dutilleul et al., 2015). Taken together, these observations might be related to the fact that ICH is the hardiest species in the genus Citrus (Swingle and Reece, 1967), tolerating both frost temperatures, even at –20°C, and damp conditions (Yang et al., 2017). It should be notice that these observations were made in samples not subjected to cold conditions, while the natural habitat of the papeda in montane regions of China (Yang et al., 2017) is rather chiller.

Since temperature stress increases the generation of ROS (Hasanuzzaman et al., 2013), this circumstance might be also connected with the enrichment of upregulated DEGs with several roles on antioxidant defense, that were detected in the Redox category (Supplementary Table 3), in the salvage pathway of the central coenzyme NAD + /NADH (Supplementary Figure 4.067) and in the synthesis of other coenzymes and vitamins, such as pantothenate (vitamin B5) and CoA (Supplementary Figure 4.068), riboflavin and flavin mononucleotide (Supplementary Figure 4.065), tocopherol and tocotrienol (vitamin E) (Supplementary Figure 4.013), and the compounds integrating vitamin B6 (Supplementary Figure 4.066).

### Differential gene expression in mandarin admixtures versus Sun Chu Sha Kat mandarin: Palatability increment

The comparative RNA-seq analyses between the four mandarin transcriptomes was characterized by the predominant downregulation in the three segregants of genes involved in both Abiotic Stress and Plant Defense categories, mostly participating in central roles and general responses, such as SRG1, regulating plant immunity. Similarly, pivotal genes implicated in the synthesis of relevant terpenoids, alkaloids and glucosinolates, and hence, in chemical defense, i.e., the pheromone β-farnesene, the homoterpene TMTT, the antifeedant limonene, the alkaloid scopolamine, or benzyl-glucosinolate, were downregulated. It is worth to note that in the wild papeda the expression of cytochrome P450 82G1 and (R)-limonene synthase 1, chloroplastic-like, the regulatory genes controlling the synthesis of TMTT and limonene, was relatively high (Supplementary Tables 2, 3), while in SCM, these genes were expressed at lower levels and in the three palatable mandarins, their expression was hardly detected. However, there were other defense genes (Supplementary Material) upregulated in the three segregants, an effect perhaps related to specific responses to local pathogen attacks and/or to the contribution of pummelo.

The three palatable mandarins showed downregulation of important genes controlling structural components of cell wall such as cellulose, expansins, pectins, and lignans and lignin. Expression of cytochrome P450 84A1 (ferulate 5 hydroxylase) involved in lignin biosynthesis, for example, was high in ICH, lower in SCM (Supplementary Table 2) and even lower in the three segregants (Supplementary Table 5). These observations might suggest that cell wall stiffening of the juice sacs in the fruit pulp or the peel of the segments is reduced, a characteristic that may be associated to a higher degree of palatability.

Other DGEs that may also affect palatability and taste were methanol *O*-anthraniloyltransferase, and 2-methylene-furan-3-one reductase, that were both upregulated and glutathione S-transferase L3, that was repressed. The first gene is an acyltransferase that catalyzes the formation of methyl anthranilate in the acridone alkaloid pathway, a substance of pleasant aroma, involved in the fragrance of Concord grapes (Wang and De Luca, 2005), that has been used in flavoring foods as mandarin candies or soft drinks (cited in Lee et al., 2019; Luo et al., 2019). Interestingly, expression of this gene is barely detectable in the inedible papeda, but its expression is relatively high in SCM and even higher in the three segregants. The enone oxidoreductase 2-methylene-furan-3-one reductase, on the other hand, renders furaneol, a key flavor compound in strawberries (Raab et al., 2006), that appear to be present only in fruits. The third gene, glutathione *S*-transferase L3 catalyzes the reduction of *S*-glutathionylquercetin to quercetin, a polyphenol that has a bitter flavor, in agreement with the suggestion that in comparison with wild citrus, cultivars show decreased secondary metabolite levels, such as bitterness compounds (Rao et al., 2021).

In citrus, taste is mainly dependent of the sugar and acid content of the juice. Regarding soluble sugars, the three palatable mandarins showed repression of sucrose synthase 2, probable galactinol-sucrose galactosyltransferase 2 and stachyose synthase, suggesting that sucrose conversion and catabolism in the three segregants is limited during this immature stage favoring sucrose accumulation. Citrus fruitlets appear to operate as main utilization sinks and sucrose synthase expression and activity at these immature fruit stages generally are relatively low (Iglesias et al., 2007; Li et al., 2019). During fruit development, upregulating of sucrose synthases enhances sink strength promoting sucrose and starch accumulation and increasing fruit size (Sadka et al., 2019; Feng et al., 2021). SCM also showed downregulation of two ERD6L sugar transporters that might be relevant for sugar accumulation in citrus fruits, especially sugar transporter ERD6-like 7, as reported in kumquats (Wei et al., 2021).

There were two sodium/hydrogen exchanger genes operating in low affinity electroneutral exchange of protons for cations, expressed in opposite directions in the three segregants. One of these, sodium/hydrogen exchanger 6 is the orthologous of CsNHX, that has been reported to be involved in the regulation of acidity levels in low-acid orange mutants (Wang et al., 2021). In addition, ATPase 10, plasma membrane-type, whose expression has been associated with citric acid accumulation in lemon juice sac cells (Aprile et al., 2011) was upregulated in the acidic mandarin.

### Differential gene expression in palatable mandarin admixtures versus Sun Chu Sha Kat mandarin: Downregulation of acidity

Since acidity is one of the fundamental traits determining palatability of citrus fruits and therefore a critical trait for citrus domestication (Wang et al., 2018; Butelli et al., 2019; Rao et al., 2021) we examined expression of pivotal genes previously suggested or proposed to regulate acidity in citrus (Supplementary Table 6). The analysis of gene expression in fruit pulp revealed that total acidity in palatable mature fruits was linked to downregulation of 5 genes, CitPH1 (magnesium-transporting ATPase, P-type 1), CitPH5.2 (ATPase 10, plasma membrane-type), CitAN1 (basic helix-loop-helix protein A), CitPH3 (WRKY transcription factor 44) and CitERF13 (ethylene-responsive transcription factor 13), in both developmental stages analyzed, developing and ripening fruits (Figure 3) in all three segregants. Of the genes studied, CitAN11 (protein TRANSPARENT TESTA GLABRA 1) was not repressed in any of the two stages, CitSO (protein PIN-LIKES 6) and CitVHA-c4 (V-type proton ATPase 16 kDa proteolipid subunit) were only downregulated in ripening fruits, whereas CitMAC9F1 (uncharacterized LOC180372899, CitPH4 (transcription factor MYB34) and CitPH5.1, in contrast, were downregulated in developing fruitlets.

The current study, while providing data on mandarins, complements the proposal of Strazzer et al. (2019), that shows that CitPH1 and CitPH5, two major downstream genes involved in vacuolar acidification, are highly expressed in ripe fruits of acidic varieties of lemons, oranges, and pummelos. Expression of both CitPH5 (Shi et al., 2015, 2019; Feng et al., 2021) and AN1 (Butelli et al., 2019; Strazzer et al., 2019; Wang et al., 2021), has been associated with citric acid accumulation in a number of studies, a subject revised in Huang et al. (2021).

CitPH1 and CitPH5 appear to act together since in petunia, PH1 may bind to PH5 to promote PH5 proton-pumping activity (Faraco et al., 2014). CitPH1 and CitPH5 expression, in contrast, is strongly reduced in acidless varieties of citrus and this downregulation is associated with mutations that disrupt expression of CitPH4 (MYB), CitAN1 (HLH) and/or CitPH3 (WRKY) transcription factors (Strazzer et al., 2019). These authors also report that CitMAC9F1, a gene of unknown function, is activated by the same transcription factors as CitPH1 and CitPH5 and that CitSO does not contribute to the differences in acidity. Our analyses are in line with these results since in both developing and ripening mandarin fruits CitPH1, CitPH5.2, CitAN1, and CitPH3 were downregulated. In addition, developing fruits showed repression of CitMAC9F1, CitPH4, and CitPH5.1, while ripe fruits exhibited further downregulation of CiSO (Figures 2, 3).

It has also been proposed that CitERF13 regulates citrate accumulation by directly activating the vacuolar proton pump gene CitVHA-c4102 (Li et al., 2016). In mandarins, CitERF13 was effectively downregulated in young and mature fruits while CitVHA-c4, that appears to be normally expressed during ripening (Feng et al., 2021) was repressed only in young fruit. The above observations suggest that although this set of genes, except CitAN11, is very likely involved in the control of acidity in the fruit pulp of mandarins, there may be precise patterns of regulation of gene expression, specifically controlling the accumulation and/or degradation of organic acids at each developmental stage.

In the work by Strazzer et al. (2019), the role CitPH1 and CitPH5, was mostly deduced comparing large differences in acidity between acidic and acidless varieties. They reported that those differences are produced by mutations disrupting the expression of those transcription factors that regulate the two ATPases. Consequently, the authors wonder if small acidity differences between varieties of the same group, may also be due to small differences in the expression of CitPH1/CitPH5. The evidence presented in this current work answers this question showing evidence that smaller differences in non-palatable acidic SCM and edible mandarins are equally correlated with the expression of CitPH1 and CitPH5 and that the range of this change is enough to determine its acceptance, suggesting that this circumstance was a pivotal domestication trait in citrus.

### Differential allele expression in palatable mandarin admixtures

The study on differential allelic expression on the pummelo introgressed areas of the three segregants presented in Figure 4 indicates that the contribution of pummelo to acidity did not play a critical role. The analysis detected, however, genetic targets that in principle appear to be contributors to other relevance traits, such as sugar transporters, cell wall modifying pectinesterases or auxin responses. For instance, expression of a major regulator of auxin action, the auxin receptor TIR1 (LOC18052162), in the pulp of developing fruitlets is low in the papeda, relatively high in SCM and higher in the three segregants. Interestingly, differential allelic expression was mostly characterized by the downregulation of MA alleles and the upregulation of the PU ones (Supplementary Figure 8). This fact suggests that mandarin domestication in part was certainly based on the selection and substitution of mandarin alleles by pummelo genes.

In conclusion, we propose that during the transition of inedible papedas to sour mandarins, domestication involved a first phase of major changes in the gene regulation of central pathways of the primary and secondary metabolism, characterized by both growth stimulation and reduction of distasteful chemical defenses. It is intriguing, that this reduction appears to affect to all main alkaloids, except dopamine and serotonin, two brain neurotransmitters regulating mood and emotion in humans. We also suggest that in a second phase, several edible attributes of mandarins, especially acidity, were progressively improved through specific changes. Lastly, several observations might indicate that the strong resilience of ICH to frost could be related to the regulation of relevant genes governing cold response.

## Data availability statement

The data presented in this study are deposited in the Sequence Read Archive (SRA database) repository, accession number PRJNA853264 (https://www.ncbi.nlm.nih.gov/sra/PRJNA853264).

## Author contributions

EP-R collected the samples and phenotype data, performed the data analysis, plot the results, designed the figures and tables, and reviewed and edited the original draft. CB advised the data curation. FT assisted reviewing and editing the original draft. MT conceived and conceptualized the study, conducted and supervised the research, interpreted the results, and wrote and edited the original draft. All authors contributed to the article and approved the submitted version.
