# Supplementary material for: Transcriptome analysis of the pulp of citrus fruitlets suggests that domestication enhanced growth processes and reduced chemical defenses increasing palatability
Source: Front Plant Sci. 2022 Sep 2;13:982683. doi: 10.3389/fpls.2022.982683 (PMC9478336; doi:10.3389/fpls.2022.982683)
Supplement: Supplementary file 1 [file Data_Sheet_1.PDF]

Supplementary Material

Supplementary Data

Gene Expression in Inedible Wild Ichang Papeda versus Acidic Sun Chu Sha Kat Mandarin

Lipids and Fatty Acids. The data (Supplementary Table 3) suggest that the expression levels of genes implicated in the regulation of fatty acid biosynthesis (Supplementary Figure 4.008) and elongation (Supplementary Figure 4.009) pathways, do not drastically differ between both species. However, the KEEG mapping revealed several interesting observations (Supplementary Table 2). One of the most relevant changes, for instance, was related to the upregulation in the papeda of practically all genes controlling fatty acid degradation (Supplementary Figure 4.071), very likely providing higher amounts of Acetyl-CoA to fuel the citrate cycle. It is also noticeable that in this species, 10 out of 11 DEGs regulating steroid biosynthesis, including lysosomal acid lipase/cholesteryl ester hydrolase (LOC18051398, EC:3.1.1.13, Supplementary Figure 4.012), that renders cholesterol, were downregulated, consistently with the repression of both squalene monooxygenase (LOC18033947 and LOC18033838, EC:1.14.14.17, Supplementary Figure 4.012) and the synthesis of brassinosteroids (Supplementary Figure 6). Another interesting observation in the sphingolipid metabolism is that in the papeda, DEGs are also mostly repressed except the conversion of ceramide to ceramide-P, a reaction catalyzed by ceramide kinase (LOC18031894, EC:2.7.1.138, Supplementary Figure 4.054, Supplementary Table 2), that appears to be participating in cold tolerance. Similarly, the synthesis of glycerol and triacylglycerol do not appear to be favored in ICH, in contrast to that of glycerol 3P and diacyl-glycerol 3P (Supplementary Figure 4.047, Supplementary Figure 4.049). In the peroxisome, two genes, acyl-CoA oxidase (LOC18036670 and LOC18047109, EC:1.3.3.6, Supplementary Figure 4.115) and a long-chain acyl-CoA synthetase (LOC18042917 and LOC1805469, EC:6.2.1.3, Supplementary Figure 4.115), acting in the ß-oxidation of fatty acids were upregulated. In other aspects of the lipid metabolism related to linoleic acid (Supplementary Figure 4.052), glycosphingolipids (Supplementary Figure 4.055 and Supplementary Figure 4.056), and ether lipid (Supplementary Figure 4.050), relevant observations were not obvious. Other results focused in glycerophospholipids and inositol phosphates, wax, and jasmonate are described in the previous parts devoted to membranes and transduction, cell wall, and hormones respectively.

Gametophyte. In this category, more than two thirds of 46 genes, mainly transcription factors and receptors implicated in processes related to fertilization, karyogamy or the formation of gametophytes, were expressed at lower levels in ICH (Supplementary Table 3).

Organ Development, Differentiation. This group comprised 60 genes (Supplementary Table 3), in general transcription factors, that are well known to play relevant roles in the regulation of growth and development of practically all plant organs, such as leaf, vascular tissue, seed, stoma, flower, meristem or root. Some of these genes control directional growth, cell fate, meristem elongation, or aerial development. As many as 50

genes of this set were downregulated in ICH, including 11 genes related to meristem growth and DA1 (LOC18040667) a major regulator of final seed size.

Chloroplast. This category was clearly downregulated (94/20) in ICH, since many genes involved in specific processes listed in Supplementary Table 3, such as photosynthesis, the Calvin cycle, and the synthesis of chlorophyll or starch, showed lower number of transcripts in this species. In addition, the mapping of genes to the KEGG pathways revealed that all DEGs involved in Photosystems II and I, the photosynthetic electron transport, including ferredoxin--NADP+ reductase, petH (LOC18037401, EC:1.18.1.2) and several subunits of the F-type H+-transporting ATPase, ATPF1G (LOC18050746 and LOC18055418), and all components of the light harvesting chlorophyll protein complex), were unequivocally downregulated (Supplementary Figure 4.015 and 4.016; Supplementary Table 2). The synthesis of both chlorophylls, a and b, was also repressed (Supplementary Figure 4.071, Supplementary Table 2).

Cell division. The comparison between both species also revealed that categories containing genes directly related to cell division were essentially downregulated. The category (Supplementary Table 3) included 93 genes, 76 of which were downregulated. It was subdivided in different groups, e.g. *Cell Cycle*, that mostly contained cyclins (16/7), *Cytokinesis*, genes involved in the orientation and formation of the phragmoplast and cell plate (10/2), *Mitosis*, genes primarily related to the mitotic spindle formation, and other involved in the regulation of the G1 phase of the cell cycle, microtubule-kinetochore conjugation, chromosome condensin complex or the control of mitotic checkpoints (22/3), *DNA repair*, comprising mostly transcripts coding for components of the double-strand break repair complexes, like the post-replicative DNA mismatch repair system (MMR) or the SMC5-SMC6 complex (7/3) and *DNA replication*, genes implicated in several complexes controlling DNA replication, such as the origin recognition complex (ORC), the GINS complex, the protein A complex (RPA/RP-A) or the cohesin complex (17/0). In addition, virtually all DEGs that mapped to KEGG pathways with roles on DNA replication (Supplementary Figure 4.099), base and nucleotide excision repair (Supplementary Figure 4.102, Supplementary Figure 4.103), mismatch repair (Supplementary Figure 4.104) and homologous recombination (Supplementary Figure 4.105) were downregulated.

Meiosis. This category (Supplementary Table 3) included mostly downregulated genes (9/1), that are required for normal meiosis, including meiotic recombination and crossovers, pairing of homologous chromosomes, formation of tetrad of microspores or chromosome segregation.

Cytoskeleton. Another category related to growth that exhibited higher number of down- (34) than upregulated genes (11), was Cytoskeleton (ST 2832), that included genes associated with the regulation of *Actin* (3/5) and *Microtubule organization* (26/8) and hence, with the cytoskeletal organization and cell morphology. The majority of the transcripts included in the microtubule group corresponded to motor proteins of the kinesin-like protein KIN type (24/2), and practically all of them, were downregulated (Supplementary Table 3).

Cell Wall. The category included 74 genes that were classified in three groups, genes mostly coding for enzymes implicated in the own synthesis of the components and

structural proteins of the cell wall (57), genes related to the extracellular *Matrix* (7), and genes implicated in the formation of the *Cuticular wax* (10). In the first group of genes, downregulation was in general more prominent that upregulation (34/23) a circumstance that was observed for cellulose synthases, extensins, arabinogalactan proteins, arabinosyltransferases, expansins, polygalacturonases, pectinesterases, xyloglucan acetyltransferases, xyloglucan glycosyltransferase and other transcripts implicated in secondary wall formation. In contrast, pectinesterase inhibitors, ligases, glycosyltransferases, galacturonosyltransferases and xyloglucan endotransglucosylases (XET) were in general up. In the second and third groups, involving genes related to the extracellular matrix (cell surface adhesion proteins and signaling receptors) and to the cuticular wax (ABC transporters of the G family, lipid-transfer proteins, and alkane hydroxylases), respectively, no clear differences between the abundance of down and up regulated genes were observed (Supplementary Table 3). In addition, the papeda showed downregulation (Supplementary Figure 4.011, Supplementary Table 2) of a fatty acid omega-hydroxy dehydrogenase, ACE, (LOC18036702, 1.1.-.-, involved in the formation of oxo fatty acids in the suberin biosynthesis, and of the aldehyde decarbonylase CER1 gene (LOC18049734, EC:4.1.99.5, associated with the production of wax.

Cell Expansion. The number of down and up regulated genes in this category was only slightly different (8/11). The category mainly contained regulatory genes of cell expansion, e.g., growth-regulating factors, receptor-like serine/threonine-protein kinases, or wall-associated receptor kinases. For example, one of the downregulated genes, was protein LONGIFOLIA 1 (LOC18048398, Supplementary Table 3), that promotes longitudinal polar cell elongation.

Receptors and Protein Kinases. The 108 DEGs include in this category (Supplementary Table 3) were those not assigned to any specific cellular process, out of which 63% were expressed at lower levels in the papeda.

Membrane, ER and Signalling. Members of this category included genes related to the Rho GTPase signaling complex, calcium sensors and signaling transduction and other second messengers, such as cAMP, inositol triphosphate and diacylglycerol, phosphatidylinositol 3-kinase signaling, signal recognition particle, and genes involved in the synthesis of phosphatidylcholine and other membrane components (Supplementary Table 3). In this category downregulation was not so prominent (40/33), although some gene families like that of proteins IQ-DOMAIN 1 and 14 were manly expressed at lower levels (8/2). The synthesis of membrane glycerophospholipids, such as phosphatidylethanolamines and phosphatidylcholines, also appears to be repressed in the papeda (Supplementary Figure 4.049). In the inositol phosphate metabolism (Supplementary Figure 4.048, Supplementary Figure 4.108, Supplementary Table 2), ICH showed downregulation of genes coding for inositol mono-, bis-, tris-, pentakis-, and hexaphosphate (phytate). However, the 2 last steps in the synthesis of inositol-1,3,4P3 (IP3) which encompass 1-phosphatidylinositol-4-phosphate 5-kinase, PIP5K (LOC18042642, EC:2.7.1.68), that generates phosphatidyl inositol-4,5 biphosphate, and phosphatidylinositol phospholipase C, gamma-1, PLCD (LOC18044207, LOC18043460 and LOC18044208, EC:3.1.4.11), that hydrolyzes this last compound to diacylglycerol and IP3, were upregulated, suggesting that the availability of these two second messenger molecules is superior in ICH.

Chromatin, Histones. This category was composed of 27 genes mostly related to histones, chromatin assembly and remodeling factors, and histone methyltransferases. In this group, 22 genes, including 13 transcripts coding for the 4 core components of nucleosome and the chromatin fiber, were downregulated (Supplementary Table 3).

Transcription. In this category, as many as 75 genes out of 118 were downregulated. Most DEGs included in this category were transcription factors (54/36) and the rest of members were divided in 2 groups, *Post-transcription* including splicing (5/1) and gene silencing (6/2), and *RNA polymerase* (10/4) (Supplementary Table 3). The KEGG mapping showed that in general, minor differences were observed among the basal transcription factors for RNA polymerase II (Supplementary Figure 4.098) and the core and specific subunits of the polymerases (Supplementary Figure 4.097). In addition, most DEGs included in nucleocytoplasmic transport (9/3; Supplementary Figure 4.094) and mRNA surveillance pathway (9/4, Supplementary Figure 4.095) were downregulated and those implicated in RNA degradation were upregulated (Supplementary Figure 4.096).

Nucleic Acids Processing. The category was populated to a large degree with helicases, DNA methyl transferases, ligases, endonucleases, ribonucleases, primases and other factors acting on nucleic acids. Downregulated genes were predominant in the papeda, since 19 genes out of 27 were expressed at lower levels (Supplementary Table 3).

Translation. The category was dominated by upregulated genes (18/25) that mostly corresponded to the group of 40 S and 60 S *Ribosomal proteins*, and genes implicated in ribosome biogenesis and other factors controlling translation initiation and the termination of nascent peptide synthesis (Supplementary Table 3).

Trafficking, Vesicles. The category was characterized by a higher number of upregulated genes (9/16), mostly involved in the secretion of vesicles from ER, via the Golgi up to the trans Golgi network, regulation of clathrin and non-snare proteins, autophagy, endosome trafficking and recycling, and in the processes of micropinocytosis, endocytosis and exocytosis (Supplementary Table 3). A set of upregulated genes representative of this category was that of the GTP-binding proteins generally associated with the regulation of membrane trafficking, for instance, SAR1 (LOC112096942, Supplementary Table 3 and LOC18050081, EC:3.6.5.-, Supplementary Figure 4.112, Supplementary Table 2), a GTPase regulating the formation of Golgy bodies. Other upregulated DEGs involved in protein processing in the ER represented in Supplementary Figure 4.112 (Supplementary Table 2) were Sec61 (LOC18052999) and TRAP (LOC18035022), components of the translocon, a complex that translocates polypeptides, or several genes of the ERAD (ER-associated degradation), such as Hsp40 (DNAJA2, LOC18052272), NEF (HSPBP1, LOC18039711), Ufd1 (LOC18033916) and DUB (ATXN3, LOC18038522), a previous step to the proteasome. Upregulated genes involved in ER export included signal peptidases, translocation channels and related proteins of the Sec dependent pathway (Supplementary Figure 4.101, Supplementary Table 2). Furthermore, upregulated genes implicated in endocytosis were represented by clathrins, Rab proteins (small G proteins attached to vesicles), VPS (vacuolar protein sorting) and CHMP4 (charged multivesicular body proteins) (Supplementary Figure 4.113, Supplementary Table 2).

Ubiquitination. The category was comprised mostly by upregulated transcripts (26/38; Supplementary Table 3) involved in *Proteasome*, specifically major ubiquitin players such as the E3 ubiquitin-protein ligase complexes (13 genes, CUL3-RBX1-BTB; Elongin BC-CUL2/5-SOCS-box protein), or the E2s enzyme SCF (SKP1-CUL1-F-box protein)-type complexes (3 genes), in addition to chaperones and chaperonins, polyubiquitins and proteasome subunits. Supplementary Figure 4.112 shows additional upregulated genes located in the ER membrane or in the cytoplasm, belonging to the ubiquitin ligase complexes, while Supplementary Figure 4.110 illustrates that practically all 9 reactions describing the E2 (ubiquitin-conjugating enzyme) were upregulated.

Plant Defense. This category containing the highest number of DEGs (263), was enriched with downregulated genes (150) displaying a wide range of disease resistance proteins against pathogens (Supplementary Table 3). Among them, some are classified as general defense proteins involved in immunity, hypersensitive response, or pathogen responses, while others are defined by anti-fungal, -bacterial, and -viral activities.

Cofactors and Vitamins. DEGs implicated in the synthesis of cofactors and vitamins are listed in Supplementary Table 3, and other genes related to this category involved in different pathways are presented in Supplementary Figure 4.062 and Supplementary Figure 4.064-4.070. In general, the number of DEGs in these processes is too short to extract valuable remarks, although maybe is worth to mention that the synthesis of pantothenate (vitamin B5) and CoA, riboflavin and flavin mononucleotide, nicotinamide adenine dinucleotide, and the compounds integrating vitamin B6 show a tendency towards upregulation in the papeda.

DEGs included in the categories **Cell Death** (8/2, genes regulating programmed cell death, for instance, aspartic proteinases, co-chaperones…), **Light Signaling** (13/13, mostly transcription factors and photoreceptors involved in light-signal transduction), **Seed, Embryo Development** (5/10, seed storage, LEA proteins and embryo development) and **Flowering** (13/12, transcription factors, receptors and proteins related to FT controlling the flower-promoting signal), in principle do not appear to provide unambiguous information useful to accomplish the objectives proposed in this work (Supplementary Table 3).

The KEEG mapping also suggested that the amino sugar/nucleotide sugar metabolism (Supplementary Table 2) was also apparently more active in ICH than in SCM (Supplementary Figure 4.045). For instance, last regulatory steps controlling the synthesis of major intermediates participating in carbon metabolism, such as mannose-6P, UDP-L-arabinose, UDP-glucuronic acid, UDP-glucose, UDP-rhamnose, UDP-galactose and pectin were unequivocally upregulated. However, there were not too many differences in the expression levels of genes participating in N-glycan biosynthesis (Supplementary Figure 4.043) or glycosaminglycan degradation (Supplementary Figure 4.046).

Gene Expression in Palatable Pummelo/Mandarin Genetic Admixtures versus Acidic Sun Chu Sha Kat Wild Mandarin

Raw expression data in this section are presented in Supplementary Table 5, although the number of genes included in the below categories are in general too short to support a solid enrichment analysis.

Plant defense. The most populated category was Plant Defense (63), that surpassed even the category of uncharacterized process including genes with undetermined physiological roles (39). In comparison with SCM, the number of genes down- and upregulated in the three segregant mandarins was identical (31/32). The majority of these genes are classified as disease resistance proteins, that guard the plant against pathogens containing an avirulence protein, such as DSC1 like proteins (5/4), TMV resistance proteins N (3/0), or the RGA gene family (4/9). Several of them recognize fungal effectors and elicitors of *P. infestans, P. syringae, T. viride or C. fulvum*, modulate herbivory resistance, or act as triggers of hypersensitive response (HR). The three TMV resistance protein N detected, for instance, were downregulated in the palatable mandarins, as several genes with general responses and central roles in resistance, such as RPM1-interacting protein 4 (LOC18033466), ABC transporter G family member 34 or HAK1 (LOC18039121). Conversely, all 4-disease resistance protein At4g27220 (LOC18040211, LOC112097691, LOC18042654 and LOC18041183) and osmotin-like protein (LOC18035225) were upregulated in segregants. In this category other upregulated genes are glucan endo-1,3-β-glucosidases (LOC112101121 and LOC1803342) with roles in plant defense against fungal pathogens and β-glucosidase 17 (LOC18043267), regulating the synthesis of scopoletin/scopoline, coumarins that have deterrent effect on herbivores.

Abiotic stress. Most DEGs in the Abiotic stress category (6/1) were downregulated in the three segregants.

Amino acid metabolism. Most DEGs in the Amino acid metabolism category (5/1) were downregulated in the three segregants. Aminotransferases involved in amino acid degradation were predominant.

Cell Division. This category encompassed 6 genes which showed a down to up rate of 0.5.

Cell Wall. This group contained relevant genes (8/8) controlling major structural components of cell wall. Downregulated genes were cellulose synthase-like protein E1 (LOC18055183), expansin-like B1 (LOC18048704, Supplementary Table 5), pectin acetylesterase 8 (LOC18040742), pectinesterase/pectinesterase inhibitors (LOC18053206, LOC112095422 and LOC18040524), protein trichome birefringence-like 8 (LOC18038166, Supplementary Table 5), or cytochrome P450 84A1, which is a ferulate 5-hydroxylase involved in phenylpropanoids metabolism (LOC18036838), involved in lignin biosynthesis, were all downregulated.

Transcription. This category was populated with transcription factors and RNA polymerases and related factors, most of them repressed (10/2).

Receptors, Kinases, Transduction. The category was composed of 14-3-3 proteins, F-box/FBD/LRR-repeat proteins, and serine/threonine-protein kinases. Upregulated genes (4/12) were predominant.

Translation. The number of up and downregulated transcripts related to tRNA and ribosome biogenesis in this category were similar (5/3),

Ubiquitination. This category containing a similar number of up and down regulated genes (4/3), implicated in proteasomal degradation.

Membranes, ER, and Signaling. The group of genes (6/4) included in this category, such as adenylate cyclases, potential calcium sensors, or components of the GTPase signaling complex, did not showed a prevalent expression pattern.

Redox. DEGs clustered in the Redox category (5/5), mostly cytochromes, L-ascorbate oxidase, and probable glutathione S-transferases, contained a similar number of up and down regulated genes. In this category, the senescent protein SRG1 (LOC18054377) regulating plant immunity was repressed in the three palatable mandarins.

Trafficking, Vesicles. Synthesis of N-glycans was probably downregulated since α-mannosidase (LOC18036311), the first committed step of this pathway was repressed, although several other genes included in this category (3/5) were mainly upregulated.

Organ development, Differentiation. This category included several 3-oxo-Delta(4,5)-steroid 5-β-reductases involved in xylem and phloem pattern formation, that were mostly upregulated (1/3, LOC18041865, LOC18053615, LOC18041176 and LOC18041175).

Chloroplast. Flowering. (4/0), and **Gametophyte** (5/1) These were tree categories focused on growth and development, showing mostly downregulation, (13/1, 4/0, and 5/1, respectively).

Hormonal Regulation. In this category (14/7), most DEGs related to ABA, auxins, cytokinins, ethylene, jasmonic acid, polyamines and salicylic acid were downregulated.

Cell Death. Light Signaling. **Meiosis**. **Phytoalexins**. **S-adenosylmethionine Metabolism**. **Nucleobases**. **Seed, Embryo, Development**. For these categories, the number (1/2, 3/2, 0/1, 0/1,0/2, 1/3 and 2/2) or relevance of the DEGs was too scarce to suggest unambiguous comments.

Lipids and fatty acids. Among the upregulated genes in this category (5/7), there were two genes implicated in elongation of fatty acids (LOC18048831 and LOC18033122), a 3-ketoacyl-CoA synthase 1 (LOC18048831) relevant for the synthesis of cuticular wax and suberin, and an elongation of fatty acids protein A-like (LOC112098405). Other two genes coding for peroxisomal enzymes (LOC18050645 and LOC18055962) participating in the long-chain fatty acid catabolic pathways were also upregulated. Repressed genes were an additional 3-ketoacyl-CoA synthase 5 (LOC18033226), CDP-diacylglycerol--inositol 3-phosphatidyltransferase 1-like (LOC112096415), involved in phospholipid biosynthetic process and sterol 3-β-glucosyltransferase UGT80A2 (LOC18039007) controlling the biosynthesis of sterol glucosides, the most abundant sterol derivatives in higher plants, especially in seeds.

Flavonoids. This category contained similar number od down and up regulated genes (6/6). Although three flavonoid 3'-monooxygenases (LOC18032922, LOC18052870 and LOC18053379) were indistinctly down and up regulated in the three segregants, two central regulatory genes of the pathway, chalcone-flavonone isomerase (LOC18043493) and naringenin,2-oxoglutarate 3-dioxygenase (LOC18036490) were upregulated. Anthocyanidin 3-O-glucosyltransferase (LOC18039058) and anthocyanidin 5,3-O-glucosyltransferase (LOC18032110), participating in the biosynthesis of anthocyanins were also similarly upregulated. Further metabolism appears to be downregulated, for instance, the reduction of S-glutathionylquercetin to quercetin (LOC18043062), the conversion of phloretin to phlorizin (LOC18039246), the biosynthesis of lignans

(LOC18041854), phenylpropanoid monomers of the lignin polymers, or the acylation of the anthocyanin glucose (LOC18032737). As mentioned in the Results section of the manuscript, mandarins do not contain anthocyanins.
