# Supplementary figures and images for: Transcriptome analysis of the pulp of citrus fruitlets suggests that domestication enhanced growth processes and reduced chemical defenses increasing palatability (part 2 of 2)
Source: Front Plant Sci. 2022 Sep 2;13:982683. doi: 10.3389/fpls.2022.982683 (PMC9478336; doi:10.3389/fpls.2022.982683)

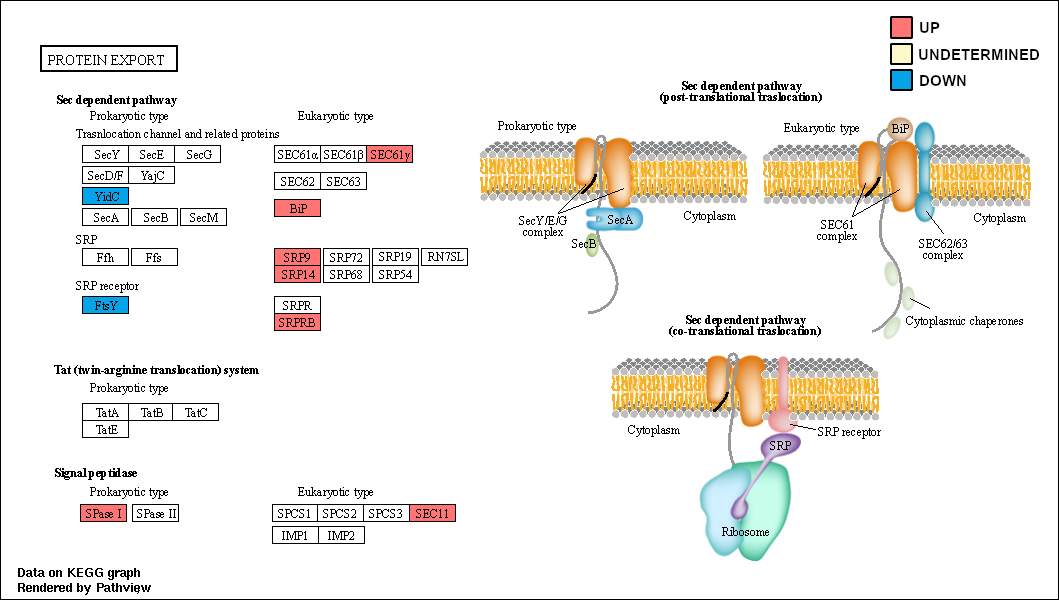

Supplement: Supplementary file 2 [file Data_Sheet_2.ZIP › Supplementary_Figure_4/Supplementary_Figure_4.101.png]

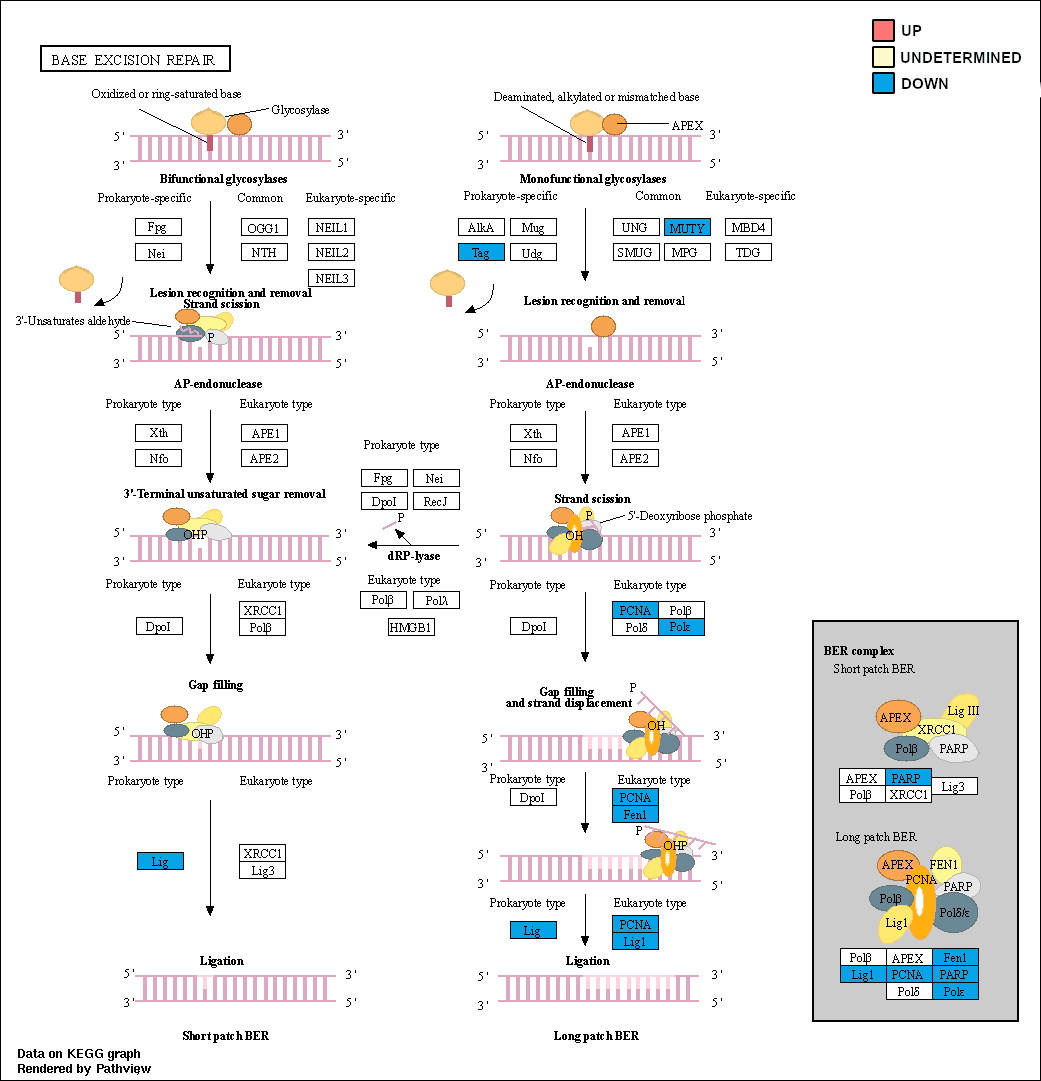

Supplement: Supplementary file 2 [file Data_Sheet_2.ZIP › Supplementary_Figure_4/Supplementary_Figure_4.102.png]

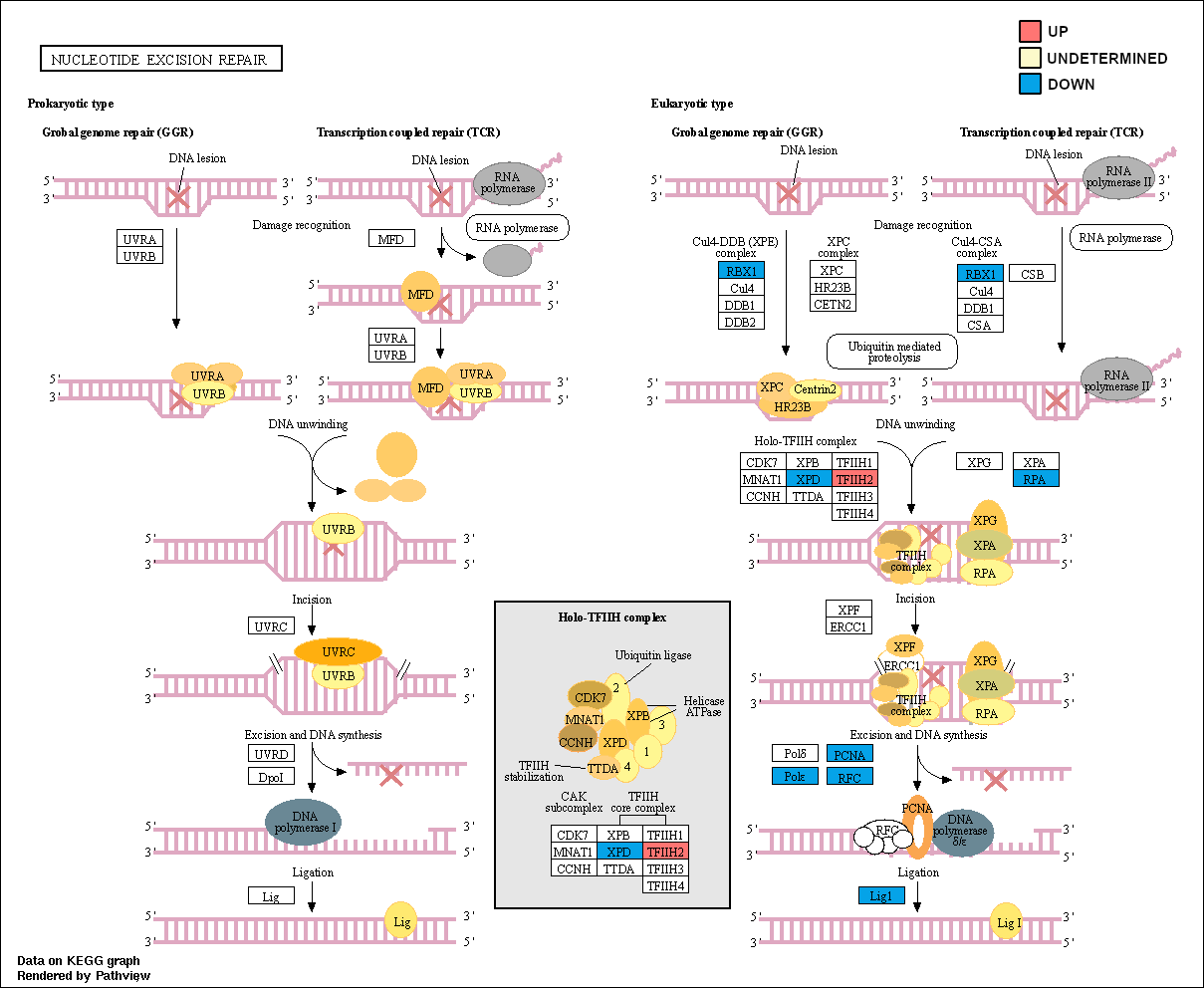

Supplement: Supplementary file 2 [file Data_Sheet_2.ZIP › Supplementary_Figure_4/Supplementary_Figure_4.103.png]

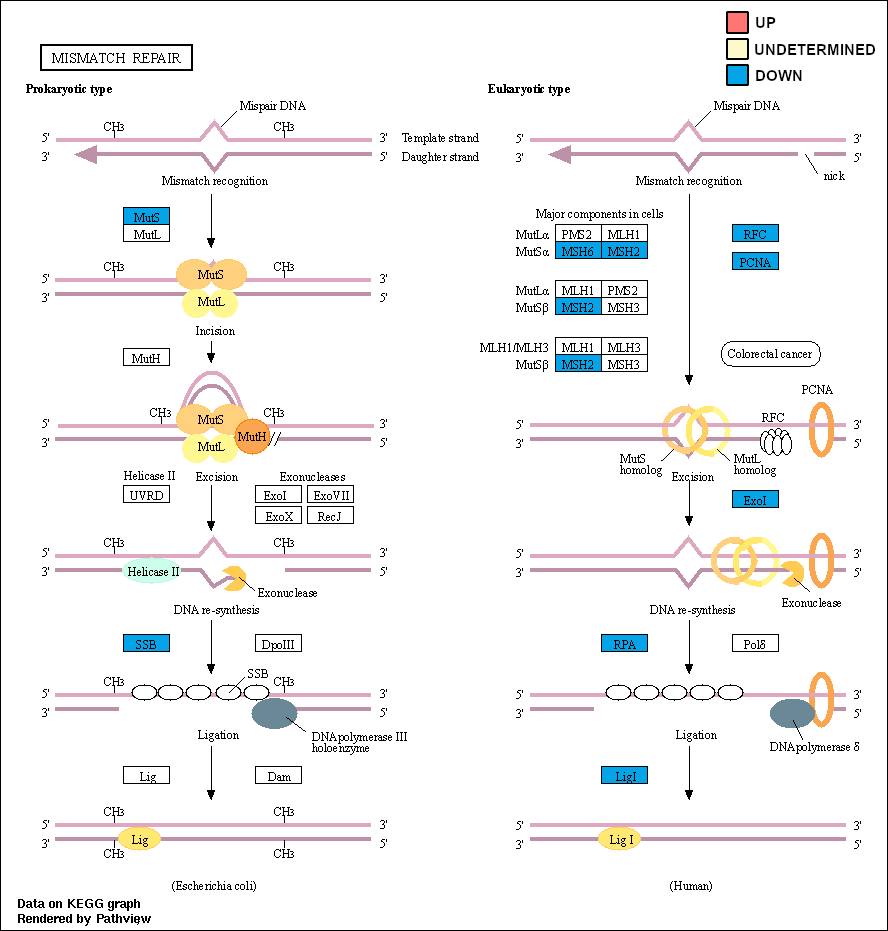

Supplement: Supplementary file 2 [file Data_Sheet_2.ZIP › Supplementary_Figure_4/Supplementary_Figure_4.104.png]

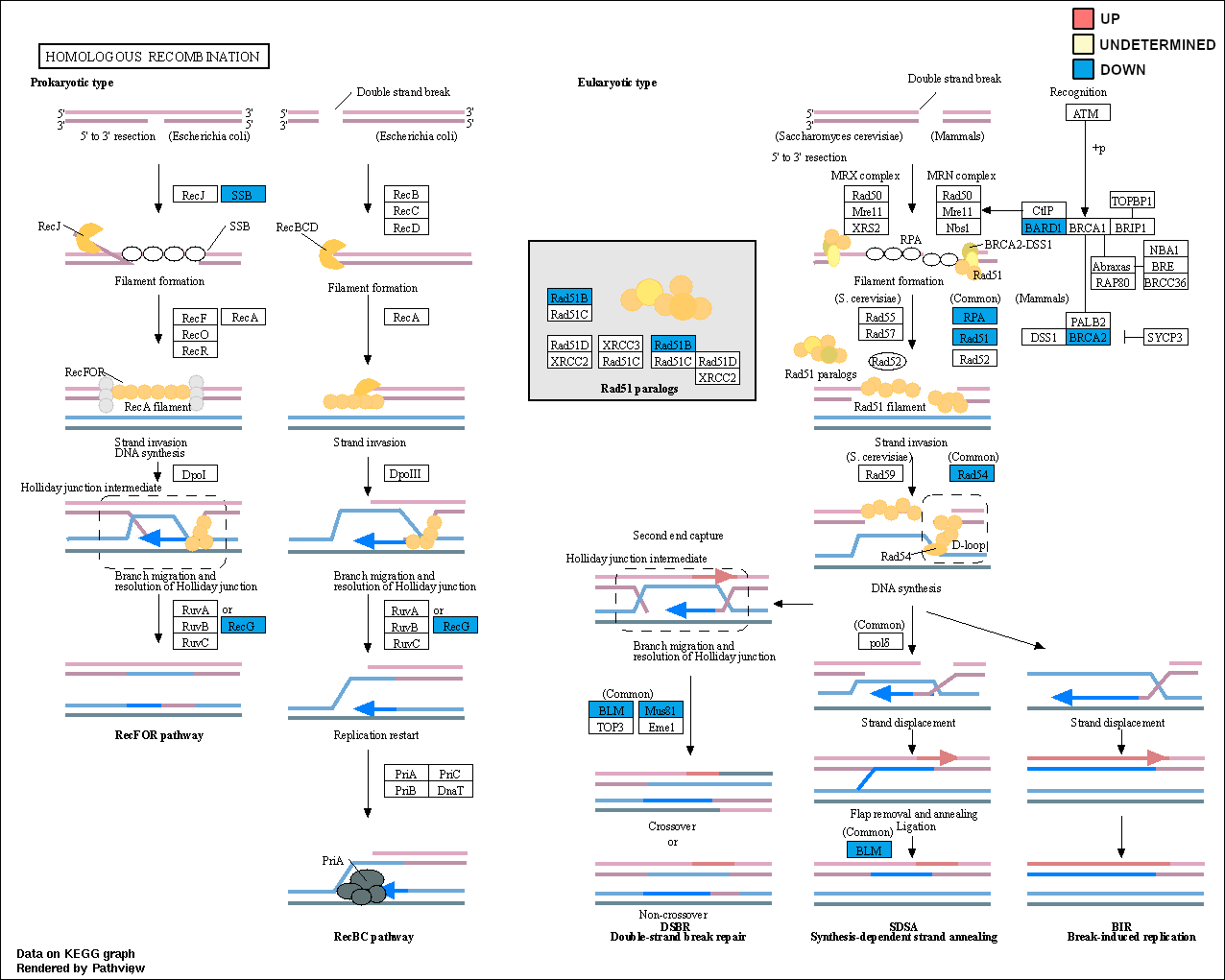

Supplement: Supplementary file 2 [file Data_Sheet_2.ZIP › Supplementary_Figure_4/Supplementary_Figure_4.105.png]

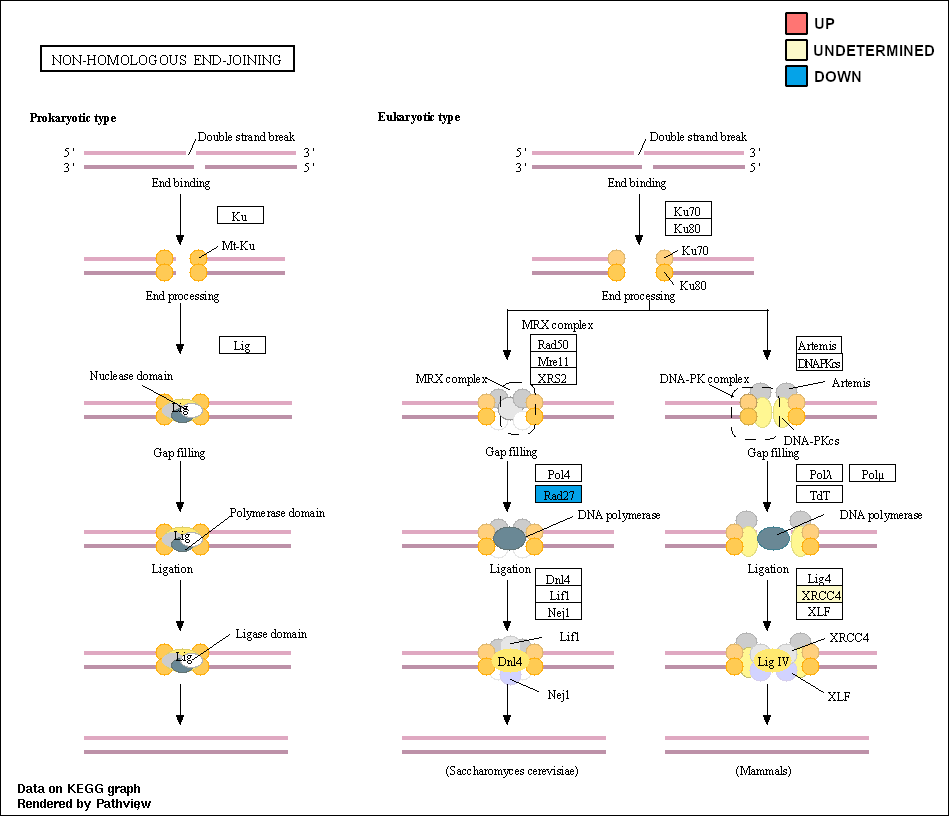

Supplement: Supplementary file 2 [file Data_Sheet_2.ZIP › Supplementary_Figure_4/Supplementary_Figure_4.106.png]

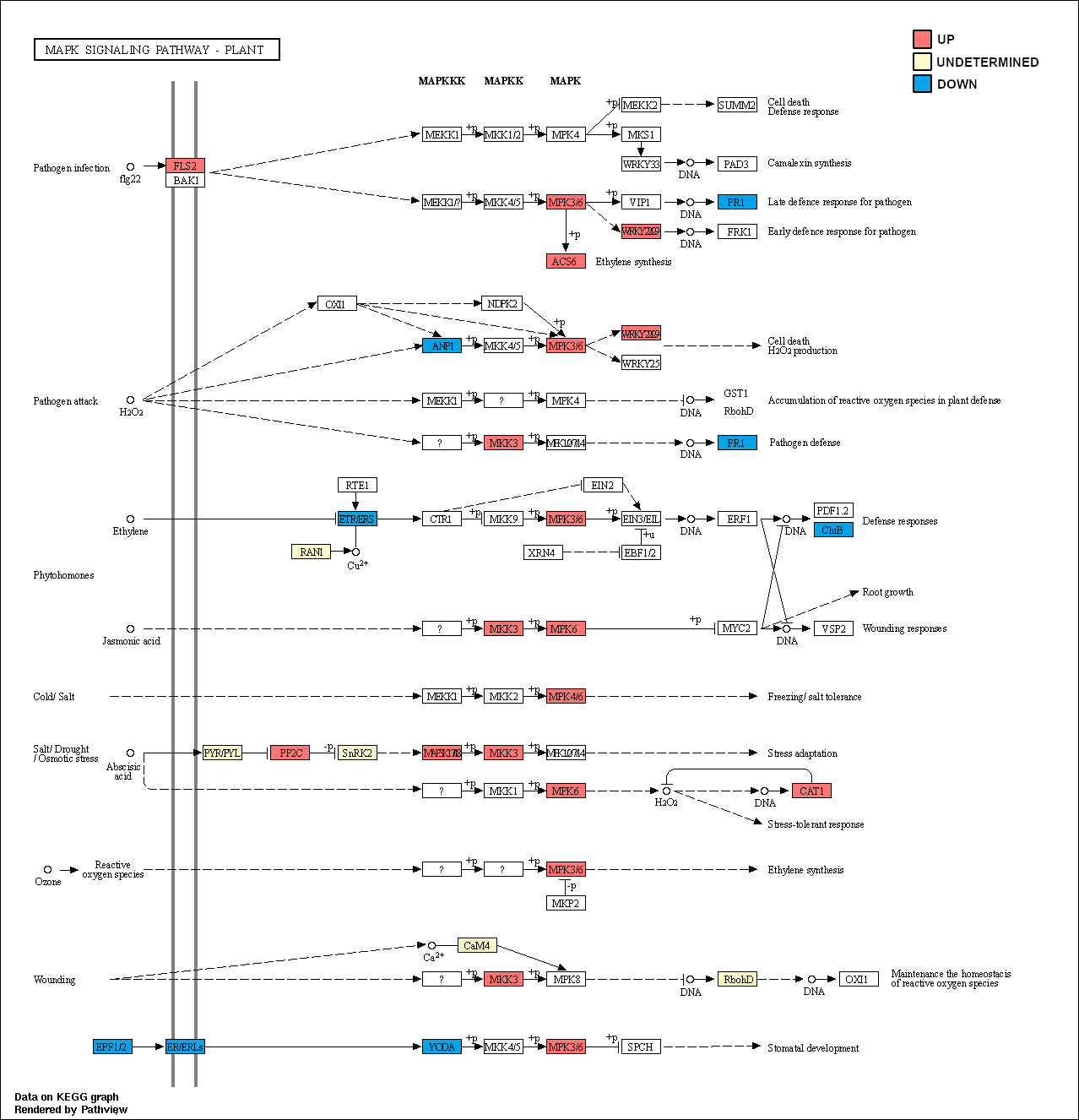

Supplement: Supplementary file 2 [file Data_Sheet_2.ZIP › Supplementary_Figure_4/Supplementary_Figure_4.107.png]

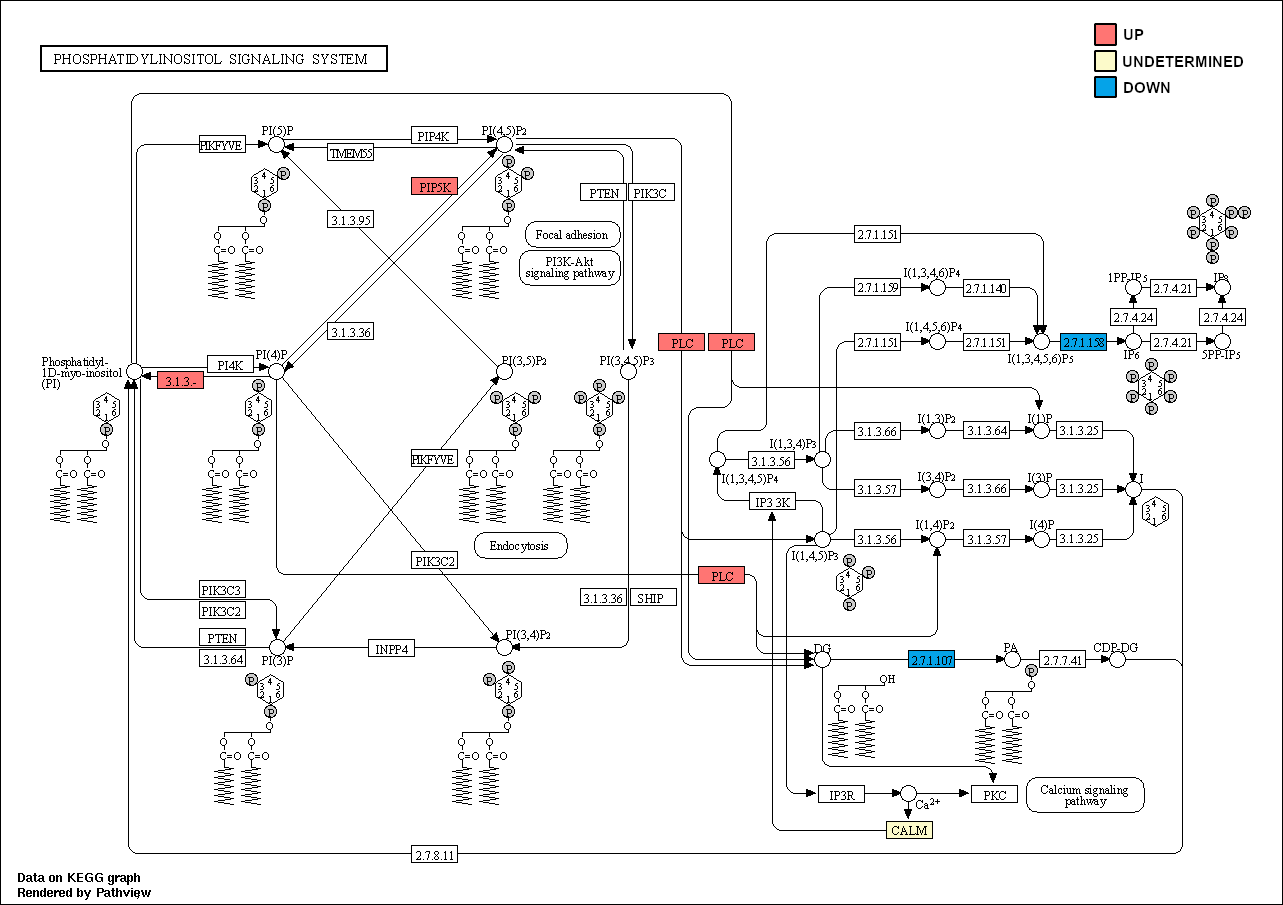

Supplement: Supplementary file 2 [file Data_Sheet_2.ZIP › Supplementary_Figure_4/Supplementary_Figure_4.108.png]

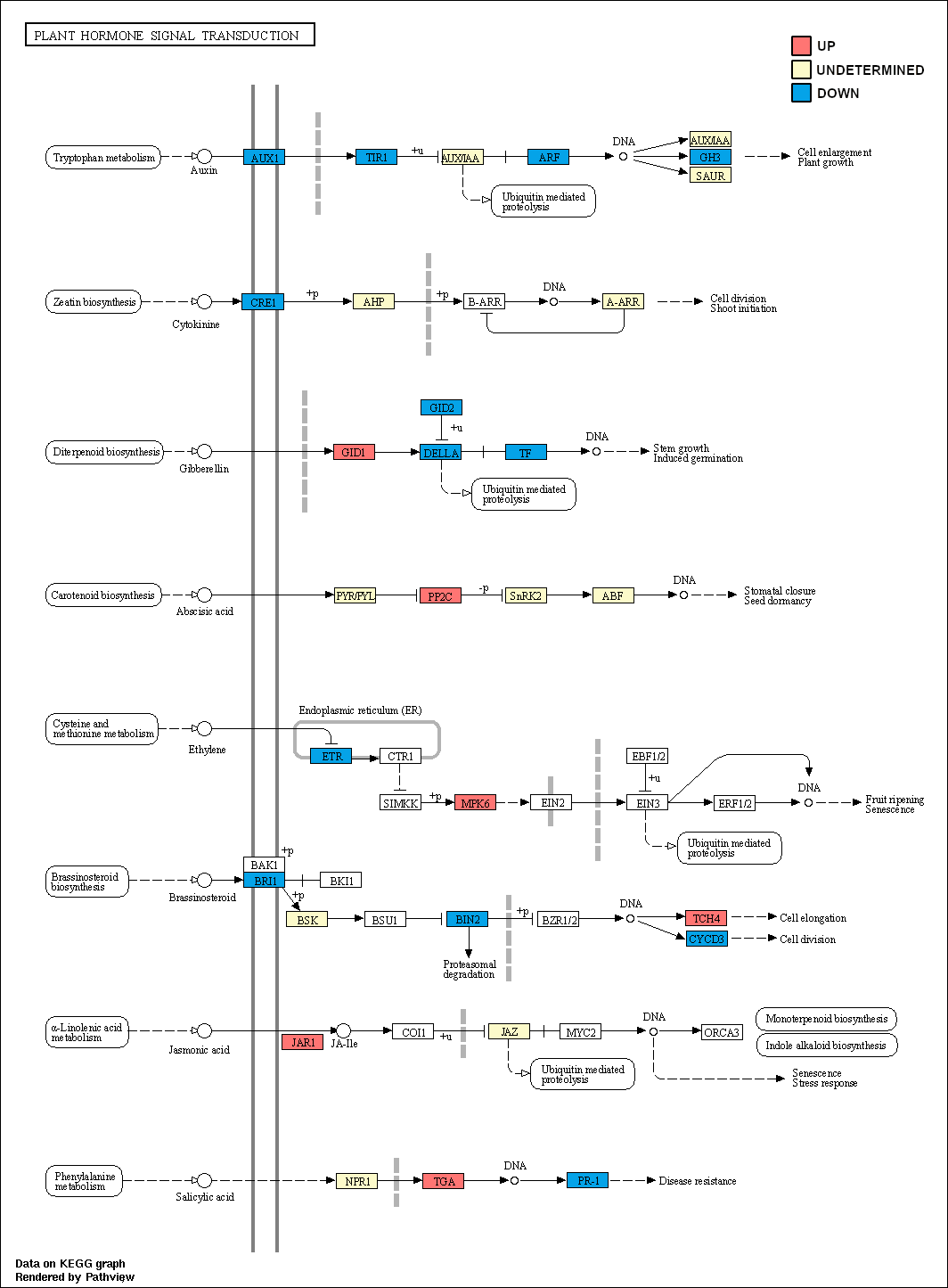

Supplement: Supplementary file 2 [file Data_Sheet_2.ZIP › Supplementary_Figure_4/Supplementary_Figure_4.109.png]

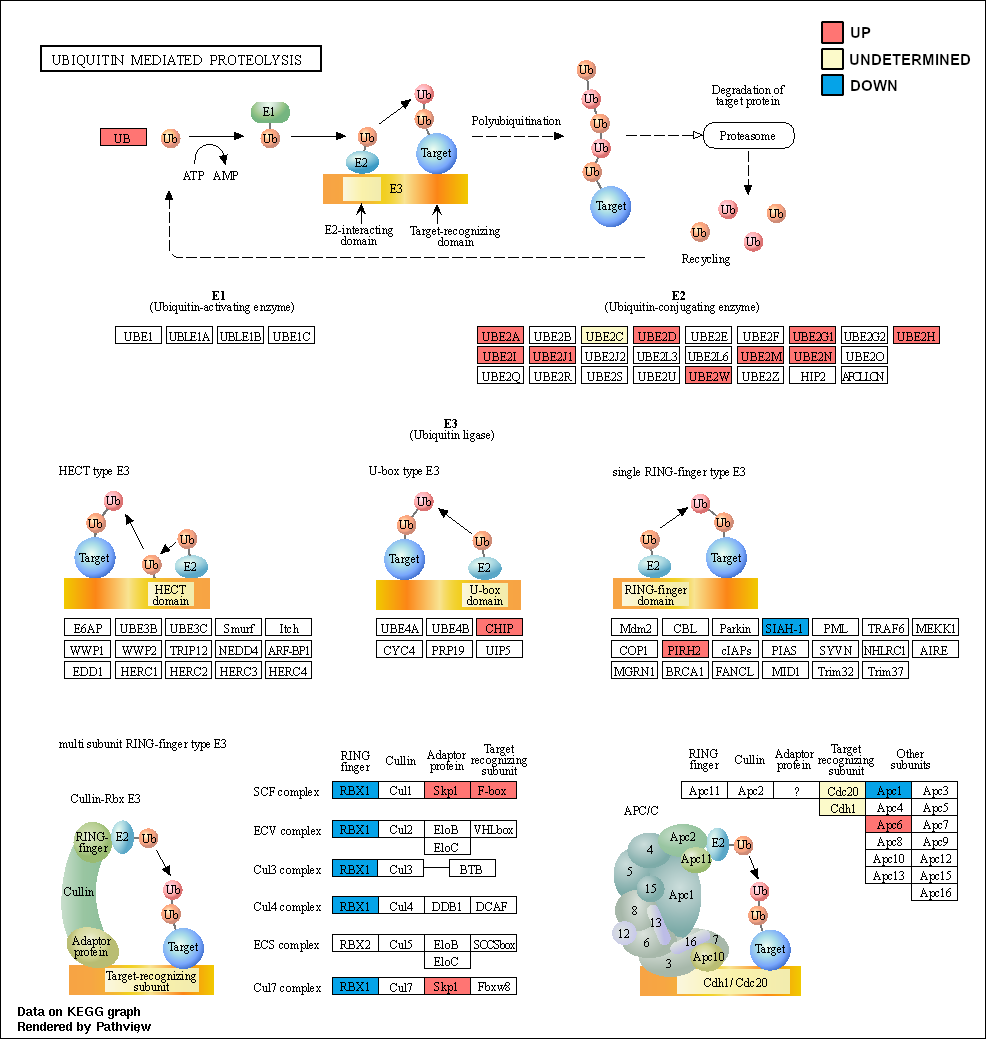

Supplement: Supplementary file 2 [file Data_Sheet_2.ZIP › Supplementary_Figure_4/Supplementary_Figure_4.110.png]

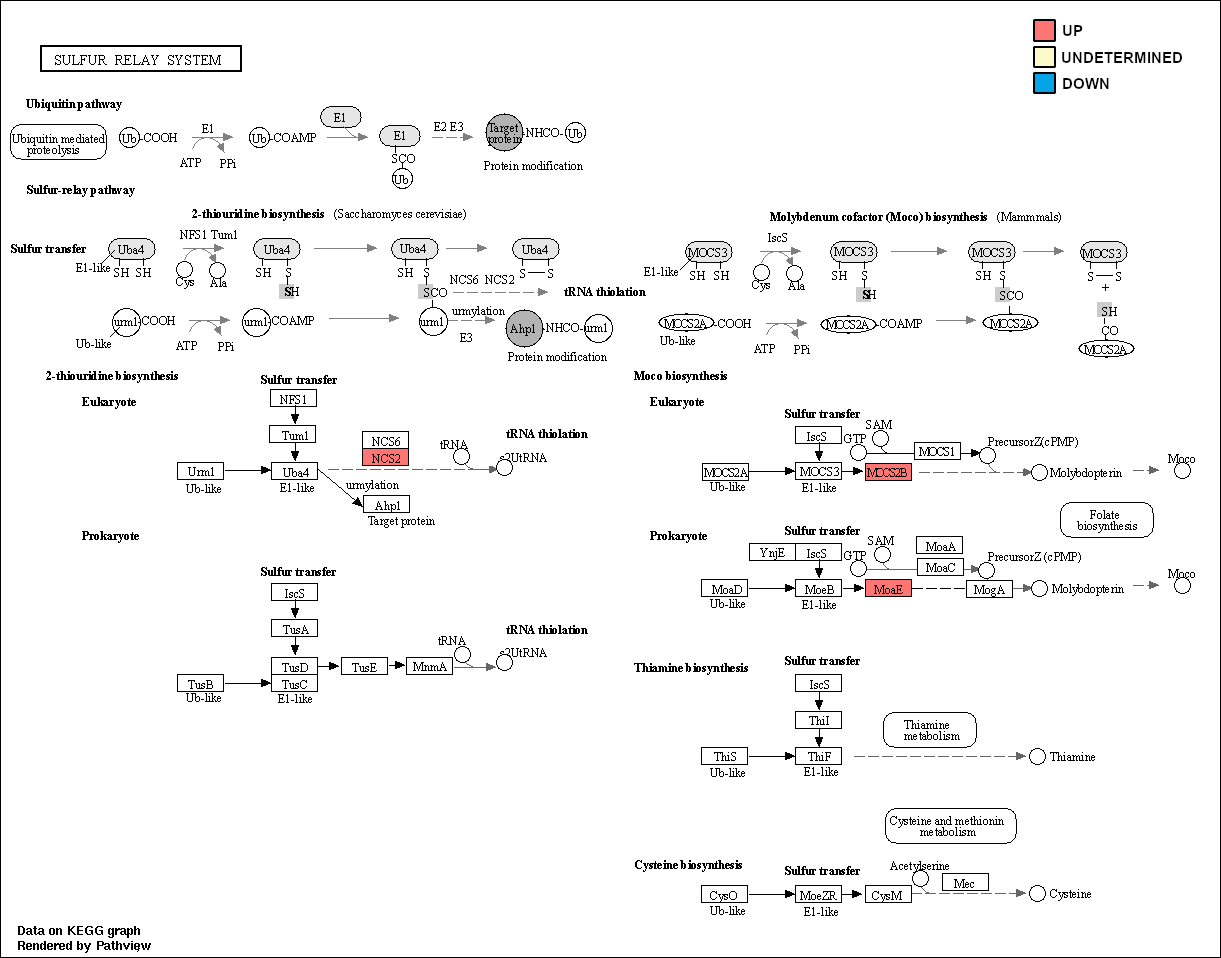

Supplement: Supplementary file 2 [file Data_Sheet_2.ZIP › Supplementary_Figure_4/Supplementary_Figure_4.111.png]

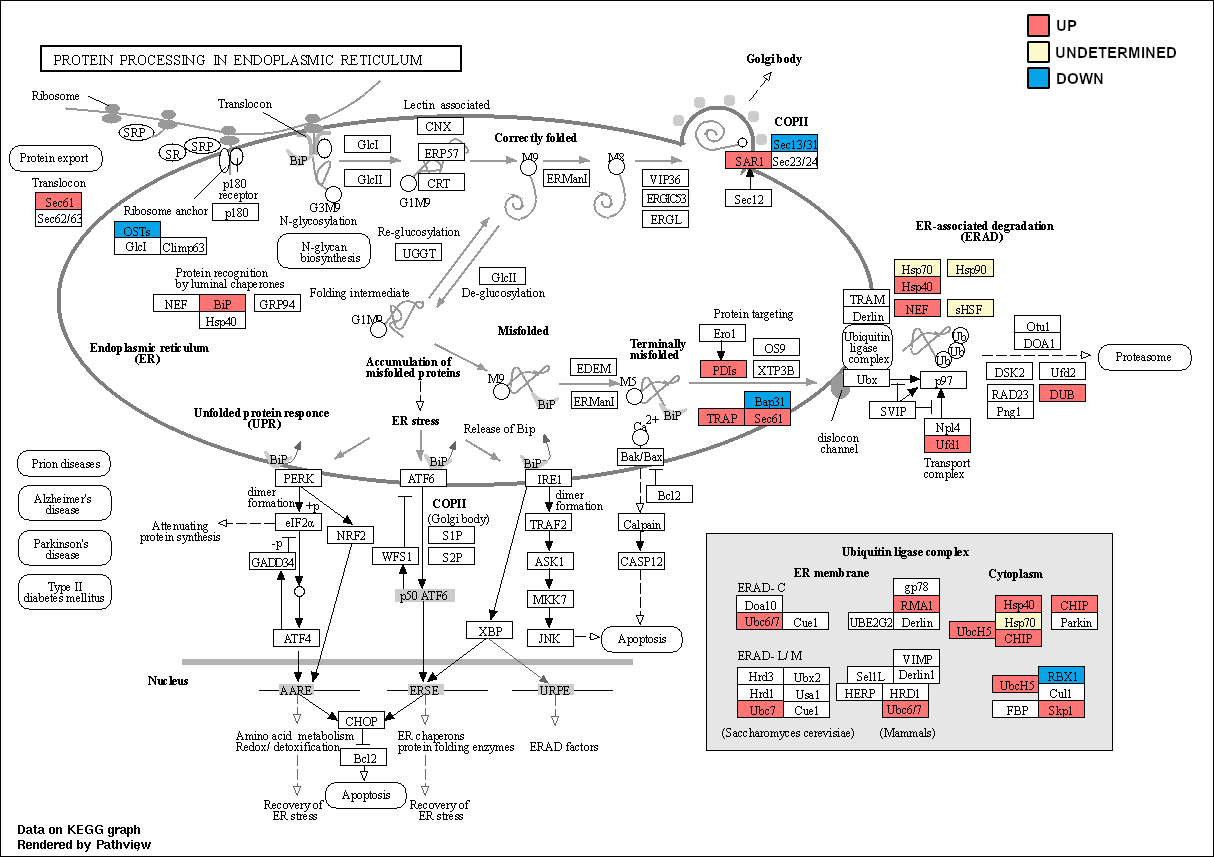

Supplement: Supplementary file 2 [file Data_Sheet_2.ZIP › Supplementary_Figure_4/Supplementary_Figure_4.112.png]

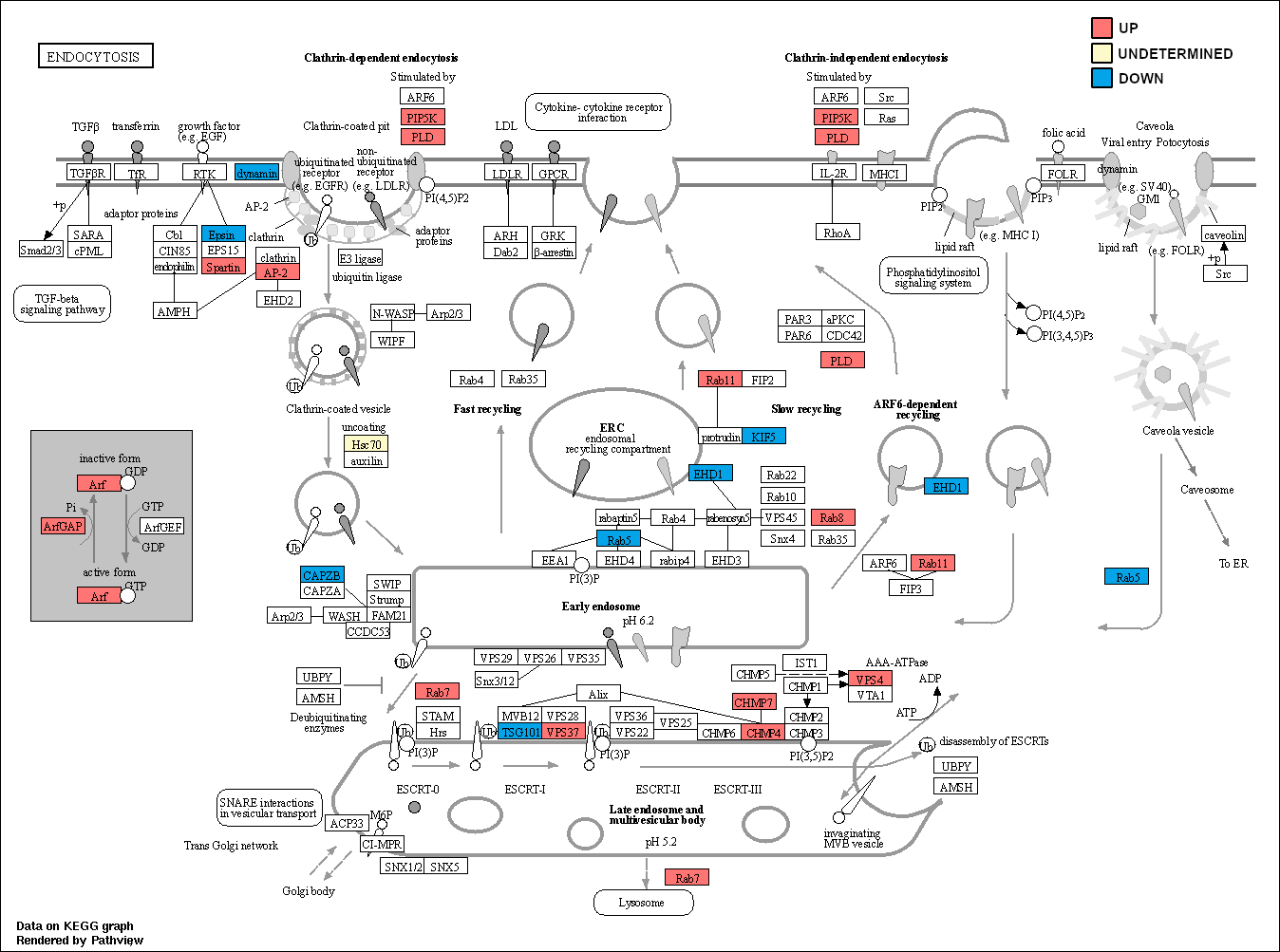

Supplement: Supplementary file 2 [file Data_Sheet_2.ZIP › Supplementary_Figure_4/Supplementary_Figure_4.113.png]

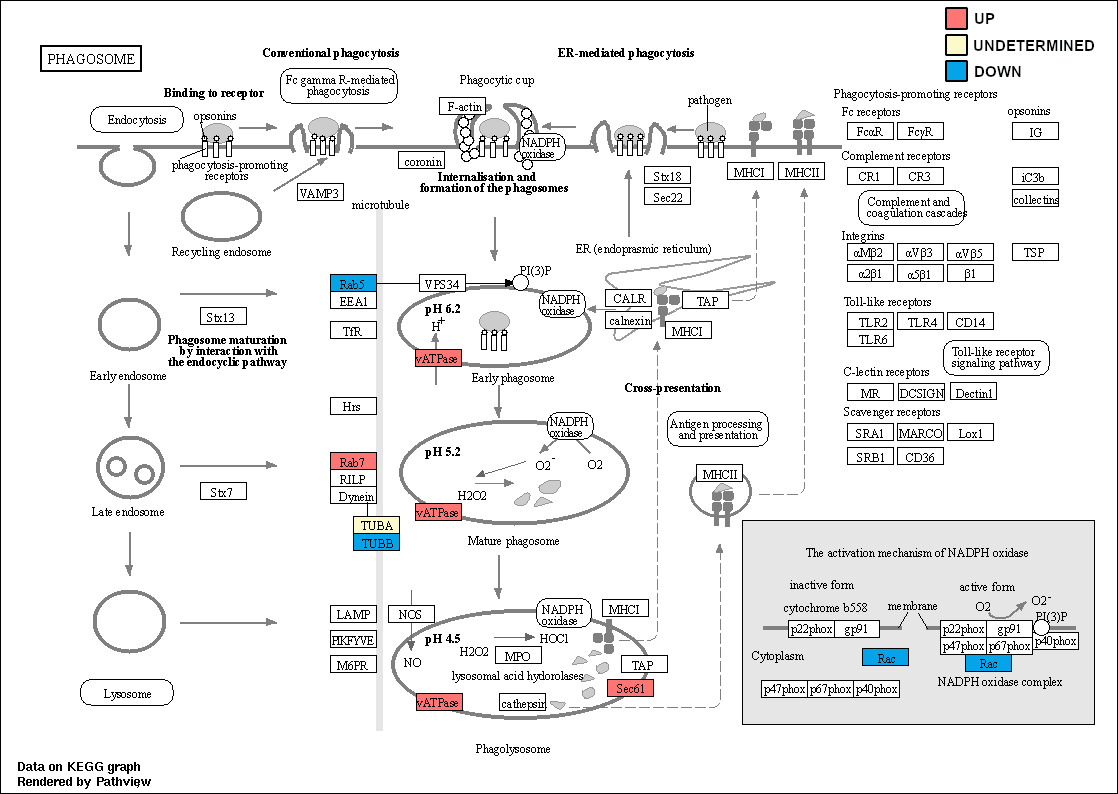

Supplement: Supplementary file 2 [file Data_Sheet_2.ZIP › Supplementary_Figure_4/Supplementary_Figure_4.114.png]

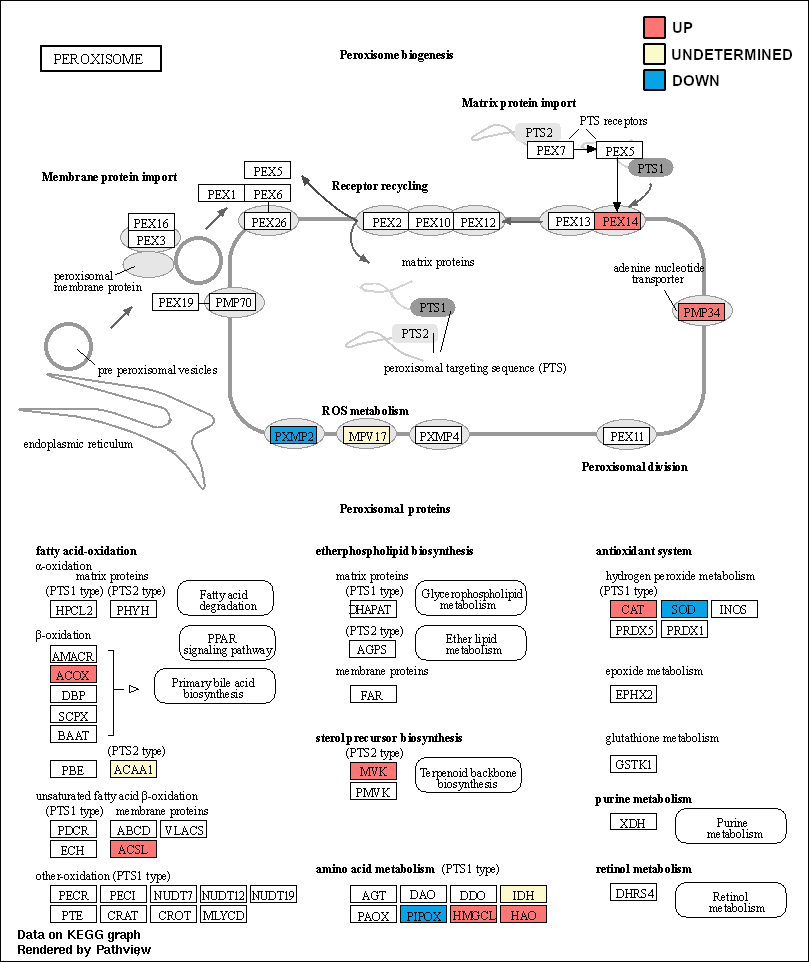

Supplement: Supplementary file 2 [file Data_Sheet_2.ZIP › Supplementary_Figure_4/Supplementary_Figure_4.115.png]

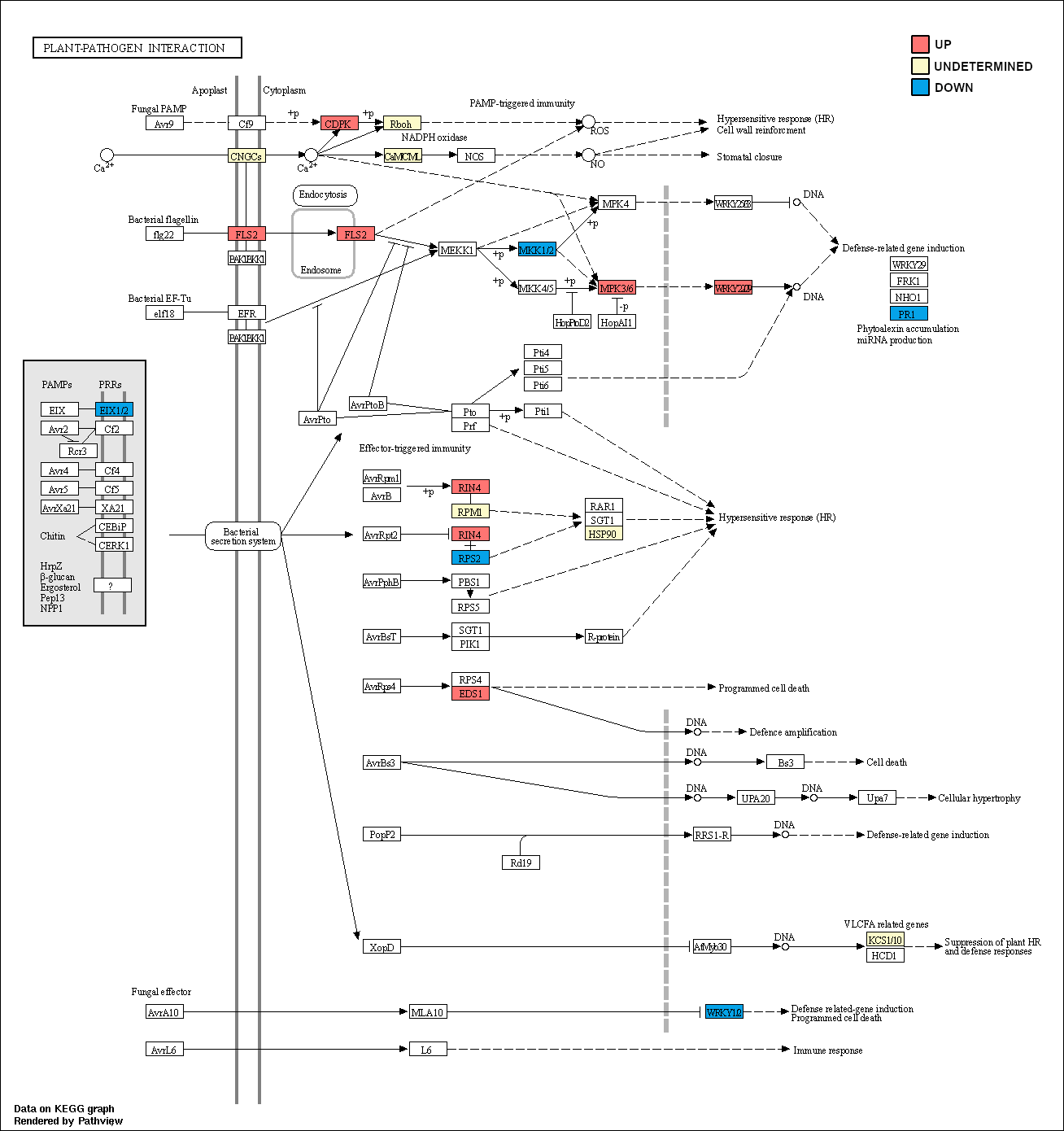

Supplement: Supplementary file 2 [file Data_Sheet_2.ZIP › Supplementary_Figure_4/Supplementary_Figure_4.116.png]

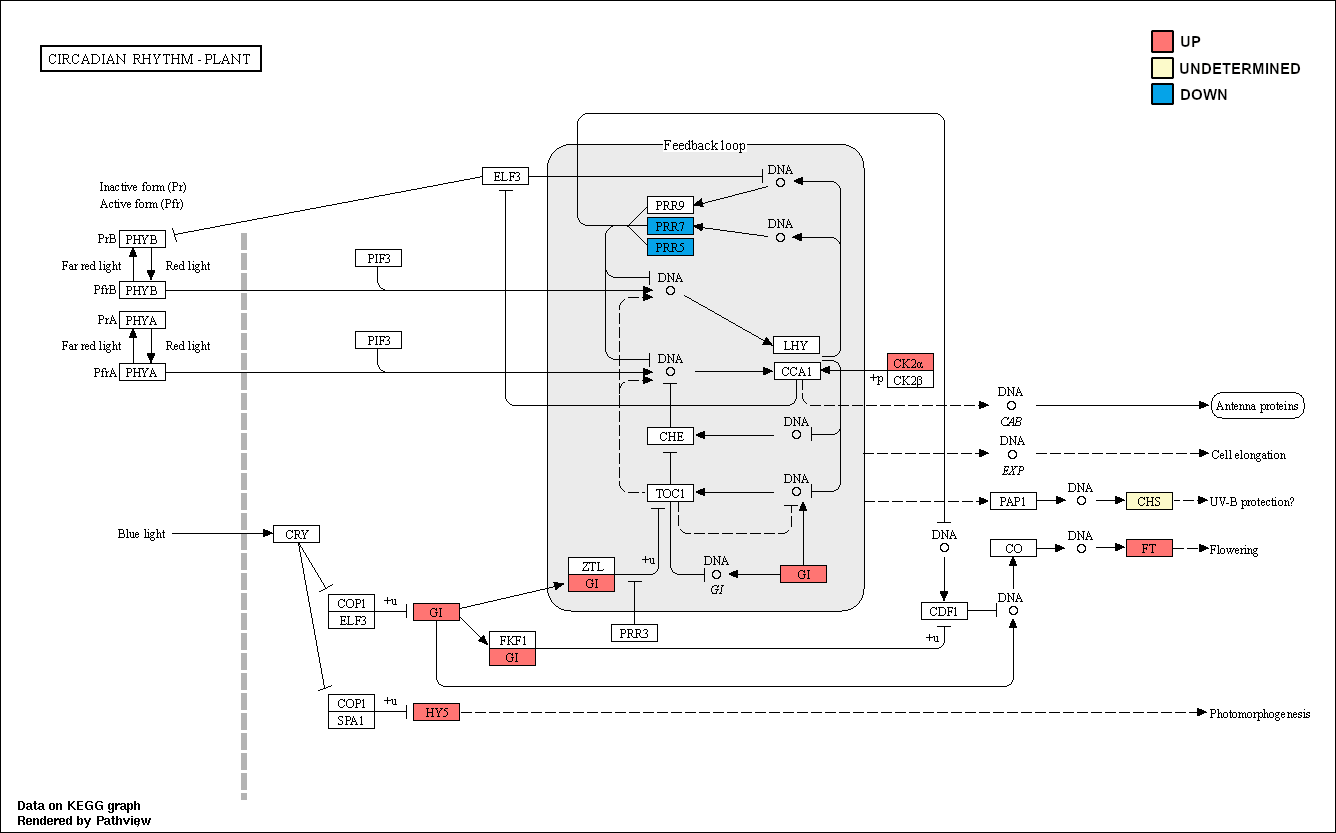

Supplement: Supplementary file 2 [file Data_Sheet_2.ZIP › Supplementary_Figure_4/Supplementary_Figure_4.117.png]

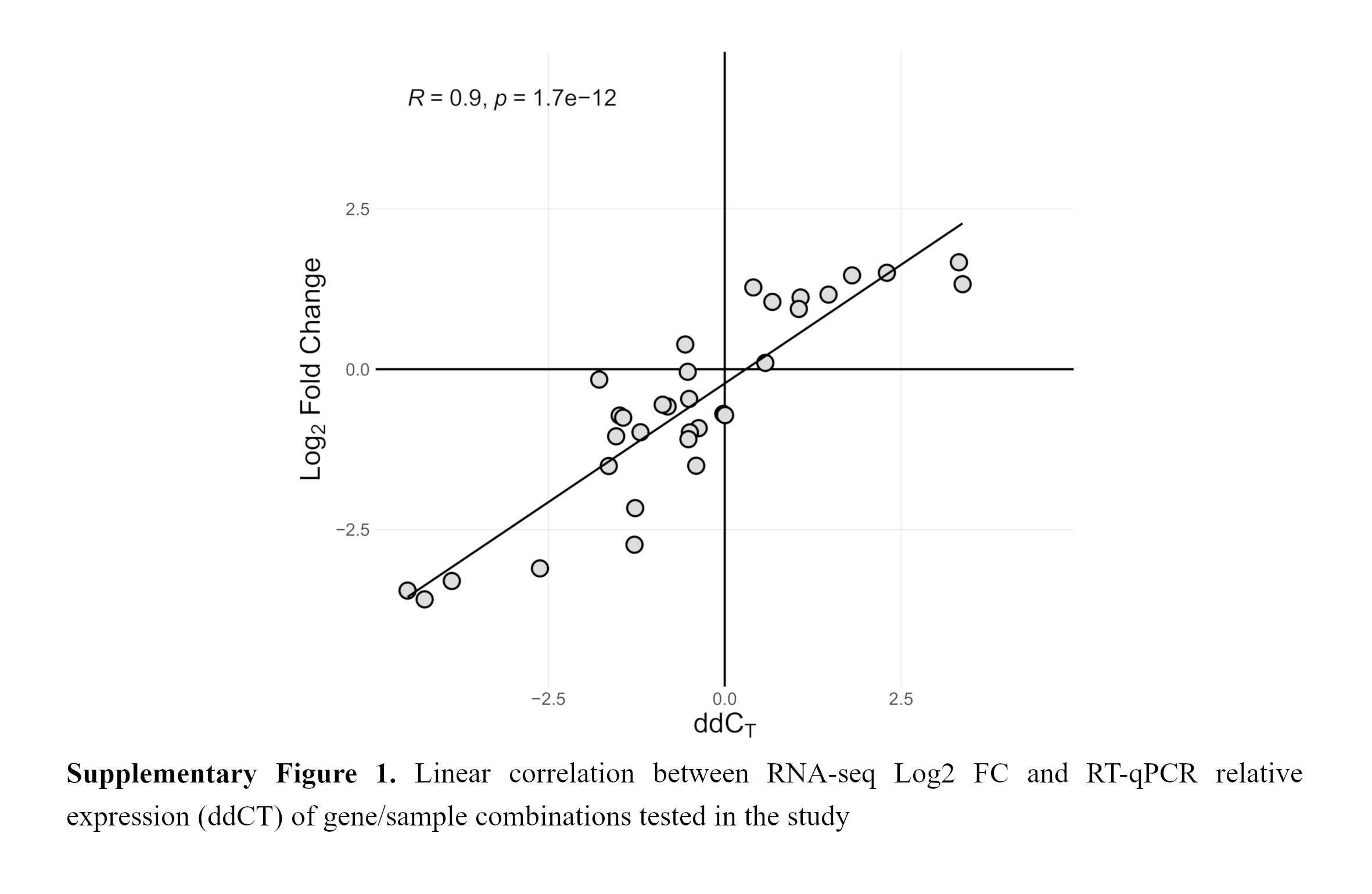

Supplement: Supplementary file 4 [file Image_1.JPEG]

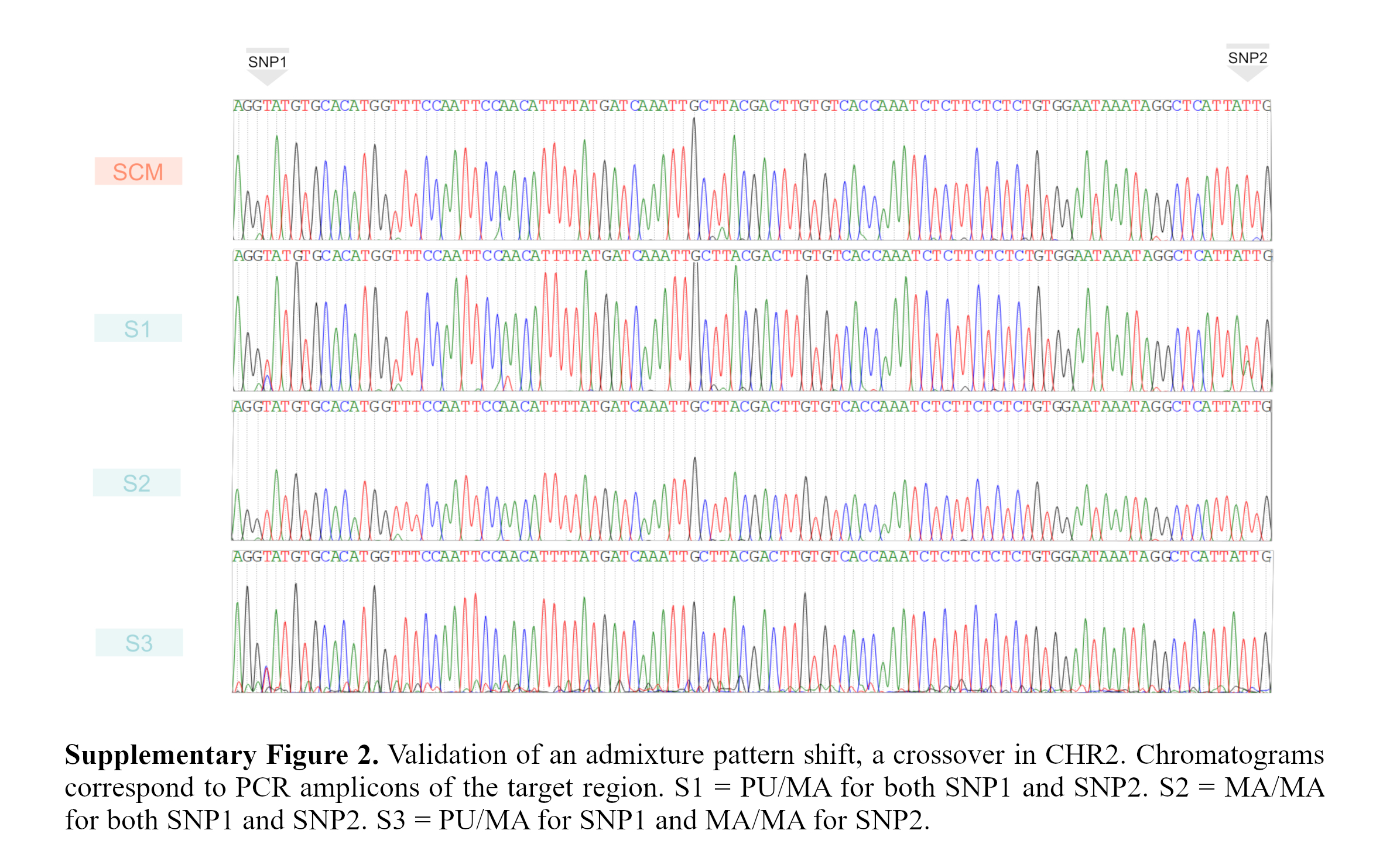

Supplement: Supplementary file 5 [file Image_2.JPEG]

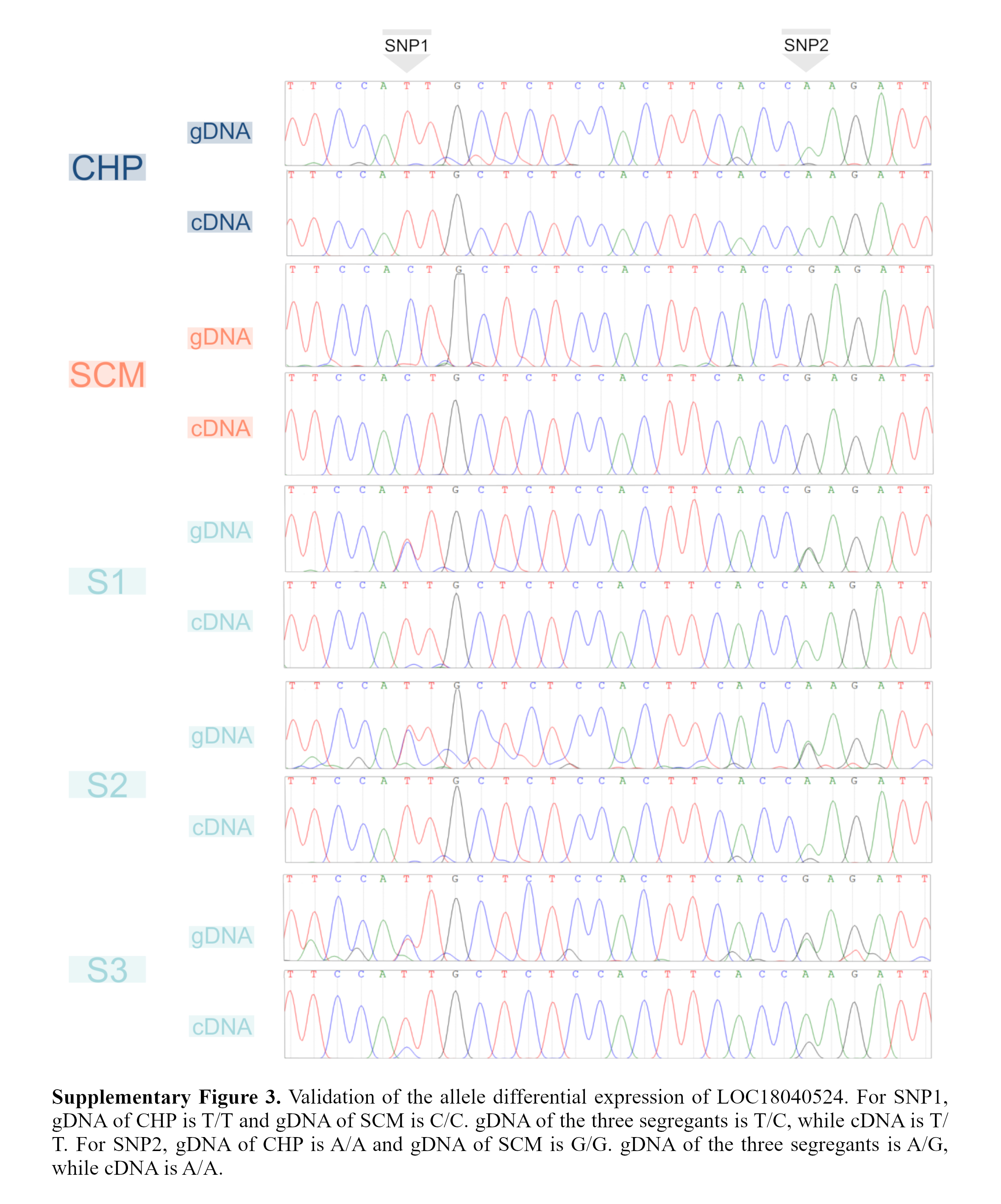

Supplement: Supplementary file 6 [file Image_3.JPEG]

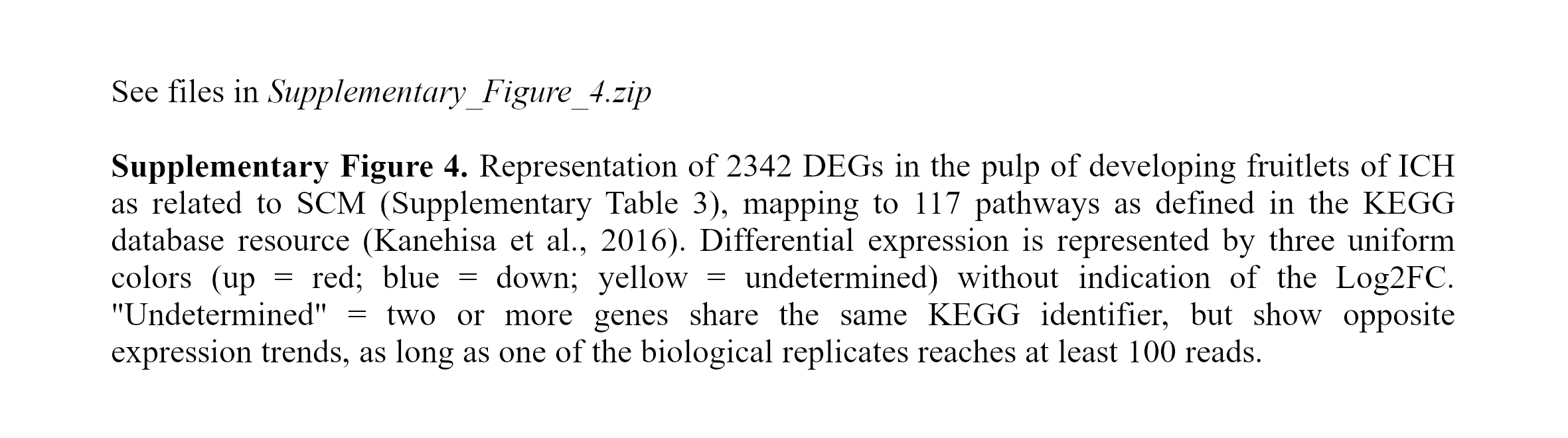

Supplement: Supplementary file 7 [file Image_4.JPEG]

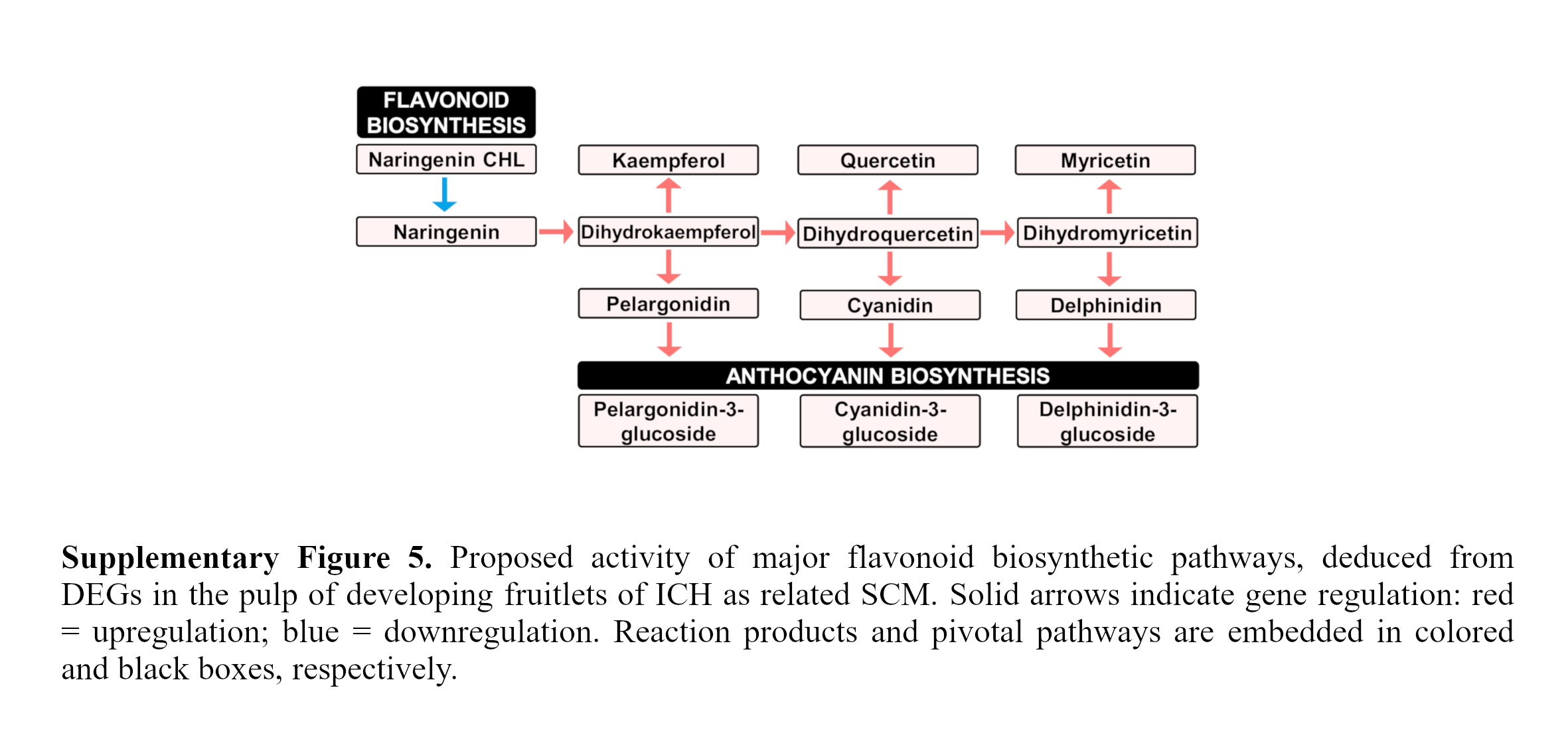

Supplement: Supplementary file 8 [file Image_5.JPEG]

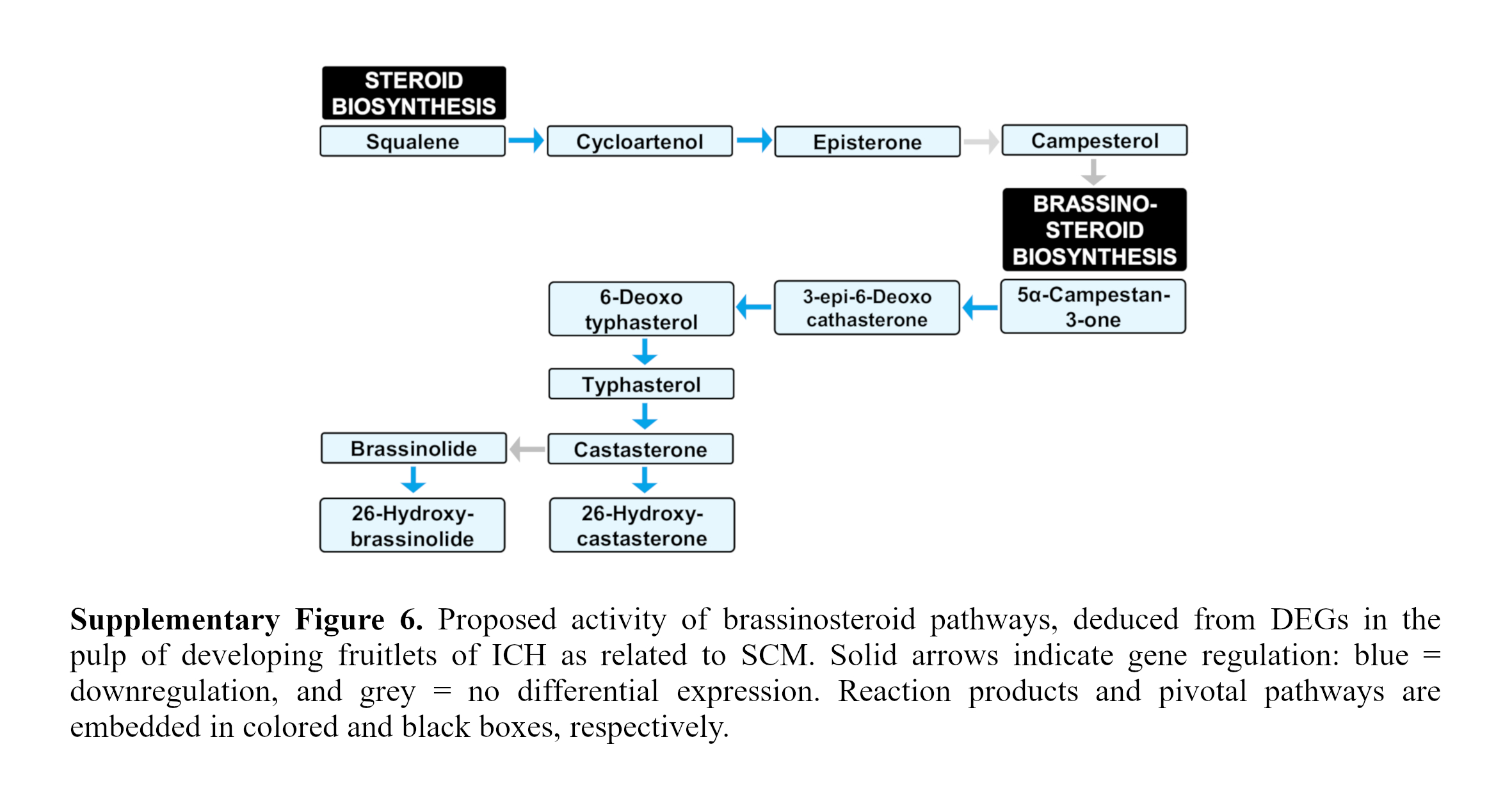

Supplement: Supplementary file 9 [file Image_6.JPEG]

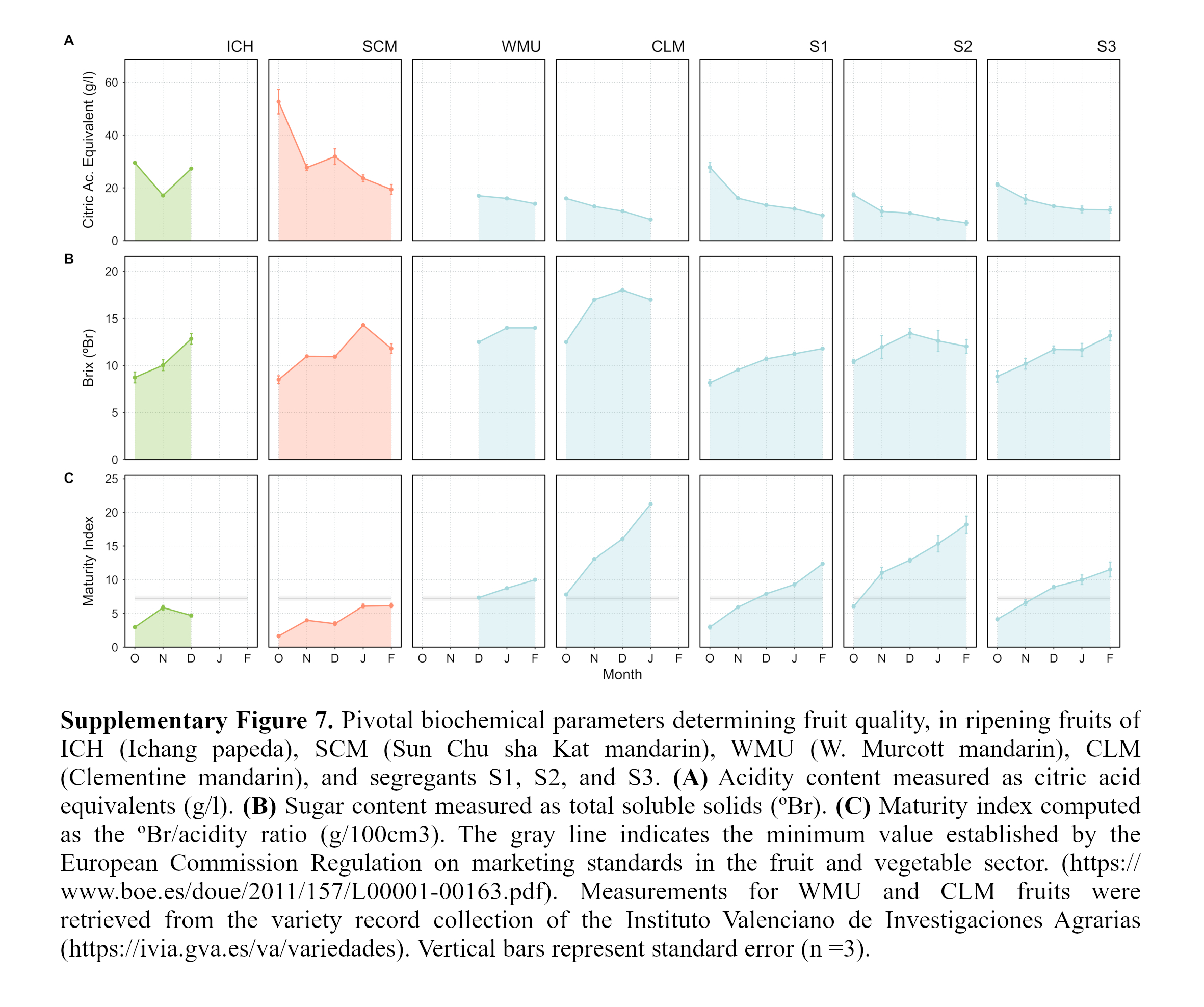

Supplement: Supplementary file 10 [file Image_7.JPEG]

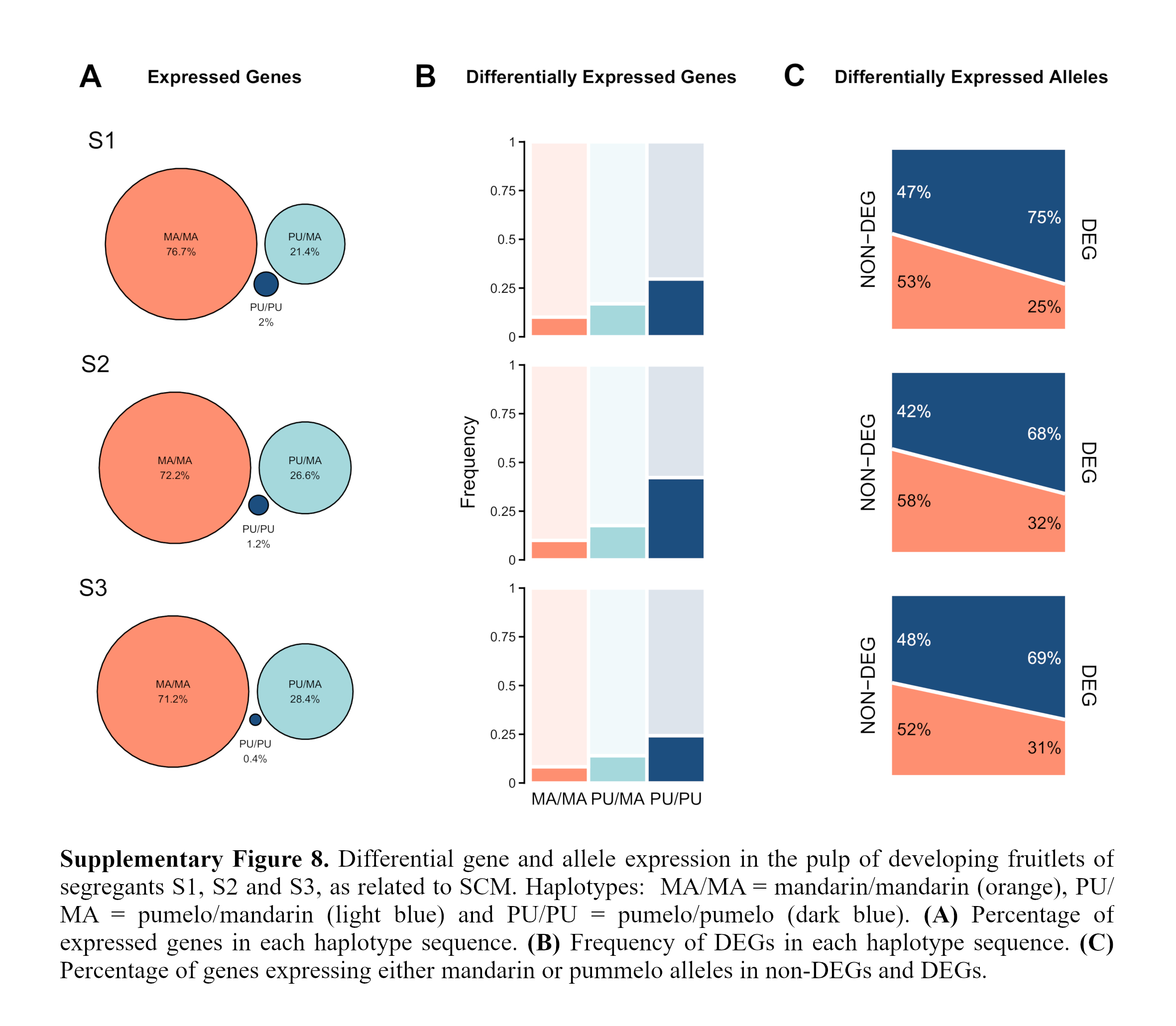

Supplement: Supplementary file 11 [file Image_8.JPEG]
