# Supplementary figures and images for: Transcriptome analysis of the pulp of citrus fruitlets suggests that domestication enhanced growth processes and reduced chemical defenses increasing palatability (part 1 of 2)
Source: Front Plant Sci. 2022 Sep 2;13:982683. doi: 10.3389/fpls.2022.982683 (PMC9478336; doi:10.3389/fpls.2022.982683)

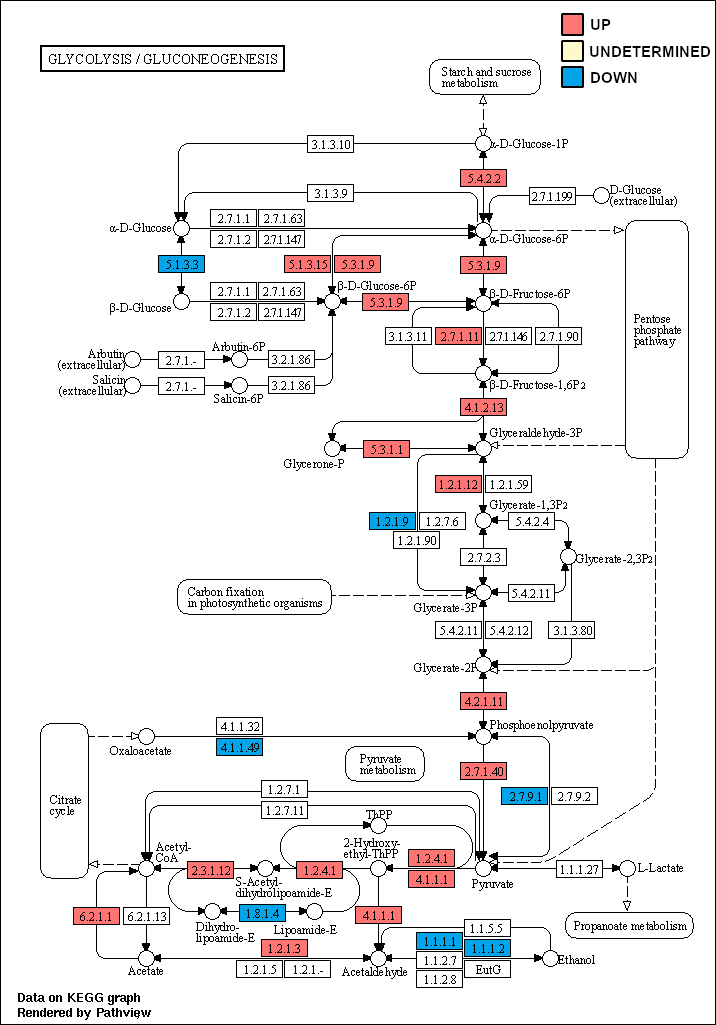

Supplement: Supplementary file 2 [file Data_Sheet_2.ZIP › Supplementary_Figure_4/Supplementary_Figure_4.001.png]

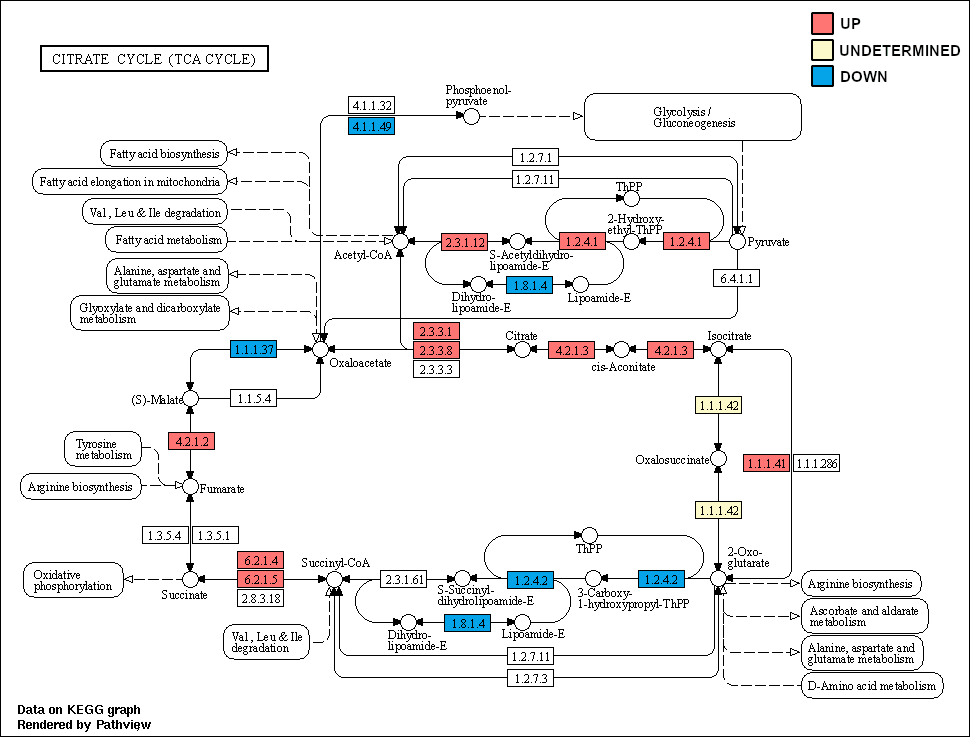

Supplement: Supplementary file 2 [file Data_Sheet_2.ZIP › Supplementary_Figure_4/Supplementary_Figure_4.002.png]

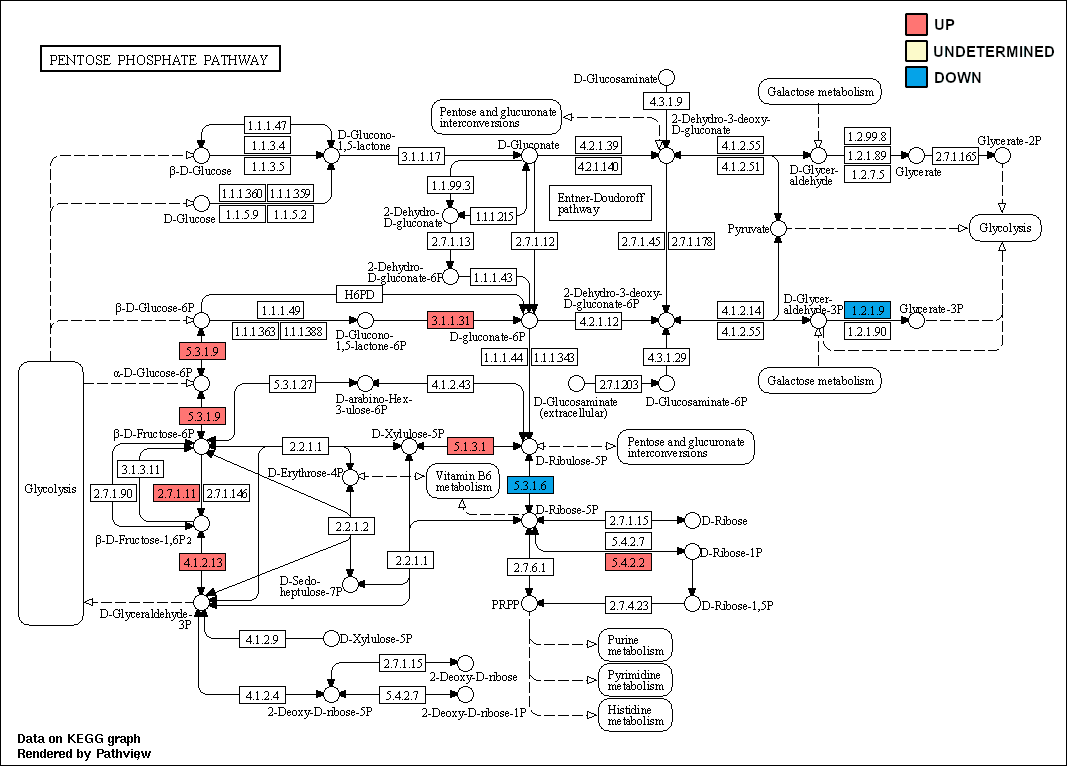

Supplement: Supplementary file 2 [file Data_Sheet_2.ZIP › Supplementary_Figure_4/Supplementary_Figure_4.003.png]

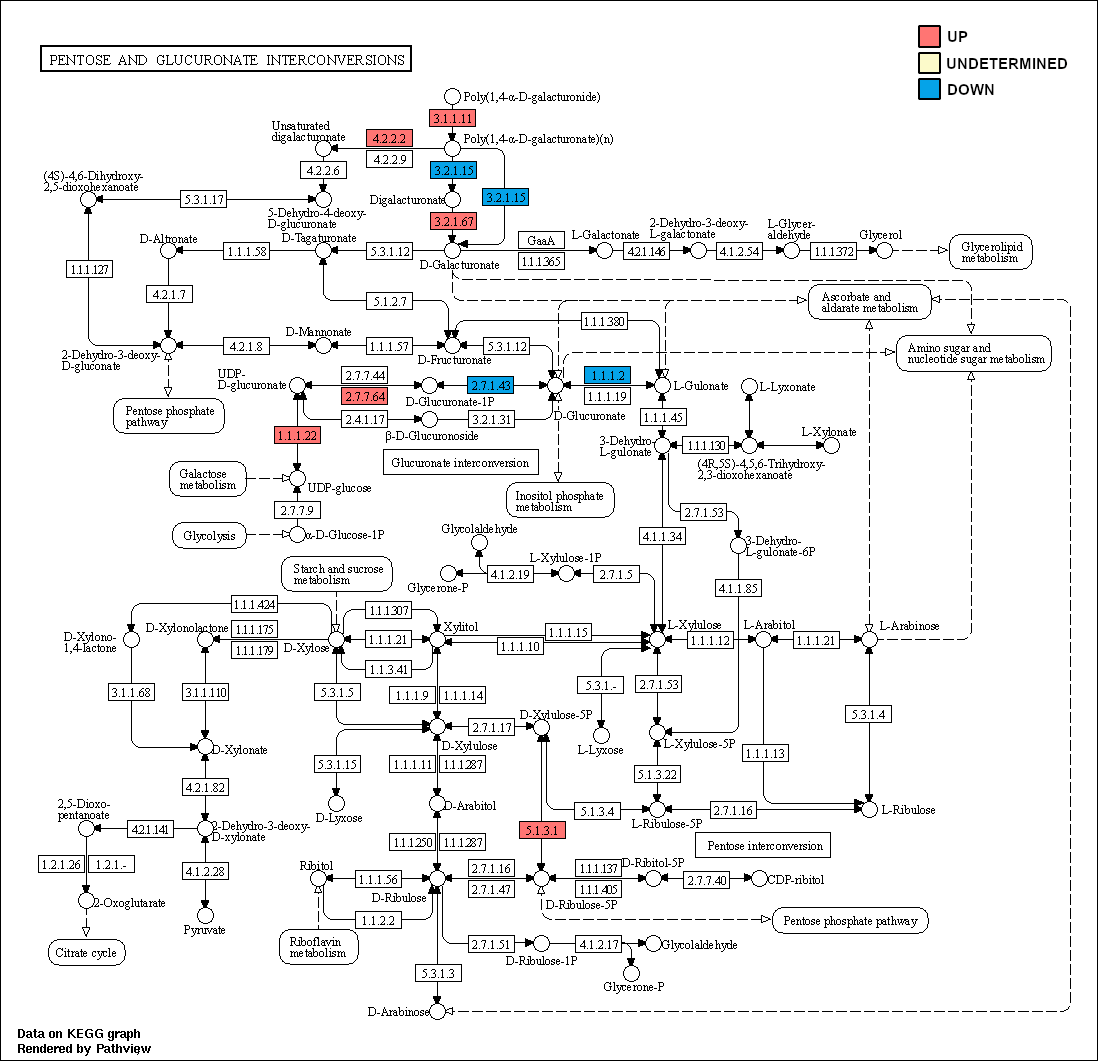

Supplement: Supplementary file 2 [file Data_Sheet_2.ZIP › Supplementary_Figure_4/Supplementary_Figure_4.004.png]

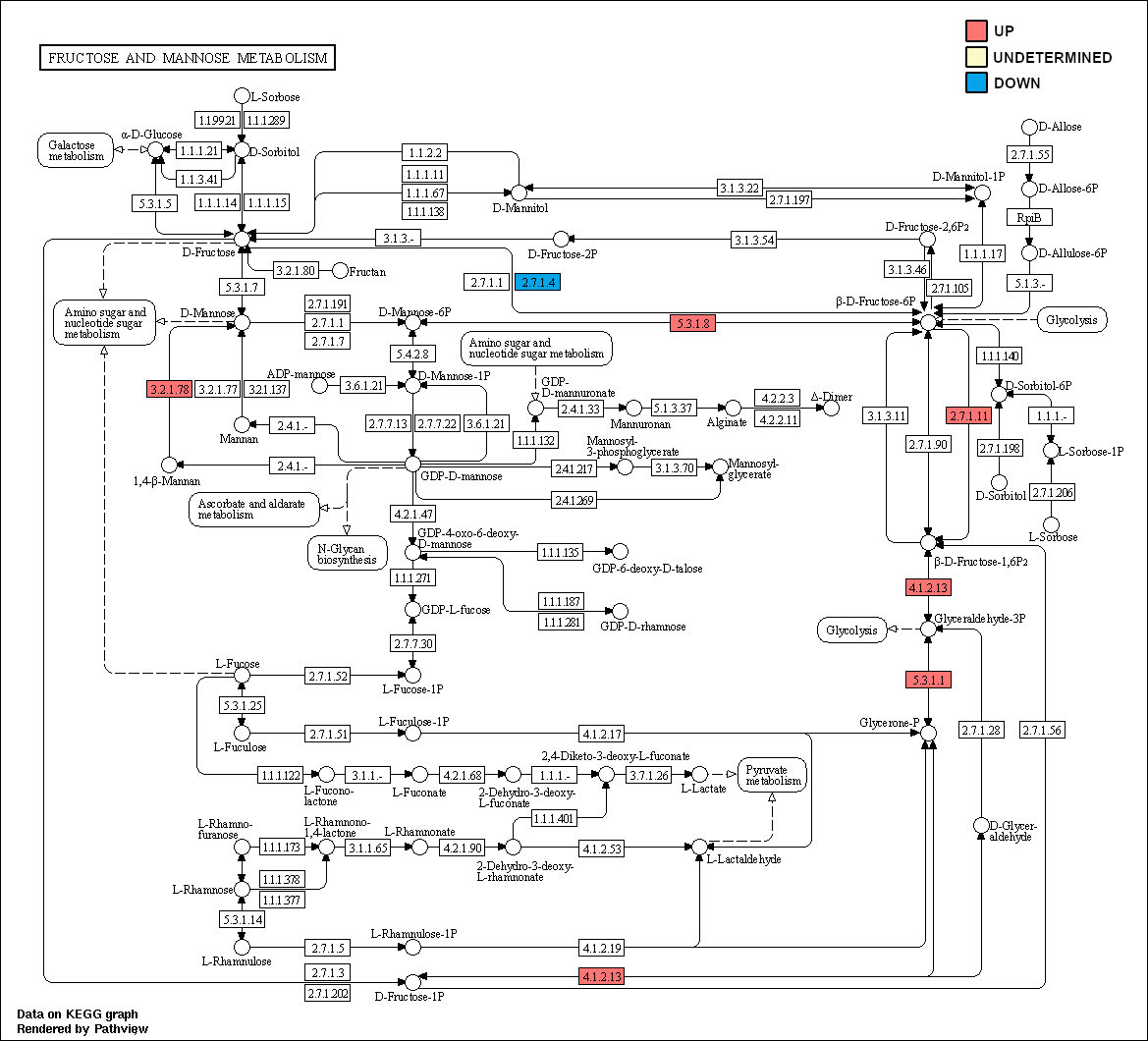

Supplement: Supplementary file 2 [file Data_Sheet_2.ZIP › Supplementary_Figure_4/Supplementary_Figure_4.005.png]

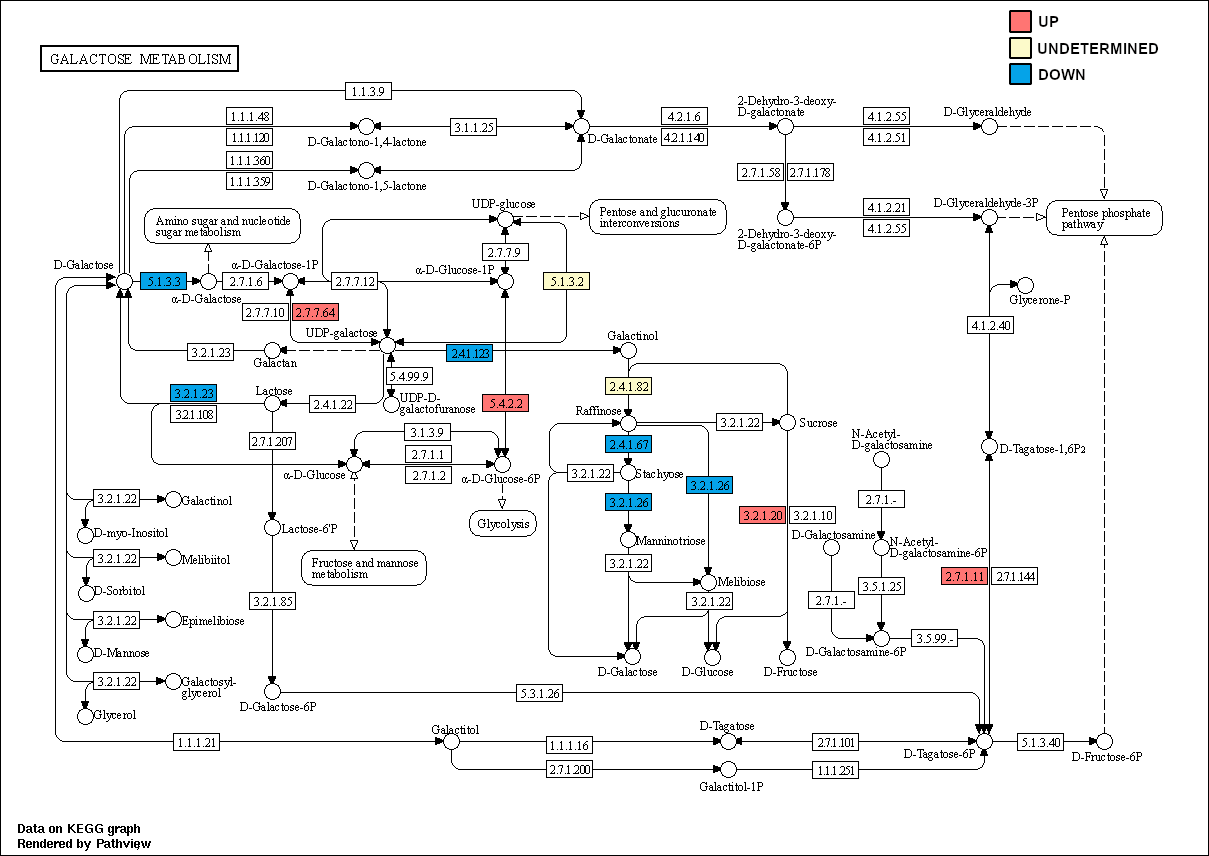

Supplement: Supplementary file 2 [file Data_Sheet_2.ZIP › Supplementary_Figure_4/Supplementary_Figure_4.006.png]

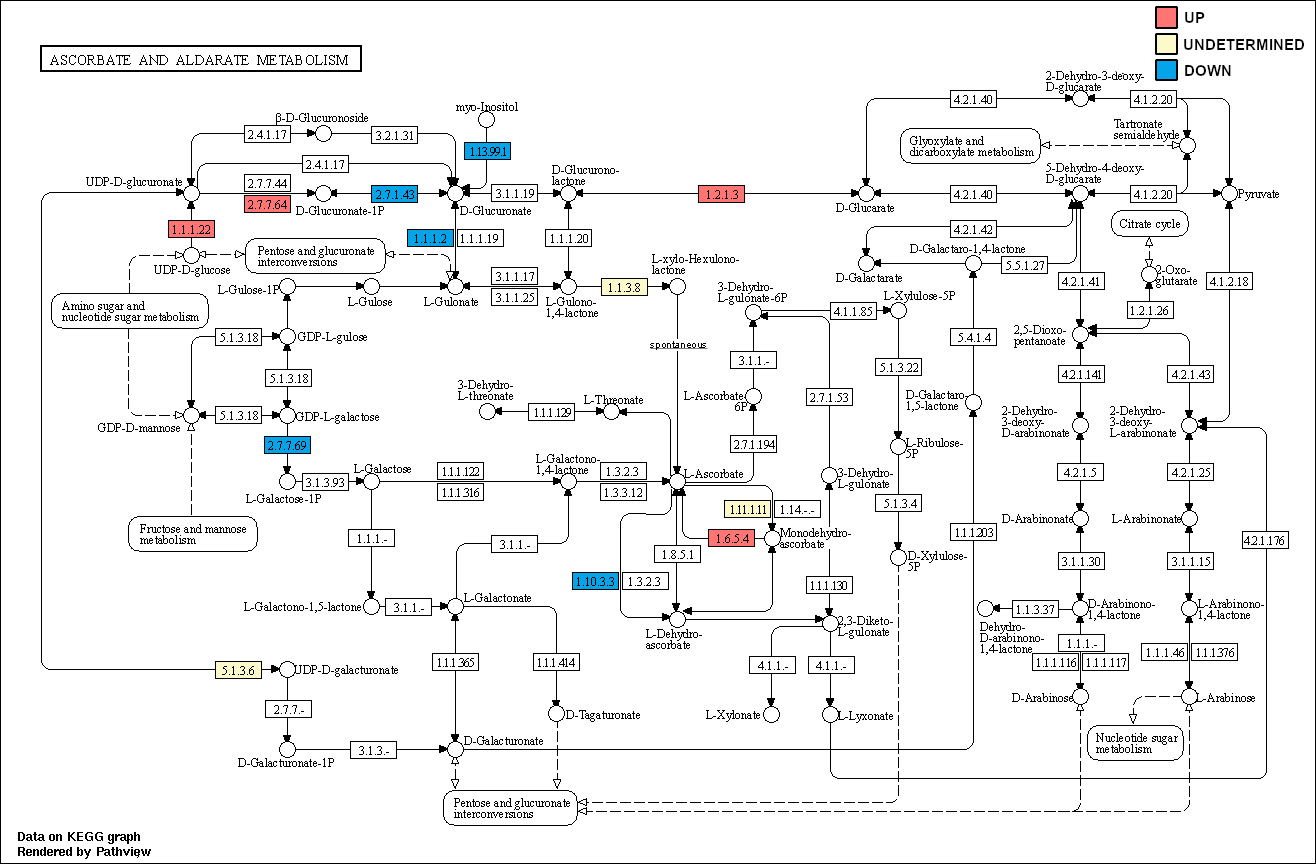

Supplement: Supplementary file 2 [file Data_Sheet_2.ZIP › Supplementary_Figure_4/Supplementary_Figure_4.007.png]

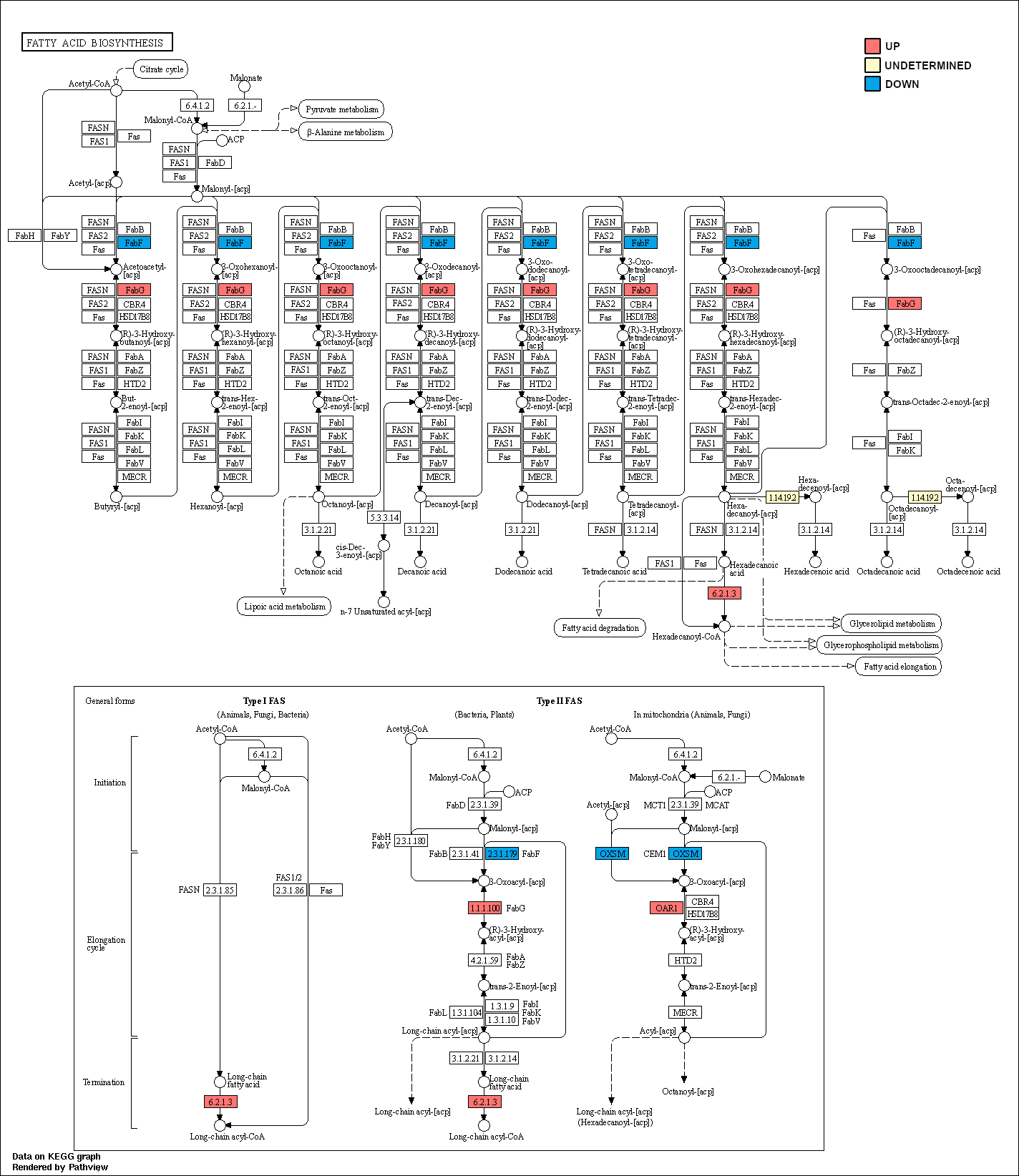

Supplement: Supplementary file 2 [file Data_Sheet_2.ZIP › Supplementary_Figure_4/Supplementary_Figure_4.008.png]

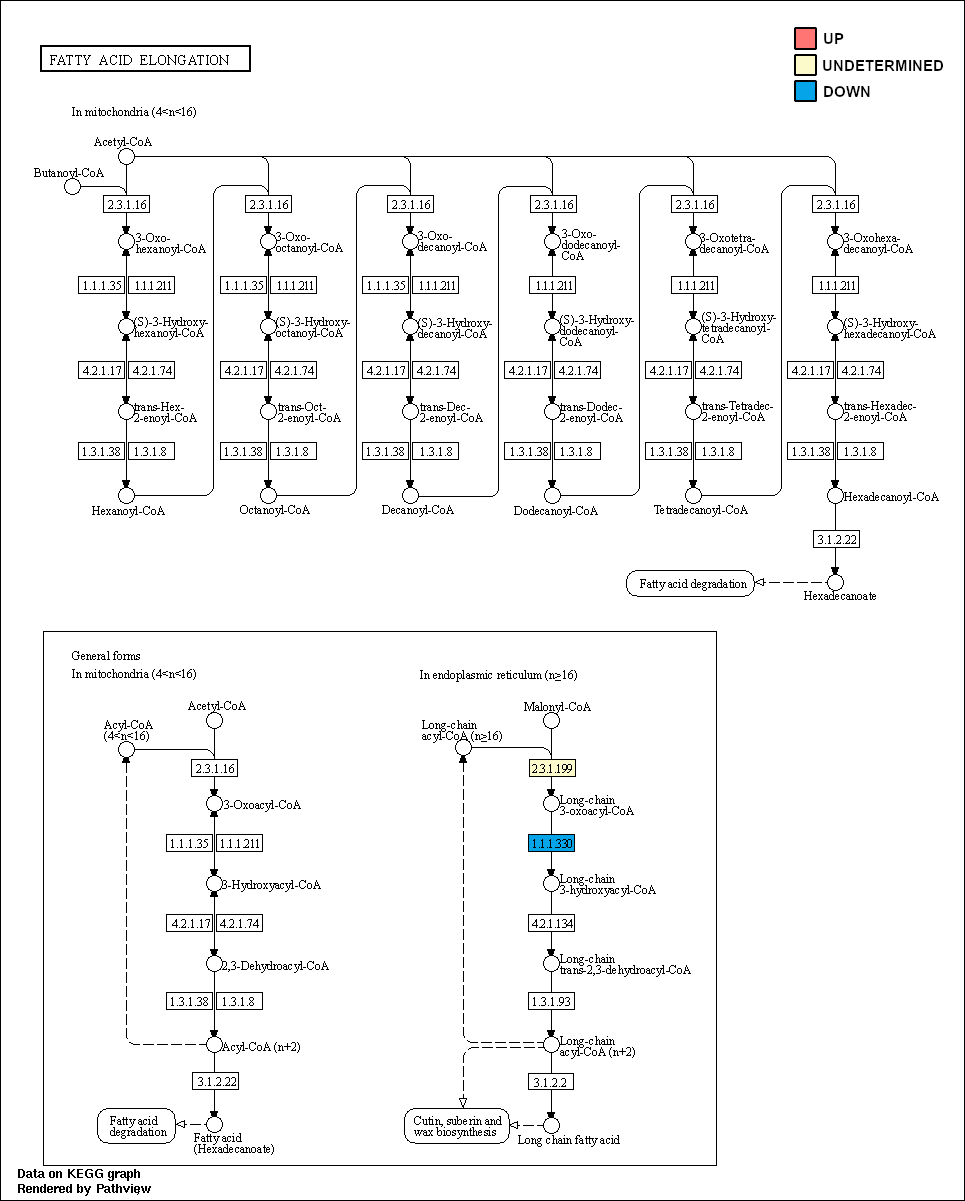

Supplement: Supplementary file 2 [file Data_Sheet_2.ZIP › Supplementary_Figure_4/Supplementary_Figure_4.009.png]

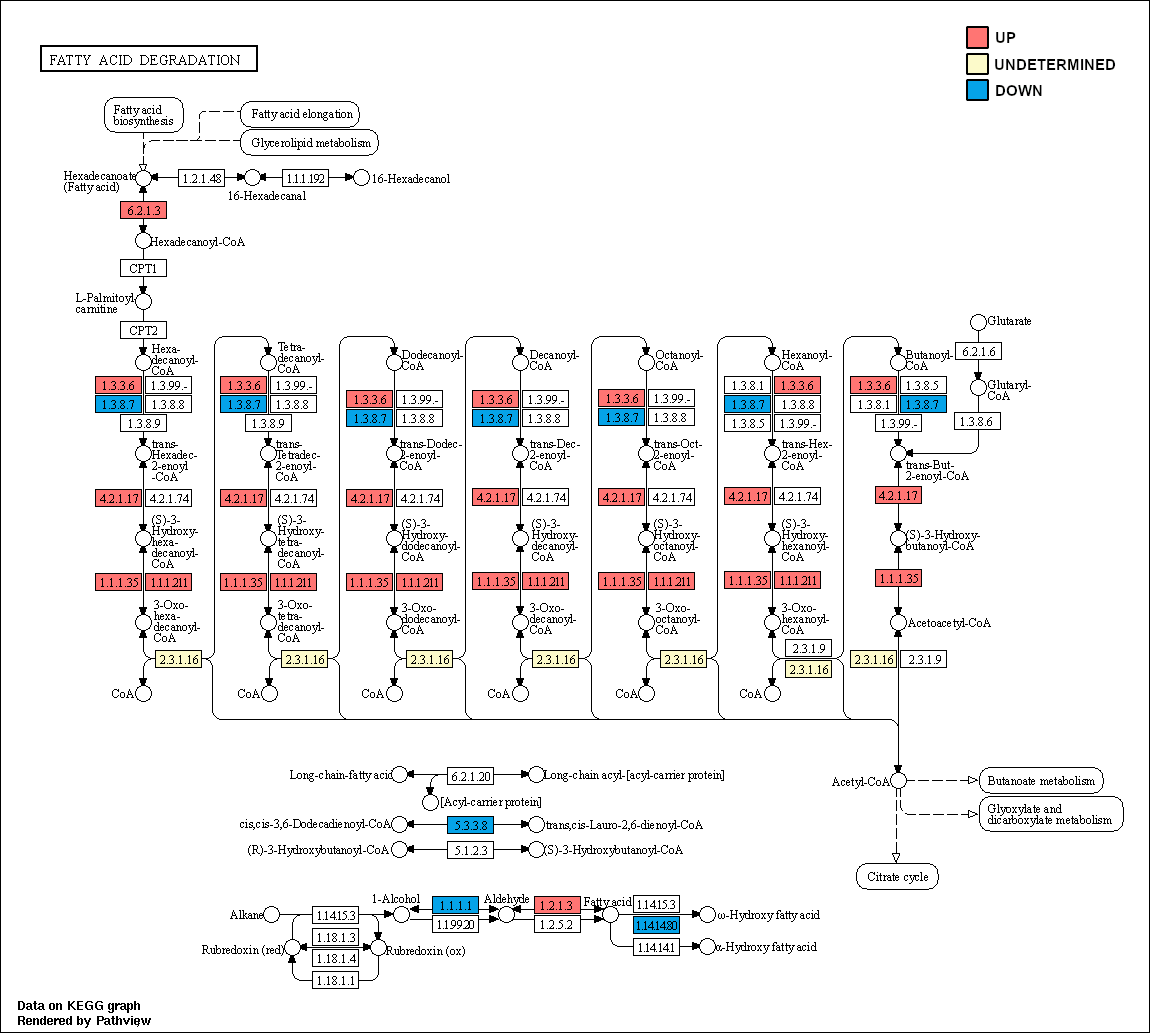

Supplement: Supplementary file 2 [file Data_Sheet_2.ZIP › Supplementary_Figure_4/Supplementary_Figure_4.010.png]

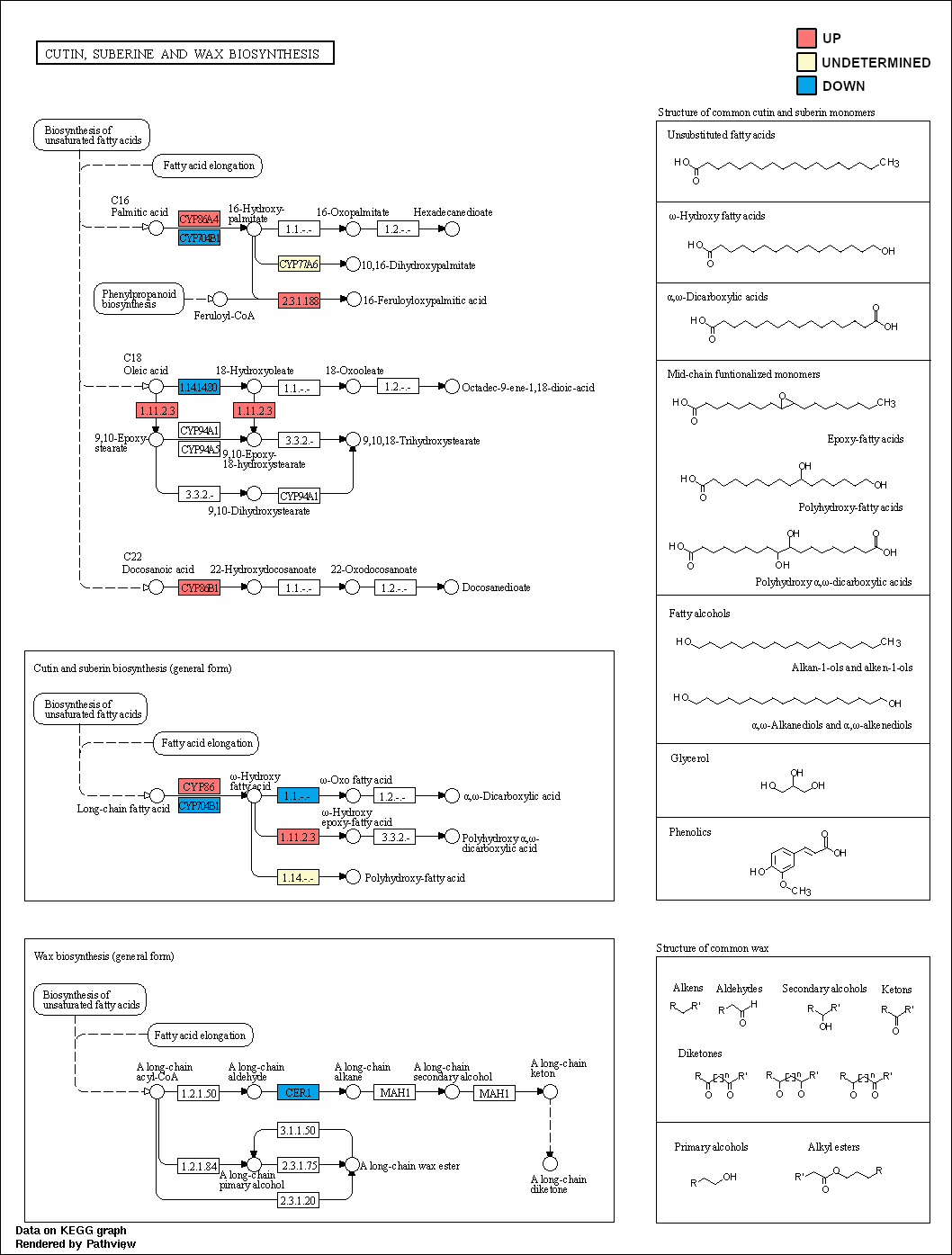

Supplement: Supplementary file 2 [file Data_Sheet_2.ZIP › Supplementary_Figure_4/Supplementary_Figure_4.011.png]

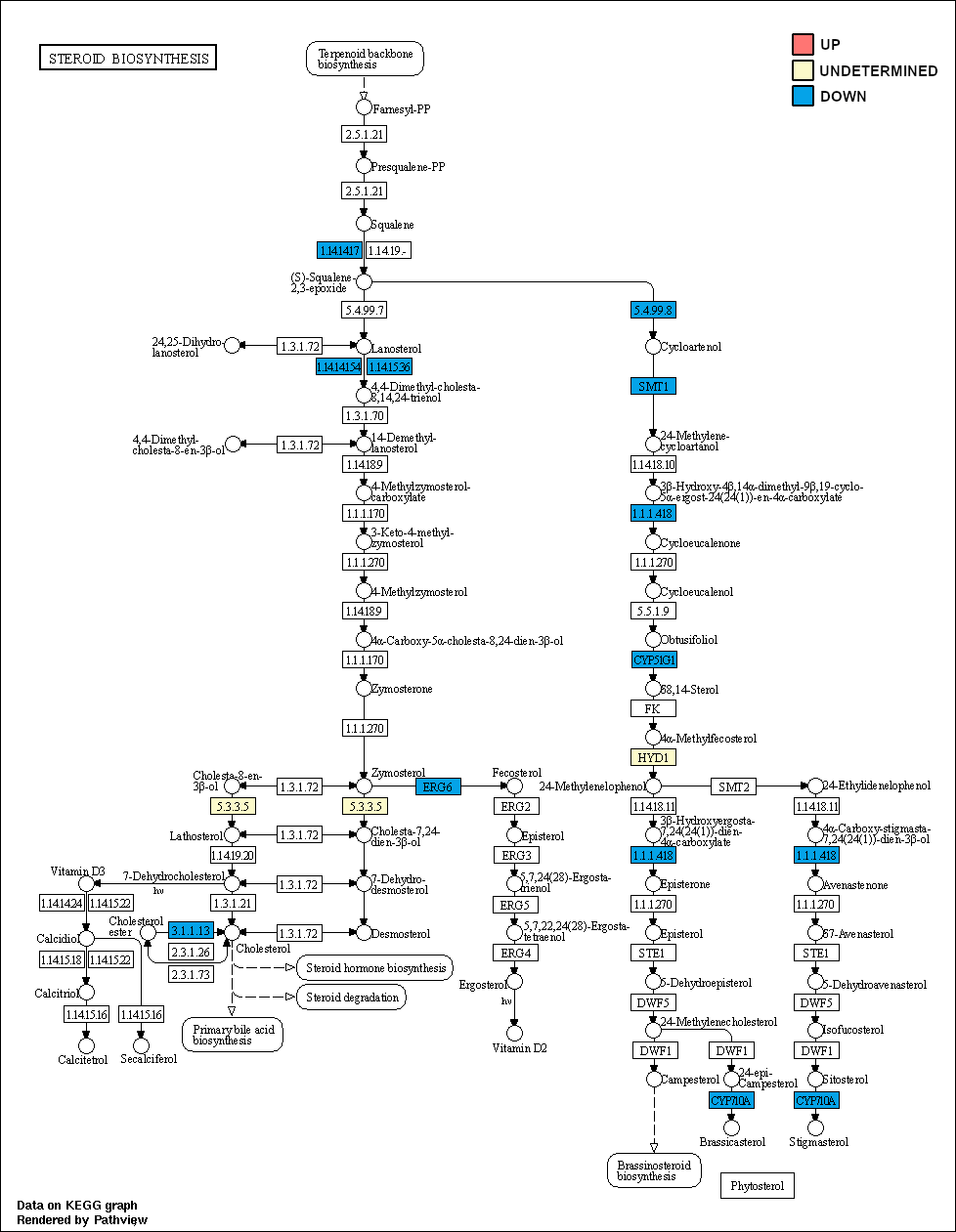

Supplement: Supplementary file 2 [file Data_Sheet_2.ZIP › Supplementary_Figure_4/Supplementary_Figure_4.012.png]

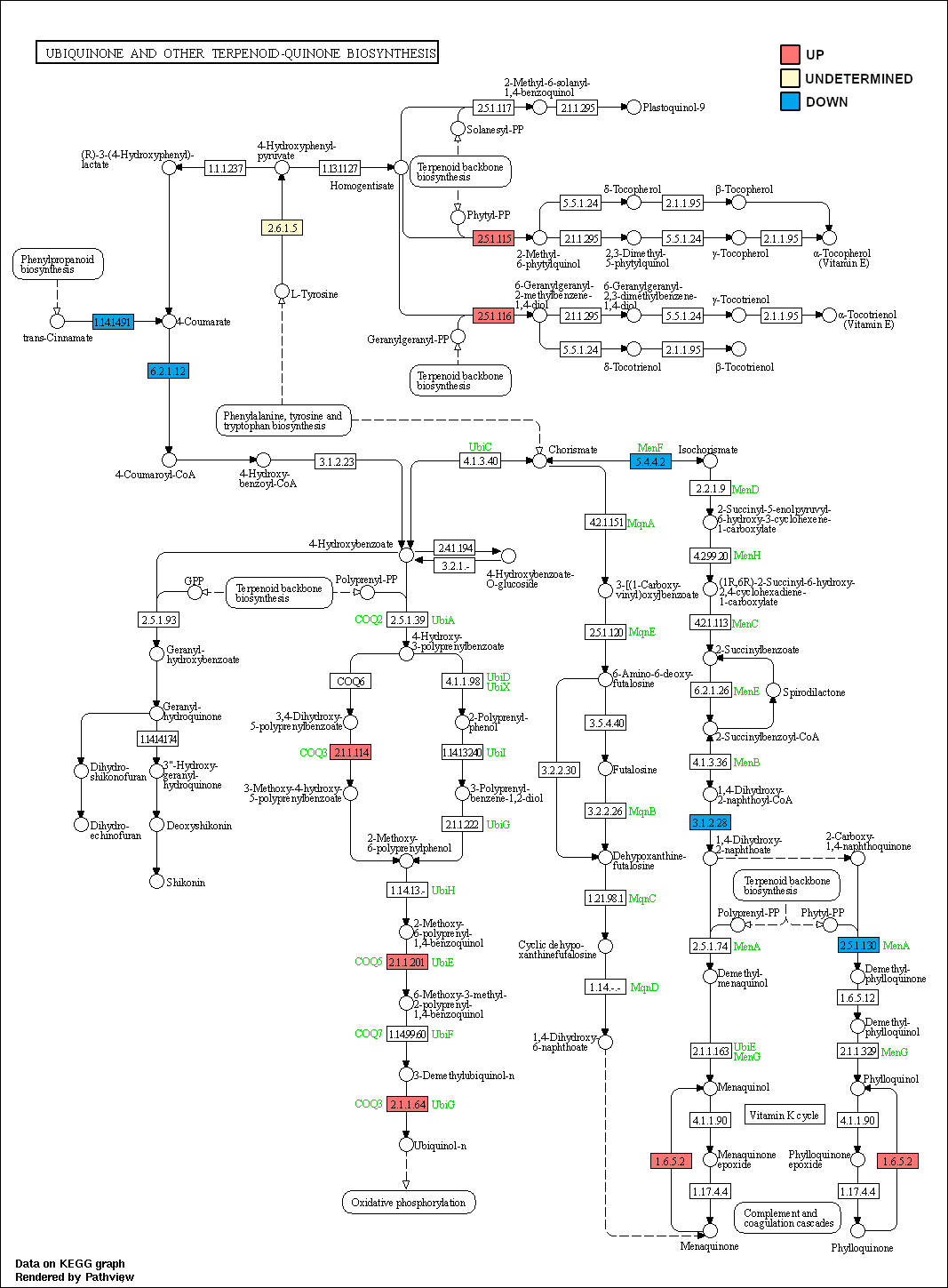

Supplement: Supplementary file 2 [file Data_Sheet_2.ZIP › Supplementary_Figure_4/Supplementary_Figure_4.013.png]

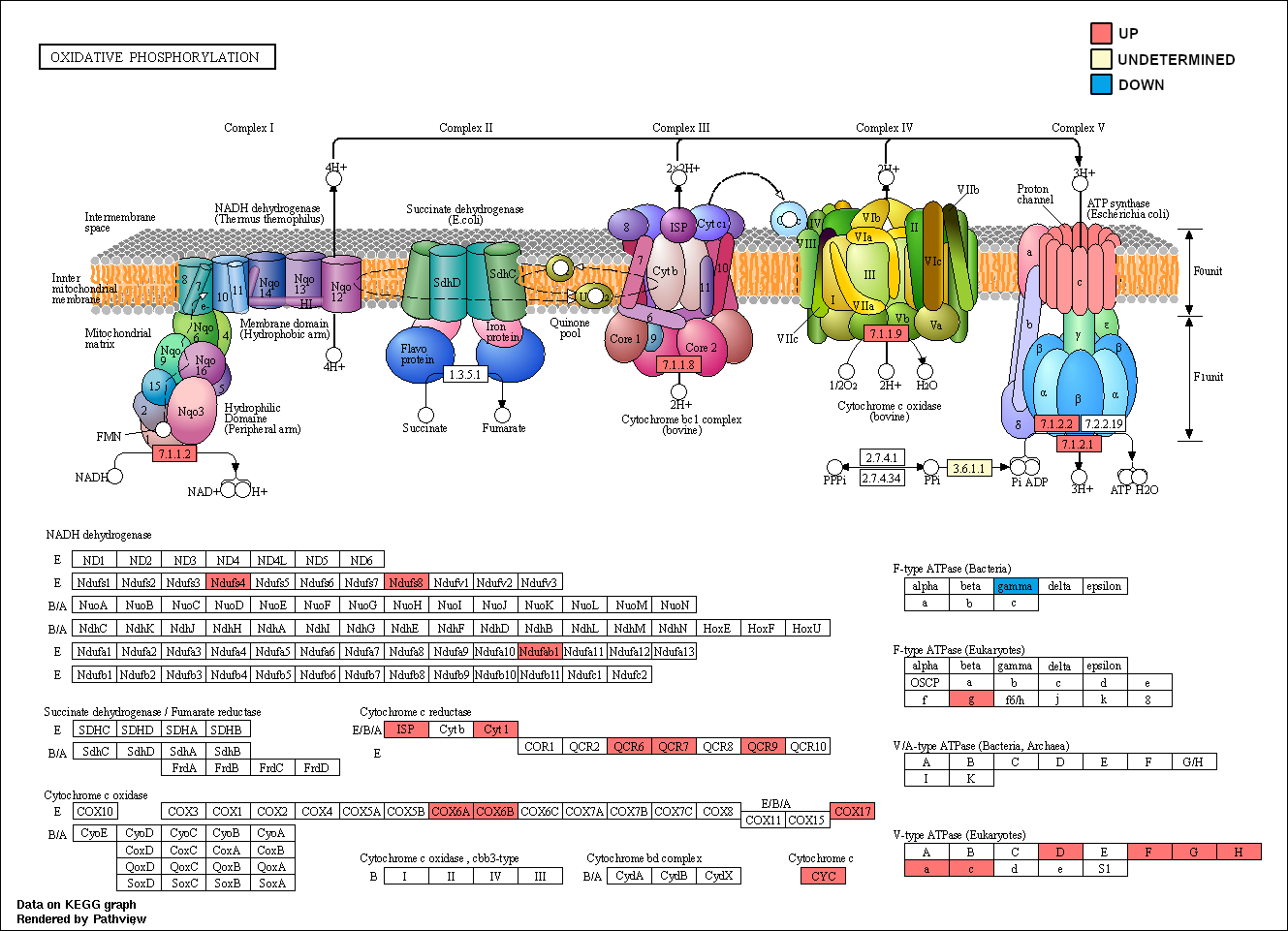

Supplement: Supplementary file 2 [file Data_Sheet_2.ZIP › Supplementary_Figure_4/Supplementary_Figure_4.014.png]

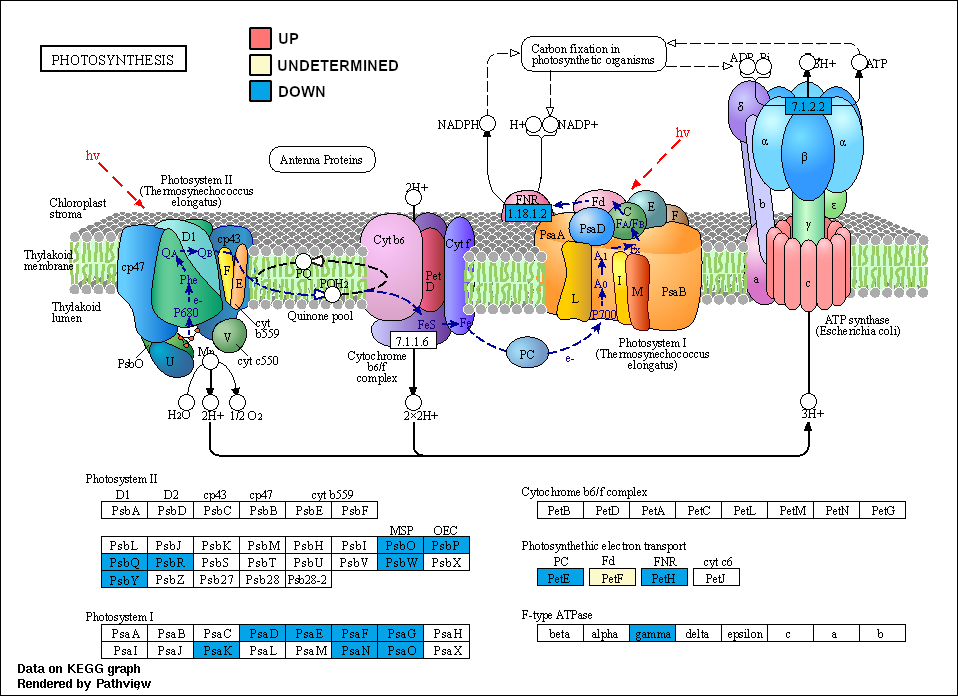

Supplement: Supplementary file 2 [file Data_Sheet_2.ZIP › Supplementary_Figure_4/Supplementary_Figure_4.015.png]

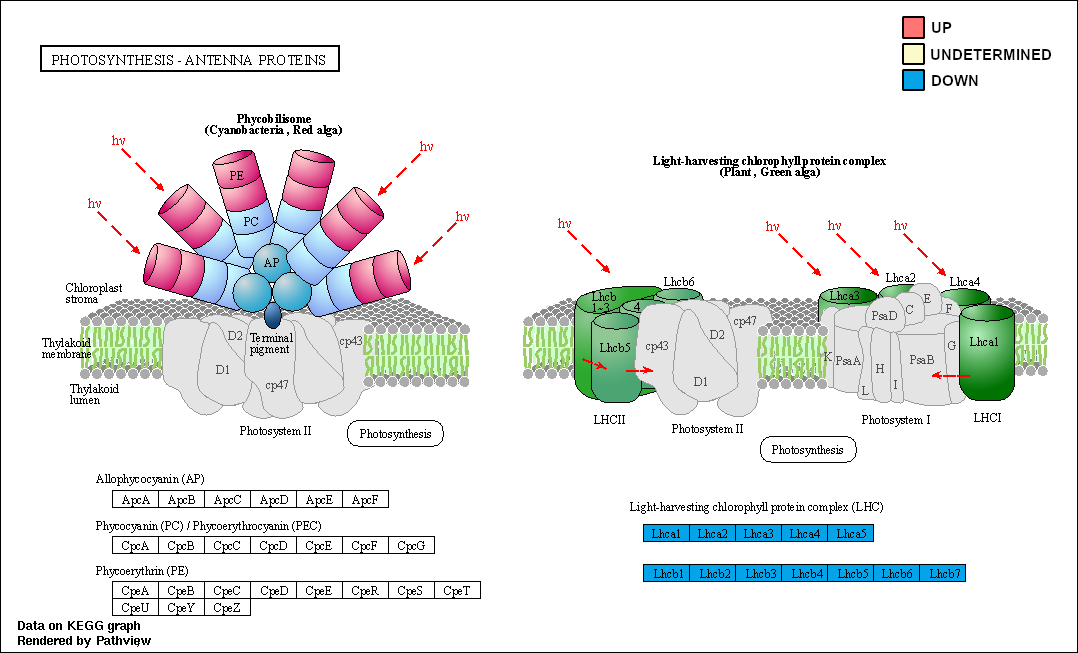

Supplement: Supplementary file 2 [file Data_Sheet_2.ZIP › Supplementary_Figure_4/Supplementary_Figure_4.016.png]

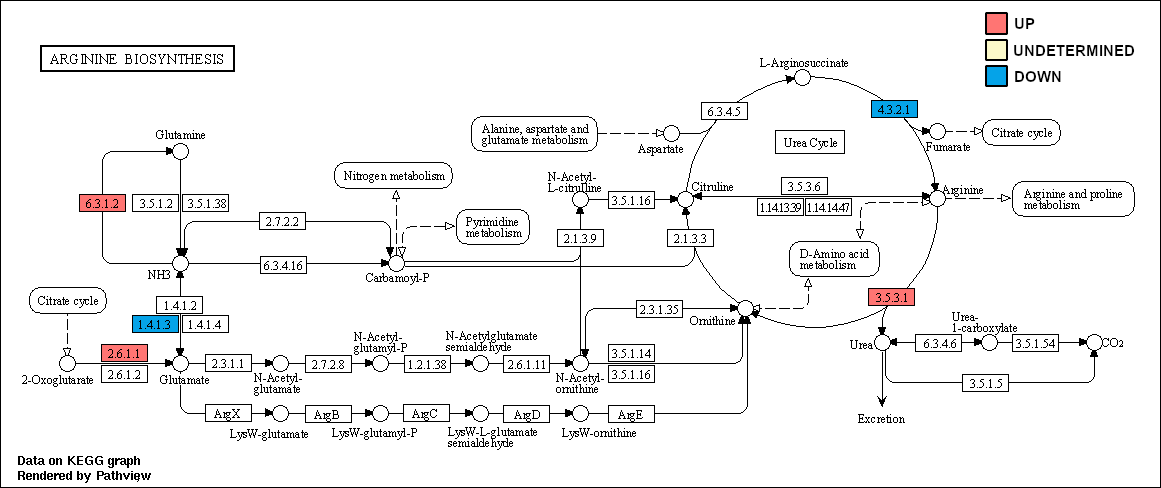

Supplement: Supplementary file 2 [file Data_Sheet_2.ZIP › Supplementary_Figure_4/Supplementary_Figure_4.017.png]

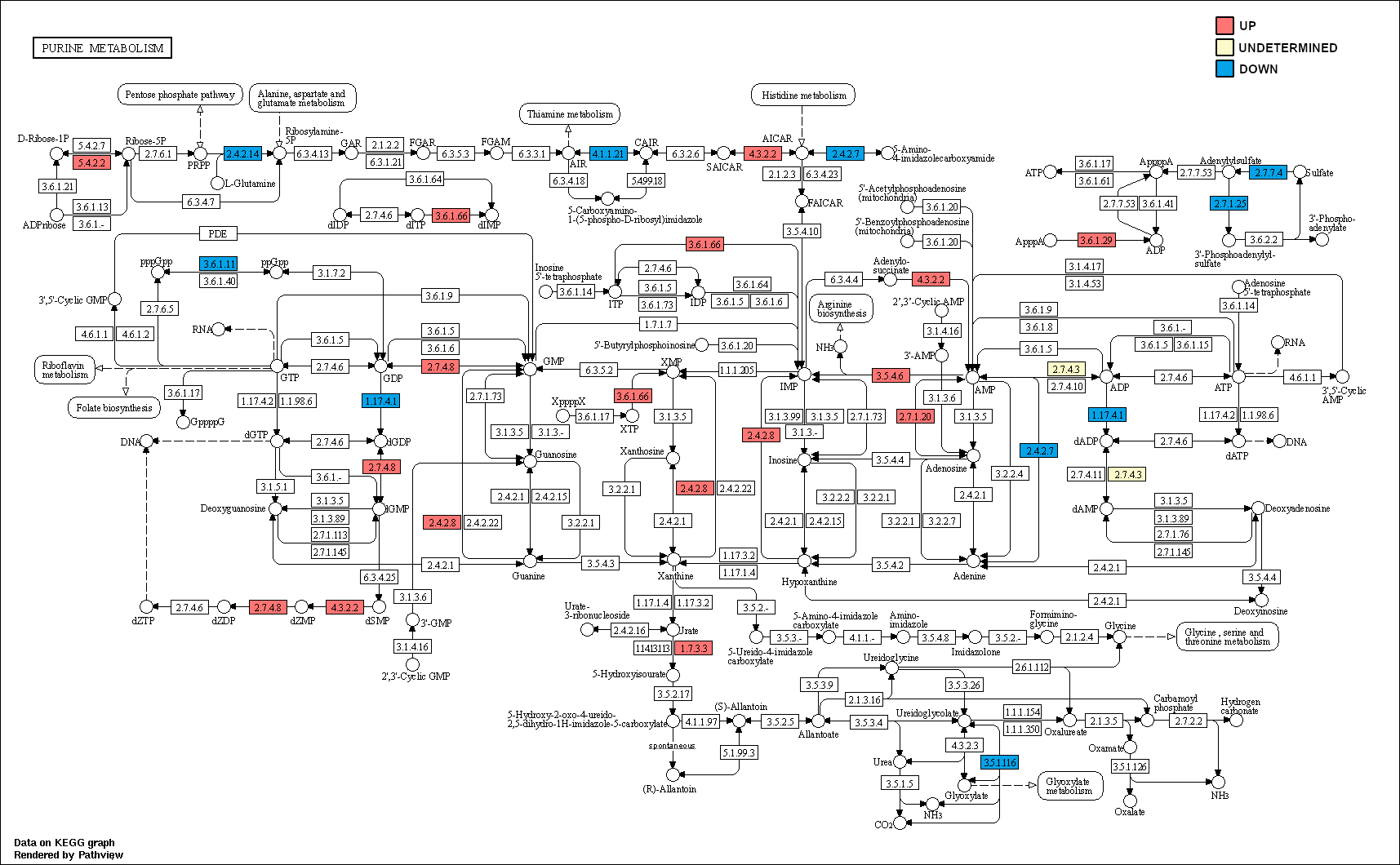

Supplement: Supplementary file 2 [file Data_Sheet_2.ZIP › Supplementary_Figure_4/Supplementary_Figure_4.018.png]

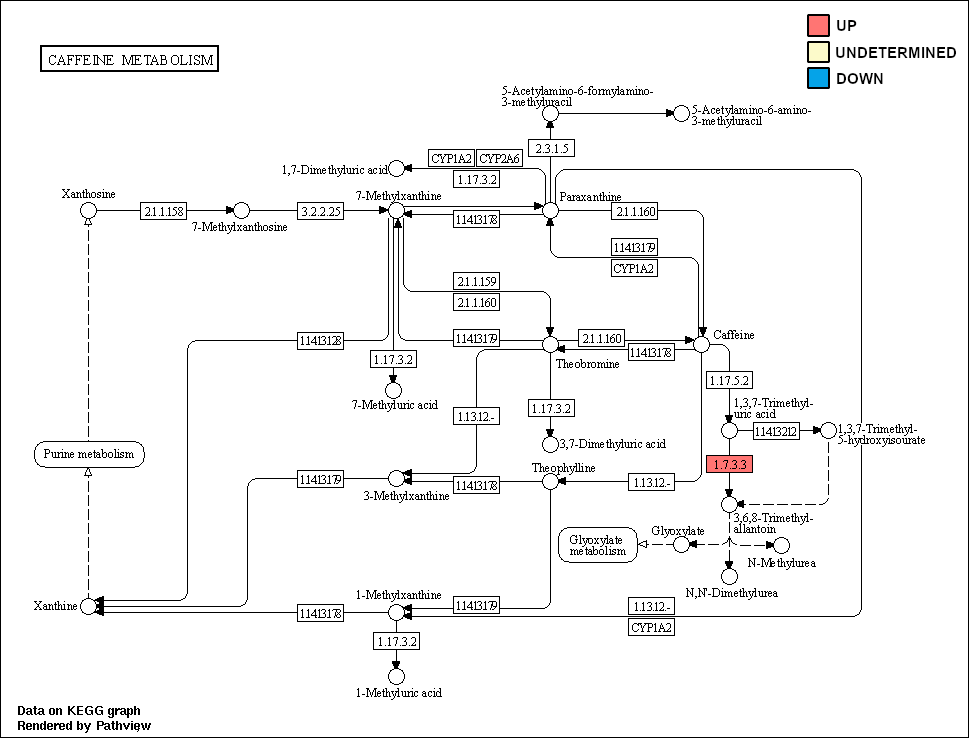

Supplement: Supplementary file 2 [file Data_Sheet_2.ZIP › Supplementary_Figure_4/Supplementary_Figure_4.019.png]

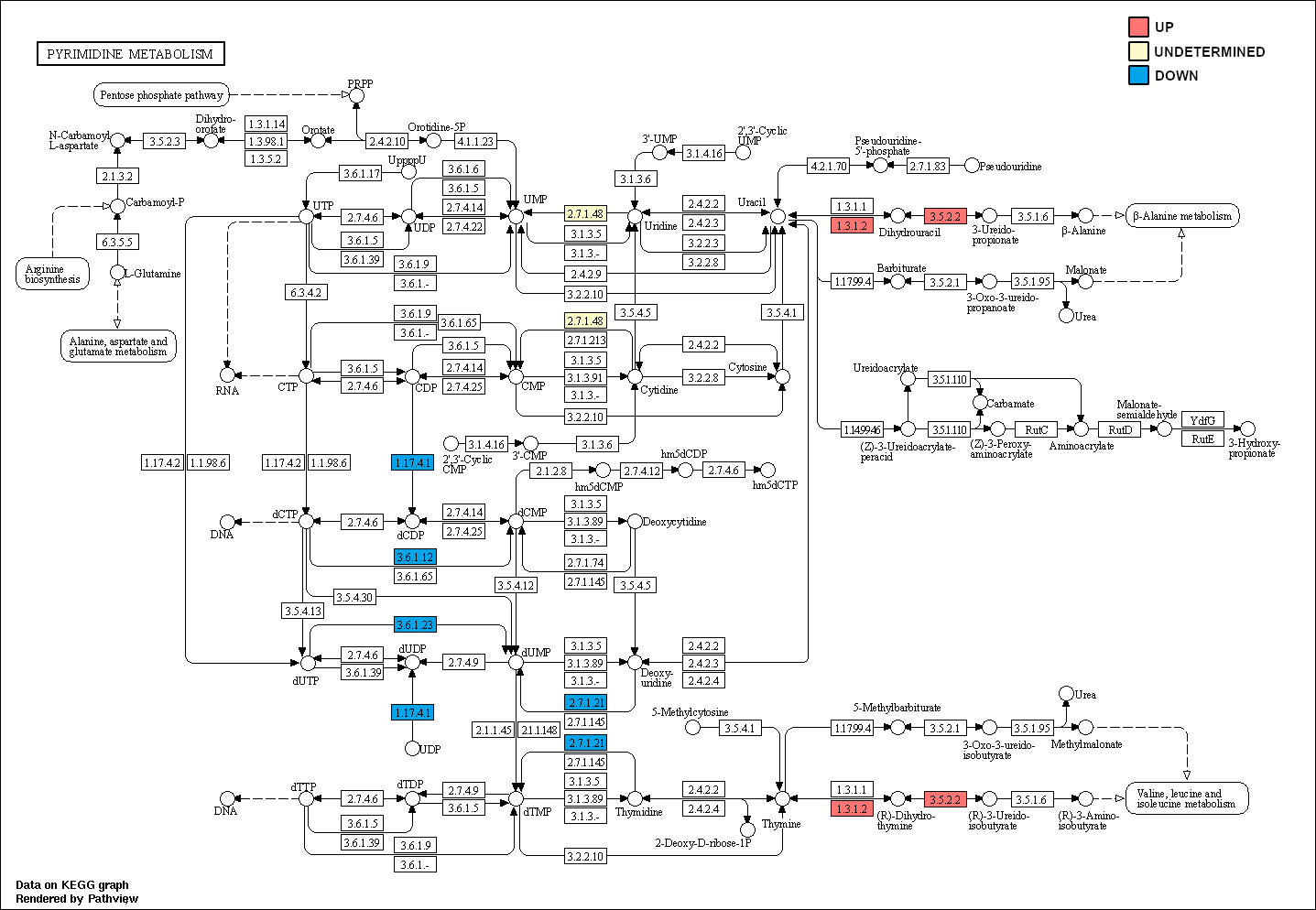

Supplement: Supplementary file 2 [file Data_Sheet_2.ZIP › Supplementary_Figure_4/Supplementary_Figure_4.020.png]

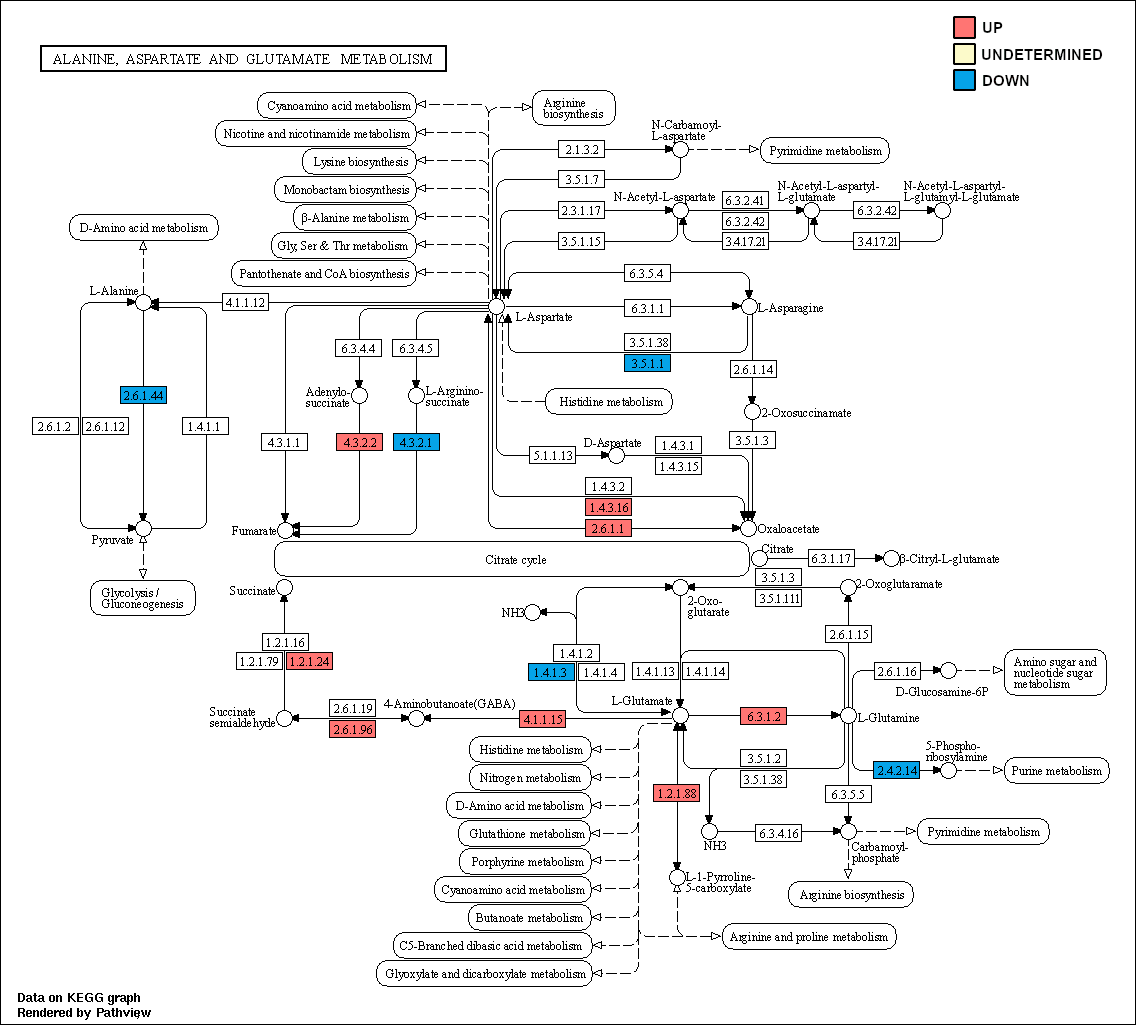

Supplement: Supplementary file 2 [file Data_Sheet_2.ZIP › Supplementary_Figure_4/Supplementary_Figure_4.021.png]

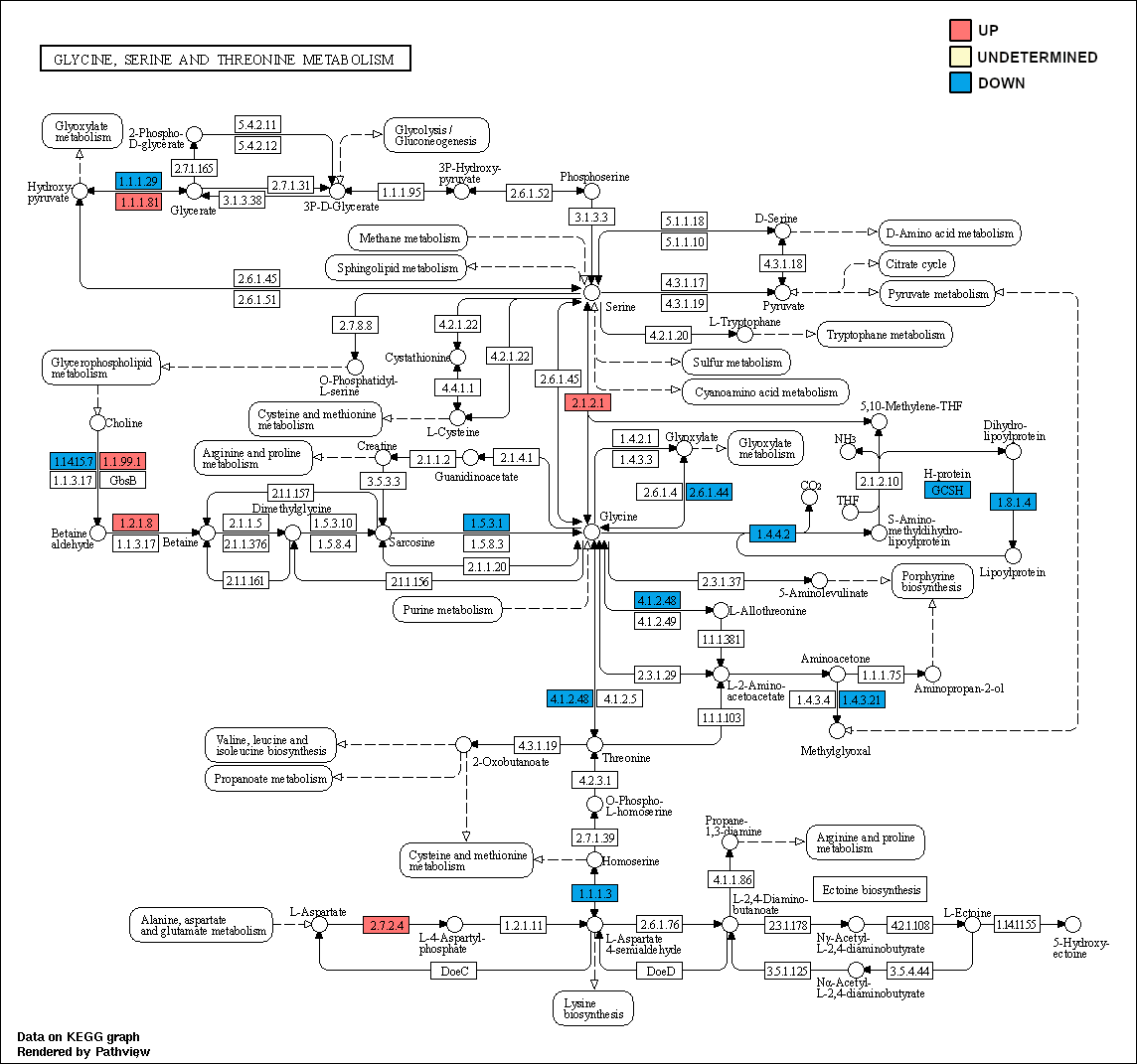

Supplement: Supplementary file 2 [file Data_Sheet_2.ZIP › Supplementary_Figure_4/Supplementary_Figure_4.022.png]

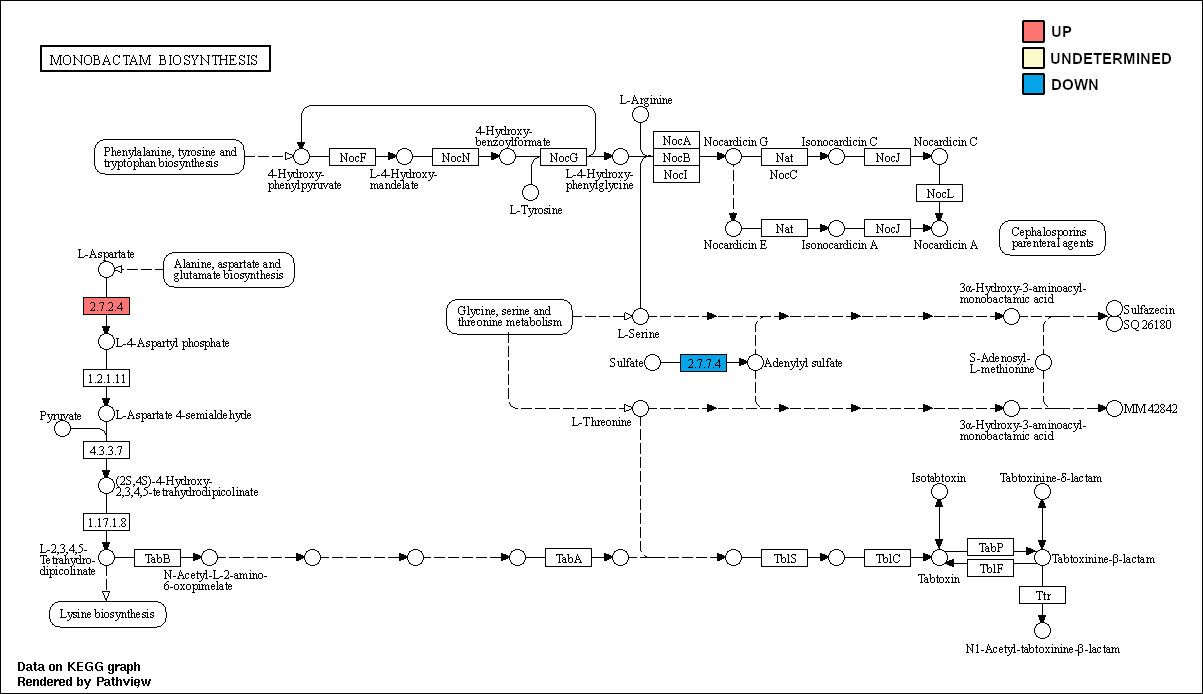

Supplement: Supplementary file 2 [file Data_Sheet_2.ZIP › Supplementary_Figure_4/Supplementary_Figure_4.023.png]

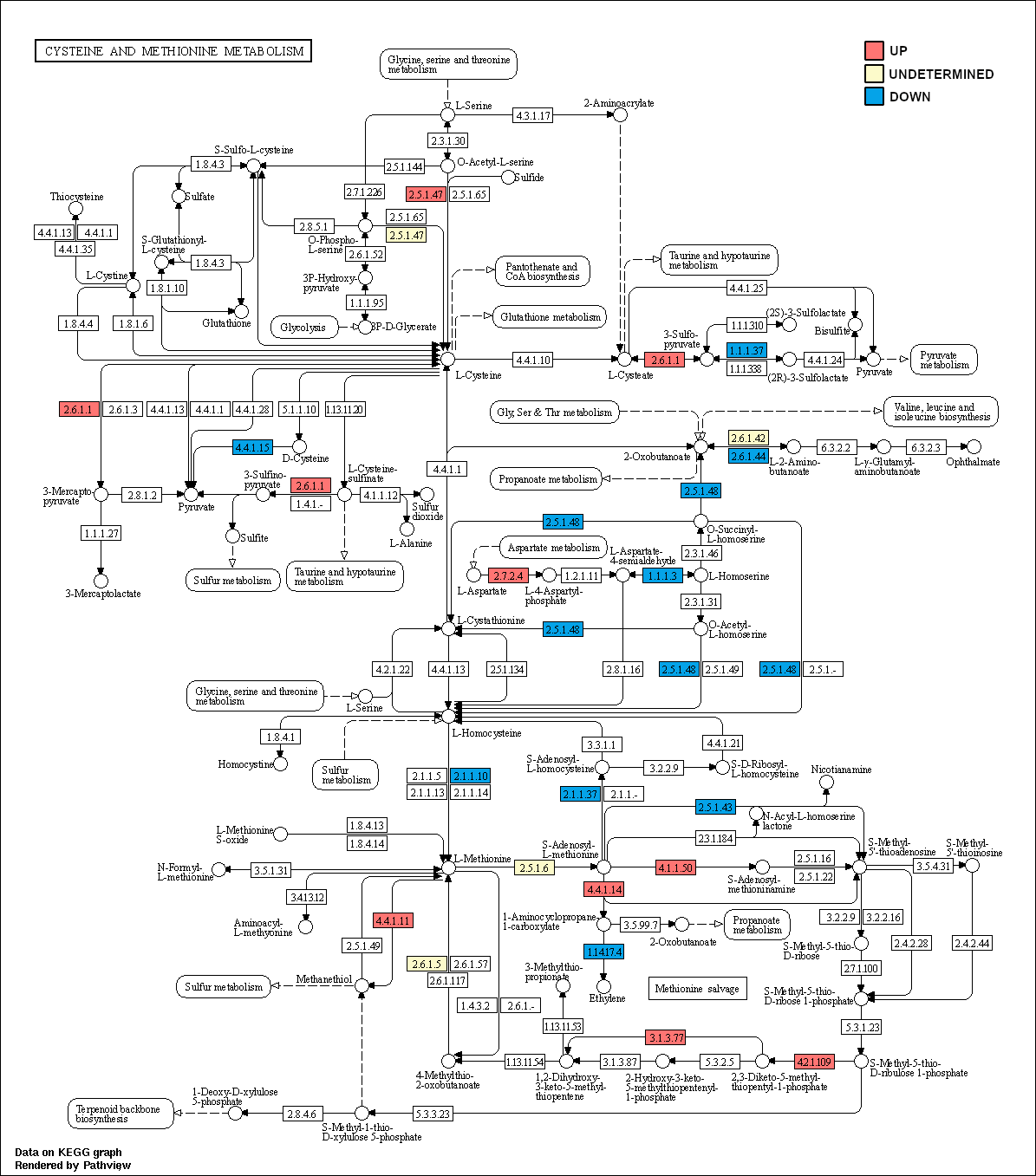

Supplement: Supplementary file 2 [file Data_Sheet_2.ZIP › Supplementary_Figure_4/Supplementary_Figure_4.024.png]

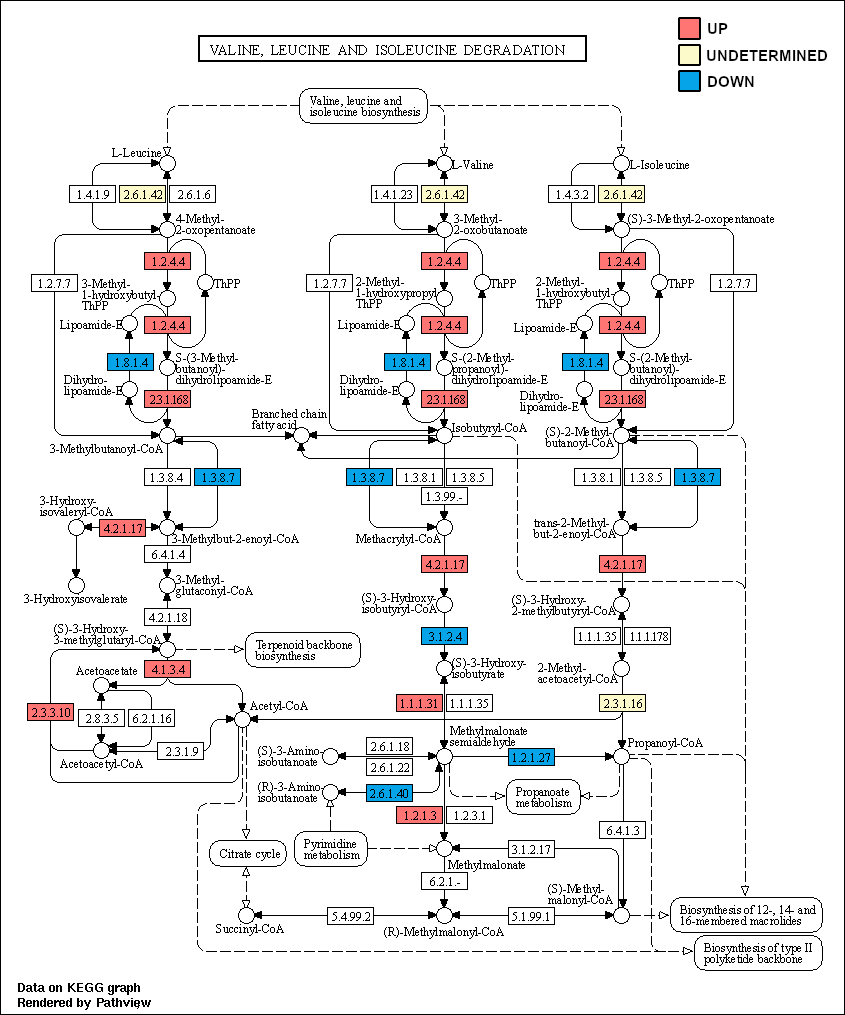

Supplement: Supplementary file 2 [file Data_Sheet_2.ZIP › Supplementary_Figure_4/Supplementary_Figure_4.025.png]

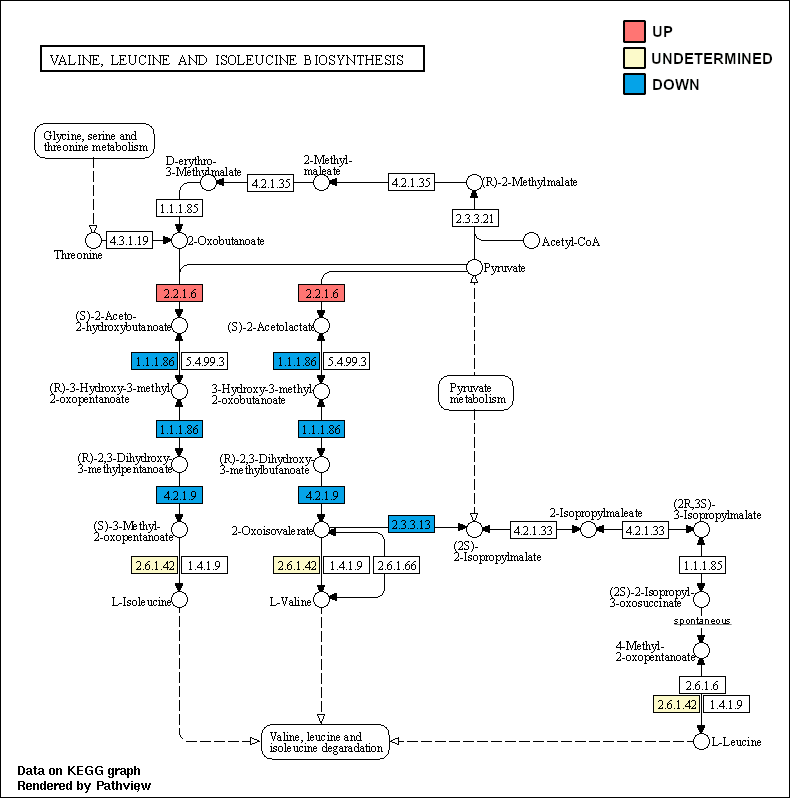

Supplement: Supplementary file 2 [file Data_Sheet_2.ZIP › Supplementary_Figure_4/Supplementary_Figure_4.026.png]

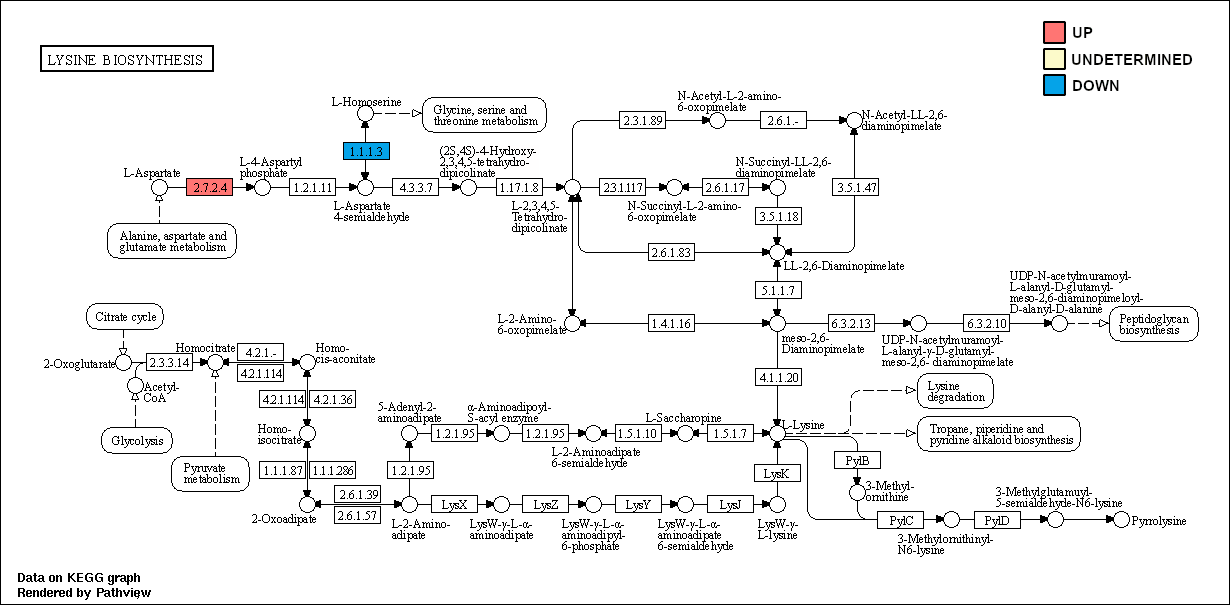

Supplement: Supplementary file 2 [file Data_Sheet_2.ZIP › Supplementary_Figure_4/Supplementary_Figure_4.027.png]

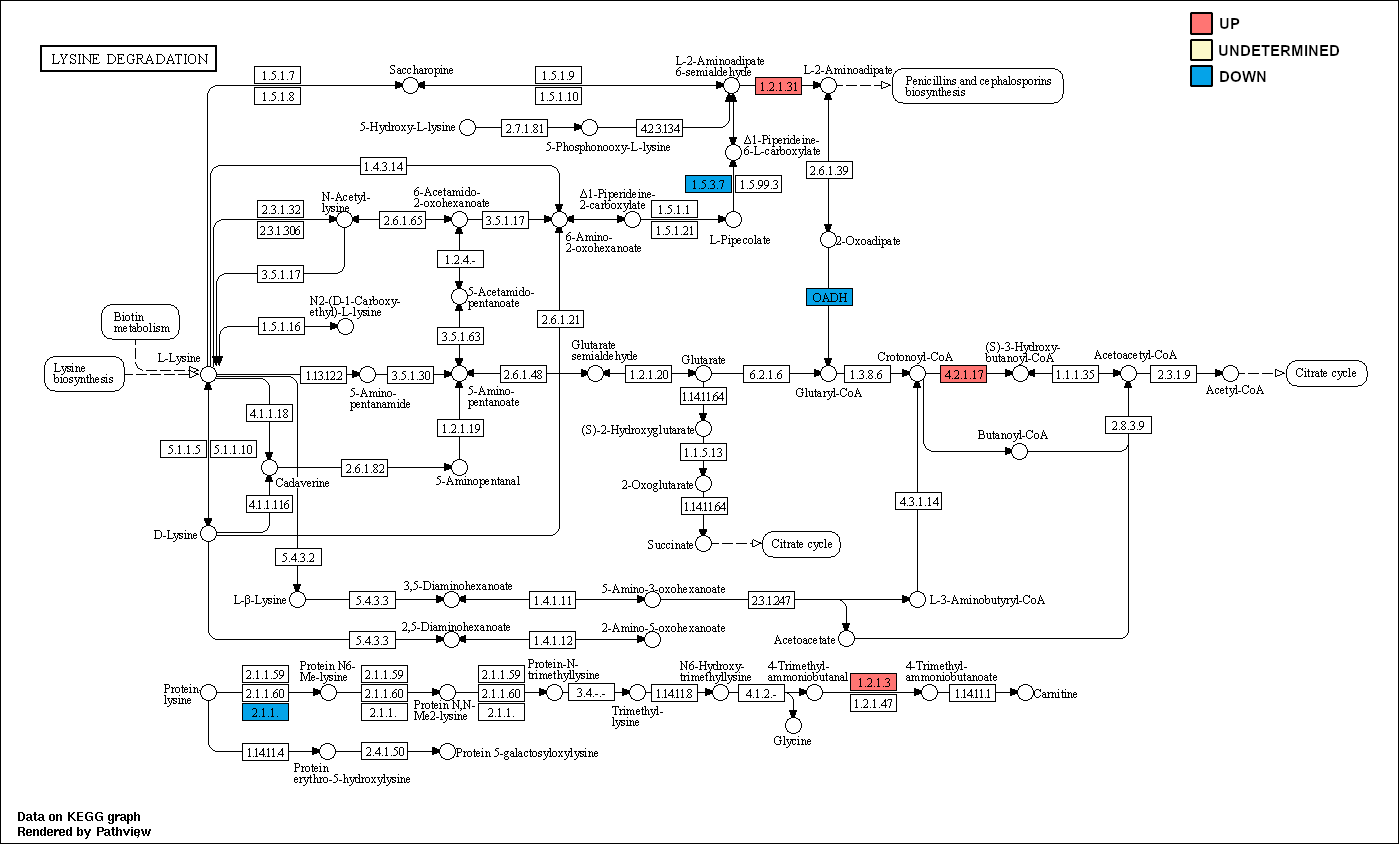

Supplement: Supplementary file 2 [file Data_Sheet_2.ZIP › Supplementary_Figure_4/Supplementary_Figure_4.028.png]

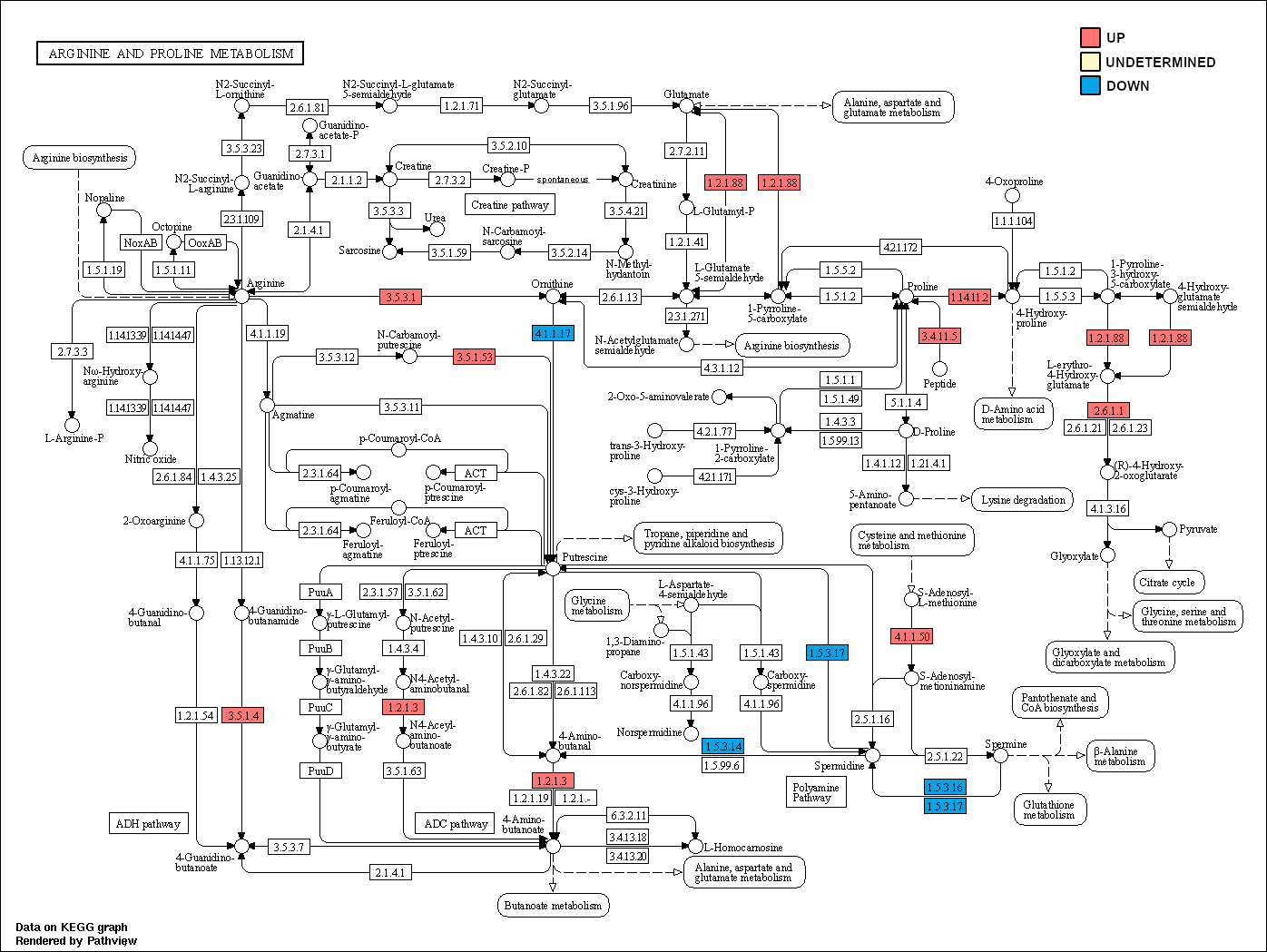

Supplement: Supplementary file 2 [file Data_Sheet_2.ZIP › Supplementary_Figure_4/Supplementary_Figure_4.029.png]

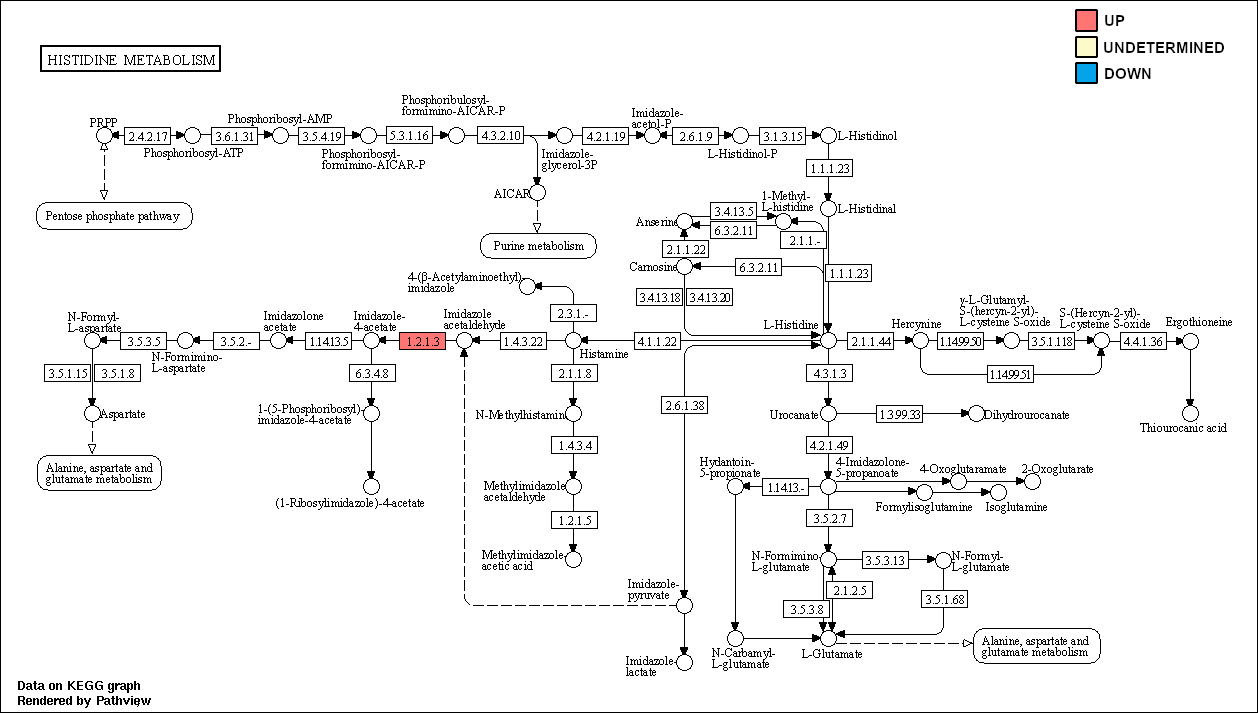

Supplement: Supplementary file 2 [file Data_Sheet_2.ZIP › Supplementary_Figure_4/Supplementary_Figure_4.030.png]

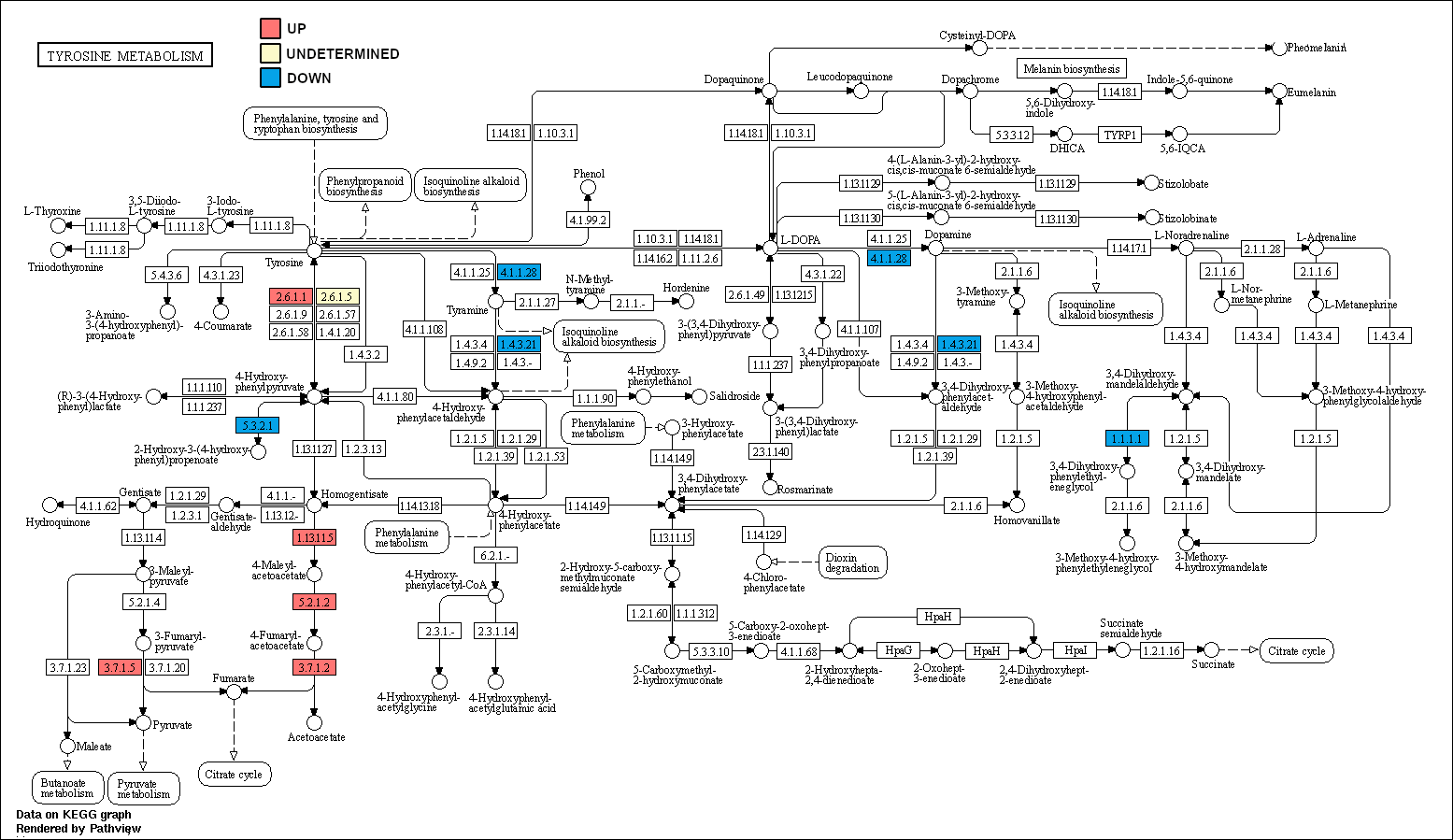

Supplement: Supplementary file 2 [file Data_Sheet_2.ZIP › Supplementary_Figure_4/Supplementary_Figure_4.031.png]

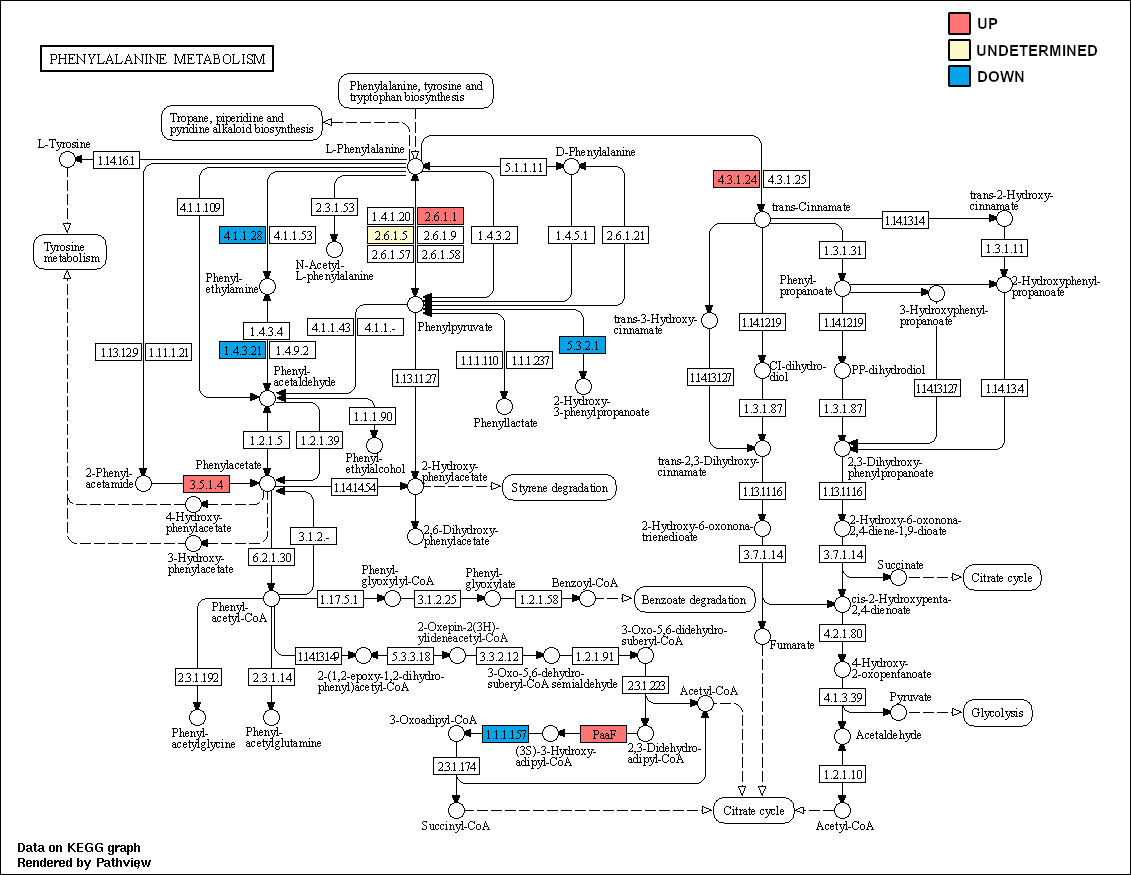

Supplement: Supplementary file 2 [file Data_Sheet_2.ZIP › Supplementary_Figure_4/Supplementary_Figure_4.032.png]

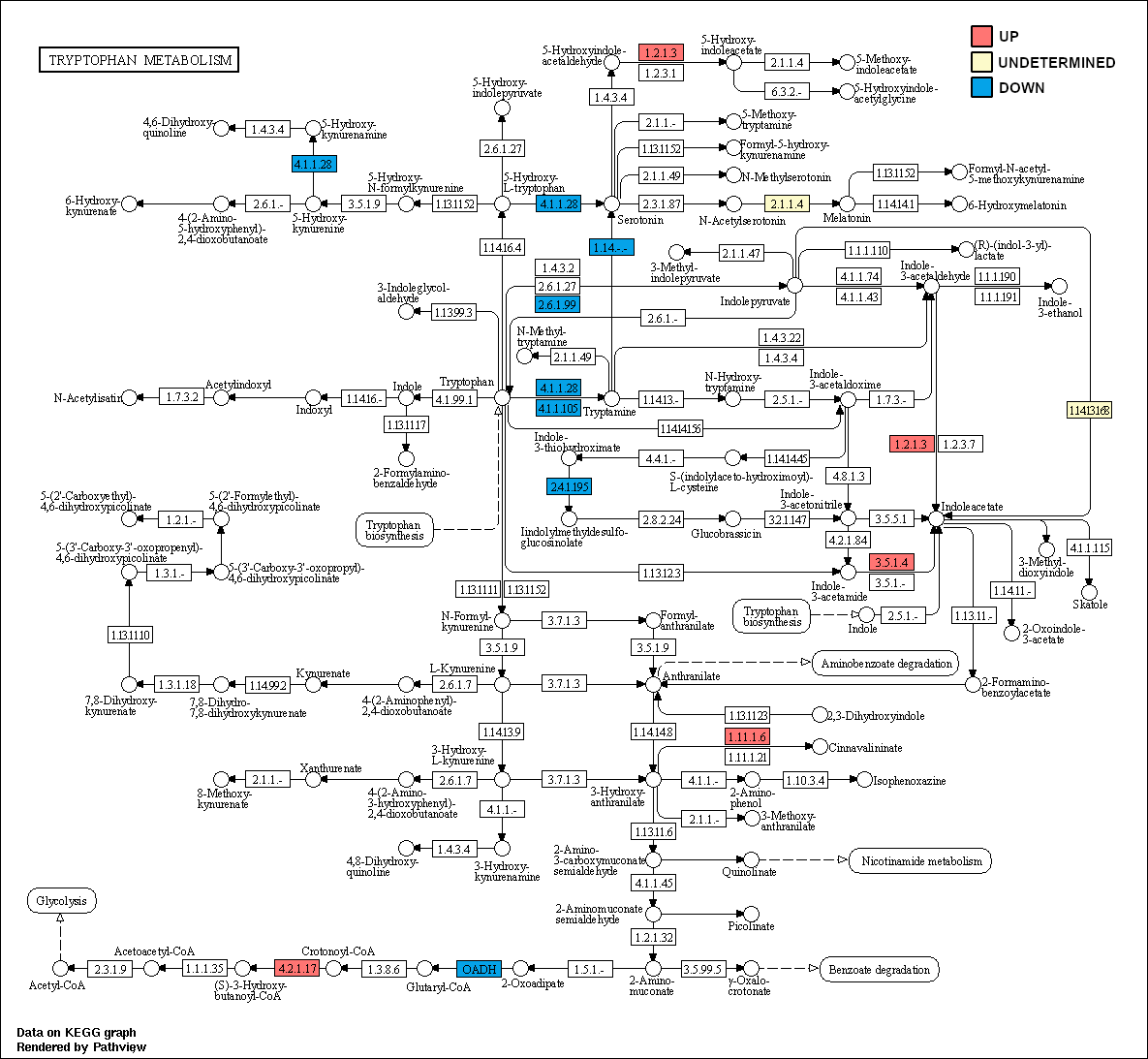

Supplement: Supplementary file 2 [file Data_Sheet_2.ZIP › Supplementary_Figure_4/Supplementary_Figure_4.033.png]

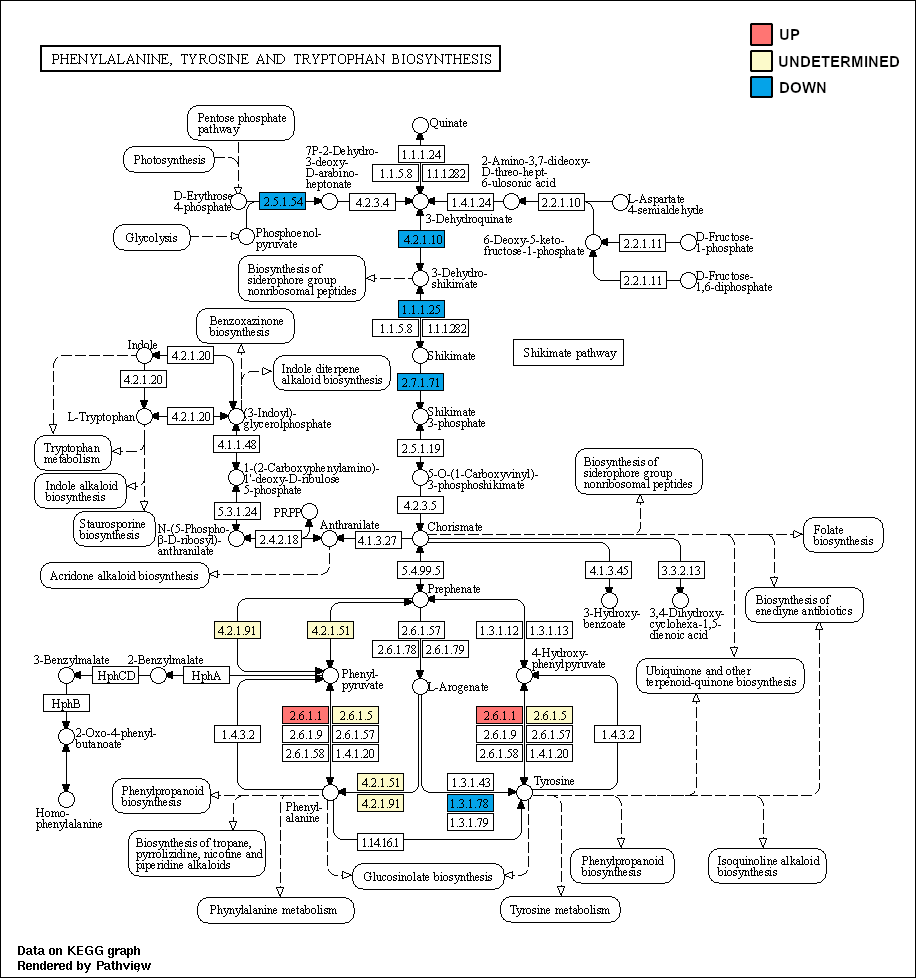

Supplement: Supplementary file 2 [file Data_Sheet_2.ZIP › Supplementary_Figure_4/Supplementary_Figure_4.034.png]

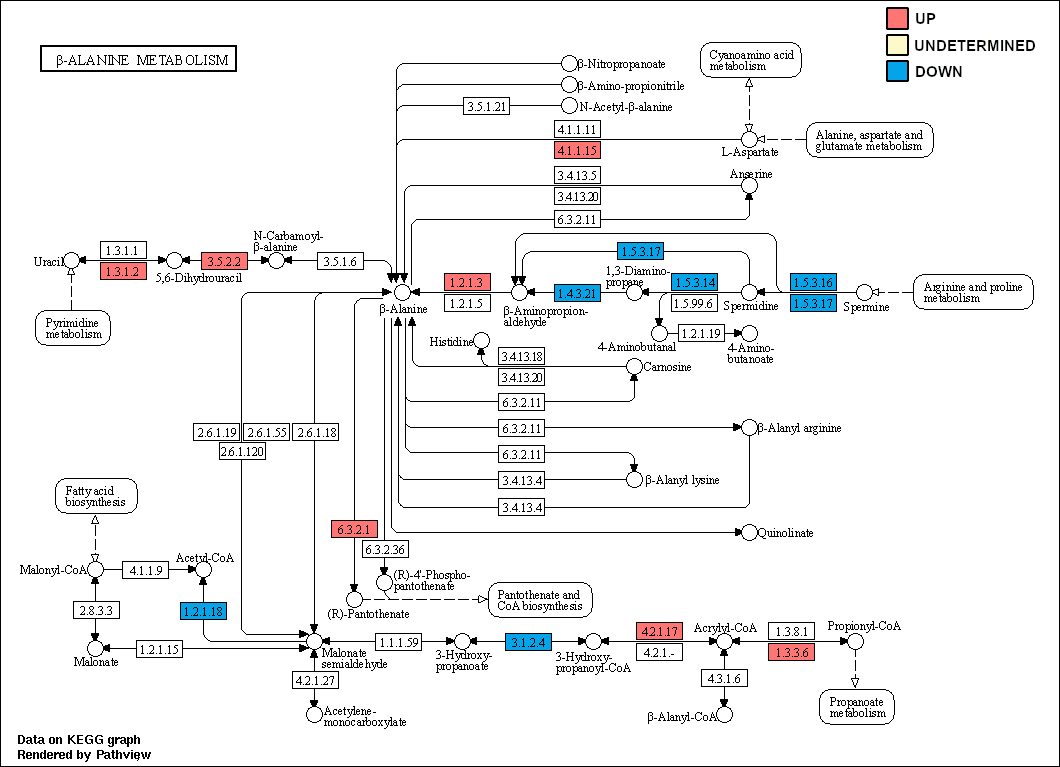

Supplement: Supplementary file 2 [file Data_Sheet_2.ZIP › Supplementary_Figure_4/Supplementary_Figure_4.035.png]

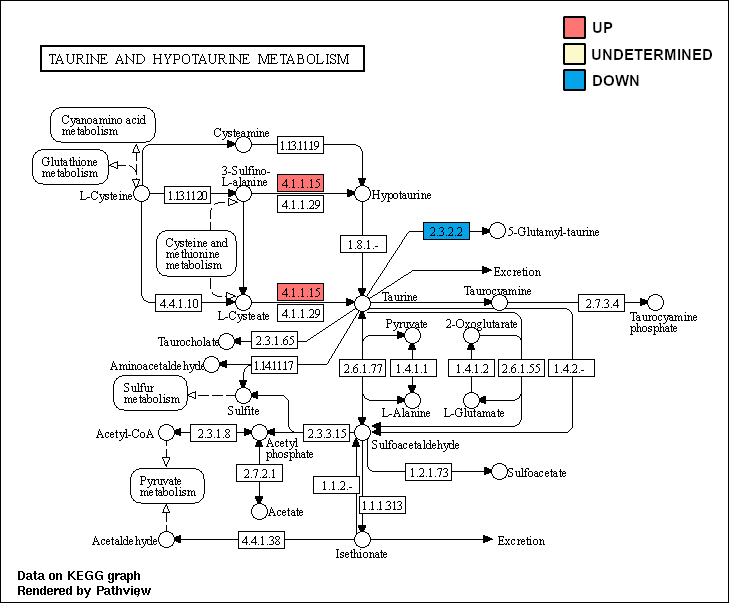

Supplement: Supplementary file 2 [file Data_Sheet_2.ZIP › Supplementary_Figure_4/Supplementary_Figure_4.036.png]

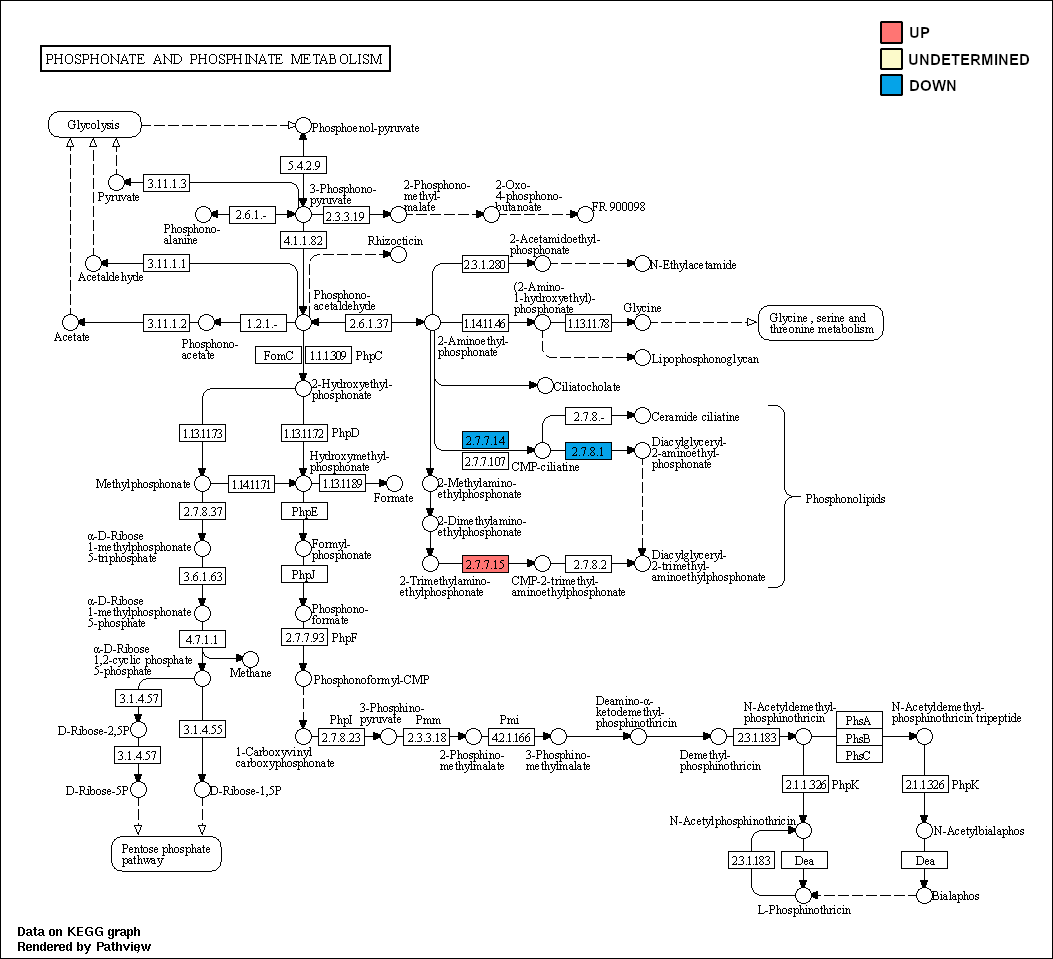

Supplement: Supplementary file 2 [file Data_Sheet_2.ZIP › Supplementary_Figure_4/Supplementary_Figure_4.037.png]

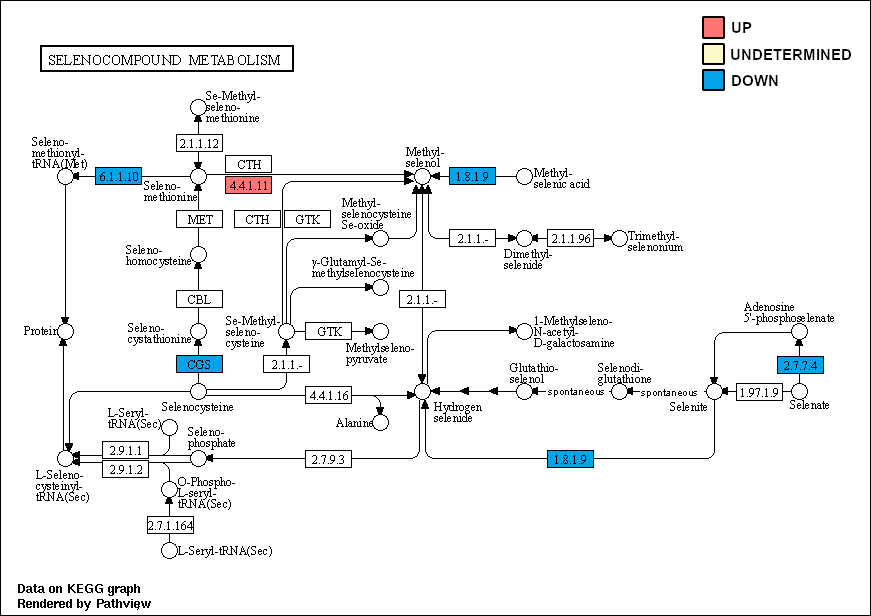

Supplement: Supplementary file 2 [file Data_Sheet_2.ZIP › Supplementary_Figure_4/Supplementary_Figure_4.038.png]

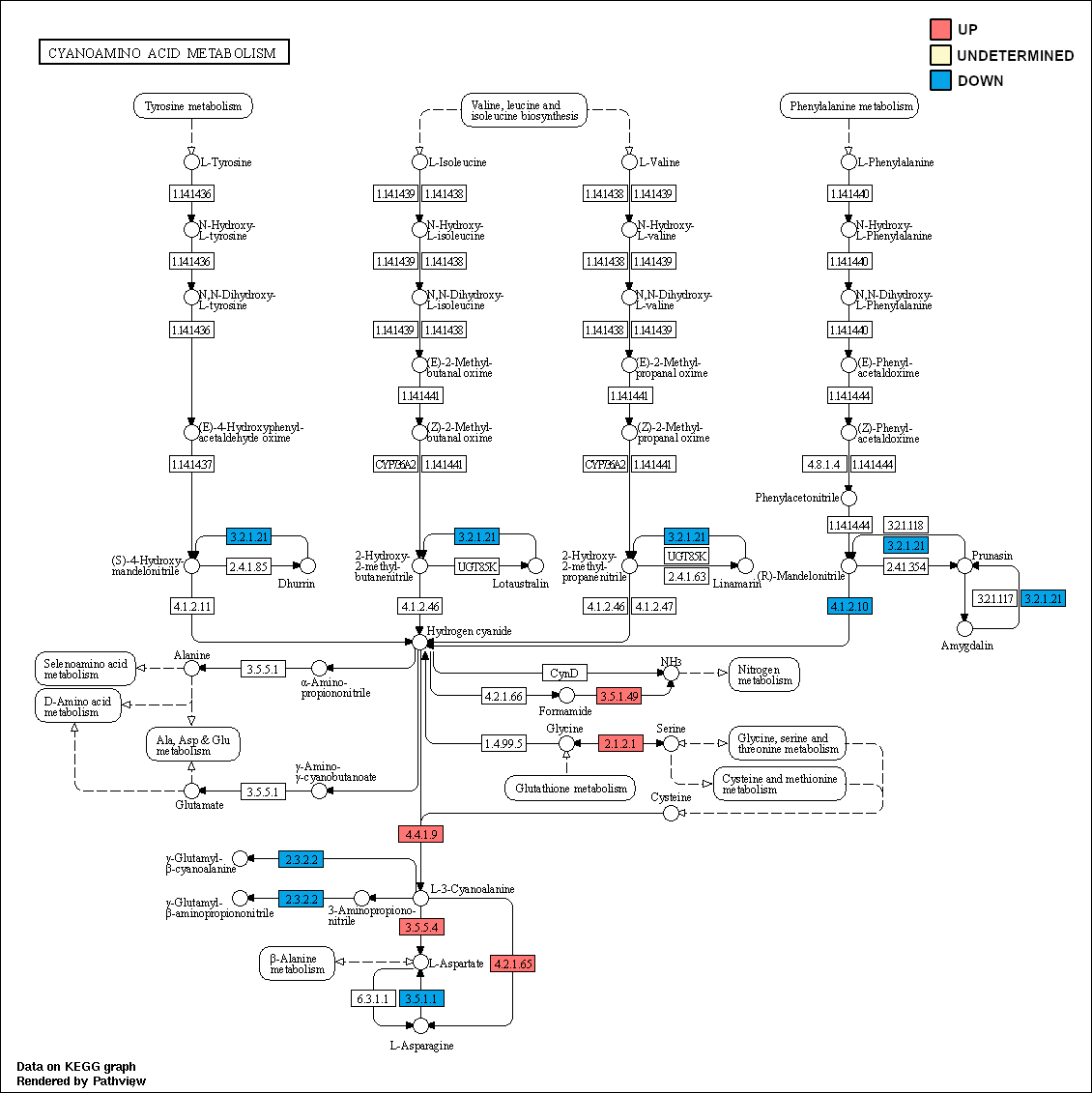

Supplement: Supplementary file 2 [file Data_Sheet_2.ZIP › Supplementary_Figure_4/Supplementary_Figure_4.039.png]

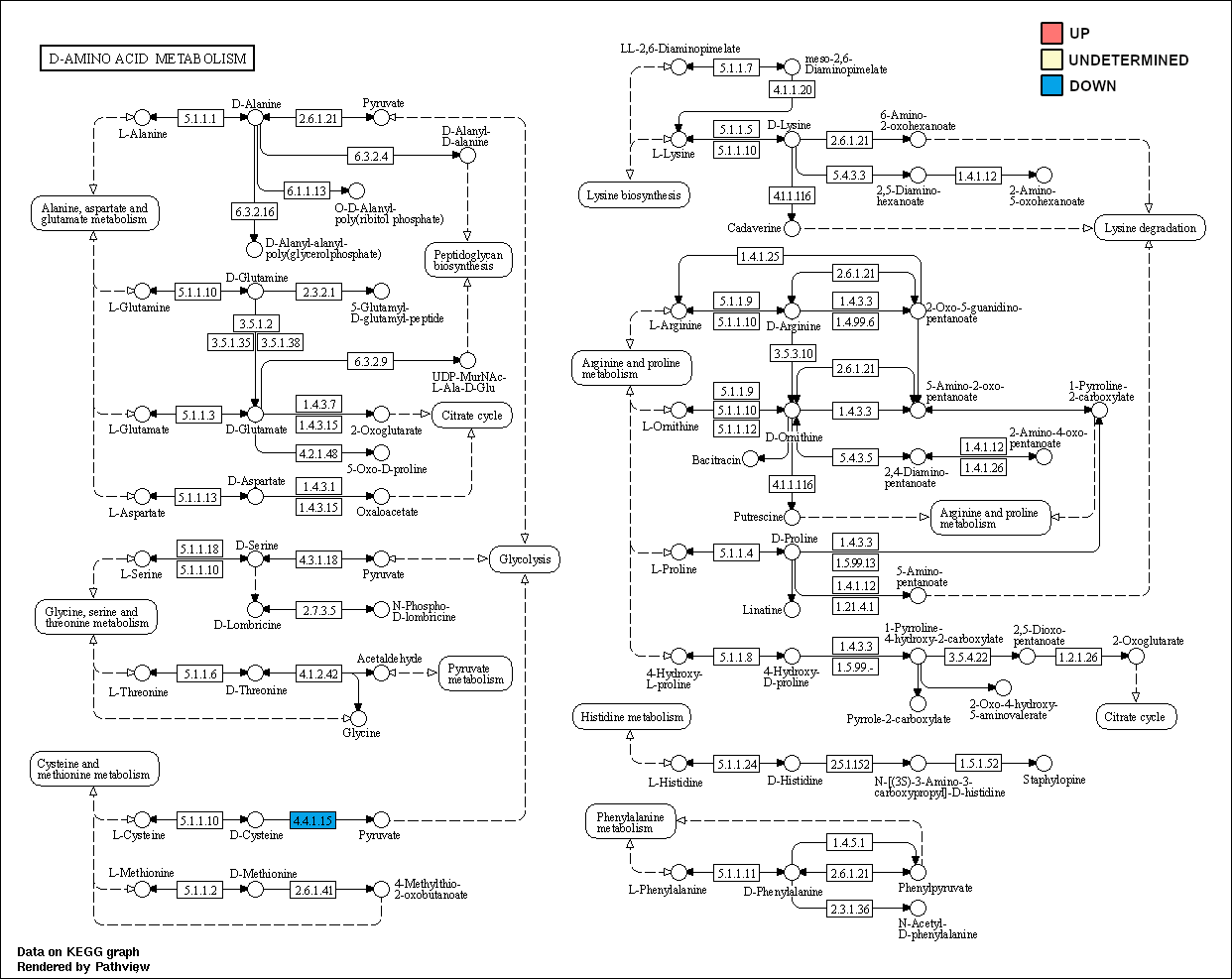

Supplement: Supplementary file 2 [file Data_Sheet_2.ZIP › Supplementary_Figure_4/Supplementary_Figure_4.040.png]

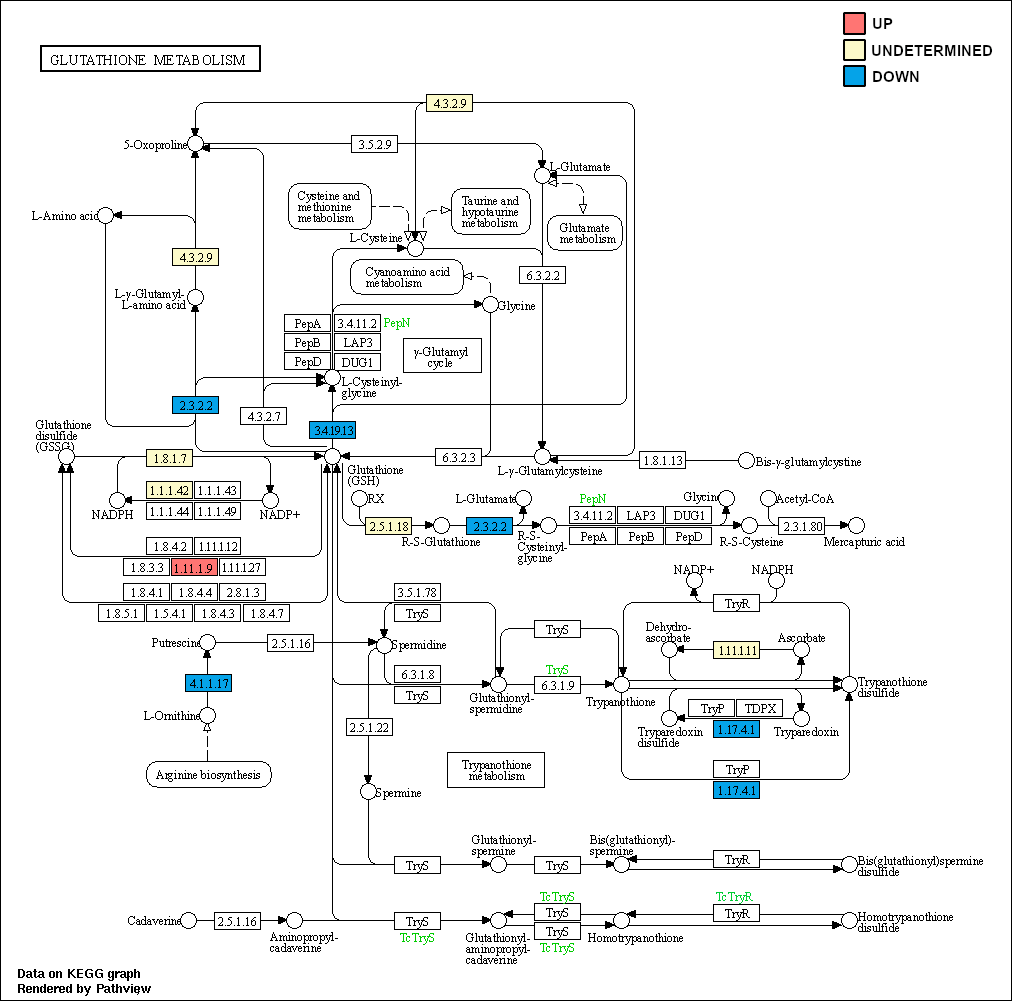

Supplement: Supplementary file 2 [file Data_Sheet_2.ZIP › Supplementary_Figure_4/Supplementary_Figure_4.041.png]

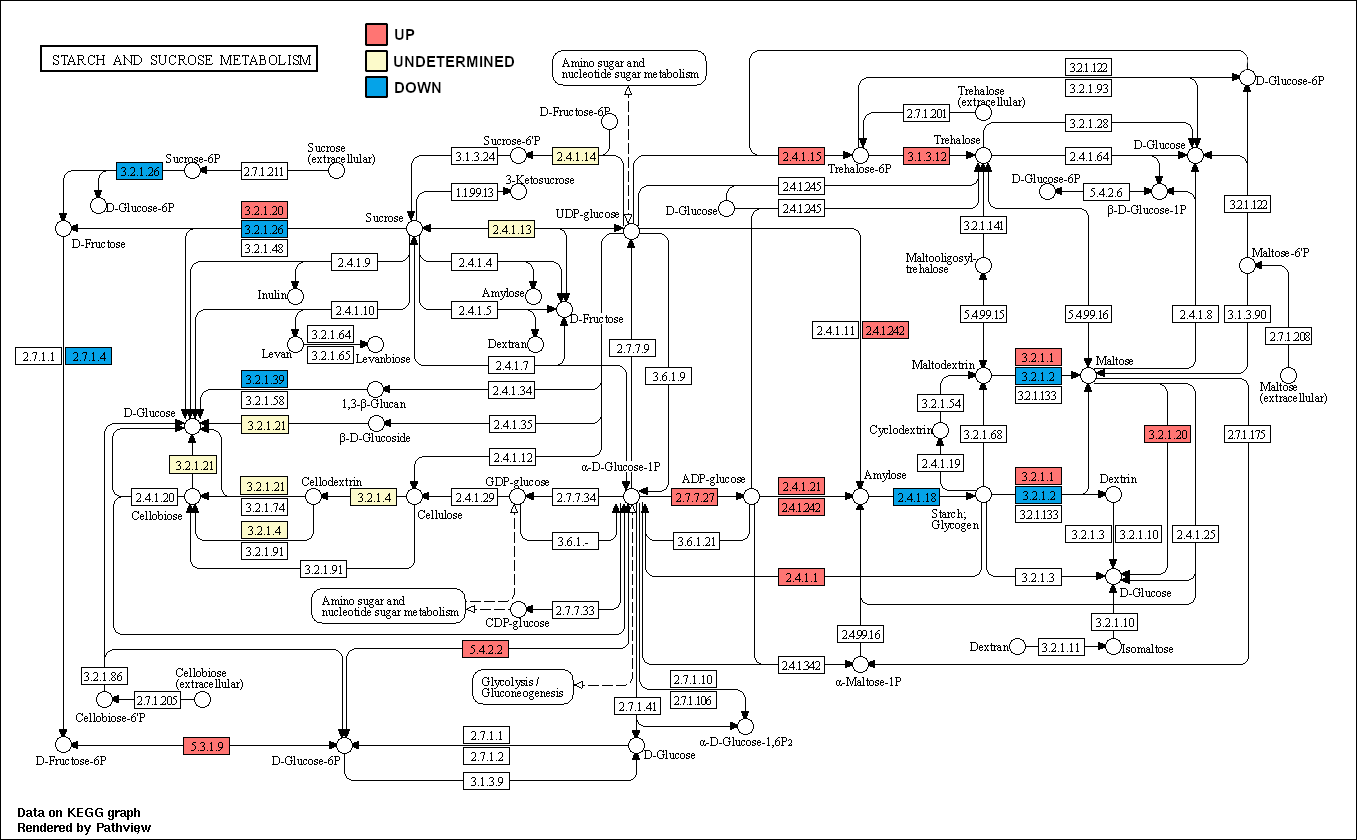

Supplement: Supplementary file 2 [file Data_Sheet_2.ZIP › Supplementary_Figure_4/Supplementary_Figure_4.042.png]

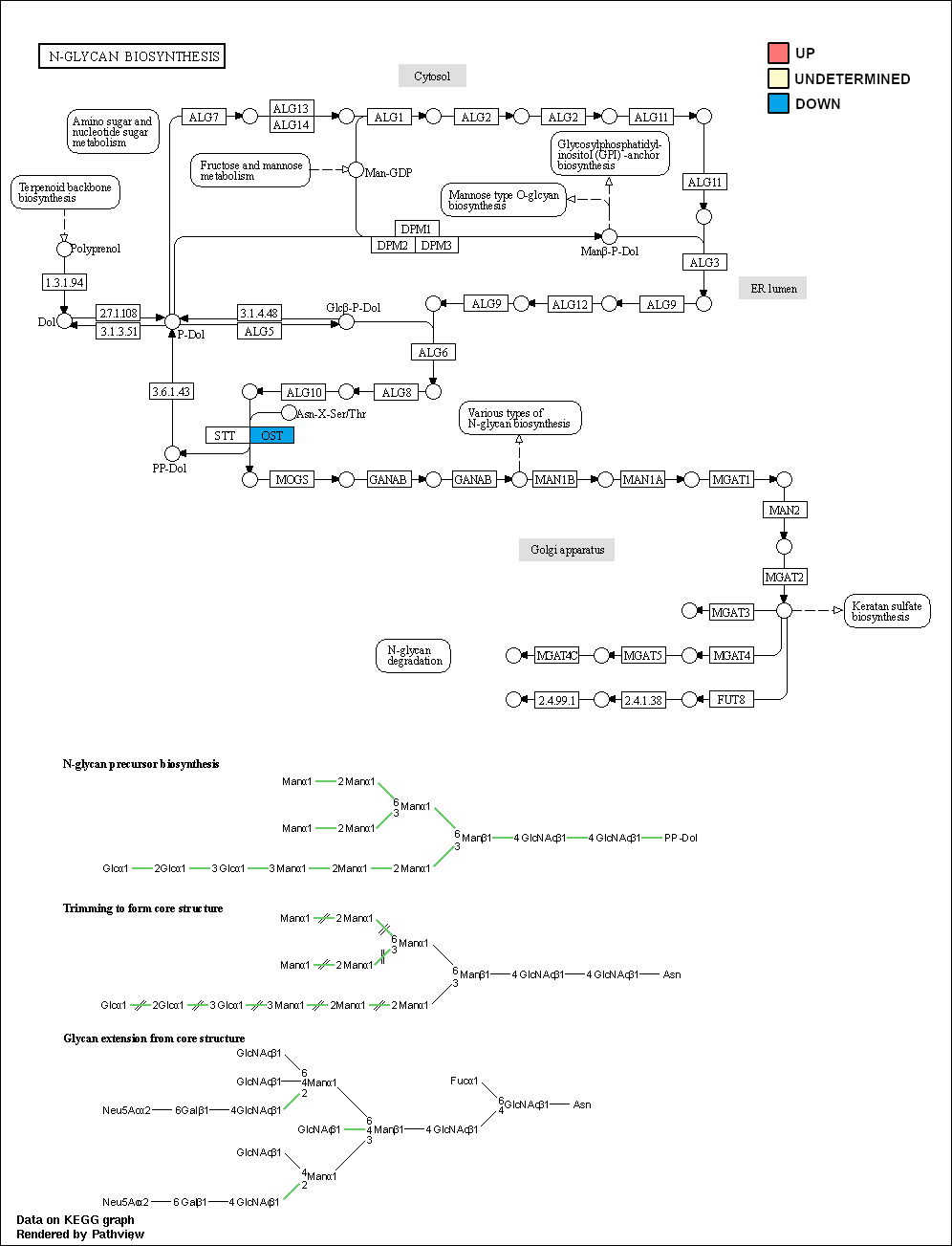

Supplement: Supplementary file 2 [file Data_Sheet_2.ZIP › Supplementary_Figure_4/Supplementary_Figure_4.043.png]

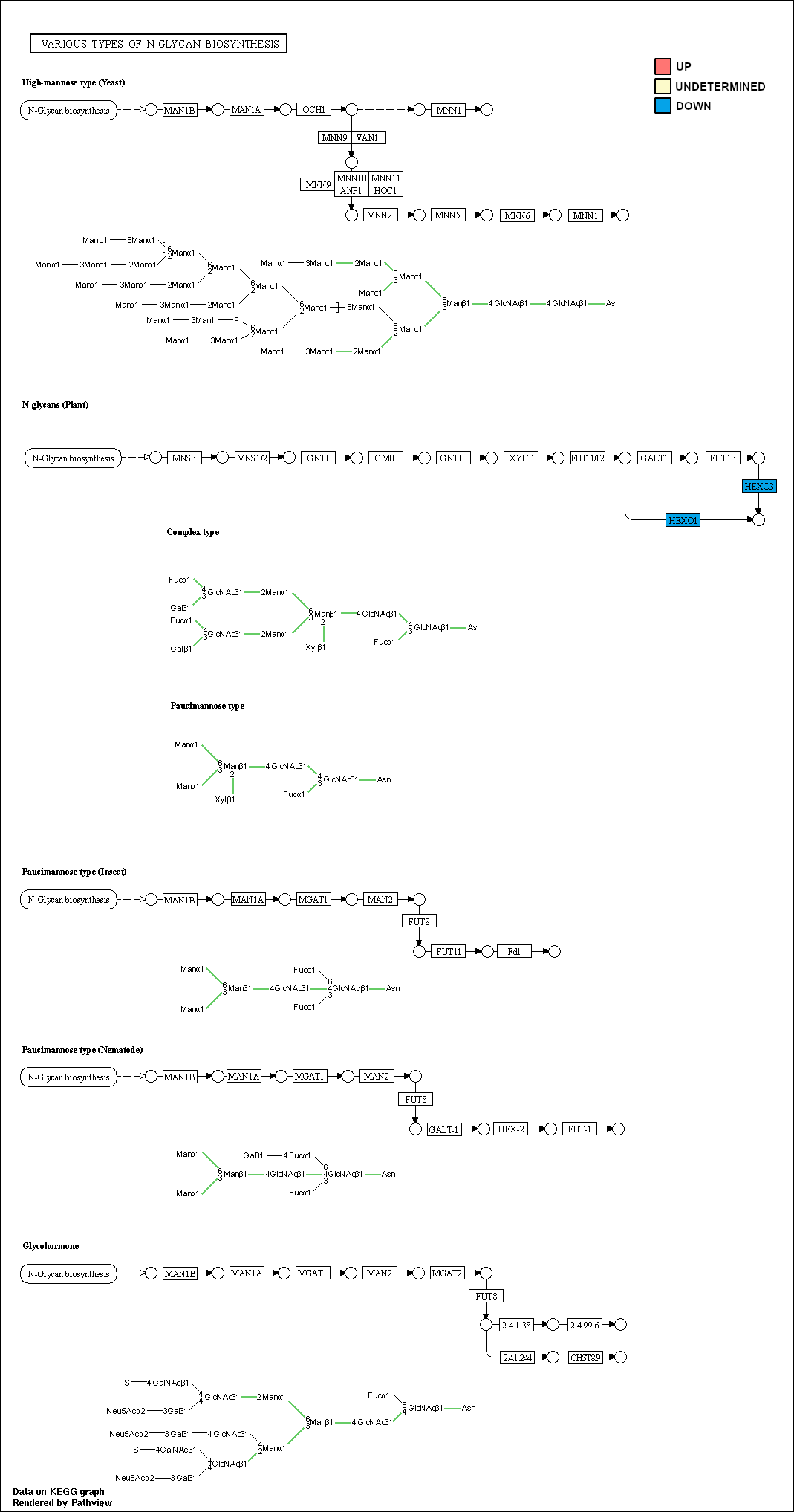

Supplement: Supplementary file 2 [file Data_Sheet_2.ZIP › Supplementary_Figure_4/Supplementary_Figure_4.044.png]

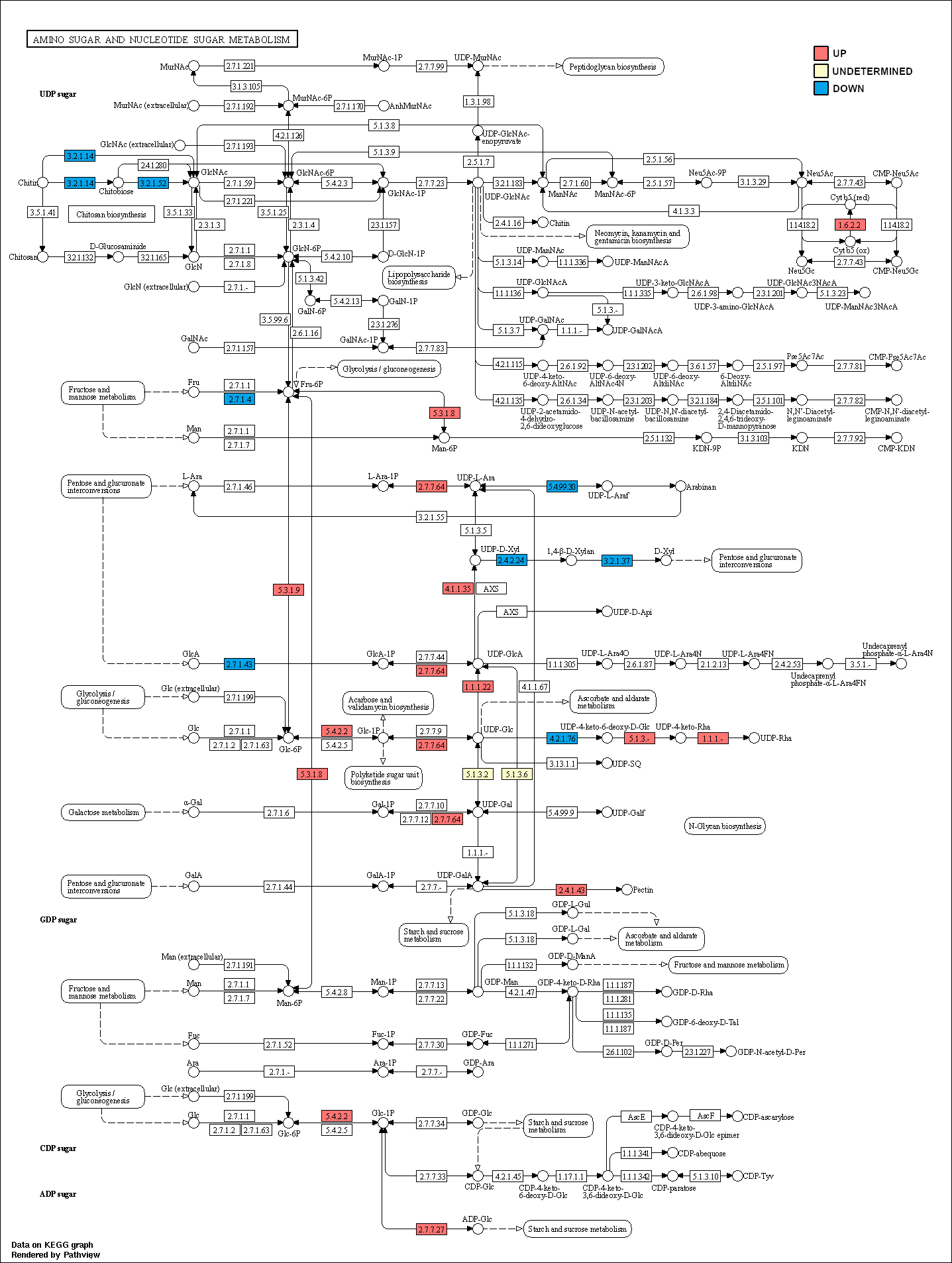

Supplement: Supplementary file 2 [file Data_Sheet_2.ZIP › Supplementary_Figure_4/Supplementary_Figure_4.045.png]

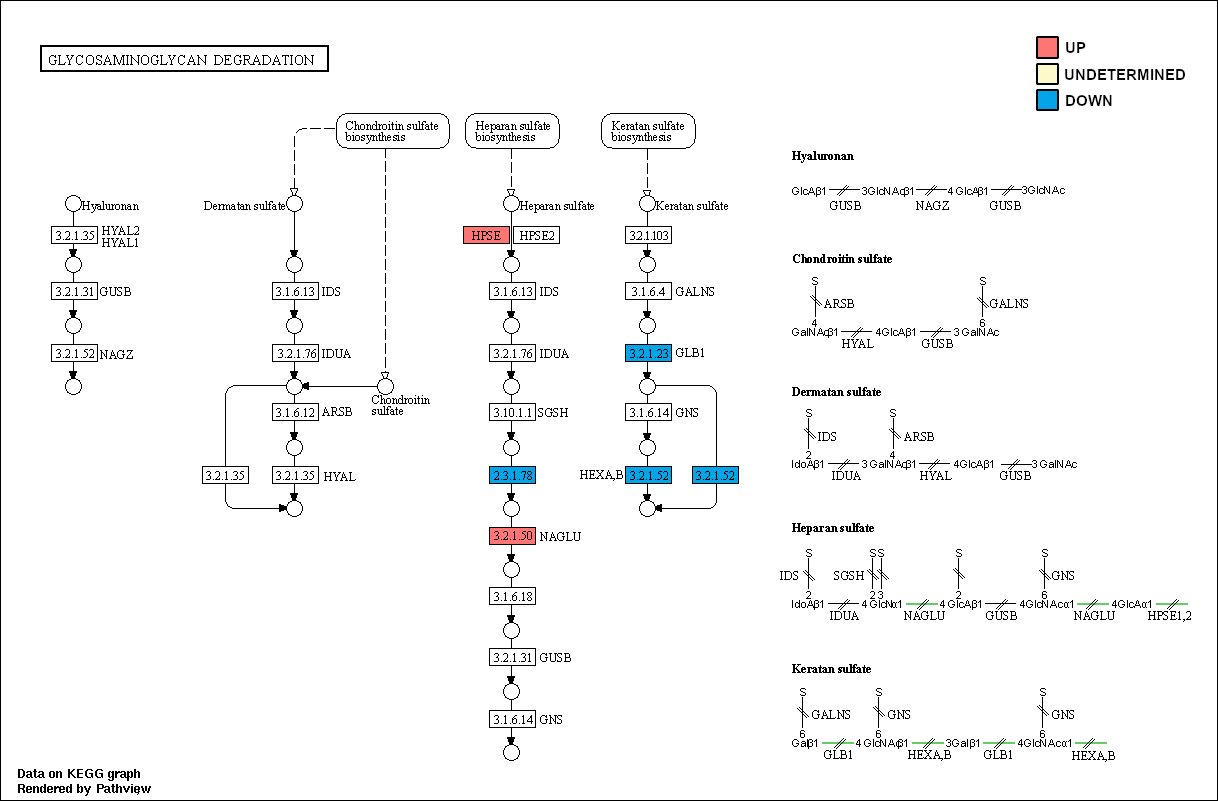

Supplement: Supplementary file 2 [file Data_Sheet_2.ZIP › Supplementary_Figure_4/Supplementary_Figure_4.046.png]

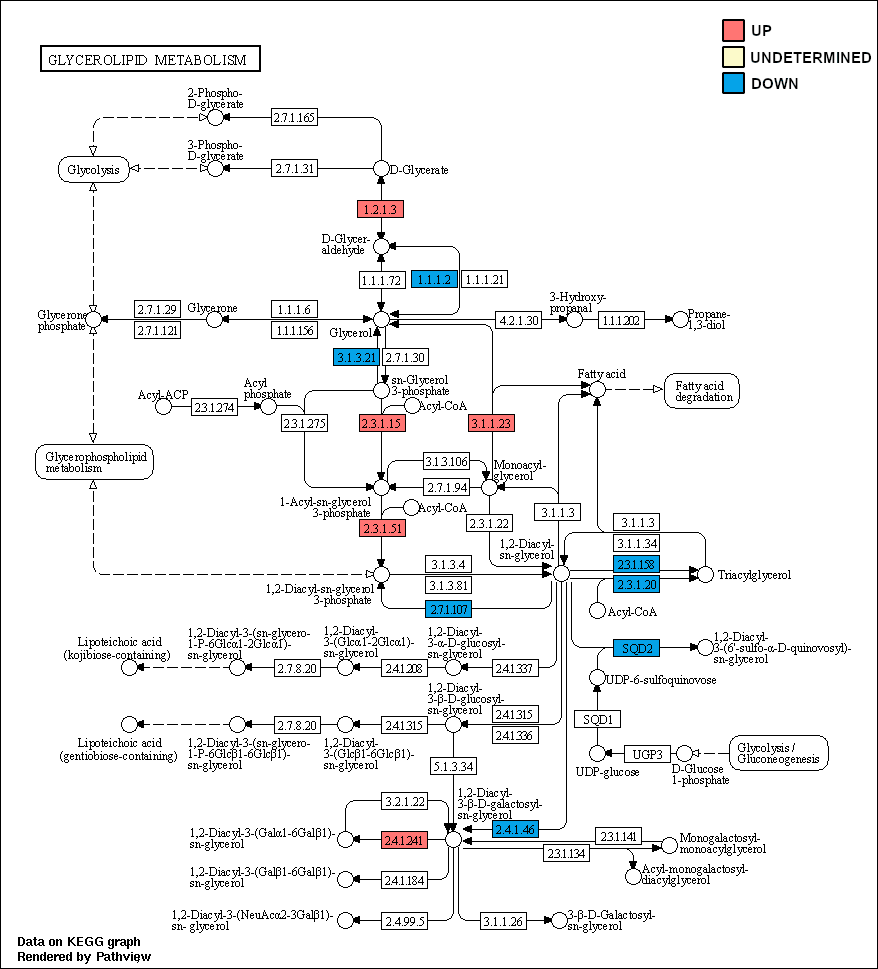

Supplement: Supplementary file 2 [file Data_Sheet_2.ZIP › Supplementary_Figure_4/Supplementary_Figure_4.047.png]

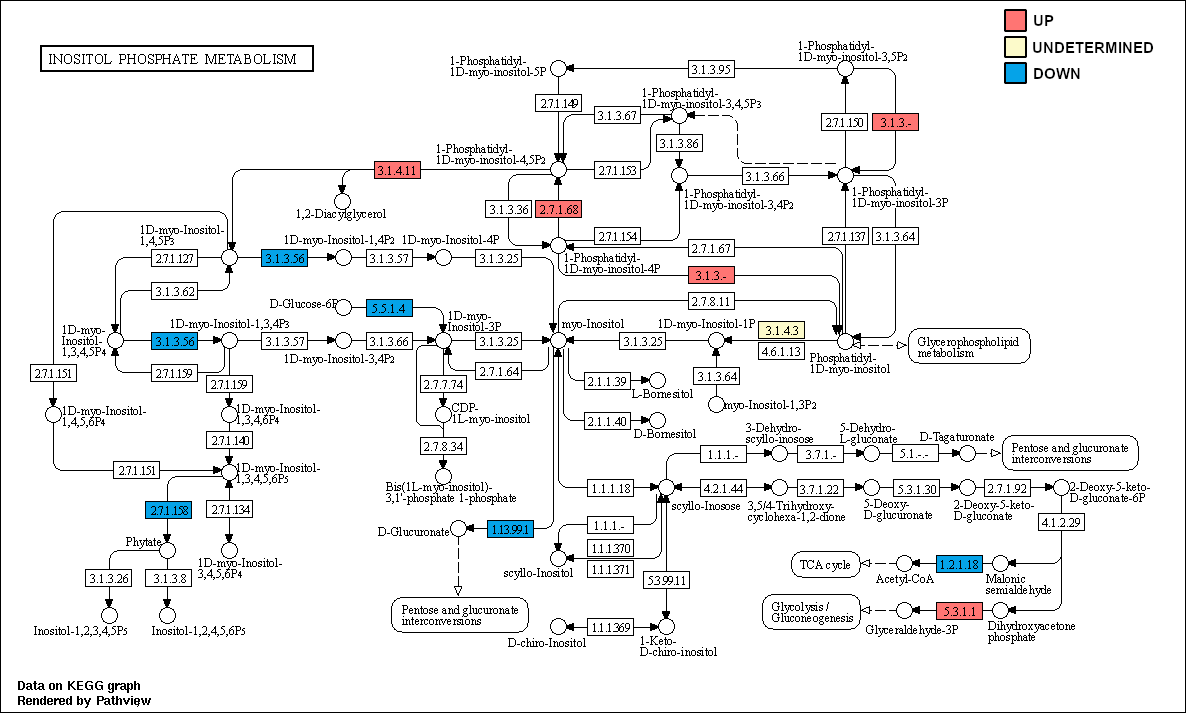

Supplement: Supplementary file 2 [file Data_Sheet_2.ZIP › Supplementary_Figure_4/Supplementary_Figure_4.048.png]

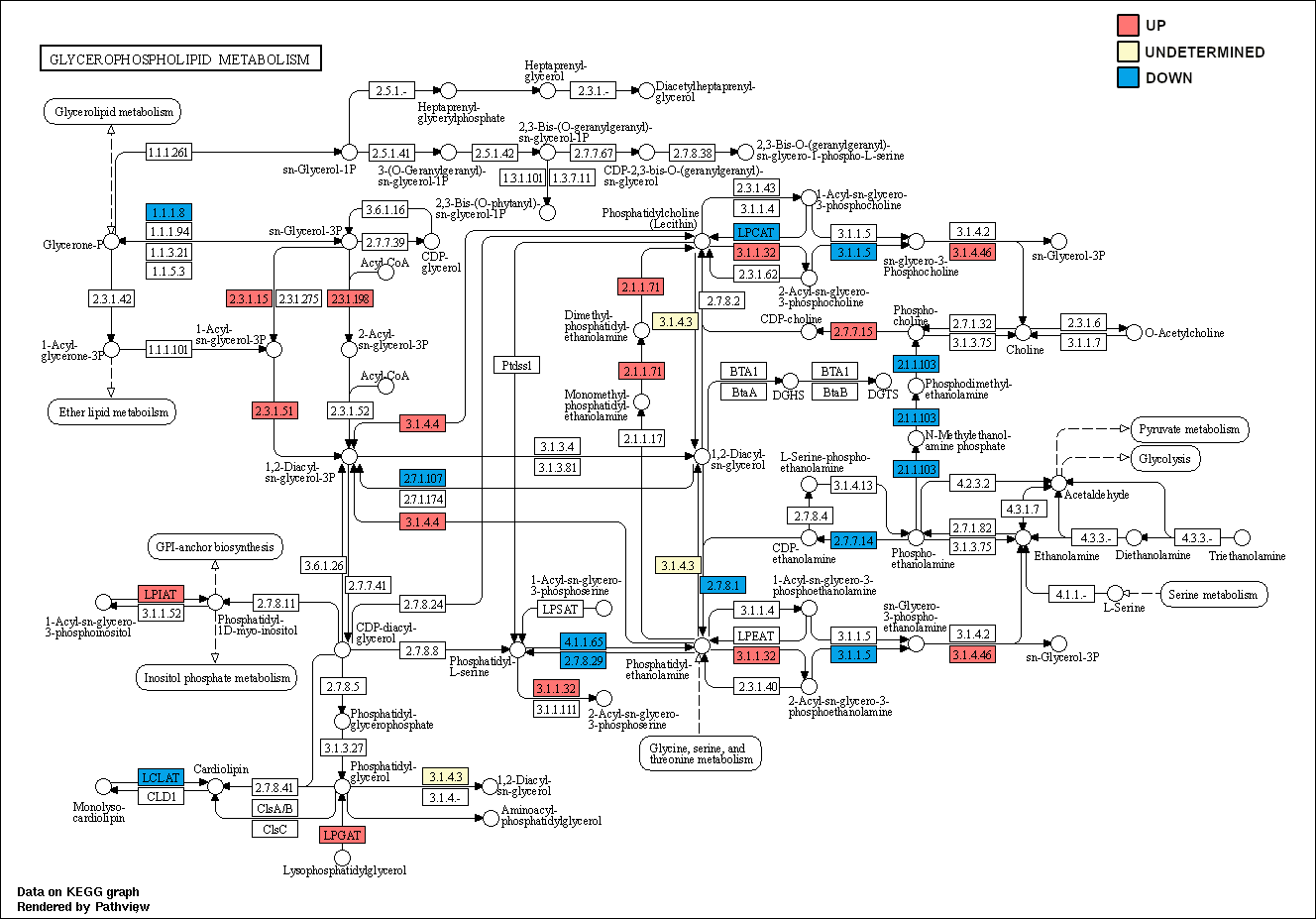

Supplement: Supplementary file 2 [file Data_Sheet_2.ZIP › Supplementary_Figure_4/Supplementary_Figure_4.049.png]

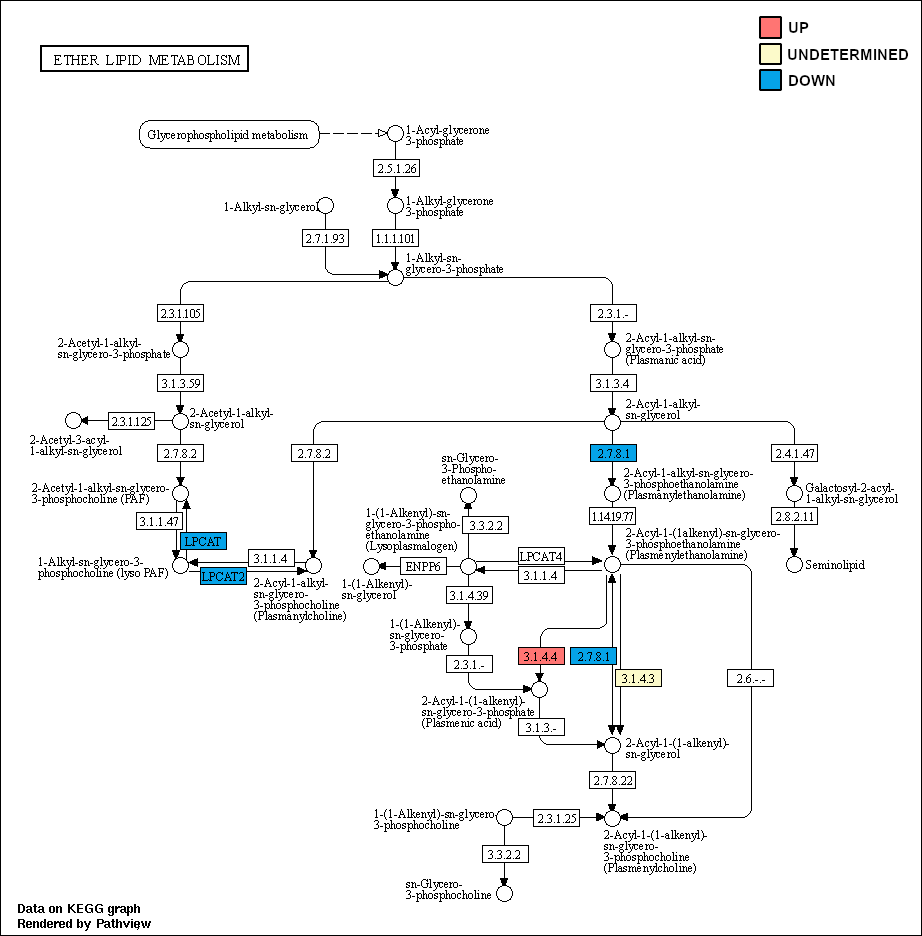

Supplement: Supplementary file 2 [file Data_Sheet_2.ZIP › Supplementary_Figure_4/Supplementary_Figure_4.050.png]

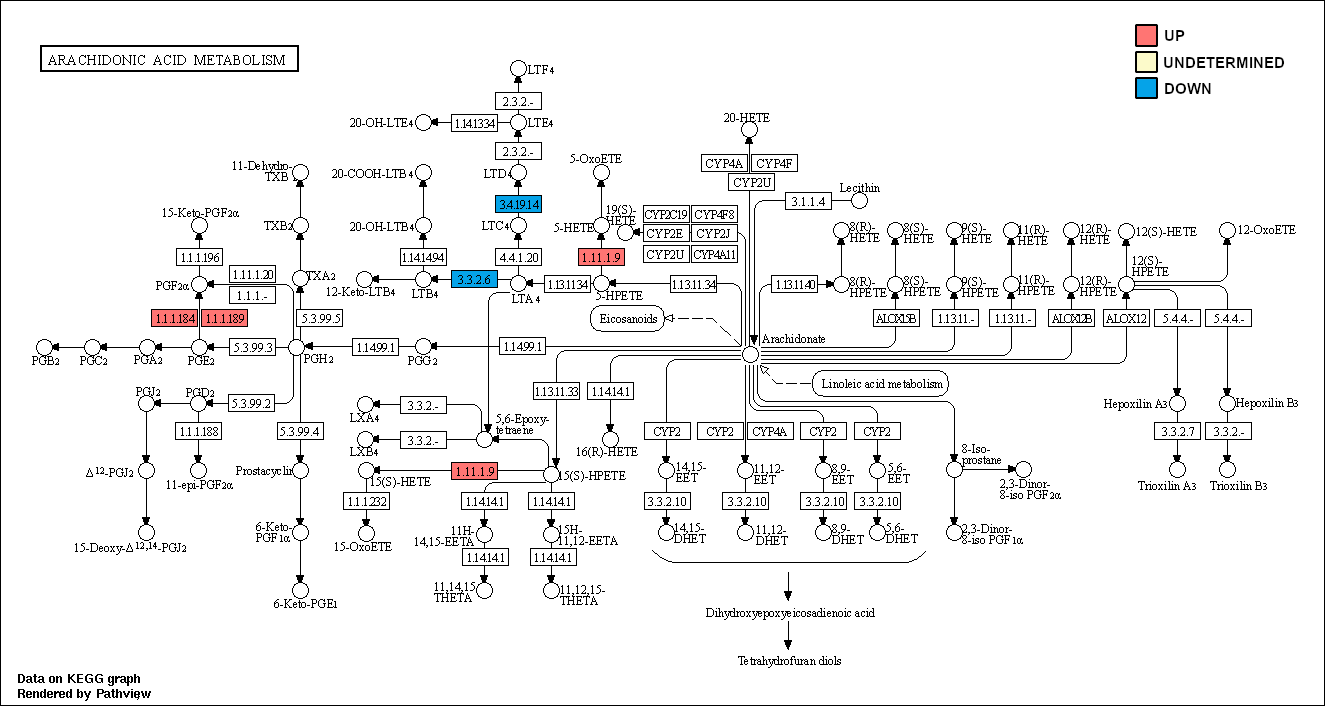

Supplement: Supplementary file 2 [file Data_Sheet_2.ZIP › Supplementary_Figure_4/Supplementary_Figure_4.051.png]

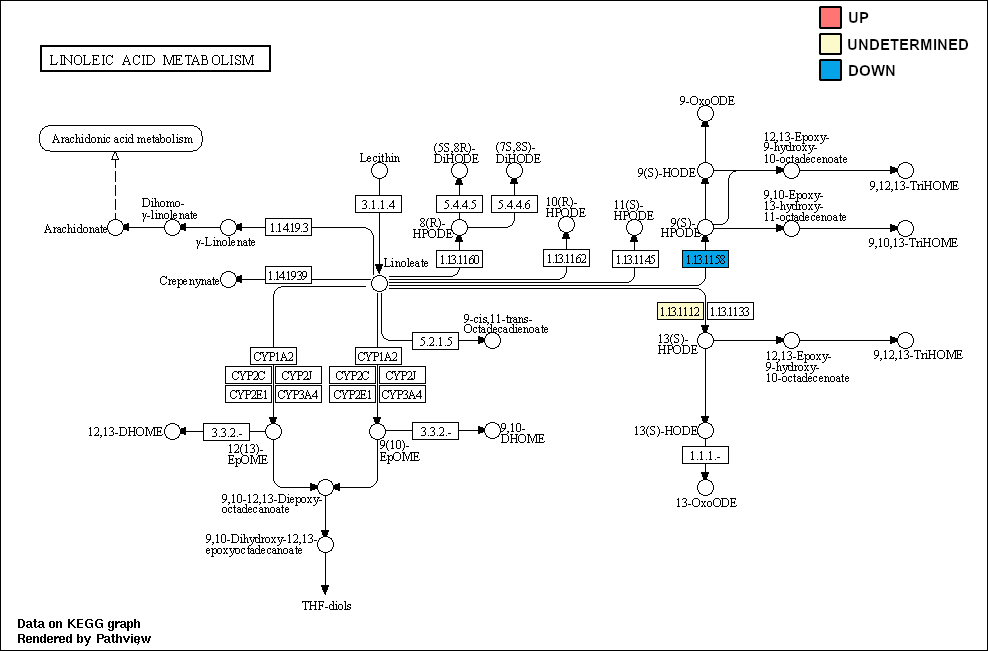

Supplement: Supplementary file 2 [file Data_Sheet_2.ZIP › Supplementary_Figure_4/Supplementary_Figure_4.052.png]

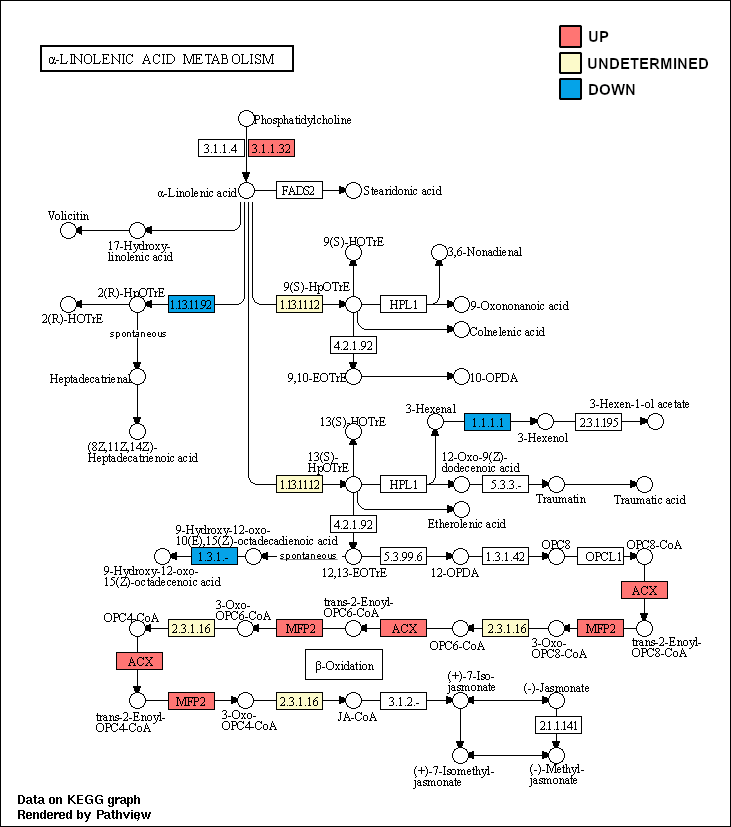

Supplement: Supplementary file 2 [file Data_Sheet_2.ZIP › Supplementary_Figure_4/Supplementary_Figure_4.053.png]

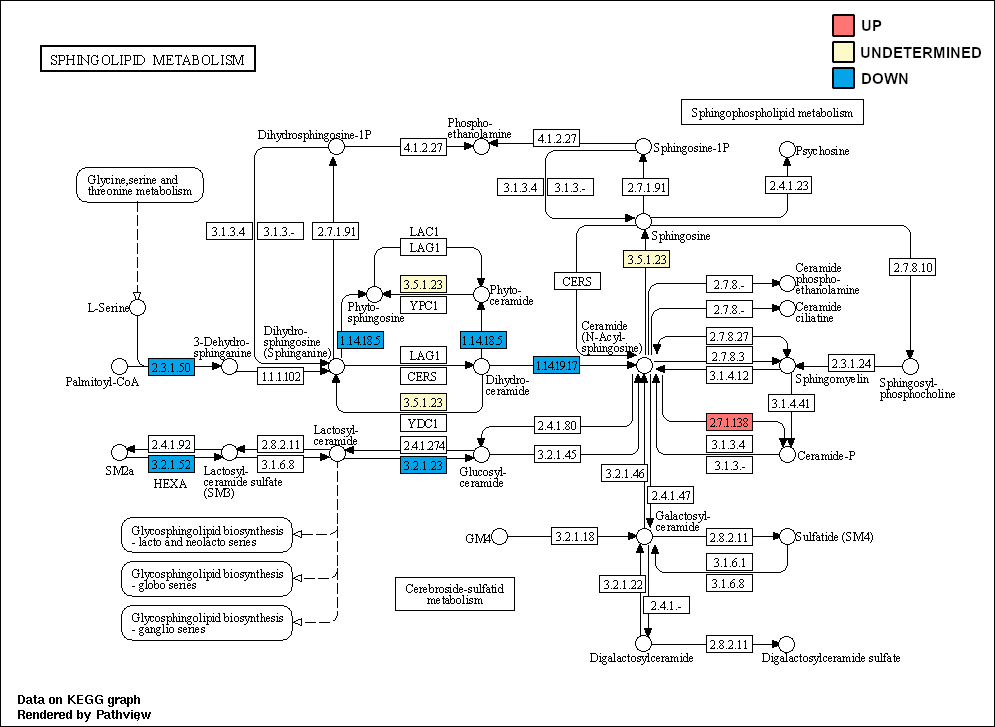

Supplement: Supplementary file 2 [file Data_Sheet_2.ZIP › Supplementary_Figure_4/Supplementary_Figure_4.054.png]

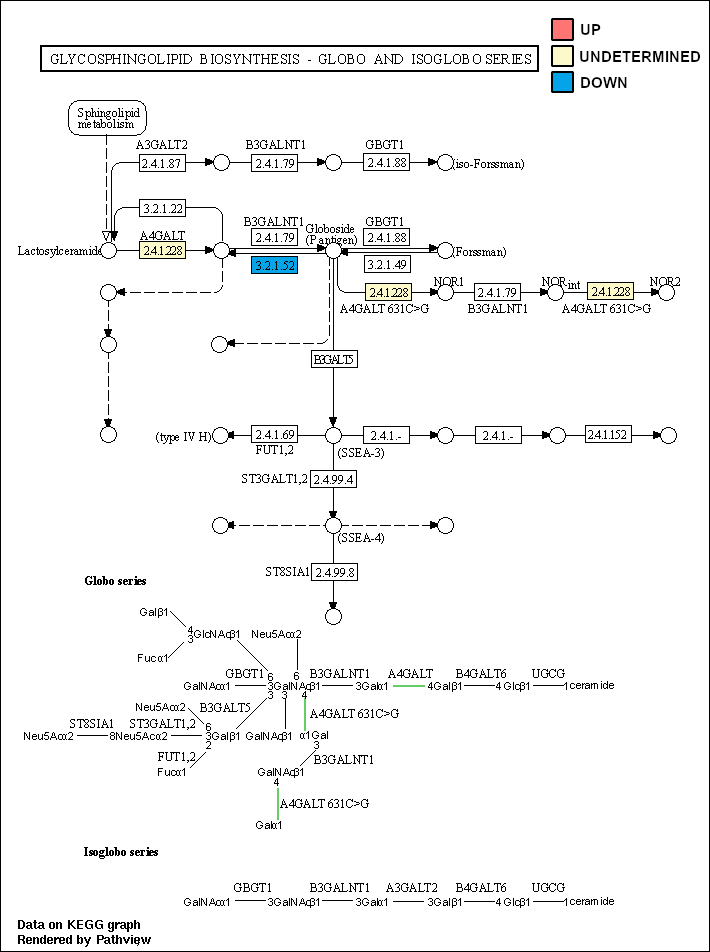

Supplement: Supplementary file 2 [file Data_Sheet_2.ZIP › Supplementary_Figure_4/Supplementary_Figure_4.055.png]

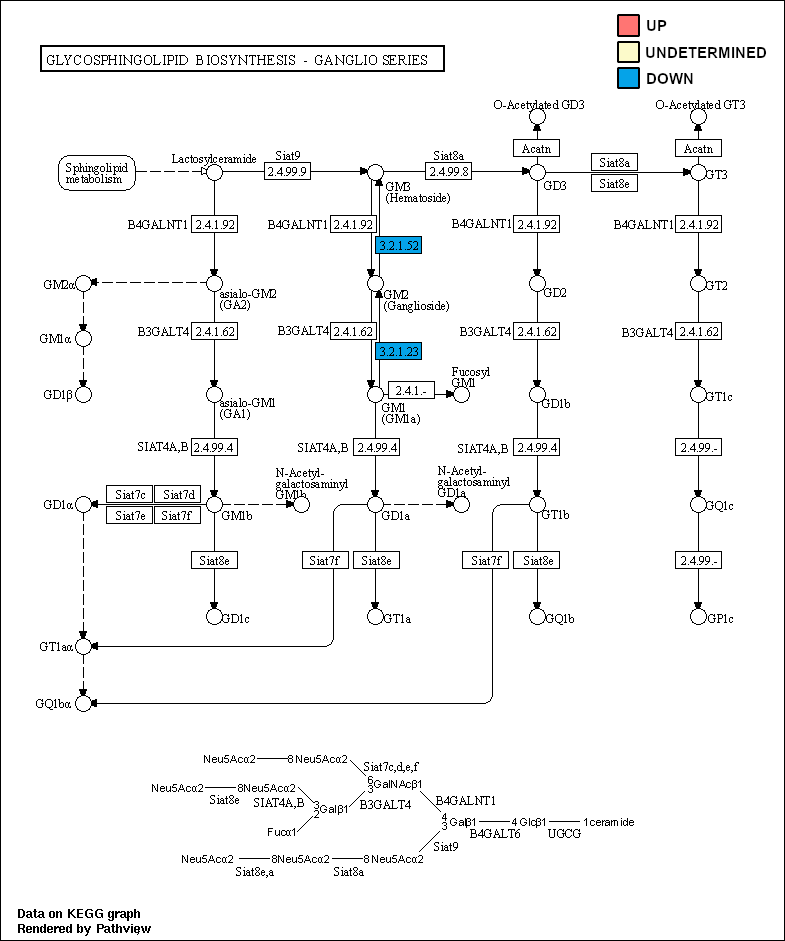

Supplement: Supplementary file 2 [file Data_Sheet_2.ZIP › Supplementary_Figure_4/Supplementary_Figure_4.056.png]

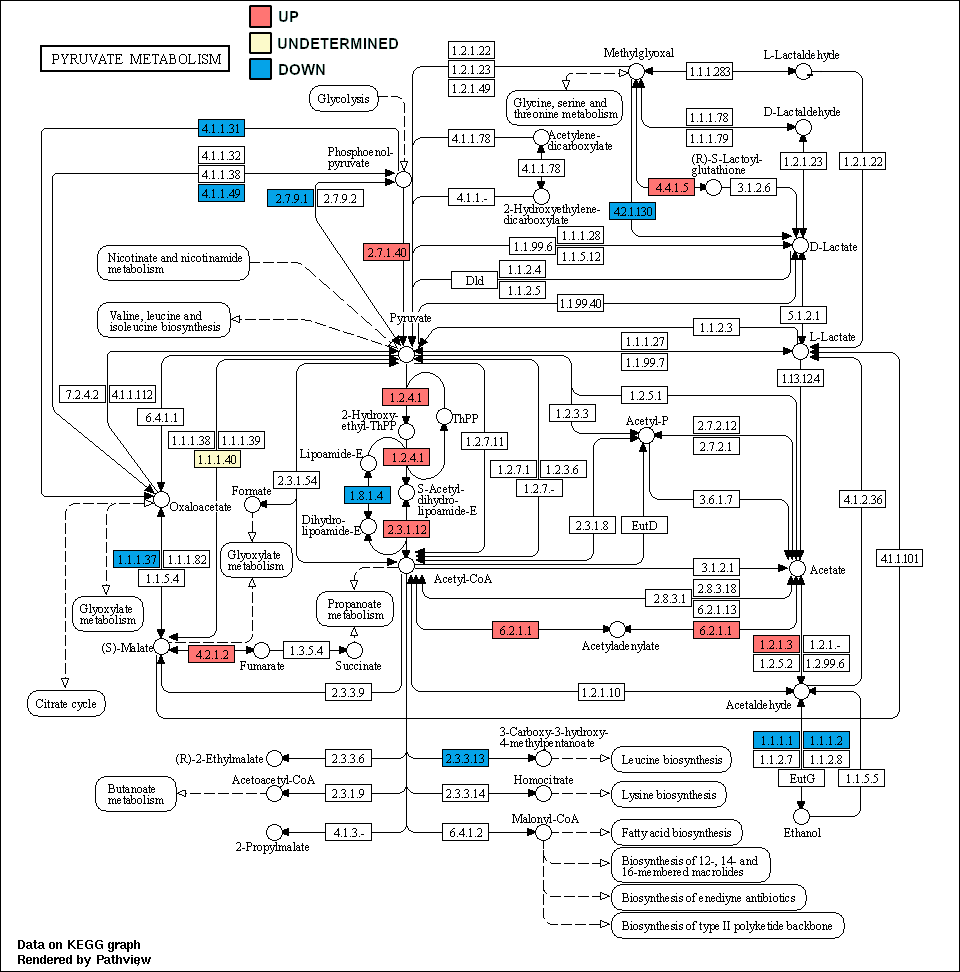

Supplement: Supplementary file 2 [file Data_Sheet_2.ZIP › Supplementary_Figure_4/Supplementary_Figure_4.057.png]

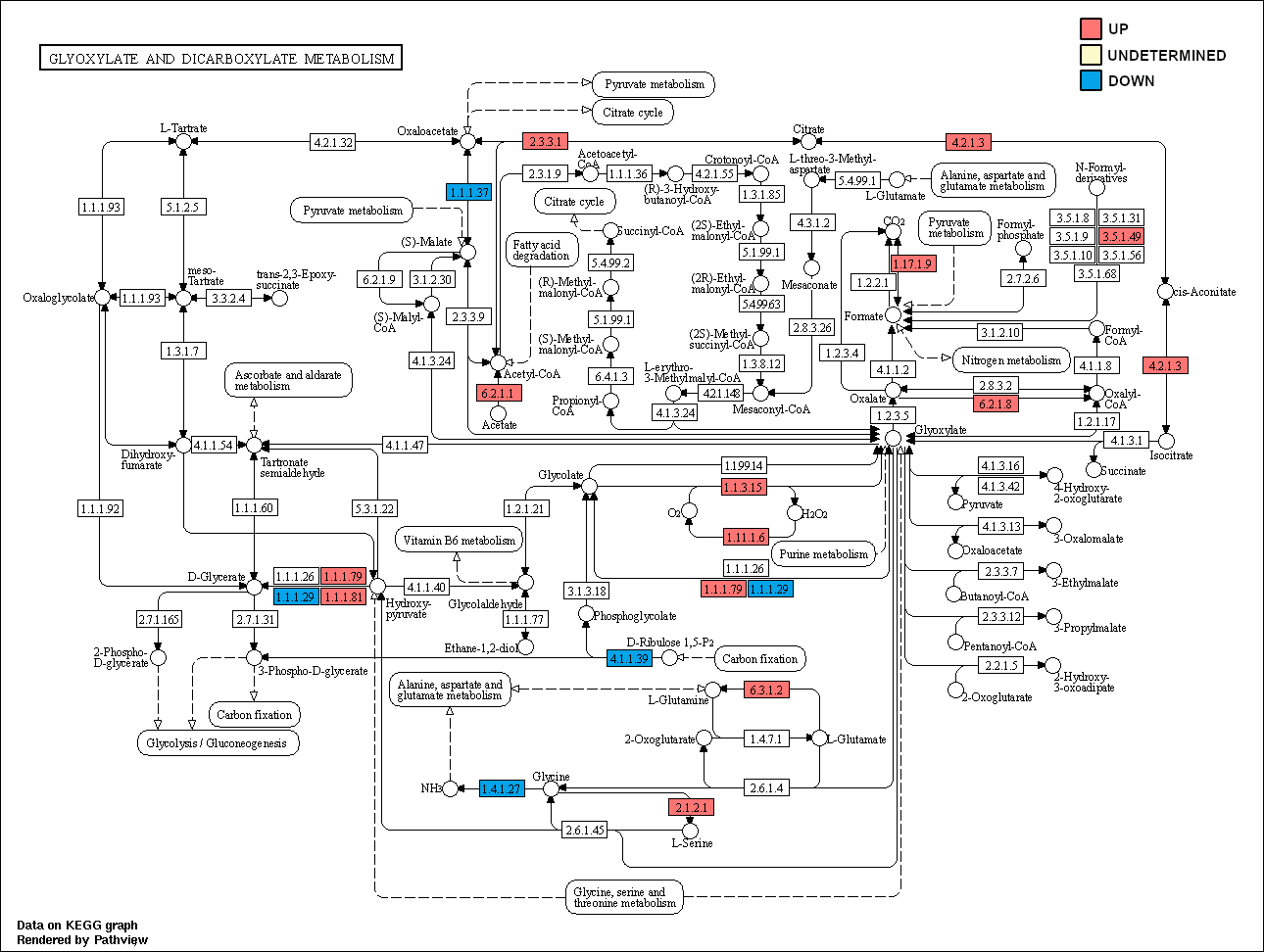

Supplement: Supplementary file 2 [file Data_Sheet_2.ZIP › Supplementary_Figure_4/Supplementary_Figure_4.058.png]

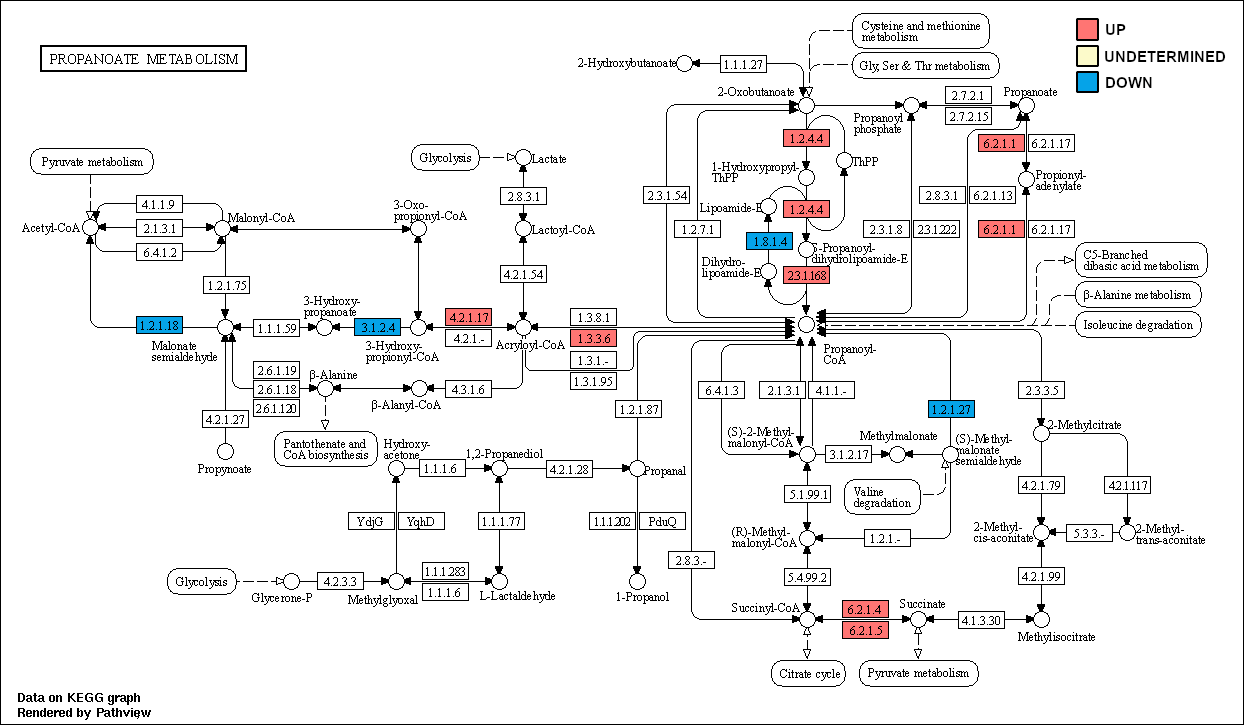

Supplement: Supplementary file 2 [file Data_Sheet_2.ZIP › Supplementary_Figure_4/Supplementary_Figure_4.059.png]

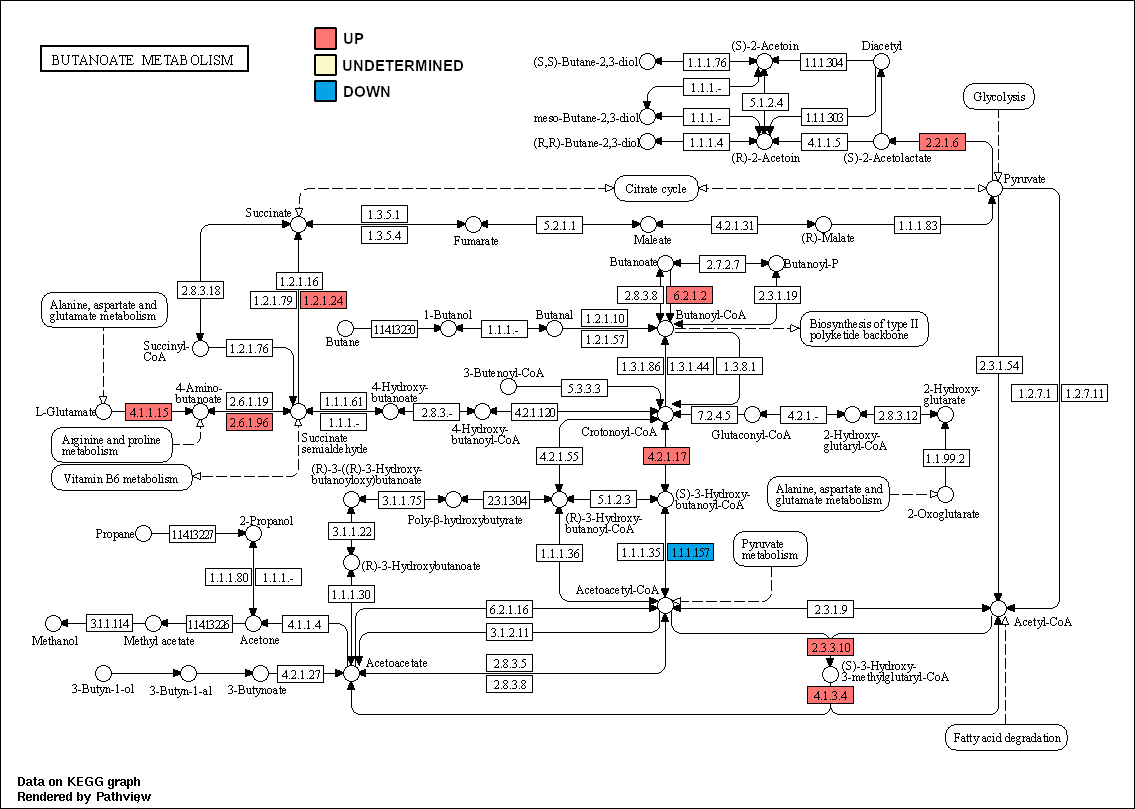

Supplement: Supplementary file 2 [file Data_Sheet_2.ZIP › Supplementary_Figure_4/Supplementary_Figure_4.060.png]

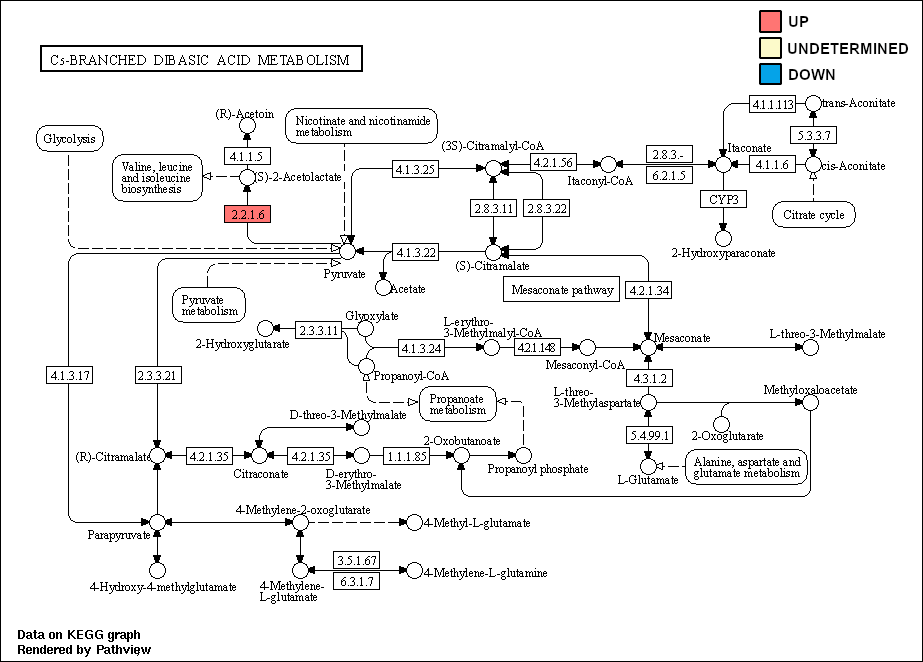

Supplement: Supplementary file 2 [file Data_Sheet_2.ZIP › Supplementary_Figure_4/Supplementary_Figure_4.061.png]

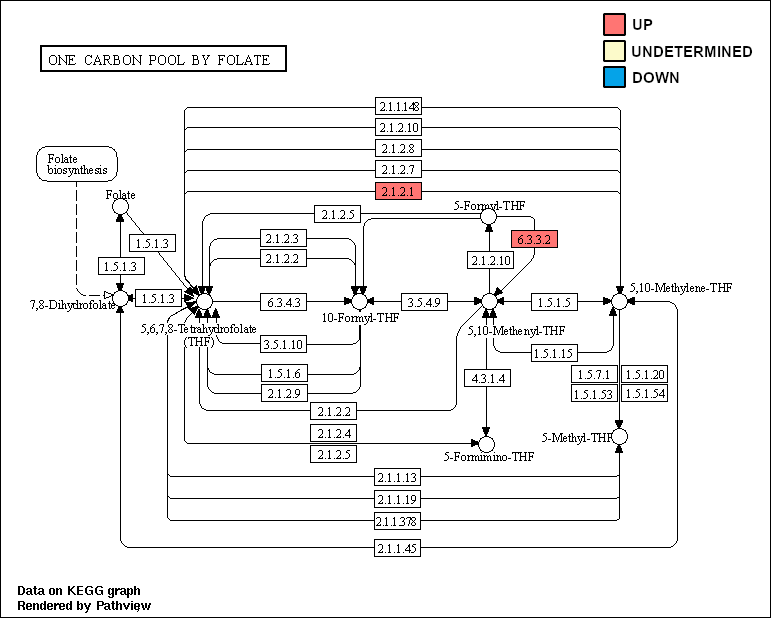

Supplement: Supplementary file 2 [file Data_Sheet_2.ZIP › Supplementary_Figure_4/Supplementary_Figure_4.062.png]

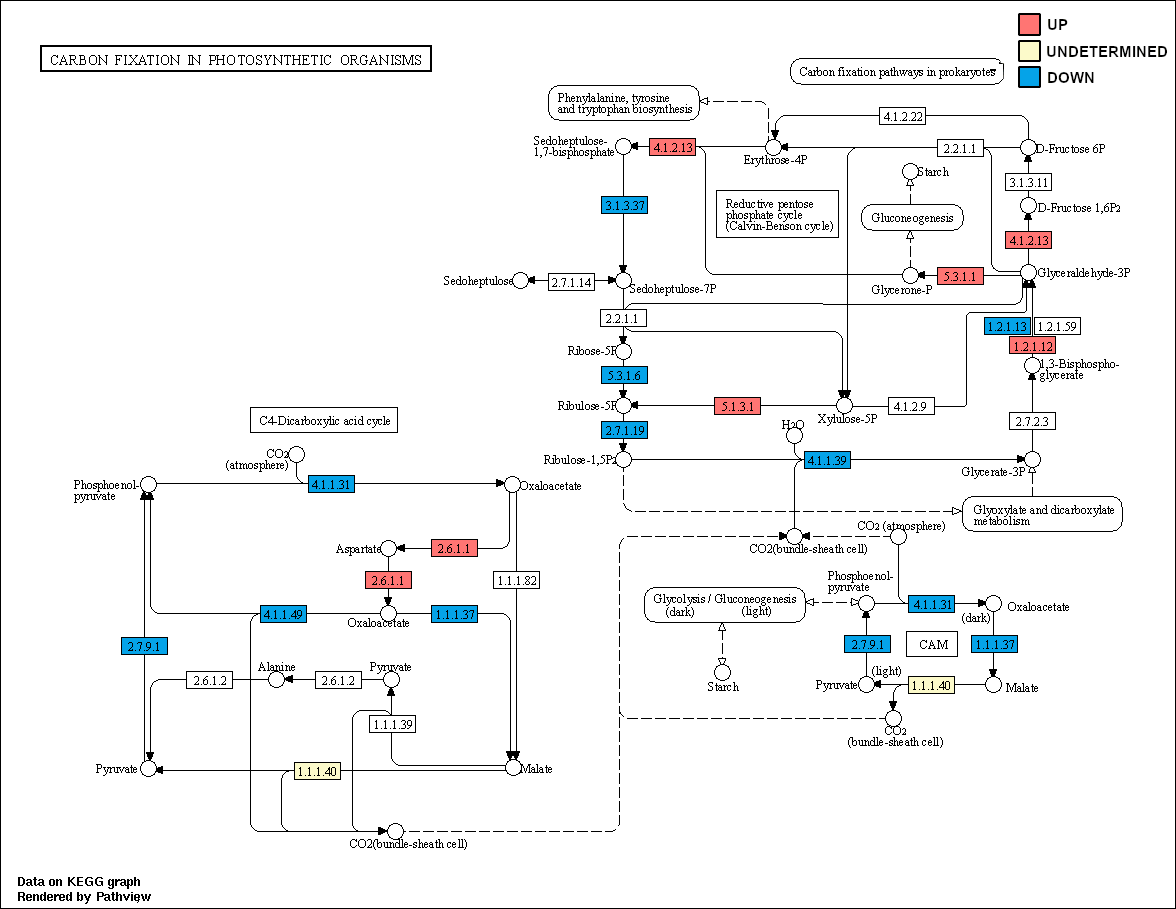

Supplement: Supplementary file 2 [file Data_Sheet_2.ZIP › Supplementary_Figure_4/Supplementary_Figure_4.063.png]

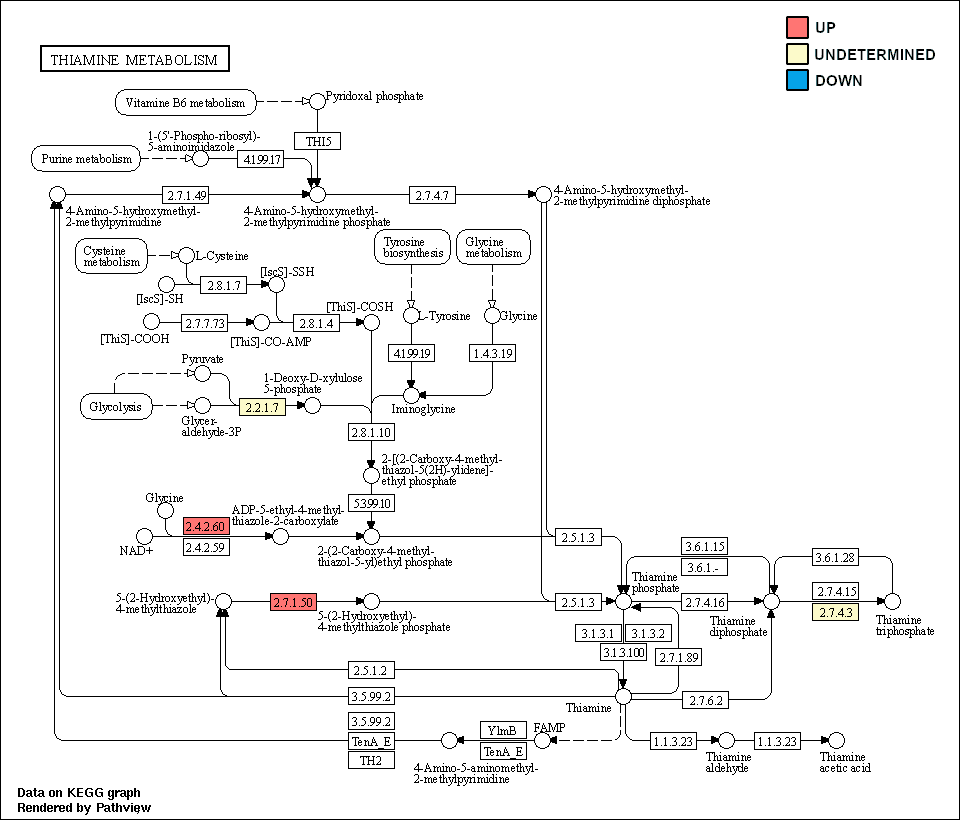

Supplement: Supplementary file 2 [file Data_Sheet_2.ZIP › Supplementary_Figure_4/Supplementary_Figure_4.064.png]

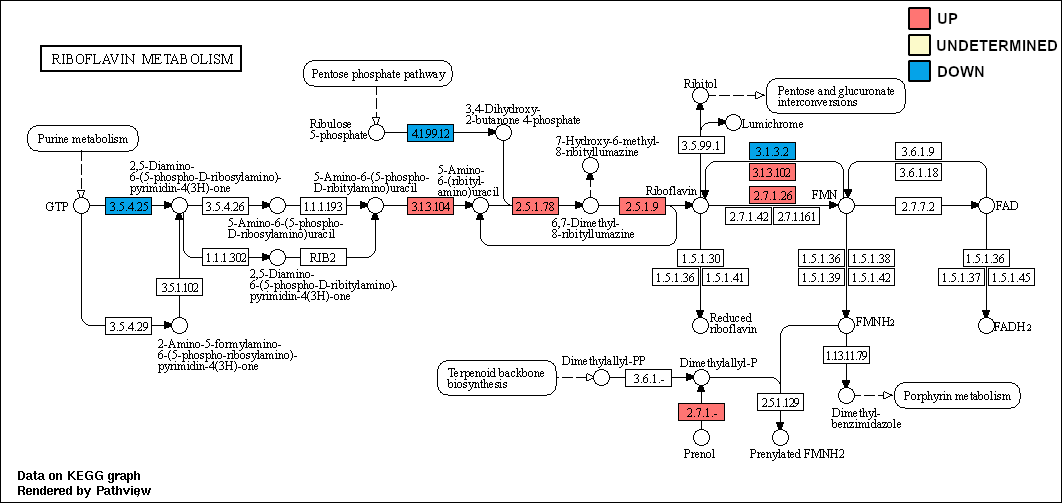

Supplement: Supplementary file 2 [file Data_Sheet_2.ZIP › Supplementary_Figure_4/Supplementary_Figure_4.065.png]

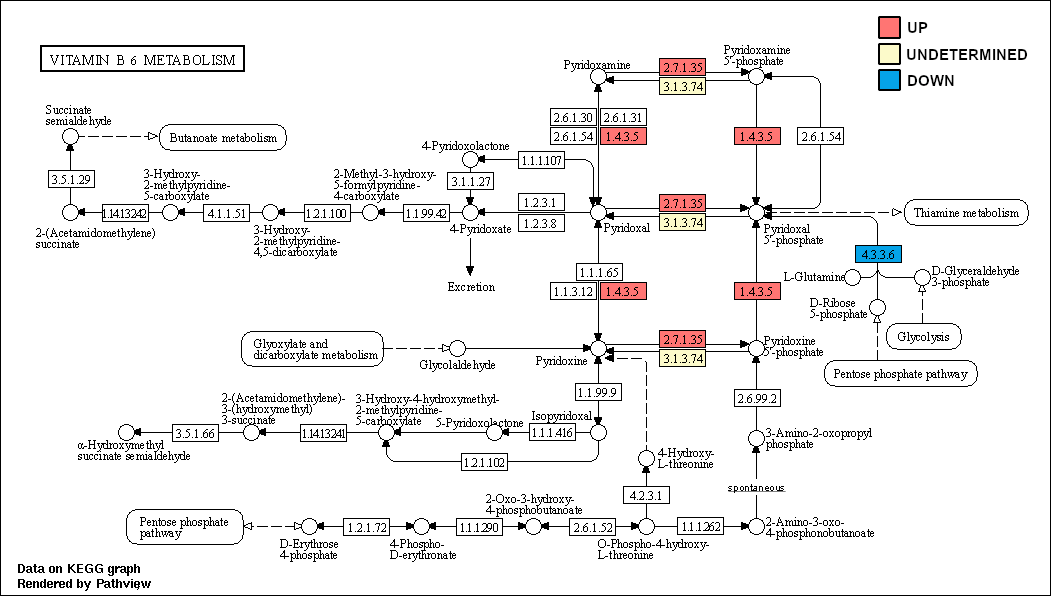

Supplement: Supplementary file 2 [file Data_Sheet_2.ZIP › Supplementary_Figure_4/Supplementary_Figure_4.066.png]

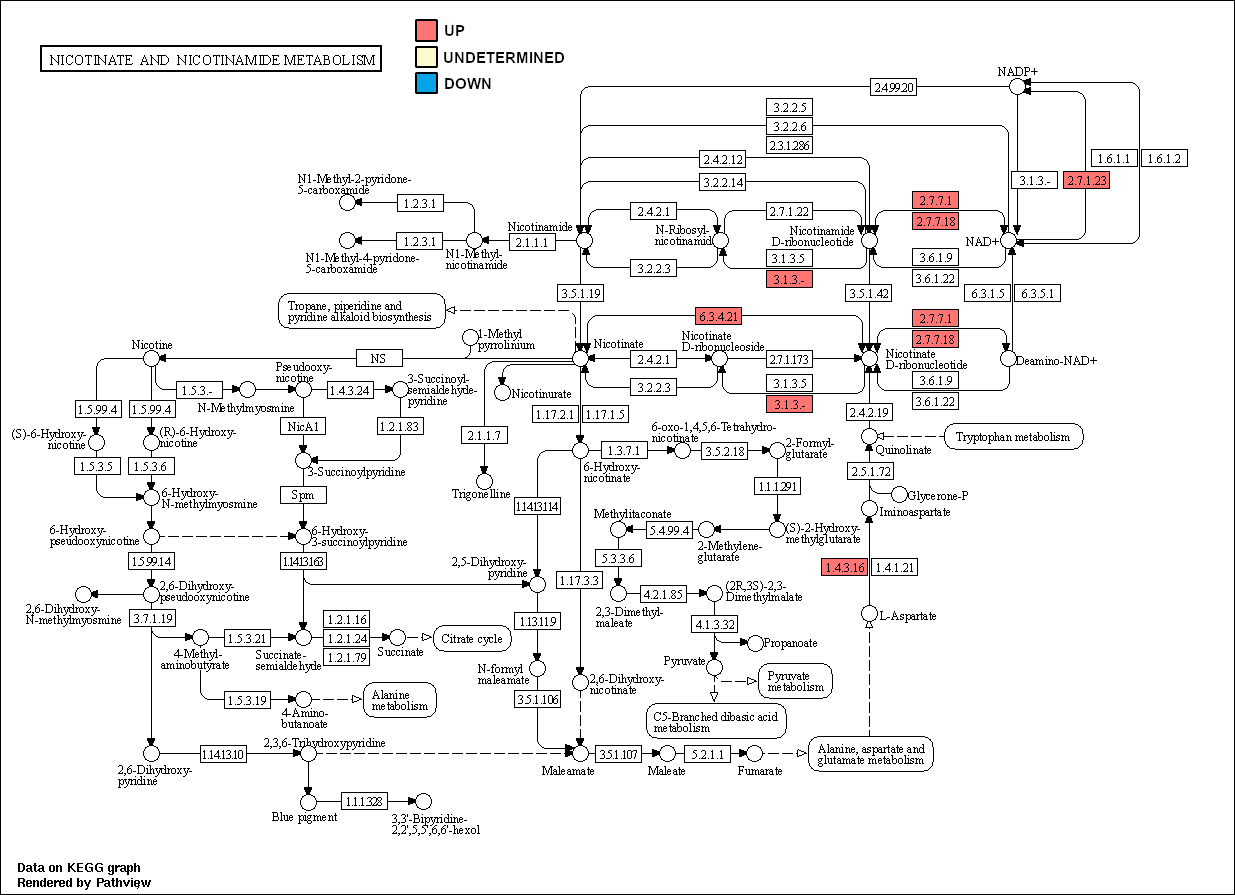

Supplement: Supplementary file 2 [file Data_Sheet_2.ZIP › Supplementary_Figure_4/Supplementary_Figure_4.067.png]

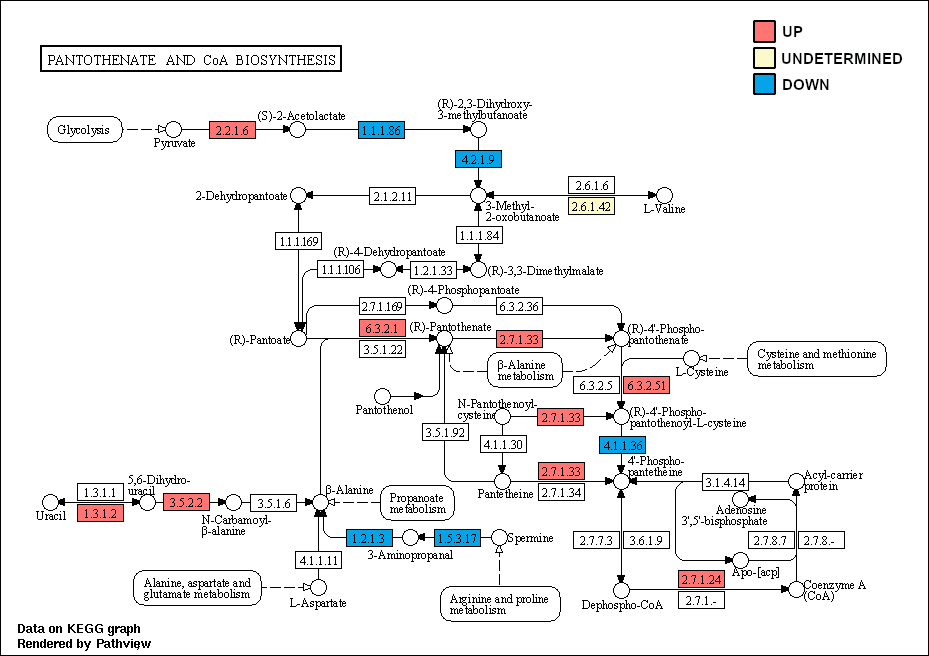

Supplement: Supplementary file 2 [file Data_Sheet_2.ZIP › Supplementary_Figure_4/Supplementary_Figure_4.068.png]

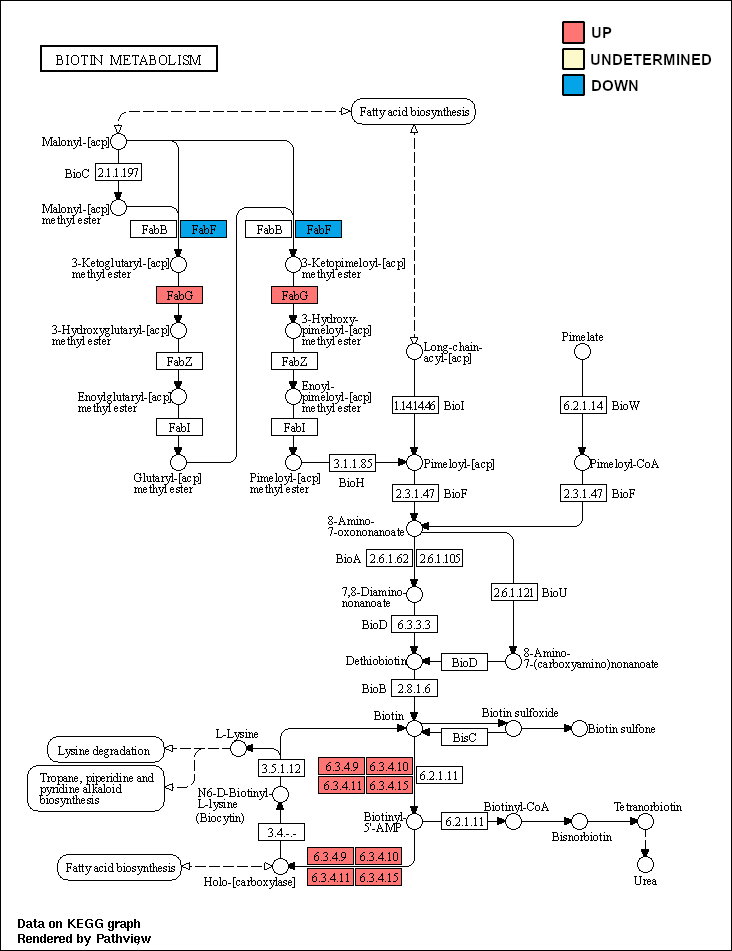

Supplement: Supplementary file 2 [file Data_Sheet_2.ZIP › Supplementary_Figure_4/Supplementary_Figure_4.069.png]

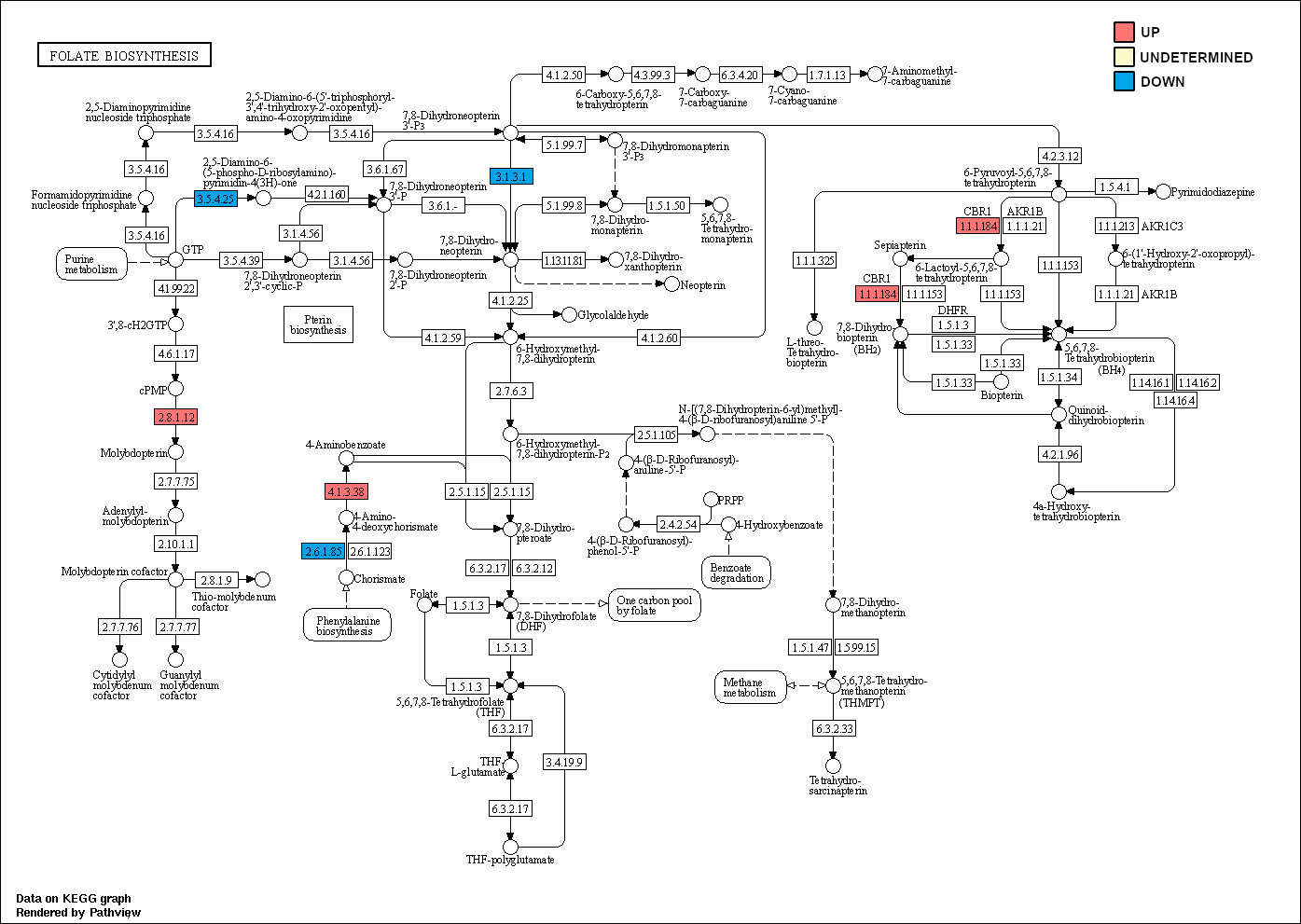

Supplement: Supplementary file 2 [file Data_Sheet_2.ZIP › Supplementary_Figure_4/Supplementary_Figure_4.070.png]

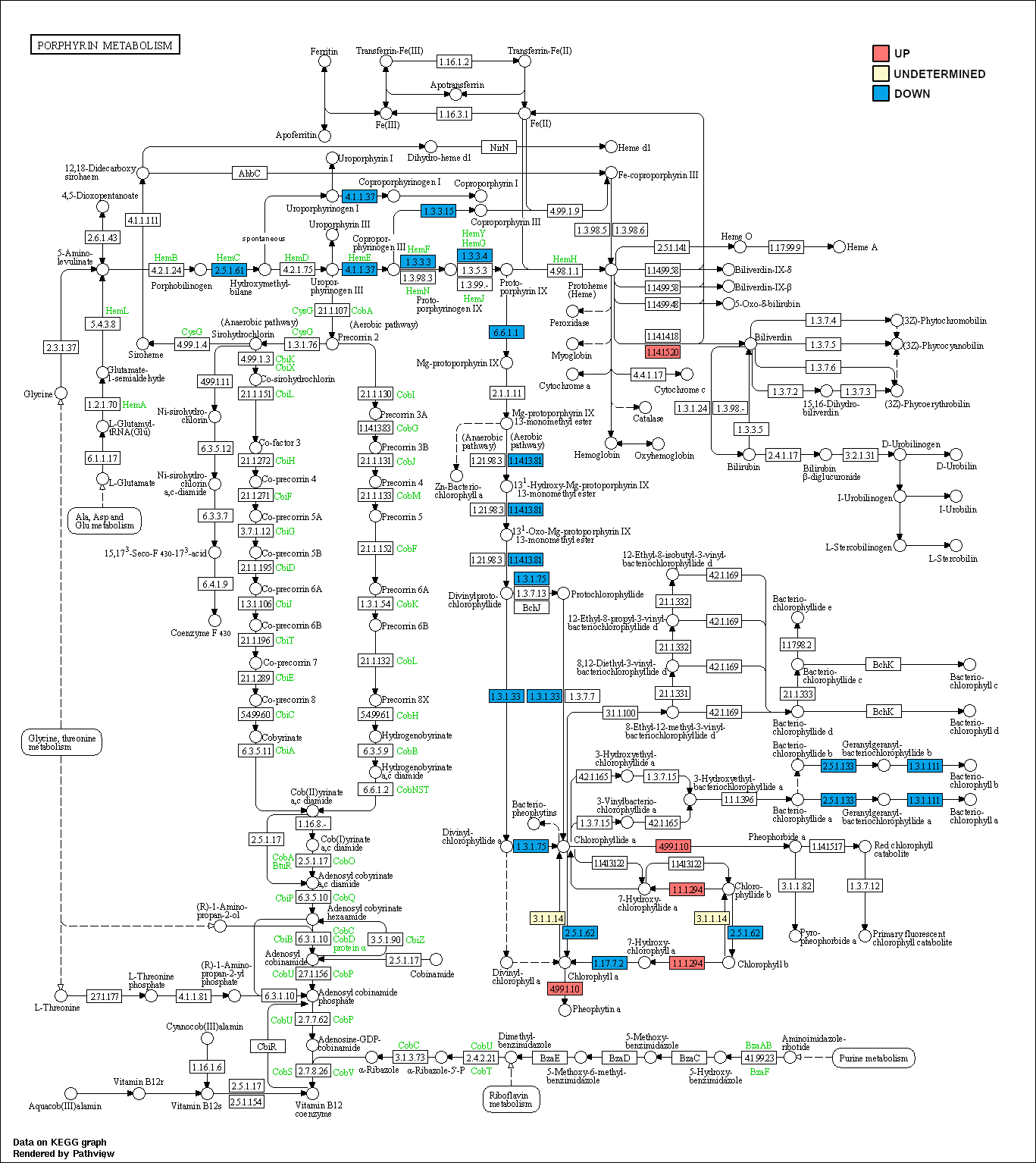

Supplement: Supplementary file 2 [file Data_Sheet_2.ZIP › Supplementary_Figure_4/Supplementary_Figure_4.071.png]

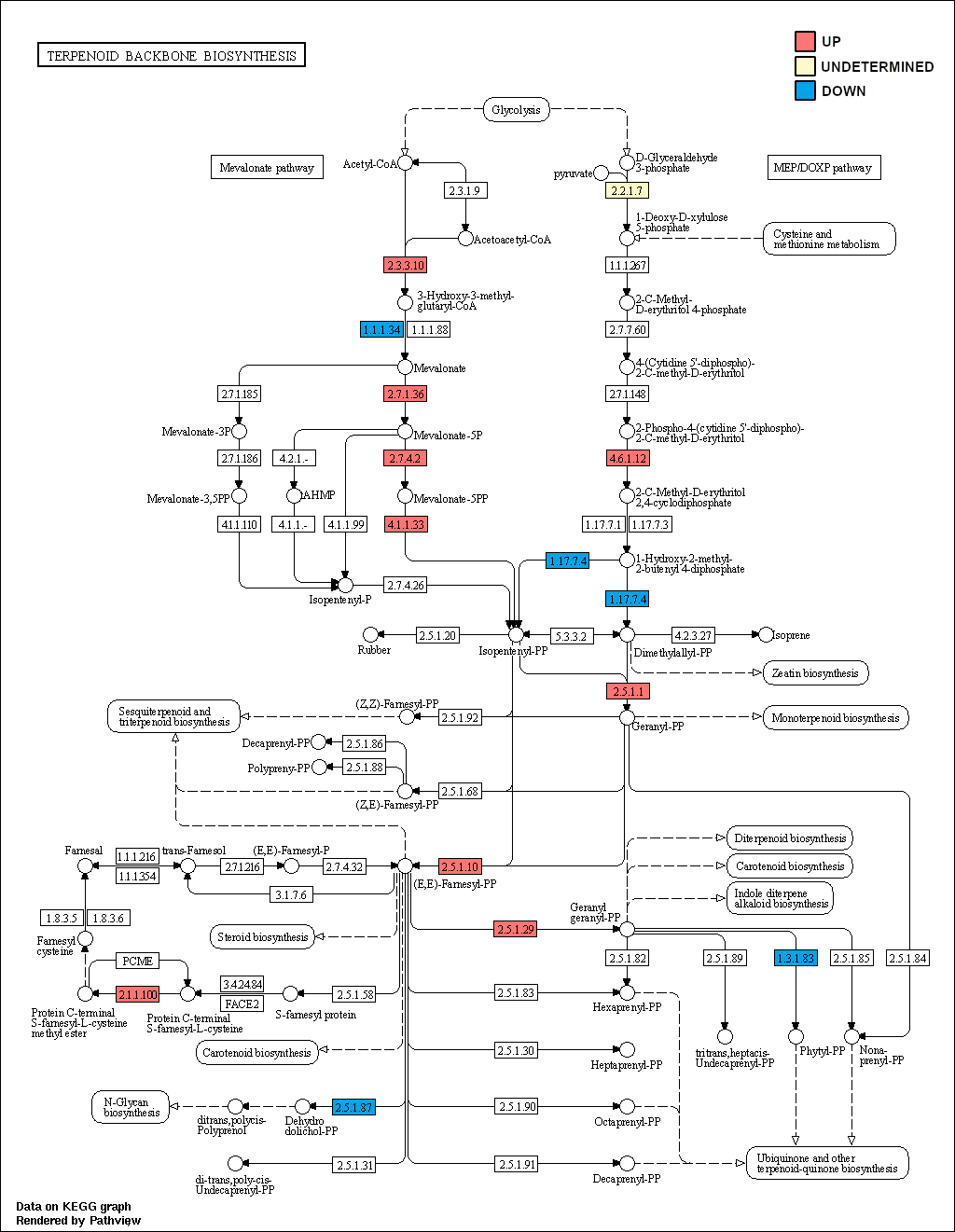

Supplement: Supplementary file 2 [file Data_Sheet_2.ZIP › Supplementary_Figure_4/Supplementary_Figure_4.072.png]

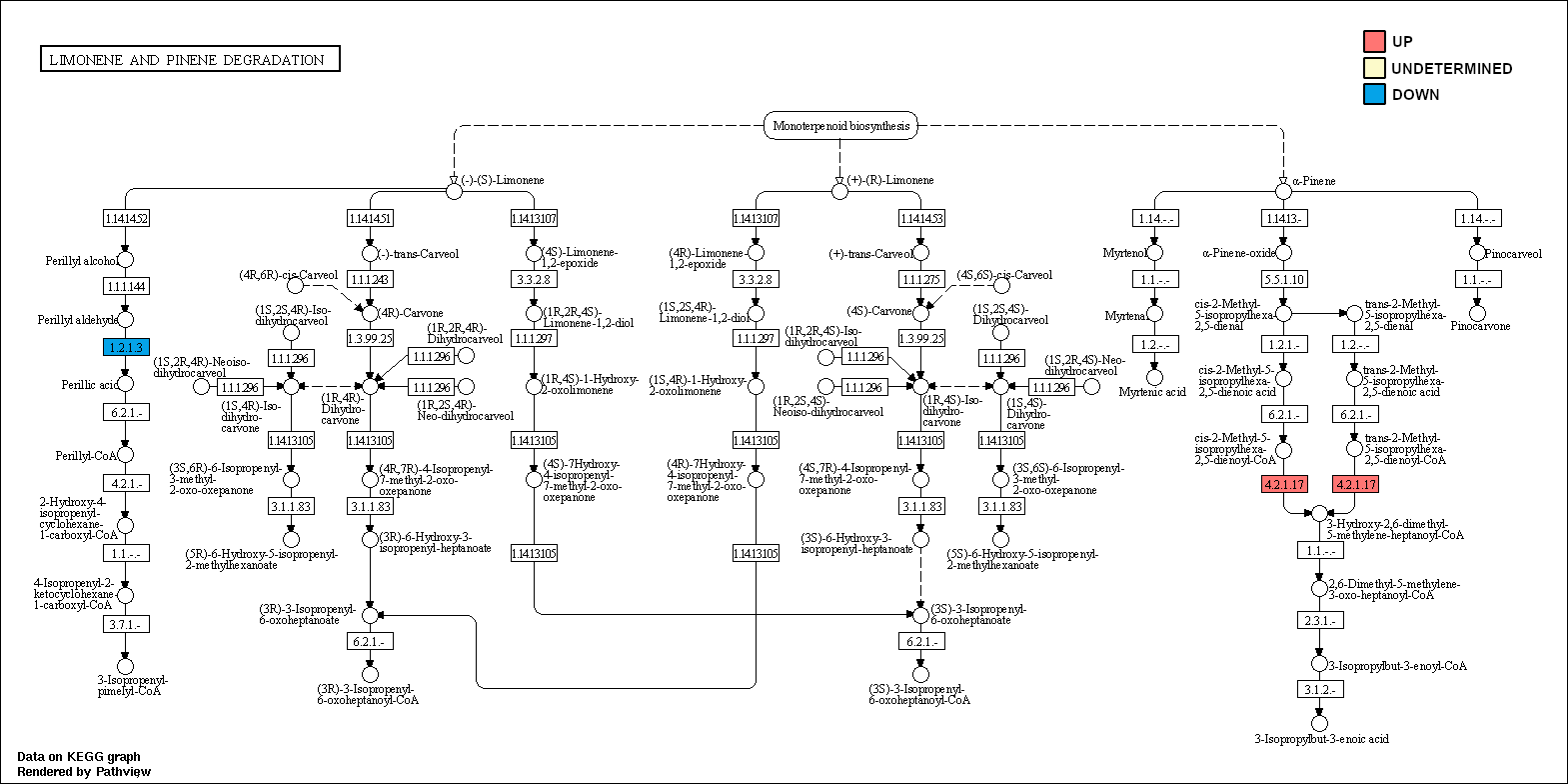

Supplement: Supplementary file 2 [file Data_Sheet_2.ZIP › Supplementary_Figure_4/Supplementary_Figure_4.073.png]

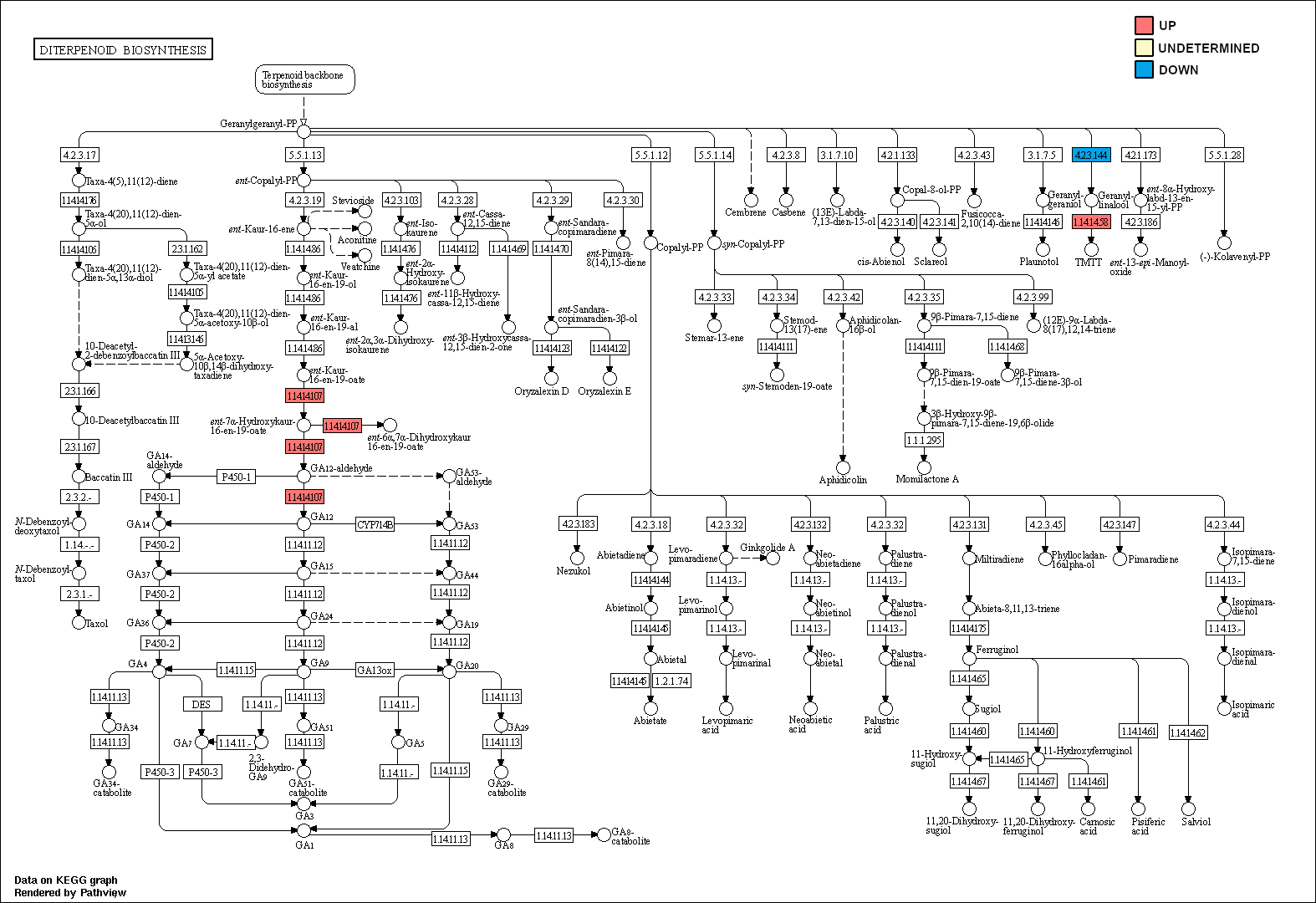

Supplement: Supplementary file 2 [file Data_Sheet_2.ZIP › Supplementary_Figure_4/Supplementary_Figure_4.074.png]

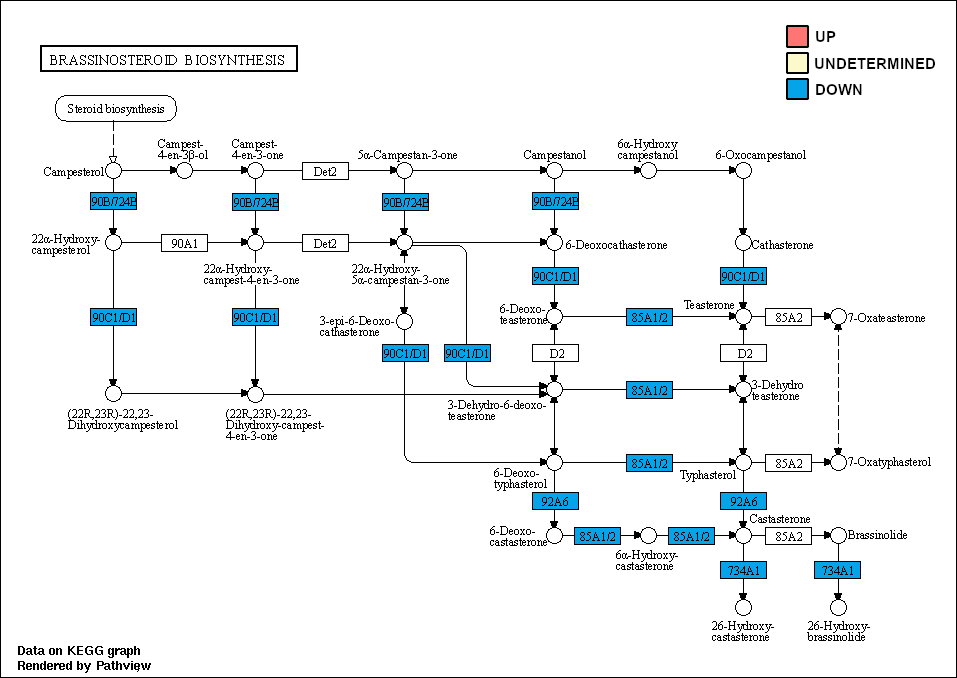

Supplement: Supplementary file 2 [file Data_Sheet_2.ZIP › Supplementary_Figure_4/Supplementary_Figure_4.075.png]

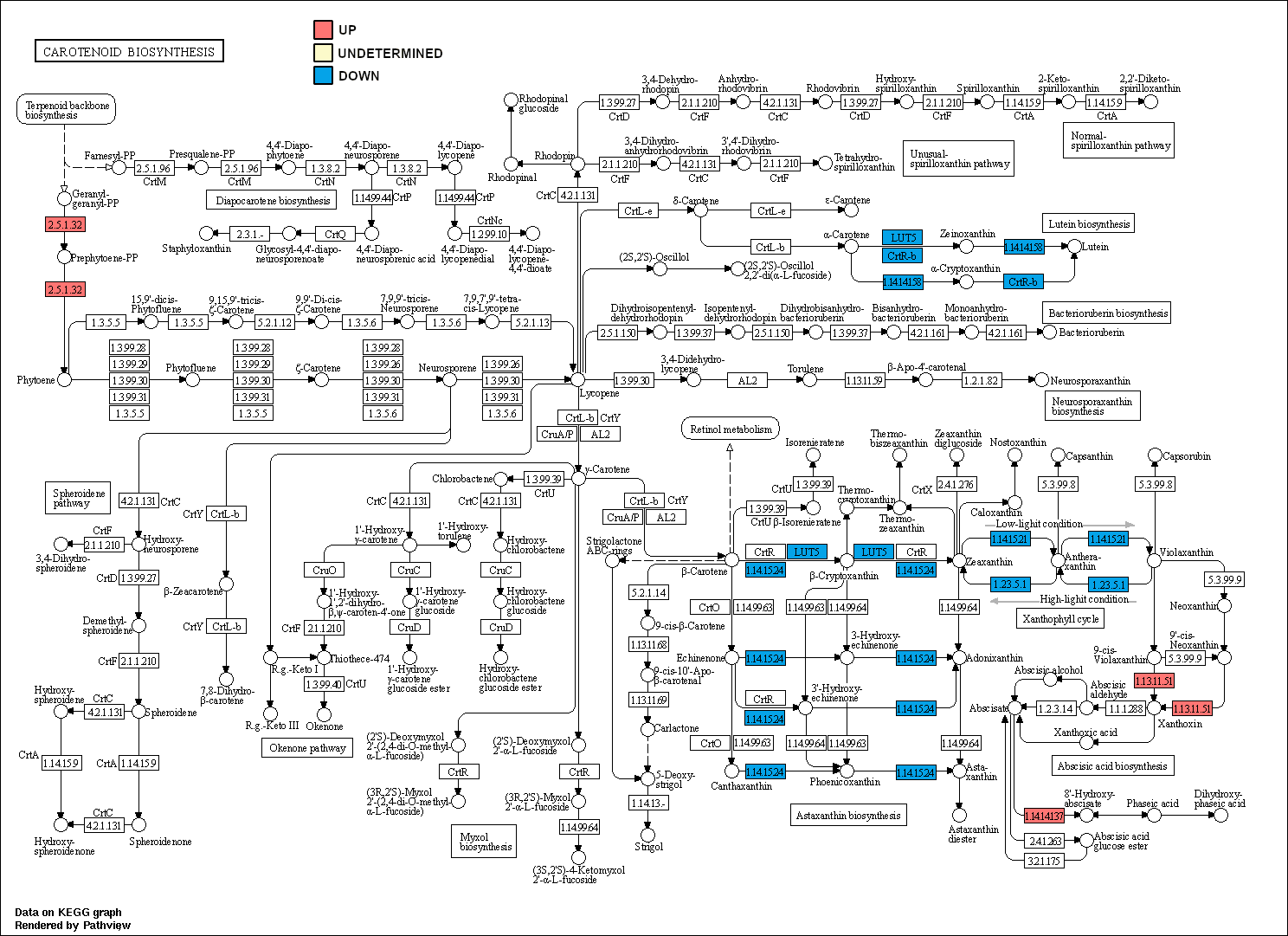

Supplement: Supplementary file 2 [file Data_Sheet_2.ZIP › Supplementary_Figure_4/Supplementary_Figure_4.076.png]

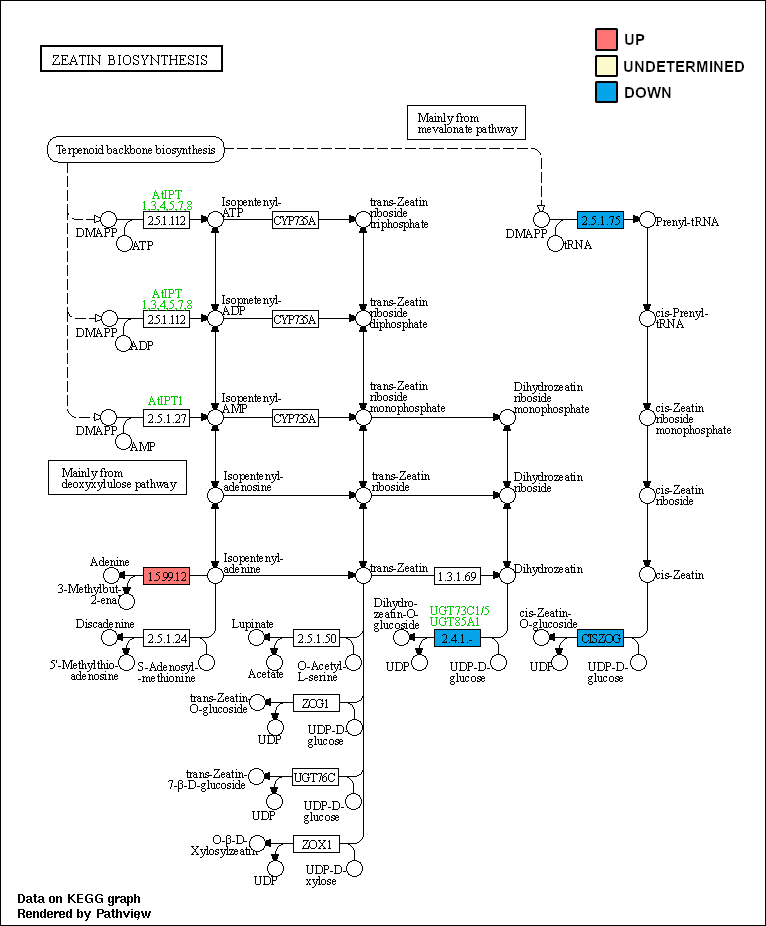

Supplement: Supplementary file 2 [file Data_Sheet_2.ZIP › Supplementary_Figure_4/Supplementary_Figure_4.077.png]

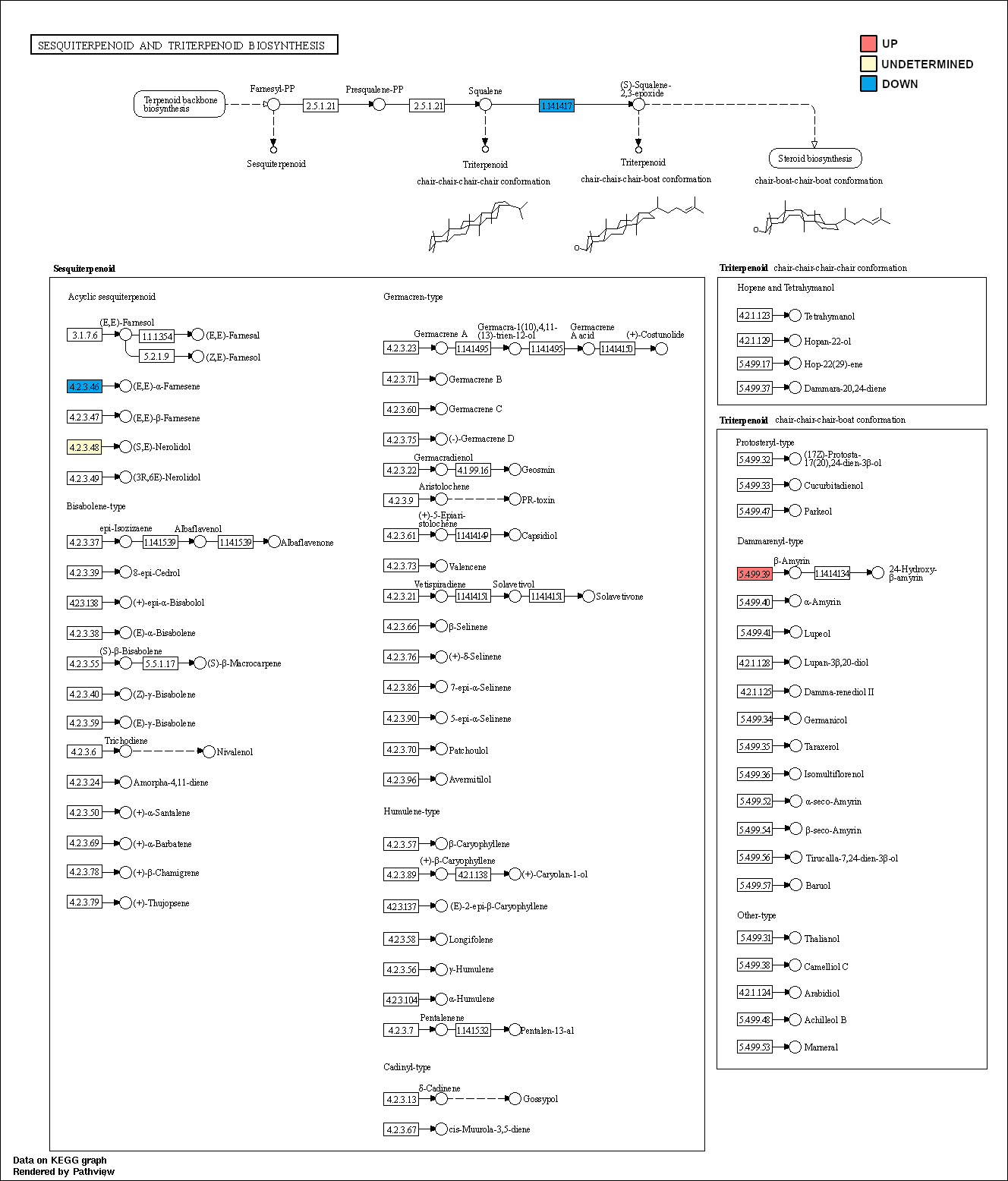

Supplement: Supplementary file 2 [file Data_Sheet_2.ZIP › Supplementary_Figure_4/Supplementary_Figure_4.078.png]

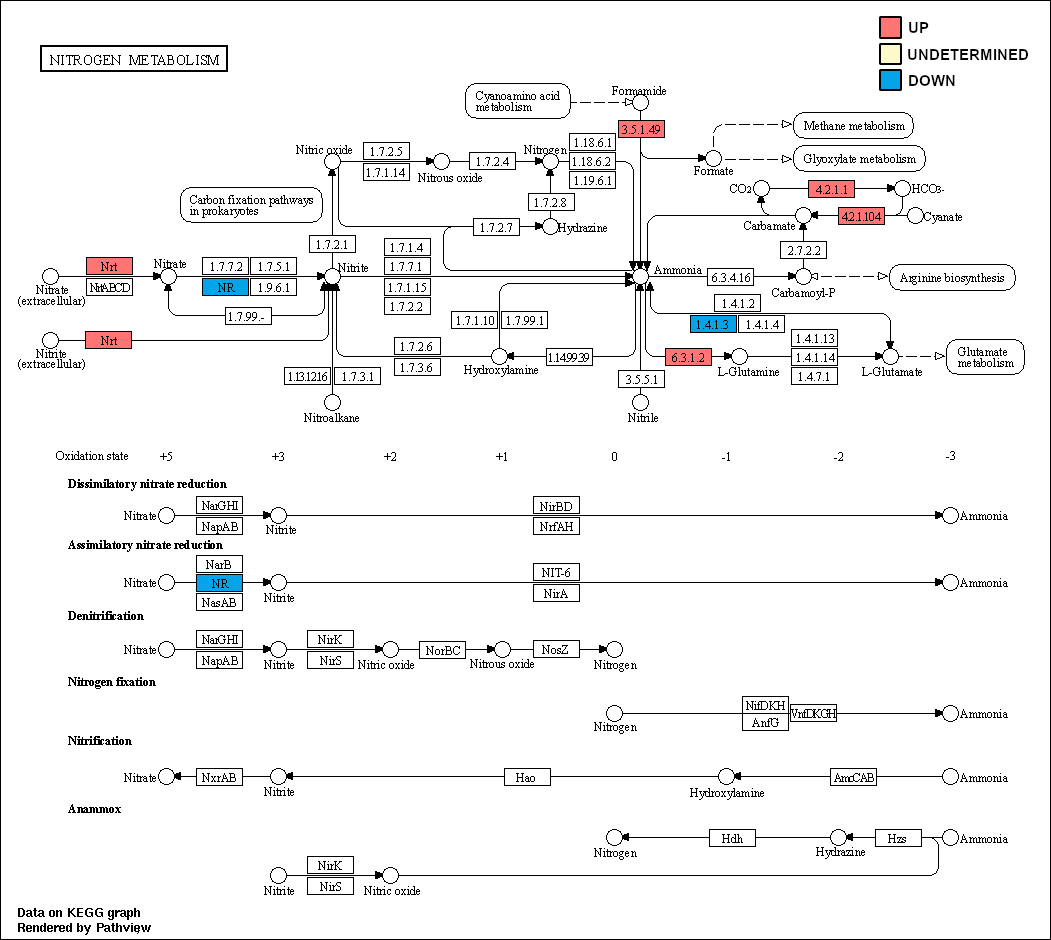

Supplement: Supplementary file 2 [file Data_Sheet_2.ZIP › Supplementary_Figure_4/Supplementary_Figure_4.079.png]

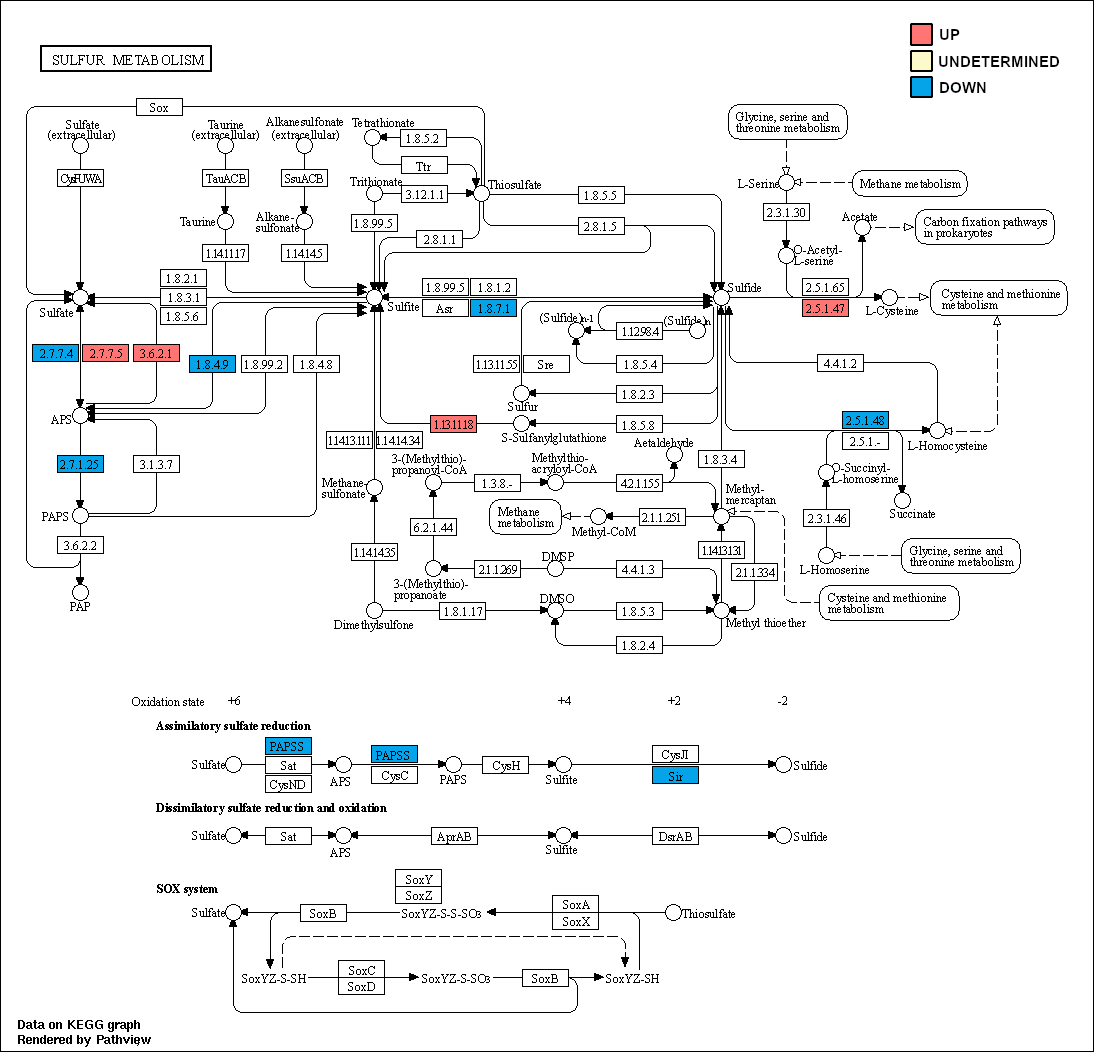

Supplement: Supplementary file 2 [file Data_Sheet_2.ZIP › Supplementary_Figure_4/Supplementary_Figure_4.080.png]

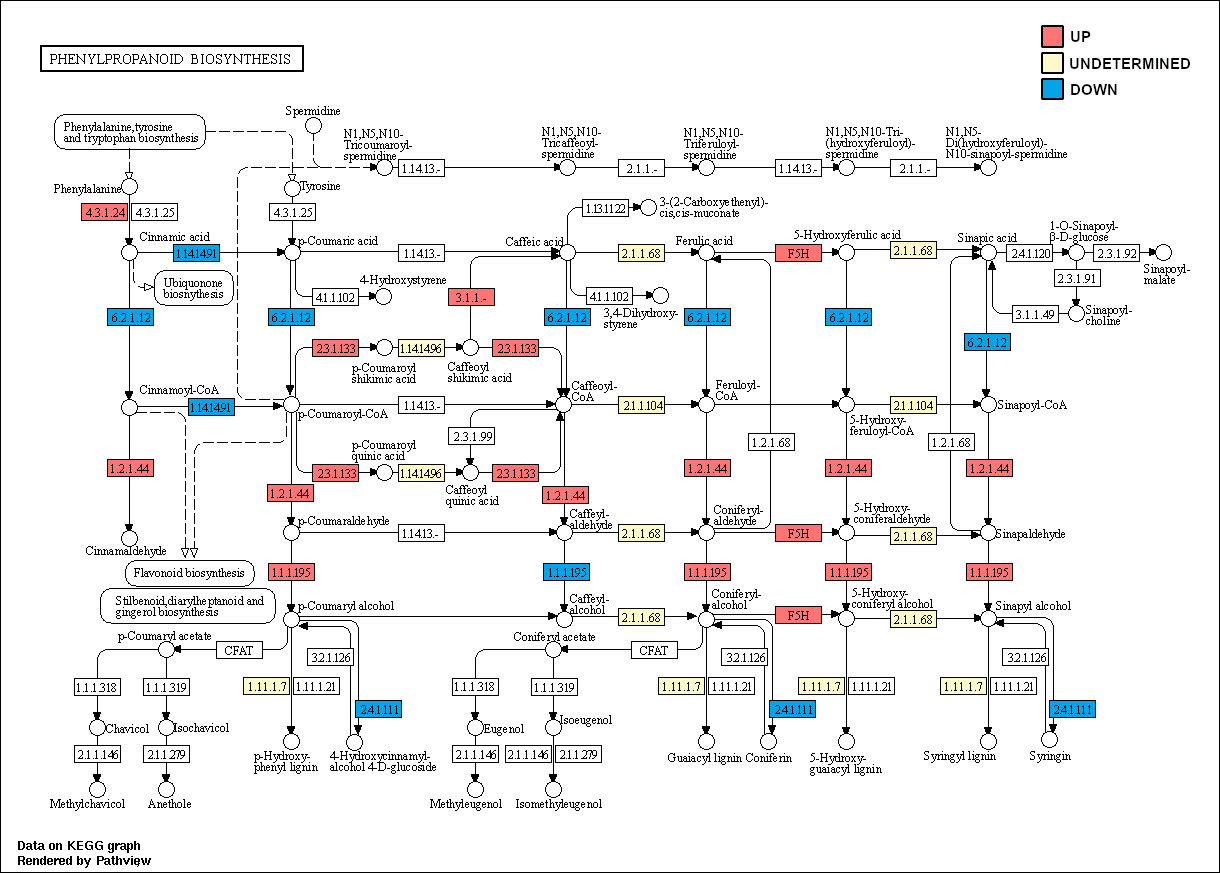

Supplement: Supplementary file 2 [file Data_Sheet_2.ZIP › Supplementary_Figure_4/Supplementary_Figure_4.081.png]

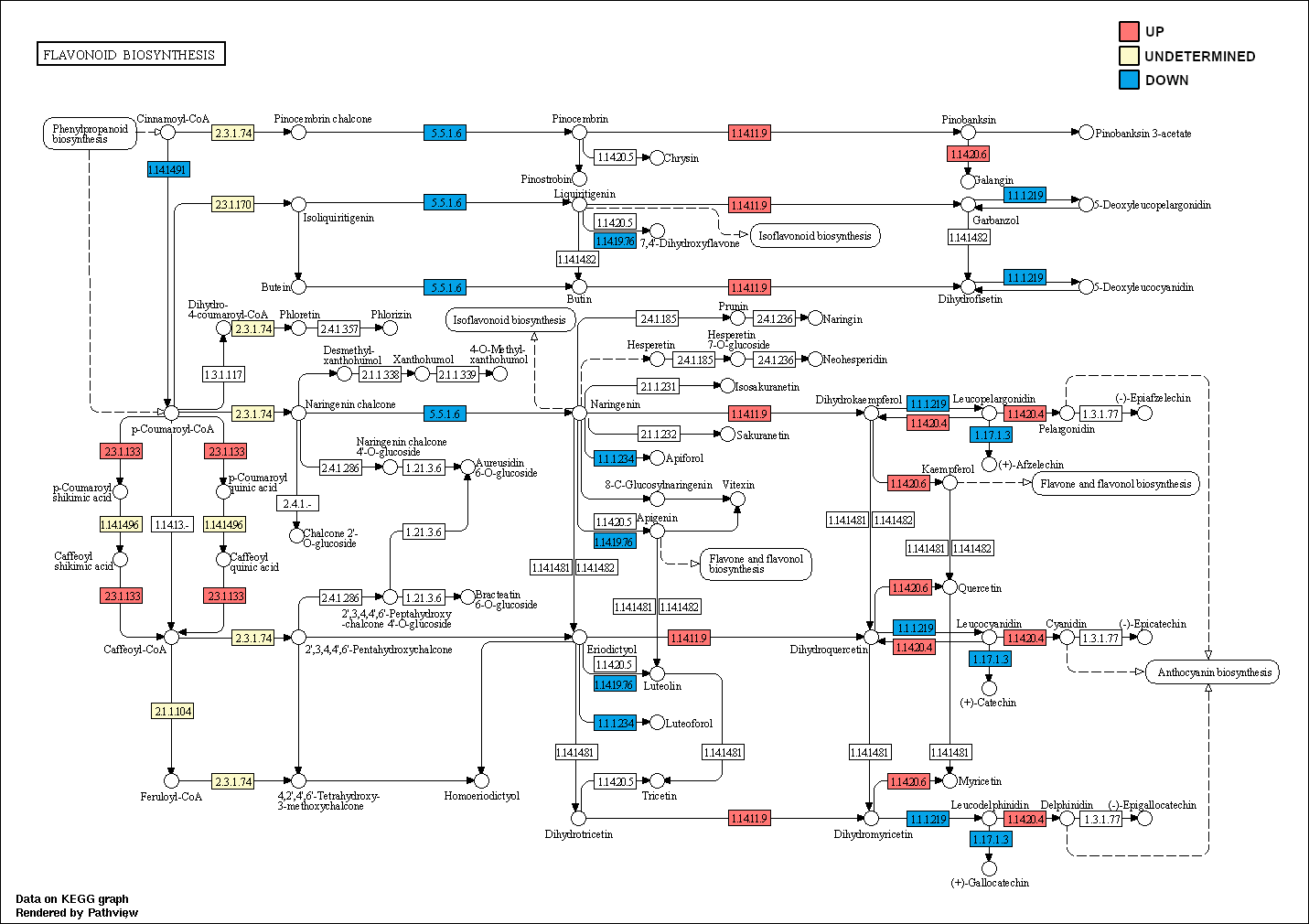

Supplement: Supplementary file 2 [file Data_Sheet_2.ZIP › Supplementary_Figure_4/Supplementary_Figure_4.082.png]

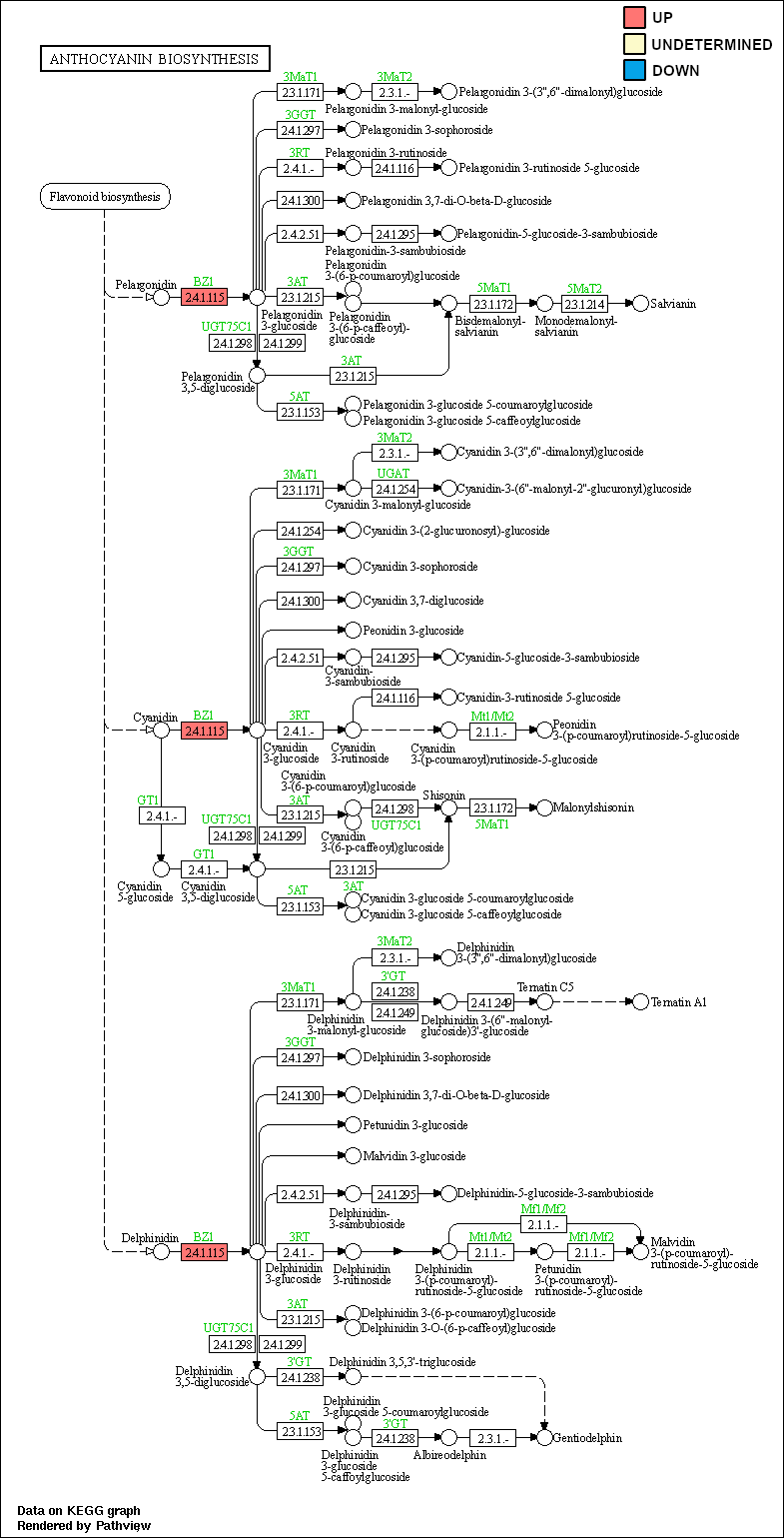

Supplement: Supplementary file 2 [file Data_Sheet_2.ZIP › Supplementary_Figure_4/Supplementary_Figure_4.083.png]

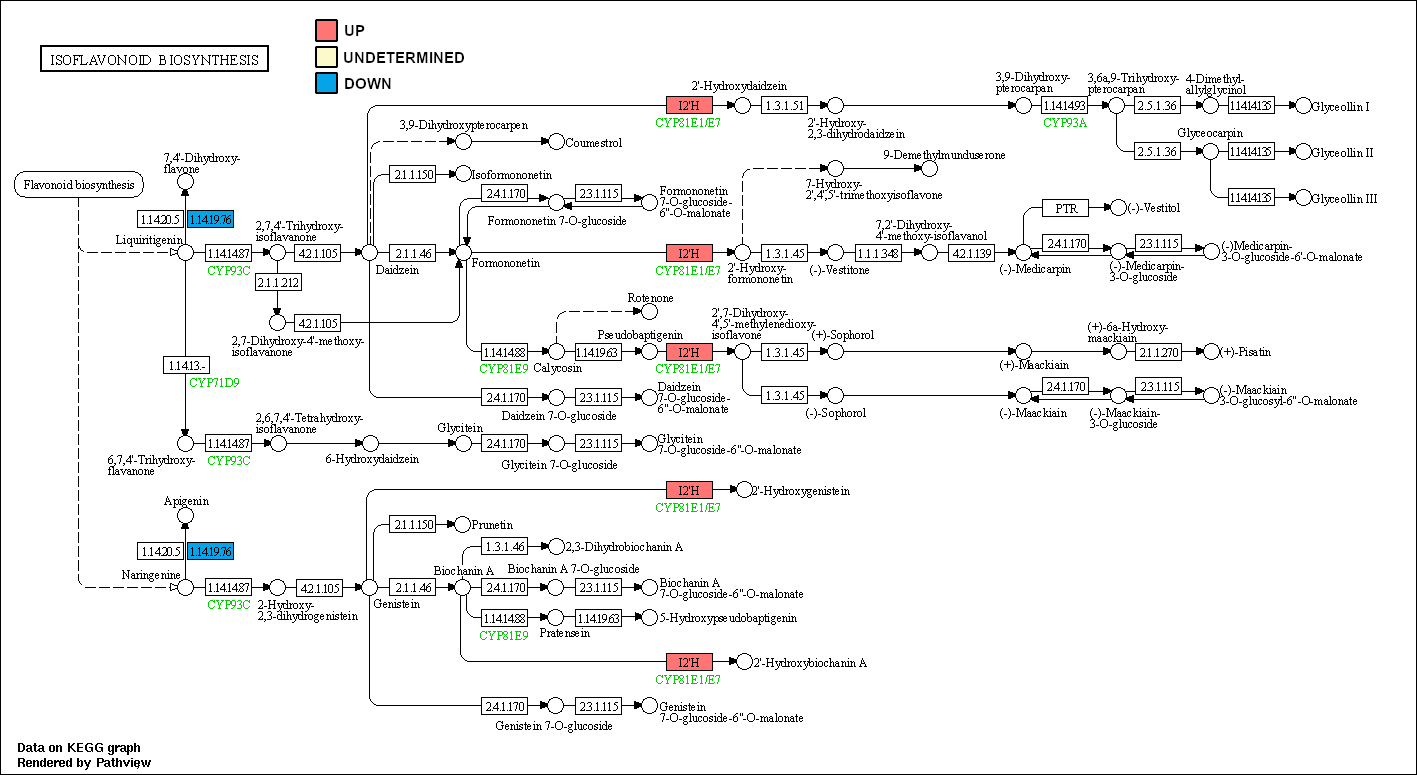

Supplement: Supplementary file 2 [file Data_Sheet_2.ZIP › Supplementary_Figure_4/Supplementary_Figure_4.084.png]

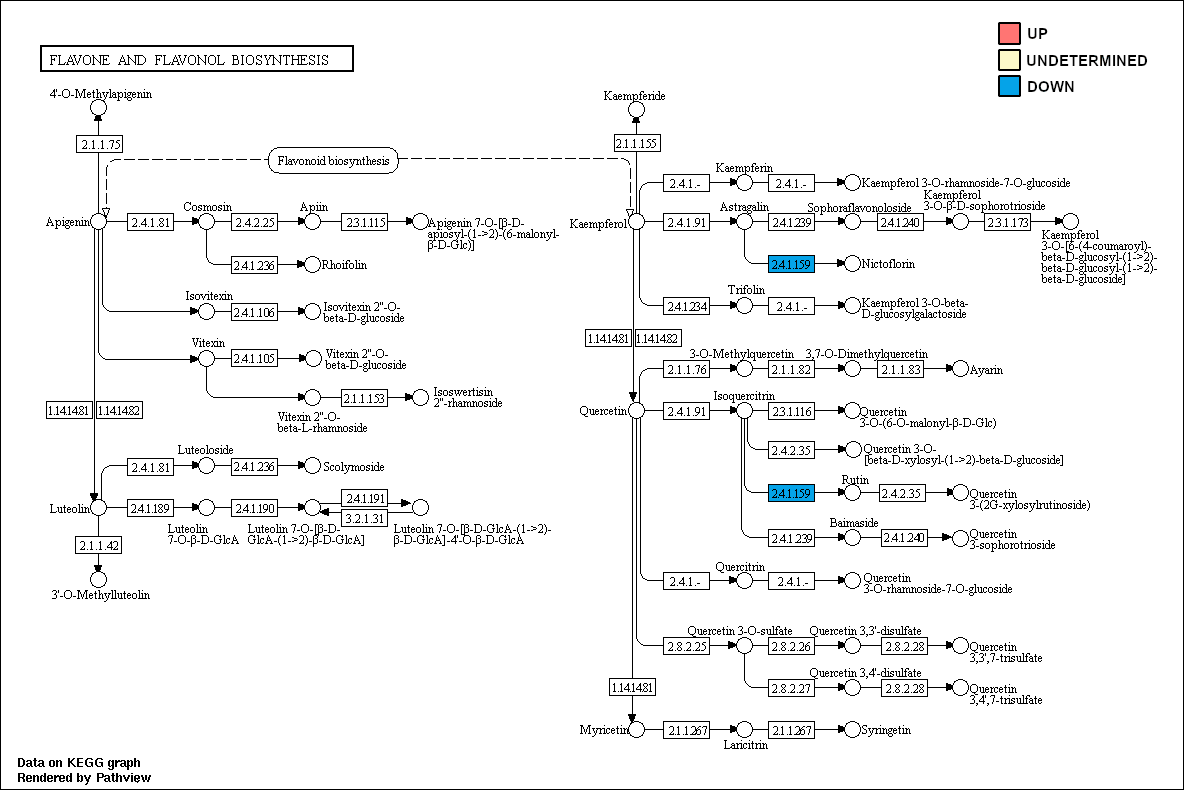

Supplement: Supplementary file 2 [file Data_Sheet_2.ZIP › Supplementary_Figure_4/Supplementary_Figure_4.085.png]

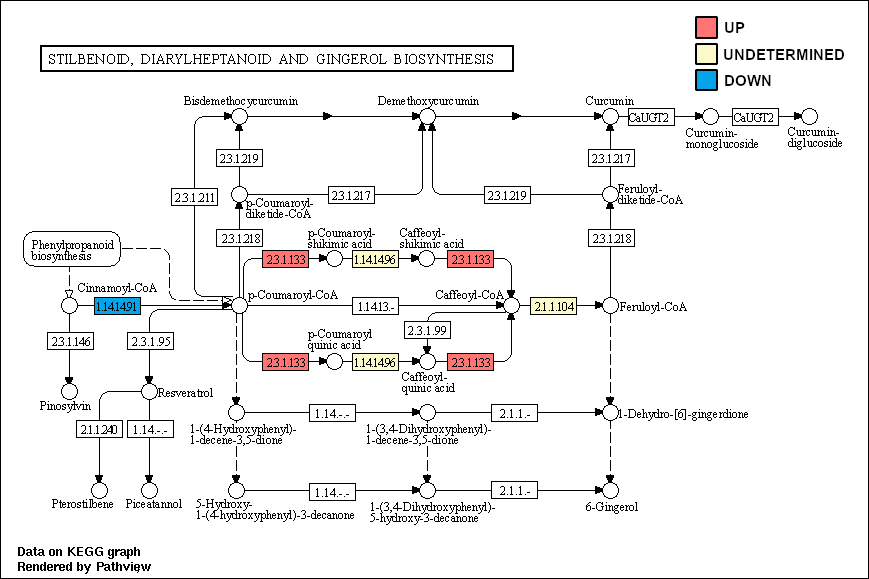

Supplement: Supplementary file 2 [file Data_Sheet_2.ZIP › Supplementary_Figure_4/Supplementary_Figure_4.086.png]

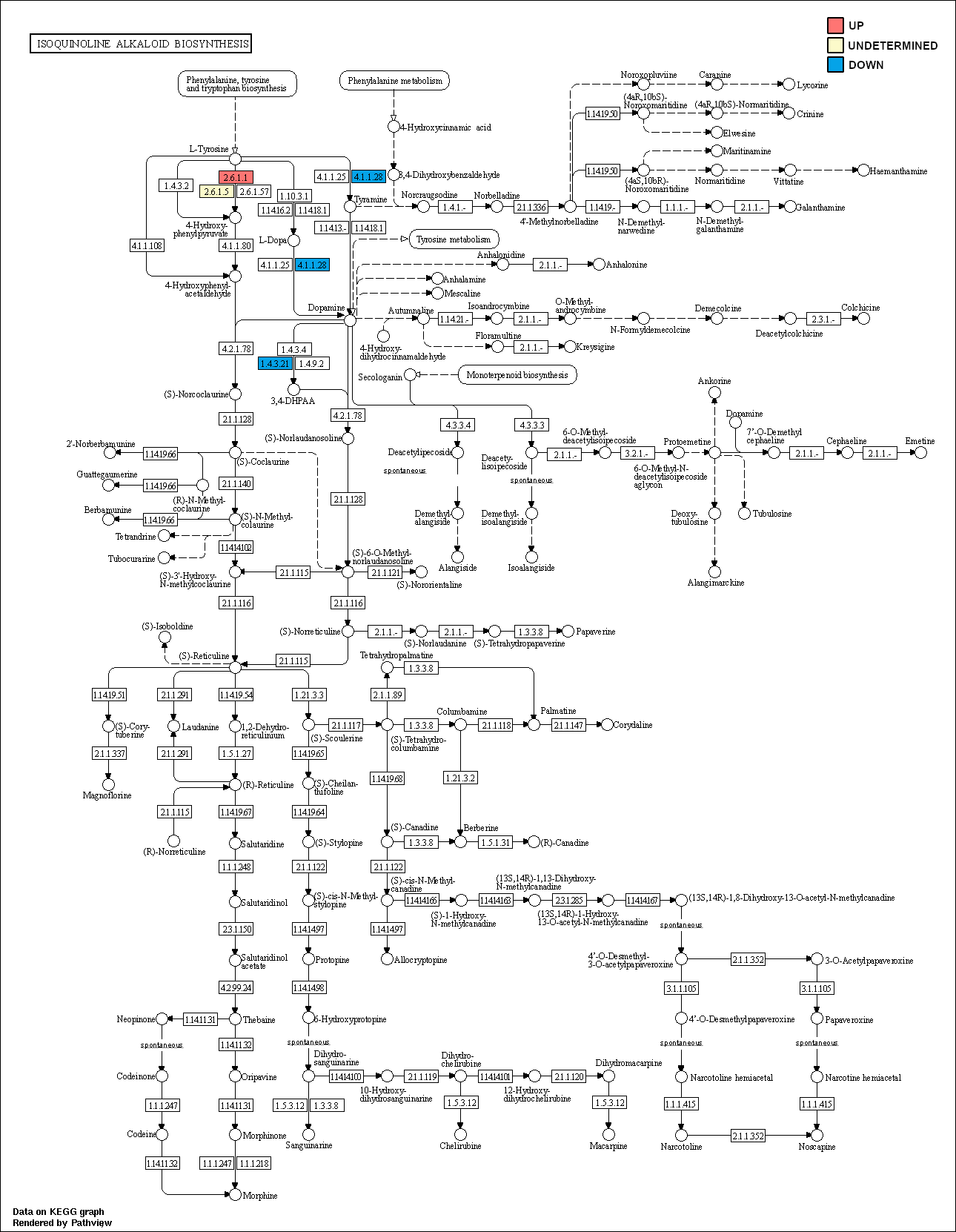

Supplement: Supplementary file 2 [file Data_Sheet_2.ZIP › Supplementary_Figure_4/Supplementary_Figure_4.087.png]

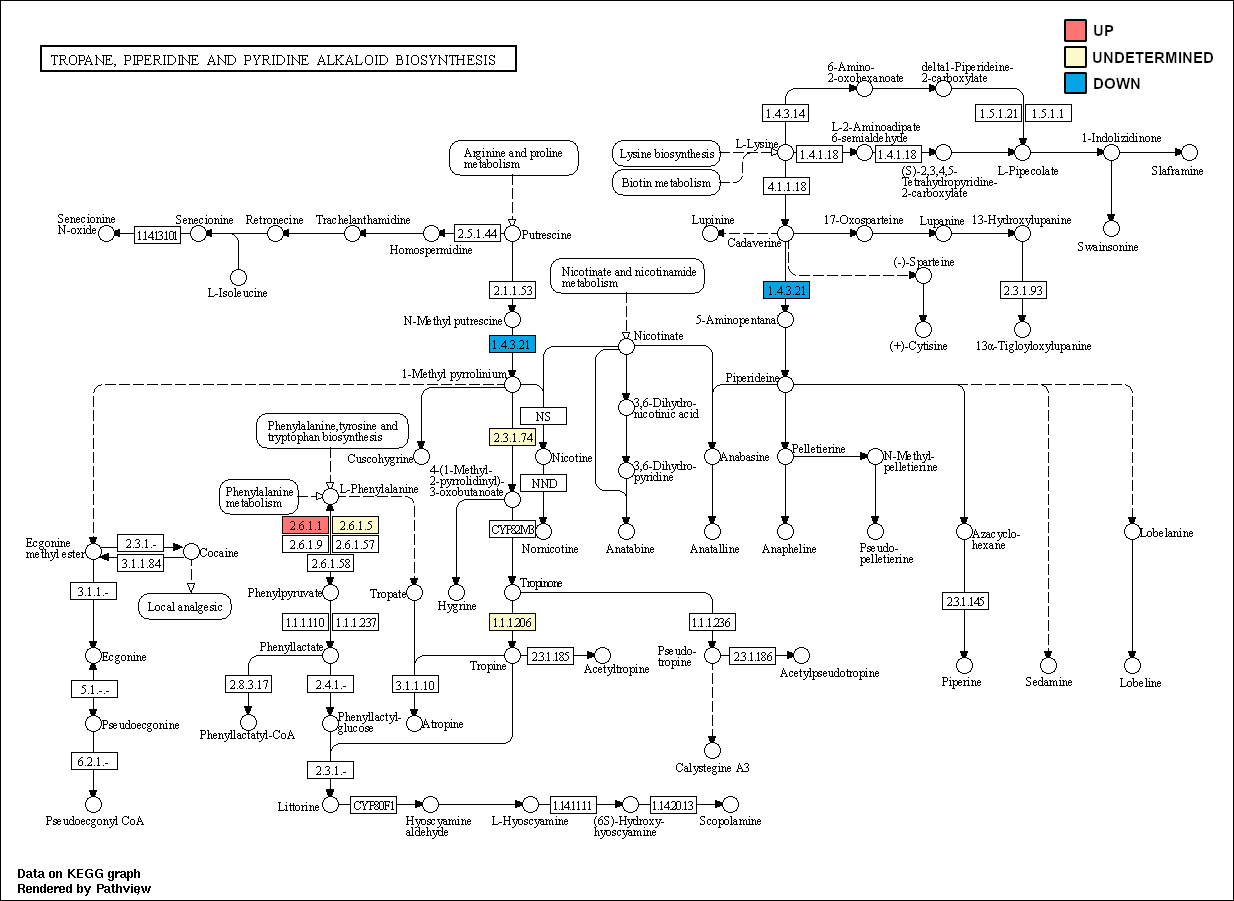

Supplement: Supplementary file 2 [file Data_Sheet_2.ZIP › Supplementary_Figure_4/Supplementary_Figure_4.088.png]

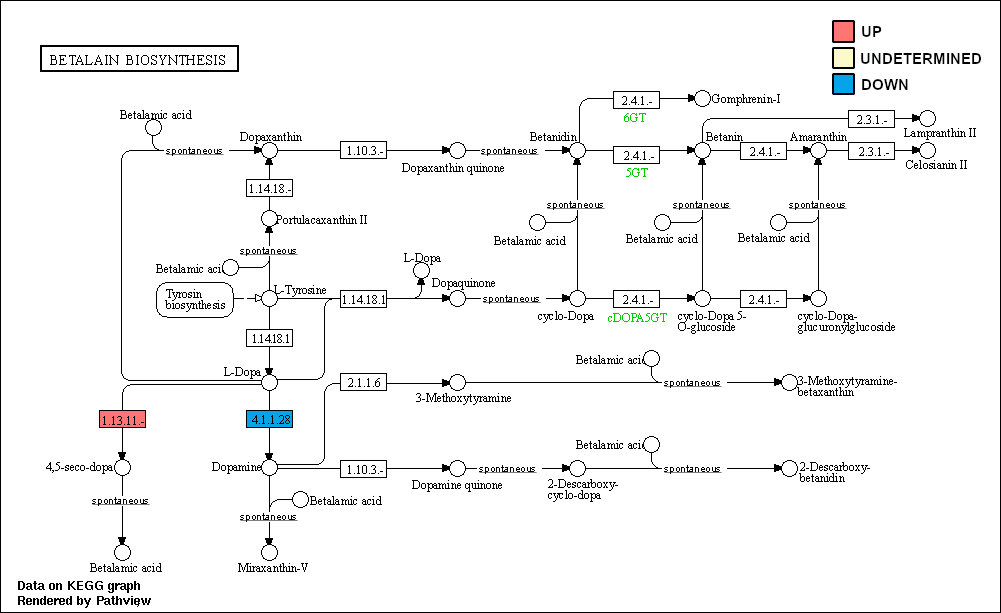

Supplement: Supplementary file 2 [file Data_Sheet_2.ZIP › Supplementary_Figure_4/Supplementary_Figure_4.089.png]

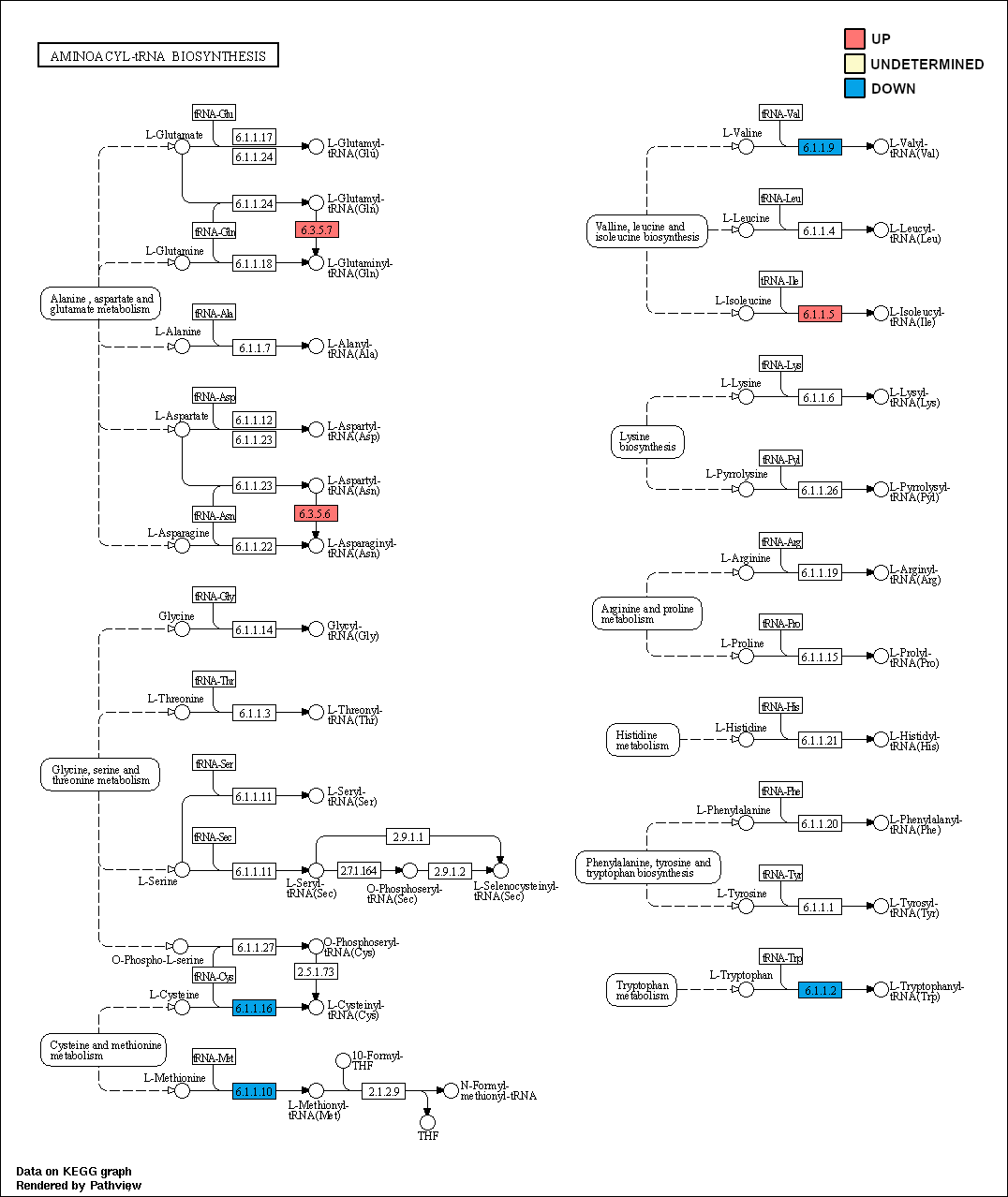

Supplement: Supplementary file 2 [file Data_Sheet_2.ZIP › Supplementary_Figure_4/Supplementary_Figure_4.090.png]

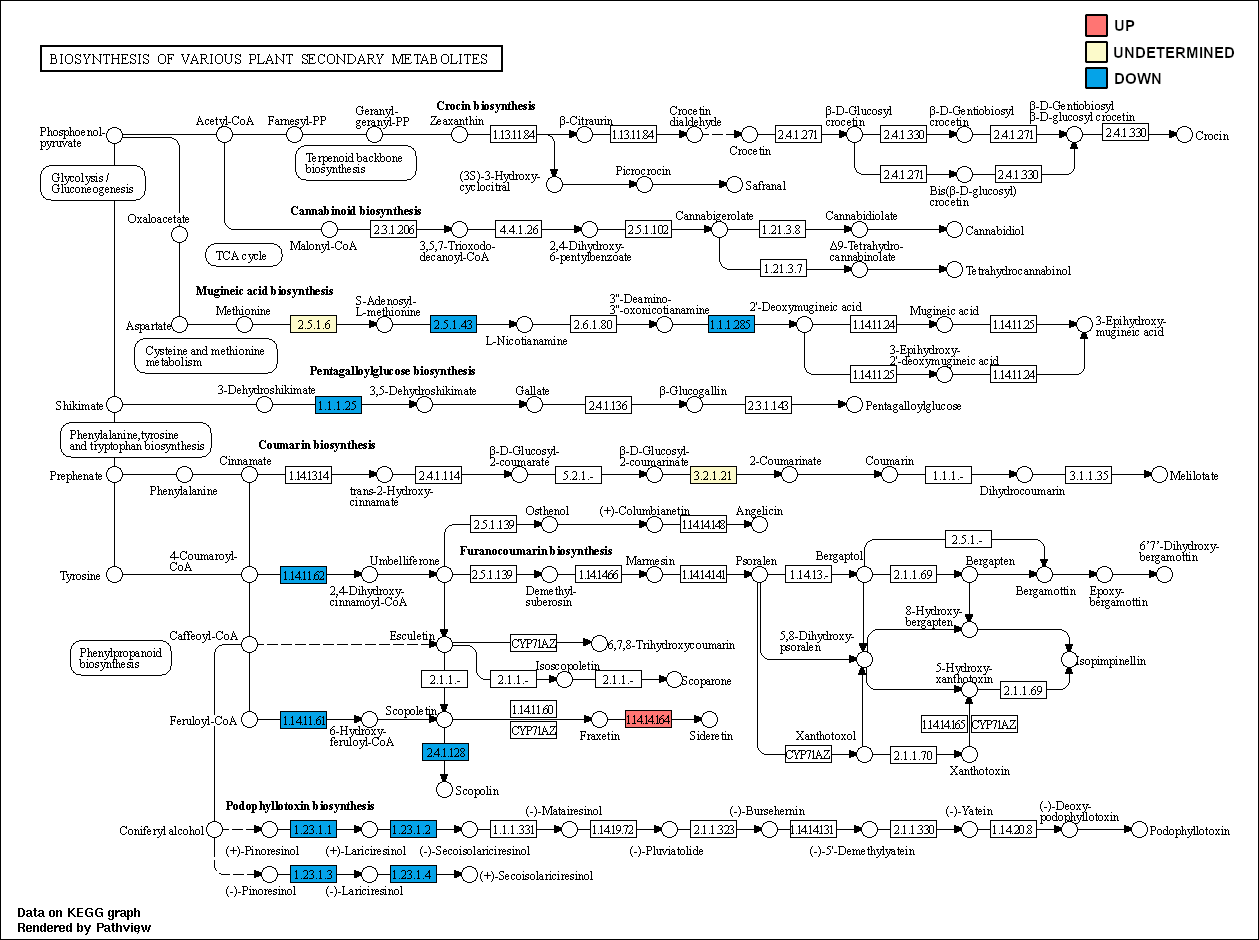

Supplement: Supplementary file 2 [file Data_Sheet_2.ZIP › Supplementary_Figure_4/Supplementary_Figure_4.091.png]

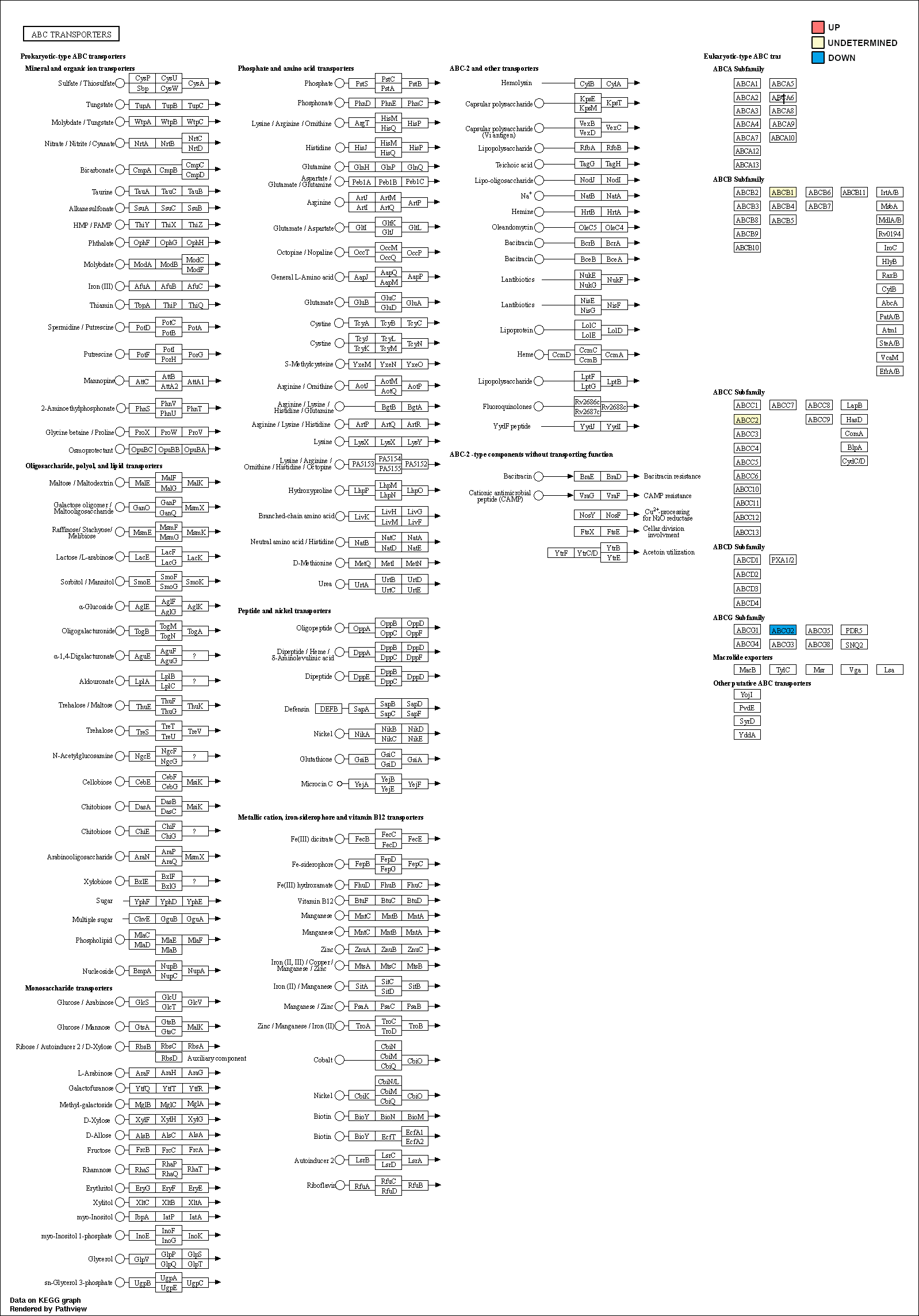

Supplement: Supplementary file 2 [file Data_Sheet_2.ZIP › Supplementary_Figure_4/Supplementary_Figure_4.092.png]

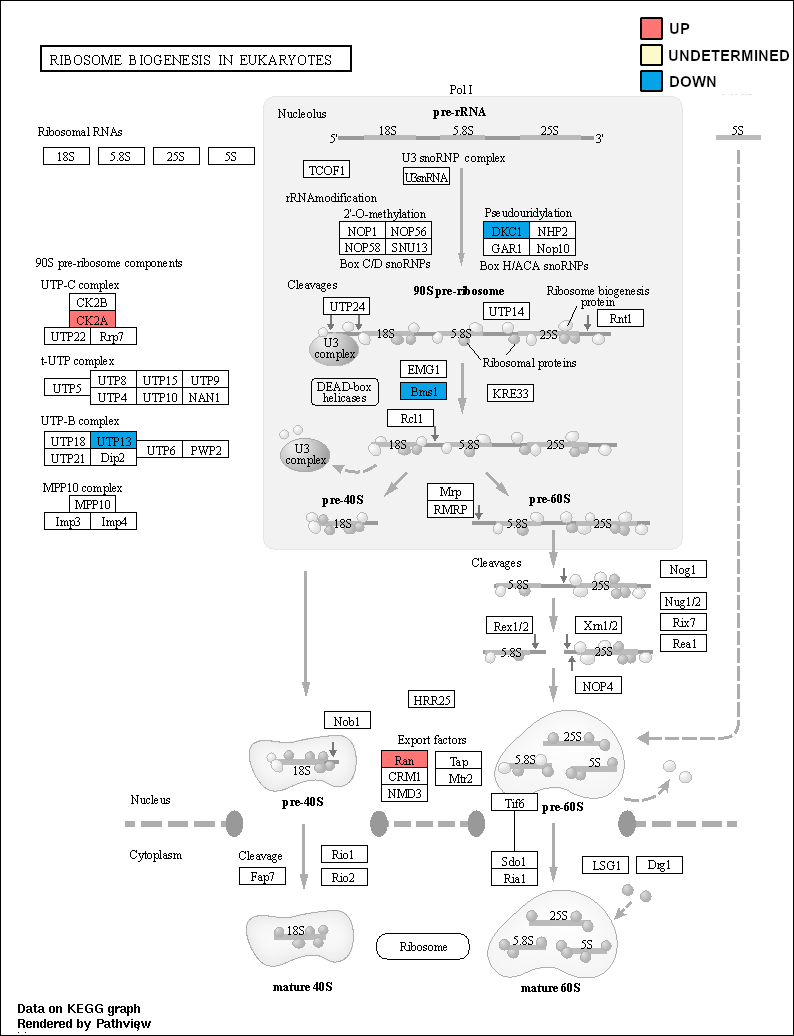

Supplement: Supplementary file 2 [file Data_Sheet_2.ZIP › Supplementary_Figure_4/Supplementary_Figure_4.093.png]

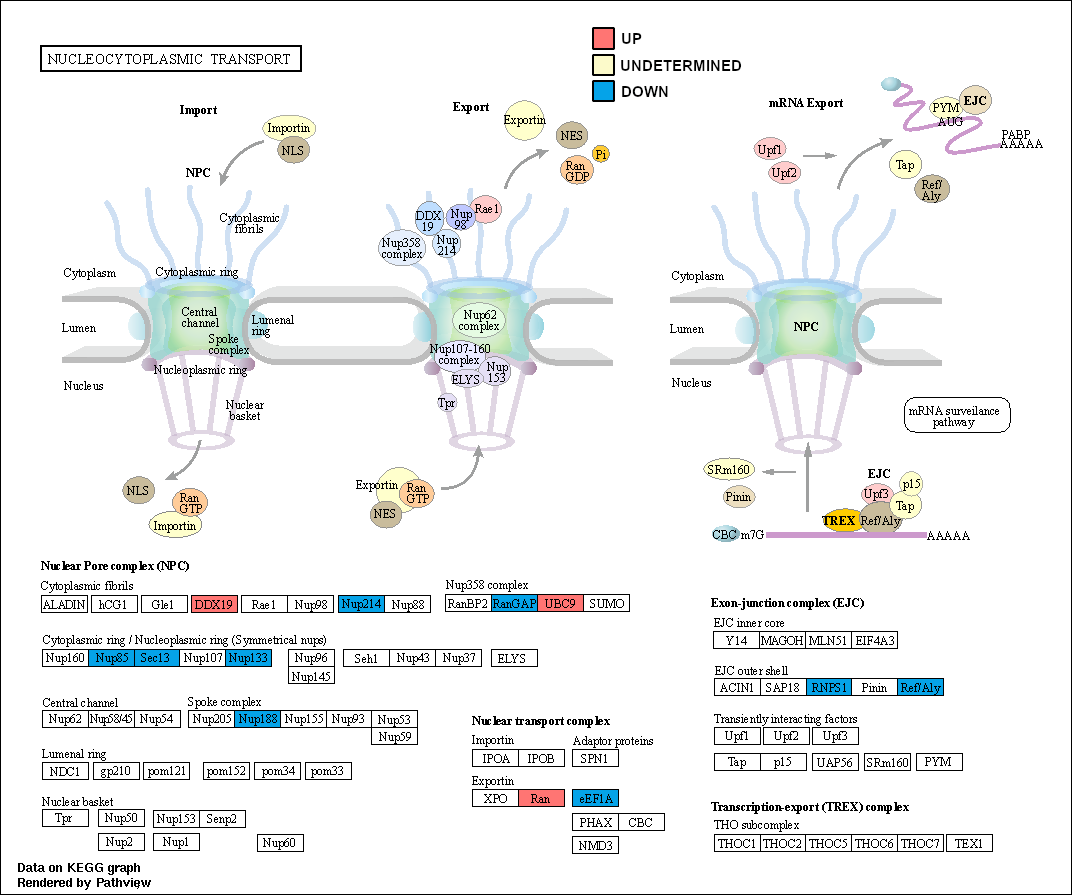

Supplement: Supplementary file 2 [file Data_Sheet_2.ZIP › Supplementary_Figure_4/Supplementary_Figure_4.094.png]

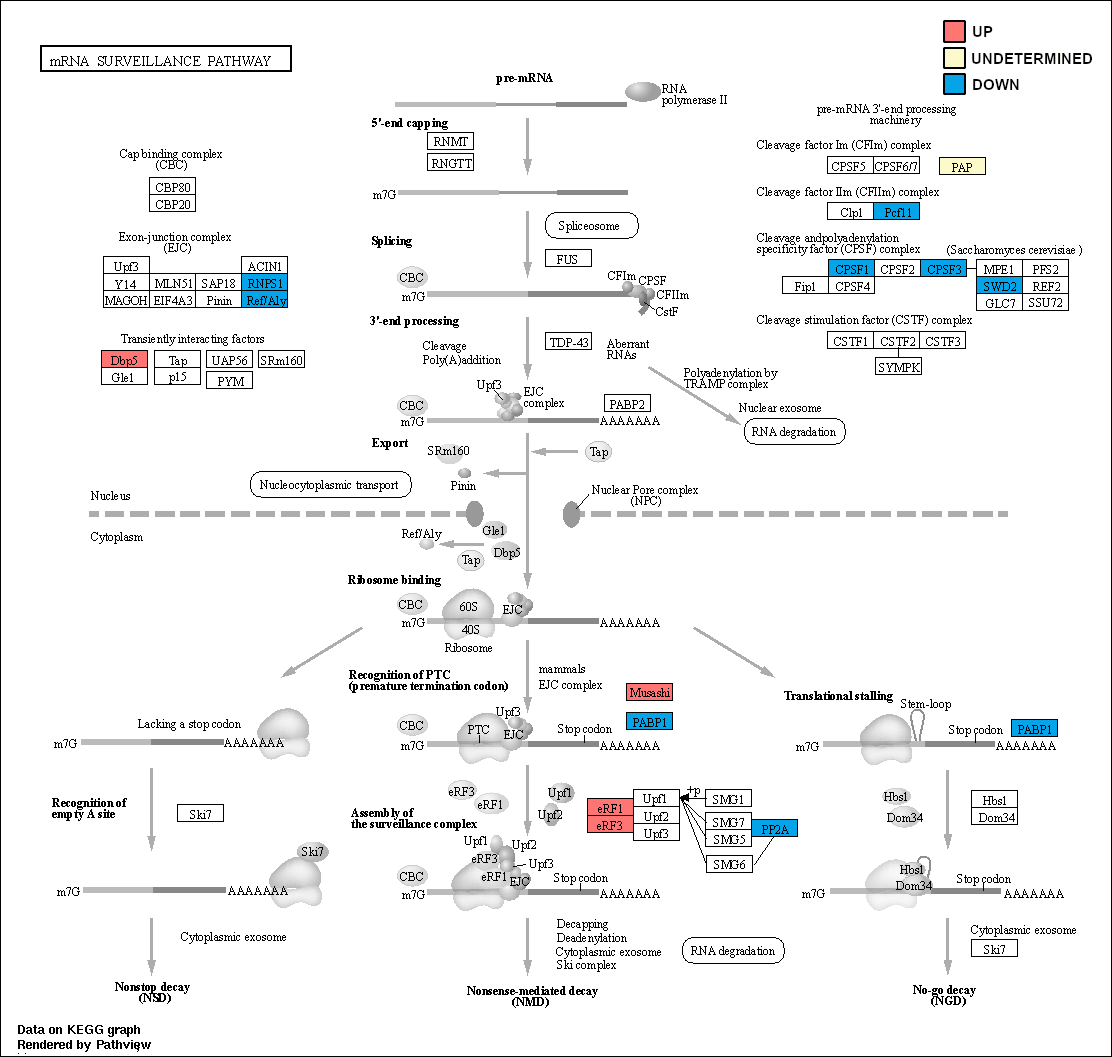

Supplement: Supplementary file 2 [file Data_Sheet_2.ZIP › Supplementary_Figure_4/Supplementary_Figure_4.095.png]

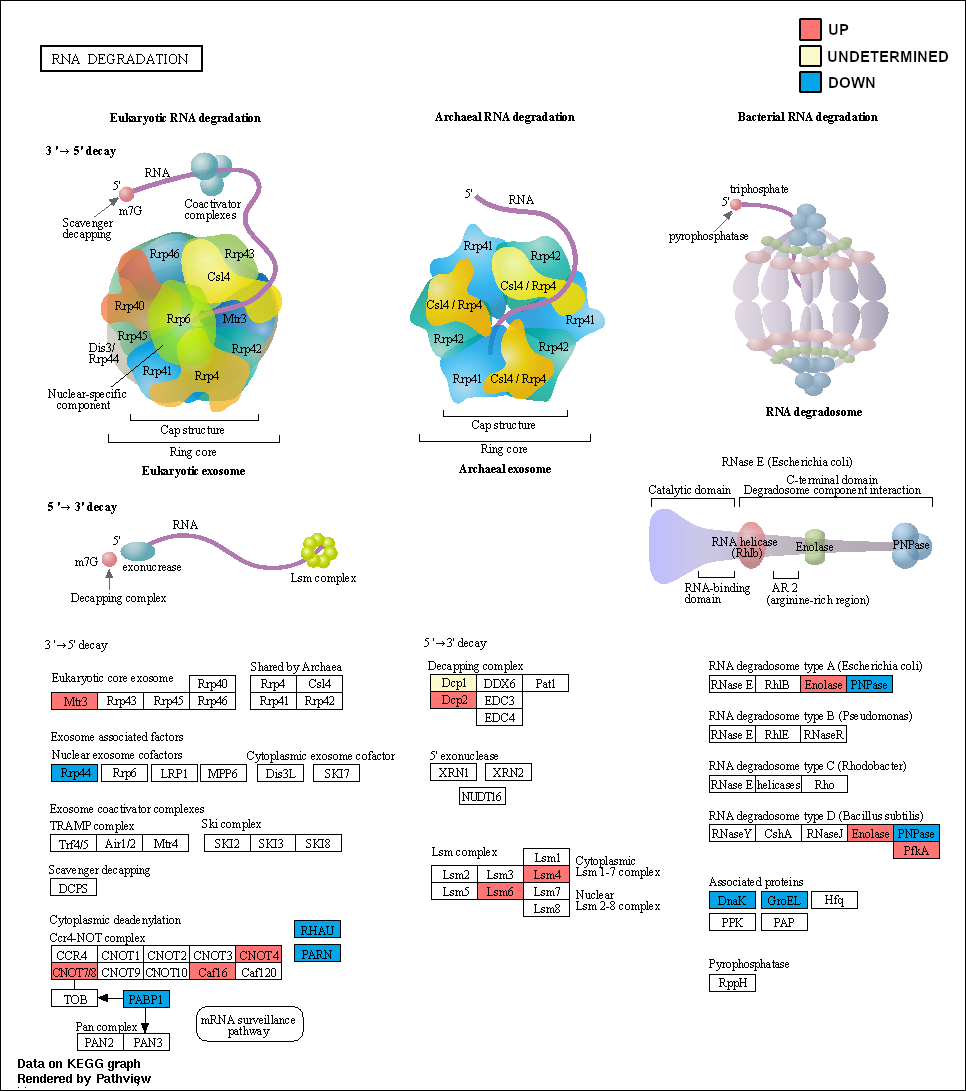

Supplement: Supplementary file 2 [file Data_Sheet_2.ZIP › Supplementary_Figure_4/Supplementary_Figure_4.096.png]

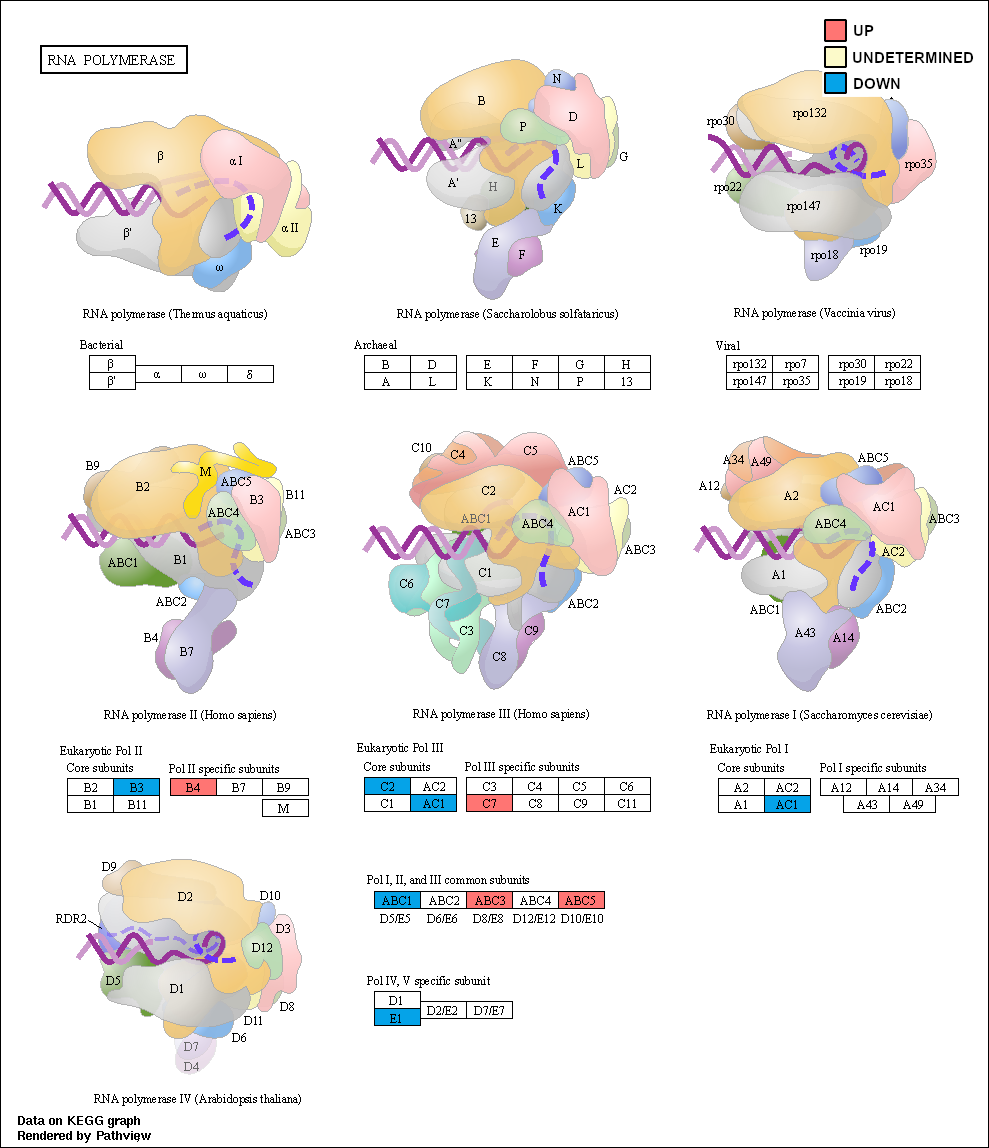

Supplement: Supplementary file 2 [file Data_Sheet_2.ZIP › Supplementary_Figure_4/Supplementary_Figure_4.097.png]

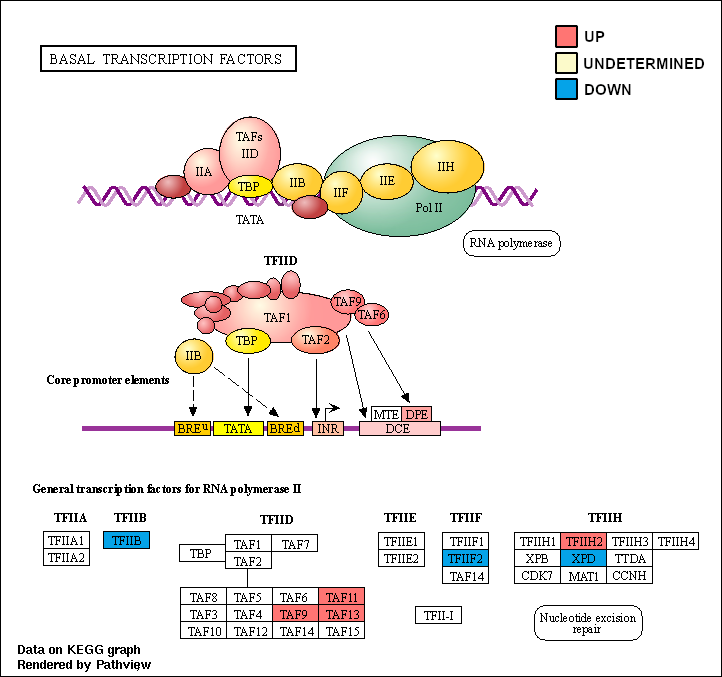

Supplement: Supplementary file 2 [file Data_Sheet_2.ZIP › Supplementary_Figure_4/Supplementary_Figure_4.098.png]

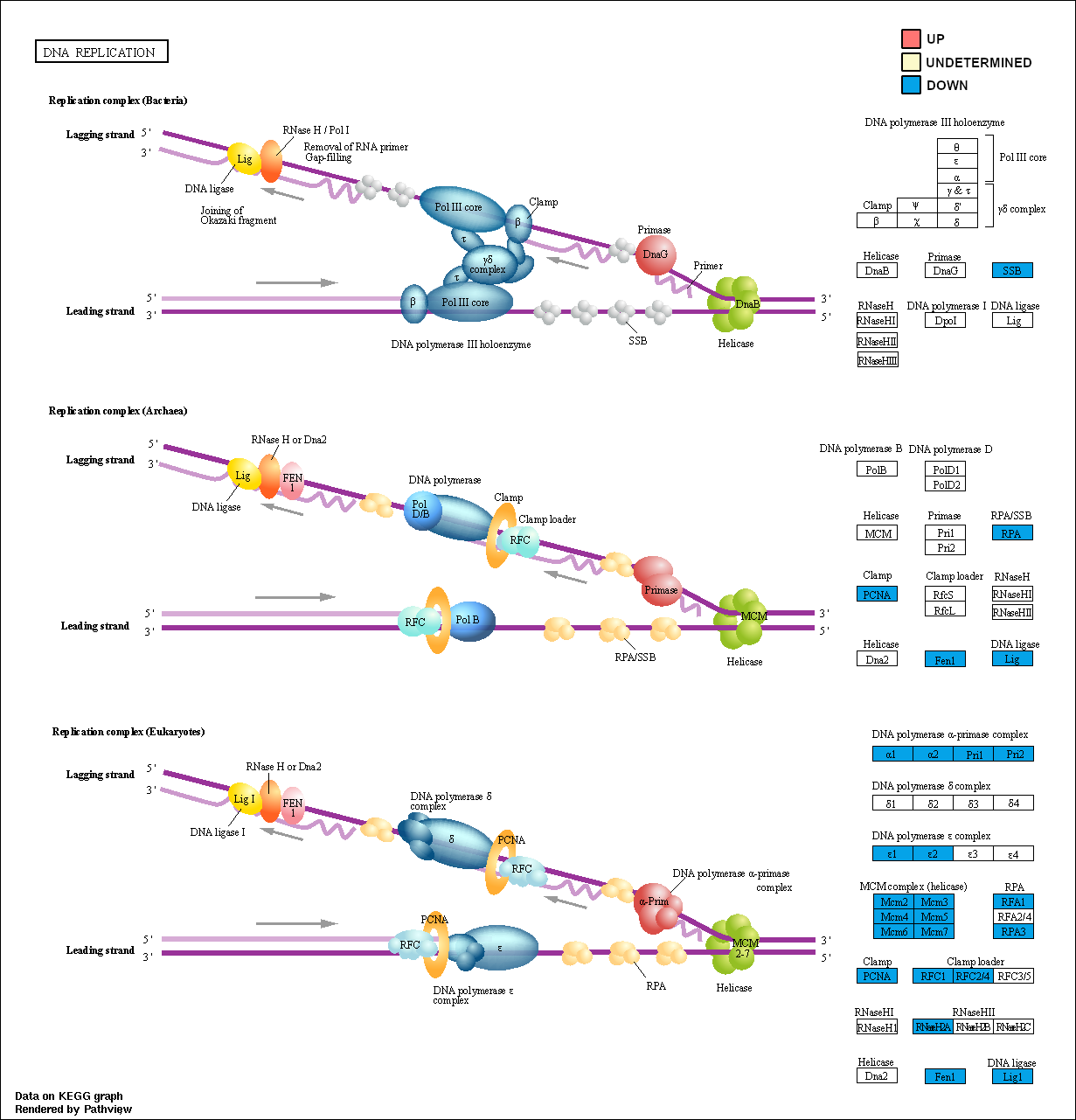

Supplement: Supplementary file 2 [file Data_Sheet_2.ZIP › Supplementary_Figure_4/Supplementary_Figure_4.099.png]

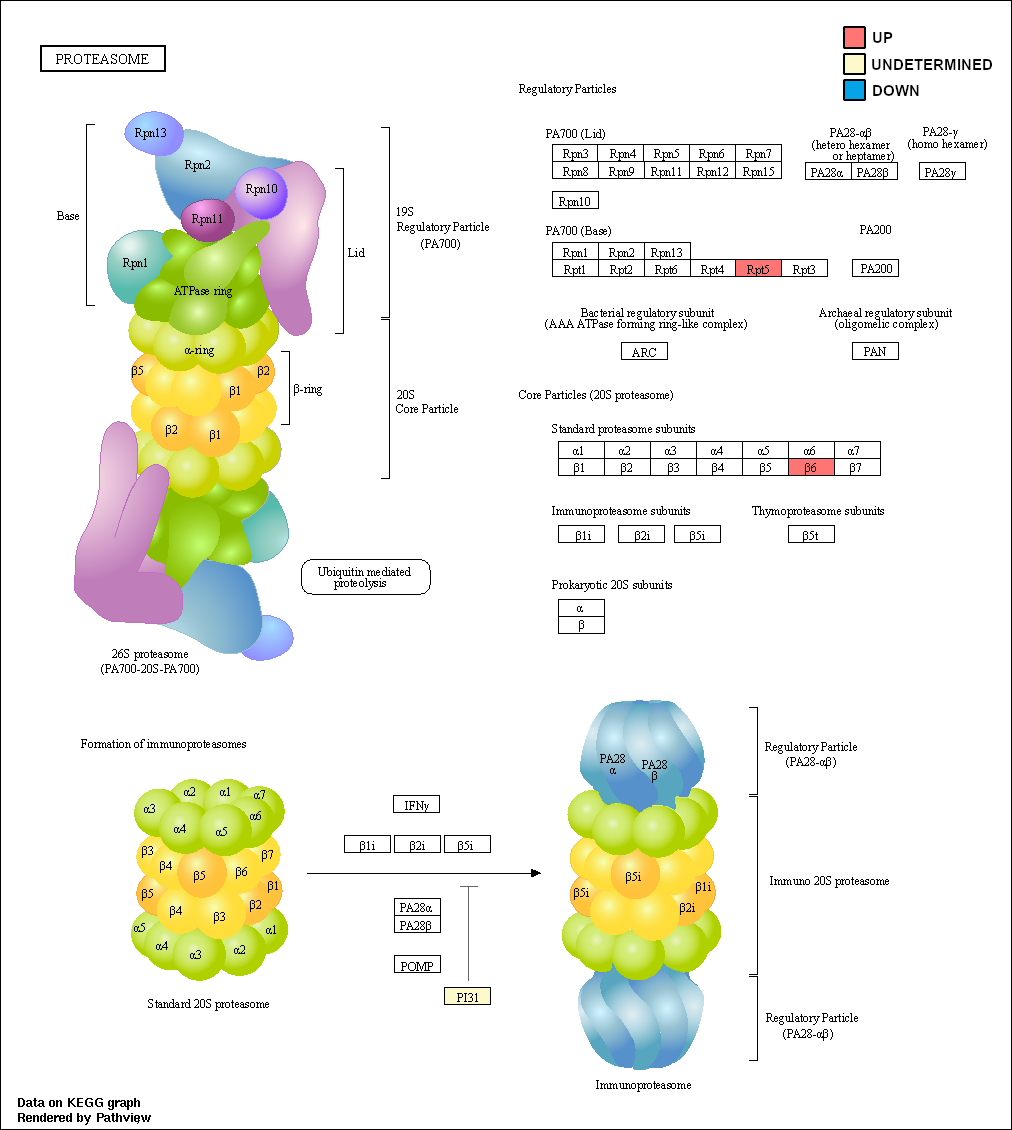

Supplement: Supplementary file 2 [file Data_Sheet_2.ZIP › Supplementary_Figure_4/Supplementary_Figure_4.100.png]
